# Identification of human MLKL Cys184 and HSPBP1 Cys201 as novel cellular targets for necroptosis

**DOI:** 10.1038/s41419-026-08764-4

**Published:** 2026-04-22

**Authors:** Hongming Shao, Jiabin Wu, Qianyu Han, Lijuan Xu, Pengcheng Dai, Rui Wang, Jiao Li, Wenbin Wu, Yanan Hao, Ruilin Hou, Yue Chai, Zhi Cheng, Pei Wang, Lei Xue, Ting Han, Chunlin Zhuang

**Affiliations:** 1 The Center for Basic Research and Innovation of Medicine and Pharmacy (MOE), School of Pharmacy, Naval Medical University/Second Military Medical University, Shanghai, China; 2 Department of Thoracic Surgery, The Second Affiliated Hospital of Naval Medical University (Changzheng Hospital), Shanghai, China; 3 School of Chemistry and Chemical Engineering, Henan Normal University, Xinxiang, China

**Keywords:** Natural products, Necroptosis

## Abstract

Necroptosis has been definitively confirmed as a caspase-deficient, non-apoptotic cellular mechanism that exhibits a profound connection to inflammatory disorders. The receptor-interacting protein kinase 1 (RIPK1), RIPK3, and mixed-lineage kinase domain-like protein (MLKL Cys86) have been recognized as three main targets for necroptosis for many years. Here, we report HSPBP1 Cys201 and MLKL Cys184 as new cellular targets for necroptosis in human cells. Parthenolide, a natural sesquiterpene lactone, was first confirmed to have anti-necroptotic activity and effectively alleviated the necroptosis-induced systemic inflammatory response syndrome and abdominal aortic aneurysm (AAA) in mice. In the elastase-induced mouse AAA model, MLKL deficiency is highlighted as attenuating AAA formation. HSPBP1 Cys201 was identified to be an upstream target contributing to the anti-necroptotic activity. Co-incubated with purified HSPBP1, followed by mass spectrometry analysis, confirmed that PTL binds to HSPBP1 at Cys201, while HSPBP1 knockdown conferred a certain degree of resilience to necroptosis. Human MLKL Cys184 was discovered as another novel anti-necroptotic target in human HT-29 cells. The human MTRP and molecular dynamics results suggested that Cys184 is the potential binding site between PTL and MLKL. Our co-incubation experiments of PTL with MLKL further demonstrated that PTL can interact with the sulfhydryl group of MLKL Cys184 via covalent modification. These findings yield important insights into the complex regulatory mechanisms of necroptosis and, concurrently, underscore the therapeutic potential of PTL and its derivatives for treating AAA.

## Introduction

Necroptosis, a caspase-independent regulated cell death pattern, has been definitively associated with the pathogenesis of various inflammatory diseases [1, 2]. The concept was first proposed by the Yuan group in 2005 [3], along with an inhibitor named necrostatin-1 (Nec-1), which was then characterized to target receptor-interacting protein 1 (RIPK1) [4]. In 2009, RIPK3 was then identified as another crucial activator for necroptosis by a genome-wide siRNA screen [5–7]. In 2012, the Wang group applied a chemical genetics approach to identify necrosulfonamide (NSA) as a specific necroptosis inhibitor in human cells [8]. Further target identification works demonstrated that NSA covalently targets the Cys86 of mixed lineage kinase domain-like protein (MLKL) [9]. Subsequently, multiple necroptosis inhibitors targeting RIPK1, RIPK3, and MLKL, with disease treatment functions, have been characterized [10–12]. Based on these chemical biology findings, the necroptosis signaling is gradually becoming clear. Upon the necroptosis initiation with stimulus triggering, a sequential activation cascade ensues, featuring the involvement of RIPK1, RIPK3, and MLKL. MLKL is the terminal protein in the pro-inflammatory necroptotic cell death program. RIPK3-mediated phosphorylation is thought to initiate MLKL oligomerization, membrane translocation, and membrane disruption, although the precise choreography of events is incompletely understood [13].

Natural products (NPs) serve as invaluable sources for the identification of active lead compounds and novel therapeutic targets [14, 15]. Despite the increasing number of necroptosis inhibitors, the naturally occurring inhibitors are still limited. In 2016, the Lei group discovered a natural product named kongensin A4 that emerged as a highly effective inhibitor of necroptosis [16]. They subsequently harnessed a bioorthogonal ligation technique to pinpoint heat shock protein 90 (HSP90) Cys420 residue as the key target of this natural compound. Thereby, the compound could disrupt the interaction between HSP90 and its co-chaperone cell division cycle 37 (CDC37), leading to inhibition of RIPK3 activation. HSP90 can be blocked by inhibitors to prevent necroptosis at all levels – RIPK1, RIPK3, and MLKL [17, 18]. In the study, the expressions of RIPK1, RIPK3, p-RIPK3, MLKL, and p-MLKL proteins were decreased with 17-DMAG treatment [17, 18]. In 2019, the Yuan group found an oleanolic acid derivative, known as 2-amino-5-chloro-N,3-dimethylbenzamide (CDDO), possessing necroptosis and ferroptosis inhibitory activity by targeting HSP90 to induce RIPK1 inhibition and glutathione peroxidase 4 (GPX4) degradation [19]. These findings highlight the potential of chaperone proteins as viable targets for modulating cell death pathways, while indicating that NPs have certain significance in uncovering novel intracellular signaling targets associated with cell death mechanisms [20].

In the present study, we, for the first time, found that a natural sesquiterpene lactone parthenolide (PTL) could enable the anti-necroptotic activity. We generate several PTL derivatives and probes, possessing activity against cell necroptosis, thereby highlighting the role of this natural molecule as a key necroptosis signaling step. Furthermore, applying PTL as a tool, we present chemical proteomics reveal HSPBP1 as an upstream target of necroptosis both in murine and human systems. We also identify MLKL Cys184 as a downstream cellular target in human cells, undergoing a new covalent modification with PTL and derivatives. Overall, these data establish MLKL Cys184 and HSPBP1 Cys201 as new regulated sites in the signaling cascade of necroptosis.

## Results

### Parthenolide is a novel necroptosis inhibitor

**Fig. 1:**
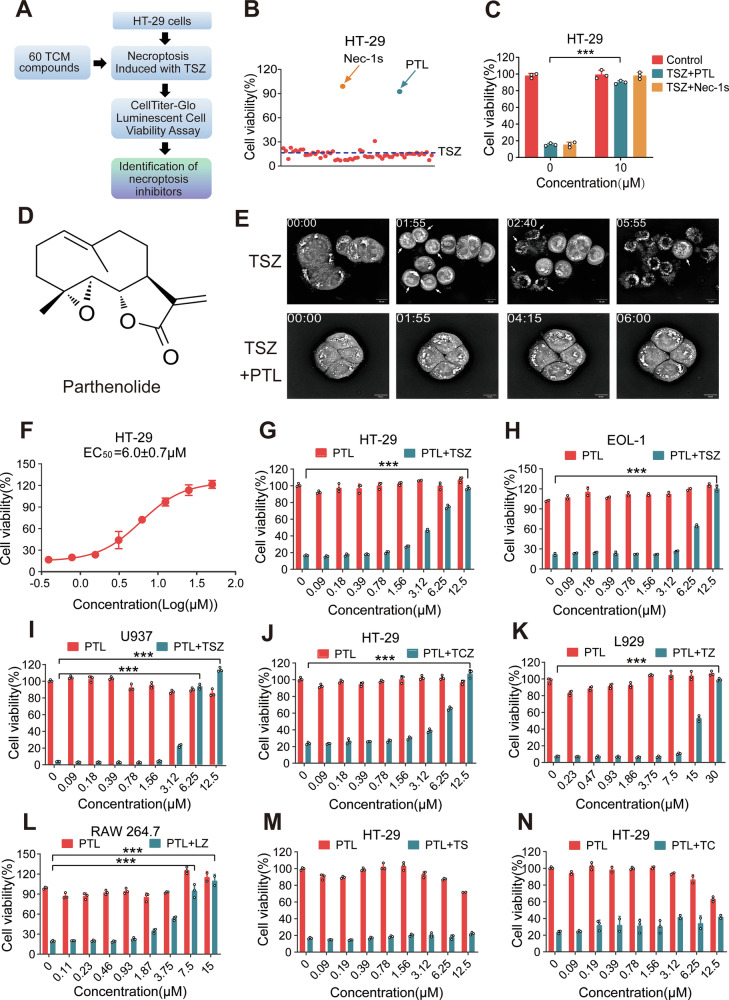
Identification of parthenolide as a natural necroptosis inhibitor. **A** Flowchart for screening the anti-necroptotic activity of natural products. TCM means Traditional Chinese Medicine. **B**, **C** Cell viability of each compound (10 μM) in the TSZ model of HT-29 cells. Nec-1s was used as the positive control compound. ***P < 0.001, versus TSZ. *N* = 3 biologically independent samples. **D** The chemical structure of PTL. **E** Live-cell imaging of HT-29 cells treated with TSZ or TSZ + PTL (10 μM). The arrows indicated the swollen cell morphology (01:55), the cellular debris after rupture (02:40), and the instant of cell rupture (05:55). Scale bar, 10 μm. **F** The determination of the EC_50_ value of PTL in the TSZ model of HT-29 cells. **G** HT-29 cells treated with PTL or TSZ + PTL. PTL/TSZ + PTL (0 μM) represented the control and TSZ groups. Cell viability in each group was expressed as the ratio of the luminescence value to that of the control group. ***P < 0.001, versus TSZ. *N* = 3 biologically independent samples. **H**, **I** EOL-1 and U937 cells treated with PTL or TSZ + PTL. ***P < 0.001, versus TSZ. *N* = 3 biologically independent samples. **J** HT-29 cells treated with PTL or TCZ + PTL. ***P < 0.001, versus TCZ. *N* = 3 biologically independent samples. **K** L929 cells treated with PTL or TZ + PTL. ***P < 0.001, versus TZ. *N* = 3 biologically independent samples. **L** RAW264.7 cells treated with PTL or LZ + PTL. ***P < 0.001, versus LZ. *N* = 3 biologically independent samples. **M** HT-29 cells treated with PTL or TS + PTL. *N* = 3 biologically independent samples. **N** HT-29 cells treated with PTL or TC + PTL. *N* = 3 biologically independent samples.

We first screened 60 natural compounds for anti-necroptotic activity through a TSZ (TNFα, SM164, and z-VAD-FMK)-induced necroptosis model in human colon cancer HT-29 cells. The cell viability was measured by CellTiter-Lumi™ Luminescent cell viability assay (Fig. 1A). Parthenolide (PTL) was found to have relatively prominent anti-necroptotic activity as compared to other compounds (Fig. 1B). At a dose of 10 μM, PTL exhibited a protective effect comparable to that of the positive control compound Nec-1s (Fig. 1C), a classic RIPK1 inhibitor [3, 4]. PTL is a sesquiterpene lactone (Fig. 1D) extracted from feverfew (*Tanacetum parthenium*) with good anti-inflammatory and anti-tumor properties [21]. Necroptosis in HT-29 cells was induced by TSZ for 12 h, and cells pretreated with PTL did not rupture compared with the TSZ model (Fig. 1E). Using a live-cell super-resolution panoramic microscopy, we observed and recorded the entire process of necroptosis in HT-29 cells (Movies S1 and S2). Approximately 2 h after TSZ treatment, the cells began to shrink and become rounded. Approximately 3 h later, the cells began to lose their structural integrity, ultimately culminating in cell death, with their contents becoming fully exposed. At the time point of 6 h, all cells underwent necrosis, with the final image capturing the precise moment of cellular rupture (Movie S1). Meanwhile, the cells treated with PTL did not exhibit significant morphological changes (Movie S2). PTL could dose-dependently inhibit TSZ-induced necroptosis with a half maximal effective concentration (EC_50_) of about 6.0 μM (Fig. 1F, G).

Next, we examined the anti-necroptotic effect in multiple cellular models of necroptosis. We found that PTL could inhibit TSZ-induced necroptosis in human eosinophilic leukemia cell line EOL-1 (Fig. 1H) and human histiocytic lymphoma cell line U937 (Fig. 1I). Similarly, in a TCZ (TNF-α, cycloheximide, and z-VAD-FMK)-induced necroptosis, PTL blocked cell death in a dose-dependent manner (Fig. 1J). Additionally, in murine fibroblast cell line L929 and monocyte/macrophage-like cell line RAW264.7, PTL dose-dependently protected cells against TZ (TNFα, z-VAD-FMK)- or LZ (LPS, z-VAD-FMK)-induced necroptosis, respectively (Fig. 1K, L). These data collectively suggest that PTL functions as an efficacious natural inhibitor of necroptosis across multiple cellular models.

To initially assess the impact of PTL on signaling pathways, we investigated its role in apoptosis induced by TS (TNF-α, SM164) and TC (TNF-α, cycloheximide). Results showed that PTL did not exhibit an inhibitory effect on apoptosis in HT-29 cells (Fig. 1M, N). From a signal transduction standpoint, the necroptosis triggered by TSZ and the apoptosis induced by TS share common signaling pathways, as necroptosis occurs when apoptosis is inhibited by the presence of z-VAD-FMK [10, 22]. Our findings suggested that PTL potentially operated by specifically targeting the distinct pathway of necroptosis, rather than the shared pathway common to both necroptosis and apoptosis.

### PTL effectively alleviates the necroptosis-induced systemic inflammatory response syndrome and abdominal aortic aneurysm in mice

**Fig. 2:**
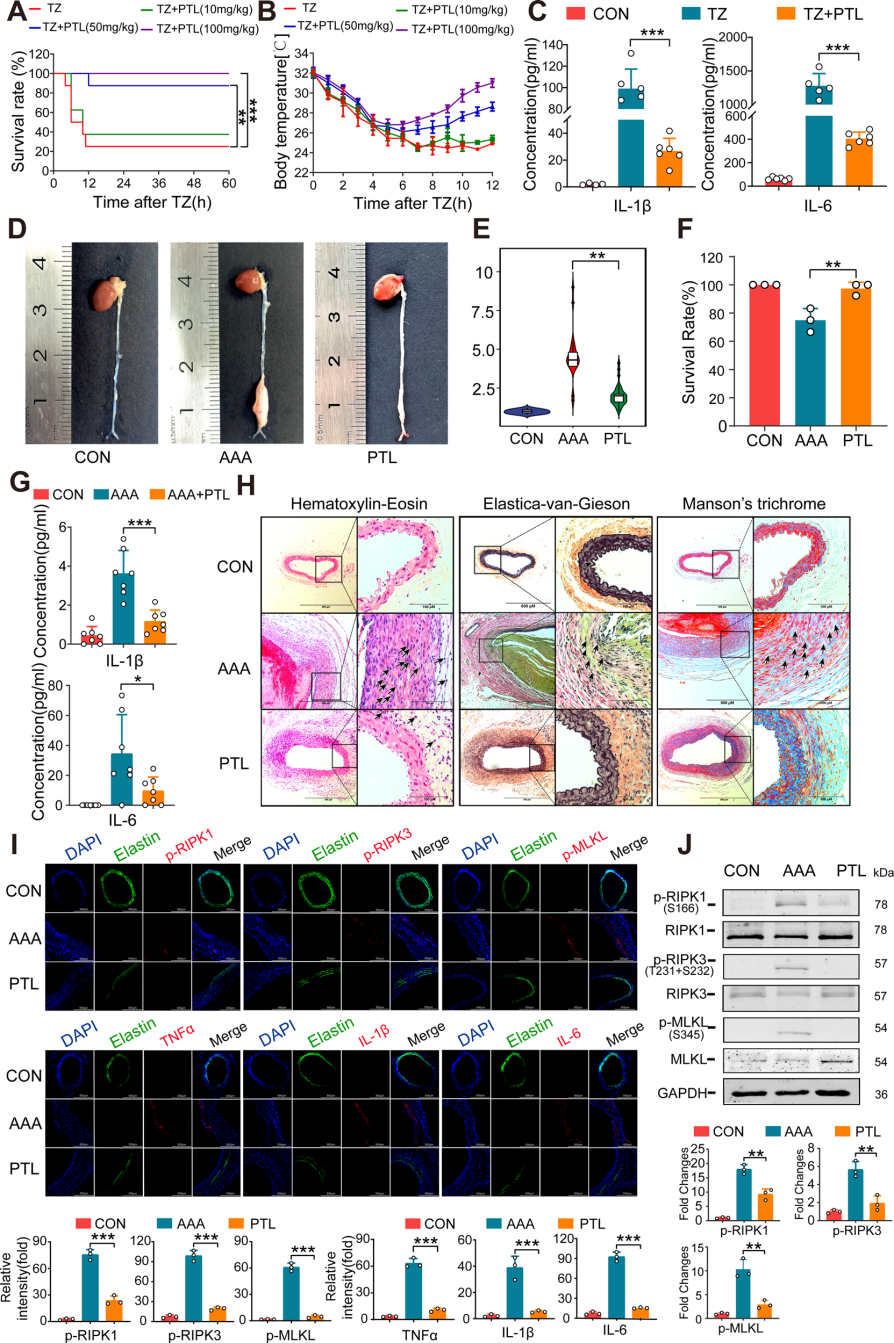
The anti-necroptotic activity of PTL in the SIRS model and the AAA model. **A** Survival rate of the SIRS model. *N* = 8. **P < 0.01, ***P < 0.001 versus with TZ. **B** Body temperature of the SIRS model. *N* = 8. **C** Serum levels of IL-1β and IL-6 of SIRS model. *N* ≥ 5 biologically independent samples. ***P < 0.001 versus with TZ. **D**, **E** Morphology and diameter statistics of the abdominal aorta in the AAA model (*N* = 30 biologically independent samples from 3 different batches). **P < 0.01 versus with AAA. **F** Survival rate of three independent batches of the AAA model (10 samples per batch). **P < 0.01 versus with AAA. **G** Serum levels of IL-1β and IL-6 of AAA model. *N* = 7 biologically independent samples. *P < 0.05, ***P < 0.001 versus with AAA. **H** Evaluation of tissue structure, cell morphology, elastin degradation, and collagen fibers analyses with HE, EVG, and Masson’s trichrome, respectively, in aortae of mice. *N* = 3 biologically independent samples. **I** Expression of p-RIPK1, p-RIPK3, p-MLKL, TNFα, IL-1β, and IL-6 in aortic tissue by immunohistochemistry. *N* (AAA and PTL) ≥ 3 biologically independent samples. **J** Immunoblotting analyses of the necroptosis signal pathway in the aortic tissue of mice.

The pivotal role of necroptosis in systemic inflammatory response syndrome (SIRS) is universally acknowledged [23, 24]. The TZ-induced SIRS model triggers severe inflammatory storms in mice, ultimately resulting in hypothermia and mortality. In this model, mice were firstly peritoneally injected (i.p.) with z-VAD-FMK, and then intravenously injected (i.v.) with TNF-α, and PTL was given by intraperitoneal injection 1 h before TNF-α. Administration of moderate (50 mg/kg) to high (100 mg/kg) doses of PTL significantly reduced the mortality rate (100 mg/kg, 100%; 50 mg/kg, 87.5%; Fig. 2A). The decrease in body temperature caused by SIRS was significantly improved with PTL treatment (Fig. 2B). PTL could also effectively lower the serum levels of inflammatory factors, IL-1β and IL-6 (Fig. 2C). In summary, PTL is capable of effectively suppressing the inflammatory storm induced by SIRS.

Subsequently, we evaluated the anti-inflammatory efficacy of PTL in a disease model of abdominal aortic aneurysm (AAA), a condition that is distinctly linked to necroptosis [25]. In the elastase-induced mouse AAA model, the abdominal aorta average diameter in mice administered with PTL treatment (100 mg/kg) was notably smaller compared to the model group, demonstrating a substantial alleviation in aorta expansion (Fig. 2D, E). Meanwhile, PTL also reduced the deaths caused by the rupture of aneurysm expansion (Fig. 2F). The concentrations of serum IL-1β and IL-6 in the PTL group were notably lower compared to those in the AAA group (Fig. 2G). Histological staining of hematoxylin and eosin (HE), Elastica-van-Gieson (EVG), and Masson’s trichrome clearly demonstrated that PTL significantly reduced the infiltration of inflammatory cells in aortic tissues, alleviated the degradation of elastin and collagen (Fig. 2H).

To determine whether necroptosis is activated in AAA tissues, we performed a transcriptomics study using bulk RNA-sequencing in aortae of different groups (Supplementary Fig. 1A). The analysis of necroptosis-associated genes revealed a clear upregulation in the expression of RIPK3 (fold change > 3 and *p* value = 2.08E-7) and MLKL (fold change > 3 and *p* value = 3.57E-11) in AAA group (Supplementary Fig. 1B). At the same time, inflammatory and immune-related genes were also analyzed, and the results showed that PTL is able to alleviate the immune response in tissues by modulating the inflammatory balance. Among them, genes related to immunoglobulin production (*Igkv1-9, Igkv17-127*) and inflammatory signaling (*Trib3, Tnfrsf9, Treml1, Il1rl1 and Ppbp*) have been downregulated, while genes that contribute to anti-inflammatory process (*Il1r2, IL-10, Il10ra, and Il10rb*) were upregulated (Supplementary Fig. 1C). The expression of inflammatory signal protein TRIB3 in aorta was confirmed to be inhibited by PTL (Supplementary Fig. 1D).

Next, we examined the expression of necroptosis pathway-related proteins and inflammatory factors in the tissue. Fluorescent immunohistochemistry clearly demonstrated the upregulation of RIPK1, RIPK3, and MLKL (Supplementary Fig. 1E) and their phosphorylation (Fig. 2I) in the AAA group, which was consistent with the transcriptomics. PTL could effectively inhibit the phosphorylation of RIPK1, RIPK3, and MLKL, suggesting that it reduced the occurrence of necroptosis in the aorta (Fig. 2I). Concurrently, the levels of inflammatory factors (TNFα, IL-1β, and IL-6) in the PTL group exhibited a marked reduction compared to the AAA group (Fig. 2I). Western blot results also indicated that PTL can effectively inhibit the activation of necroptotic signal proteins in the aorta (Fig. 2J). In summary, PTL could alleviate the progression of AAA by inhibiting the activation of necroptosis and regulating the expression of inflammation-related genes.

### MLKL deficiency is confirmed to attenuate AAA formation

Previous studies have demonstrated that MLKL may serve as a terminal executor for anti-necroptotic therapies [26]. Therefore, we employed MLKL mutants to investigate the potential role of necroptosis in the pathophysiology of aneurysms. Male MLKL^+/+^, MLKL^+/−^, and MLKL^−/−^ mice aged 8–12 weeks were selected, and aneurysm formation was induced by perivascular application of elastase. Western blot analysis of aortic tissue extracts confirmed the absence of MLKL protein in MLKL^−/−^ mice.

**Fig. 3:**
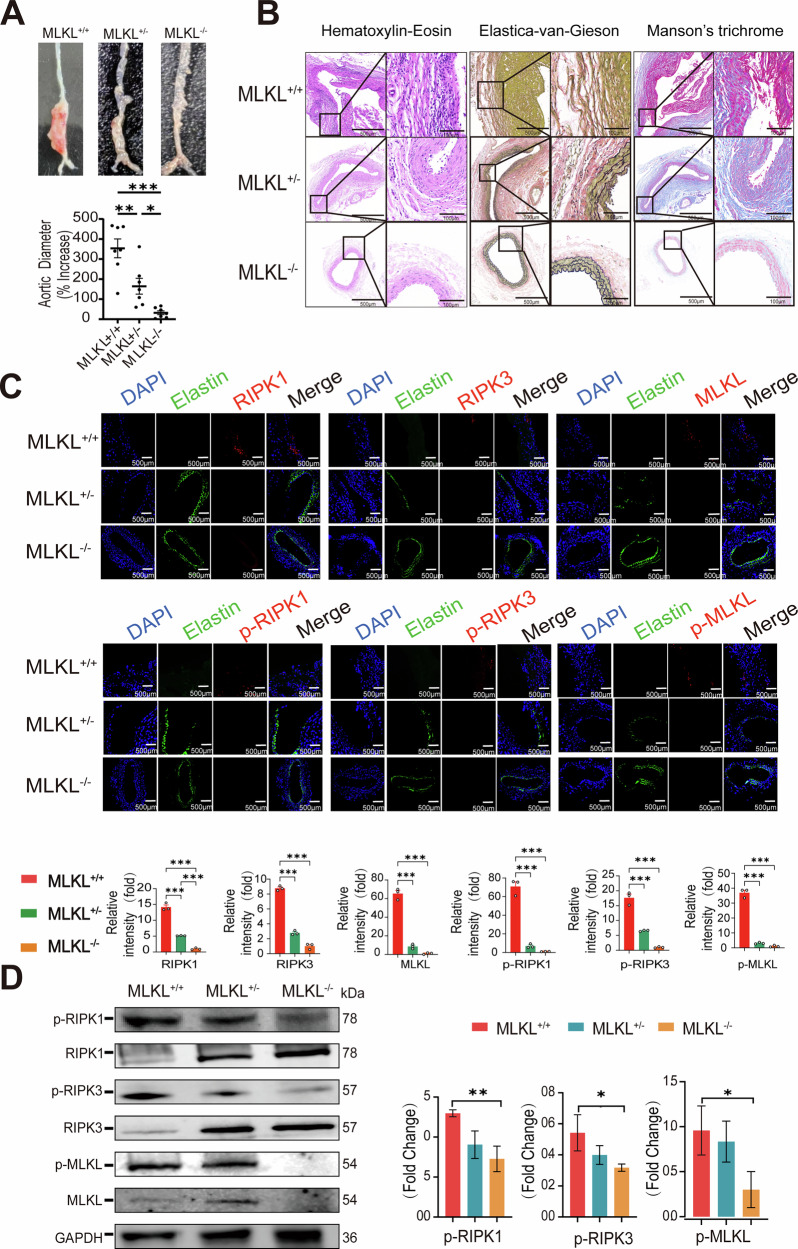
MLKL deficiency protects mice from developing AAA. **A** Mice of 3 genotypes were subjected to aneurysm induction by perivascular application of elastase. Animals were euthanized 28 days after elastase treatment. Upper, representative photographs of abdominal aortae taken during euthanization. Bottom, aortic dilatation measured as the percentage increase in maximal external aortic diameter between preperfusion and on day 28 after perfusion. An AAA is defined as a percentage increase in aortic diameter ≥100% (red dashed line). *N* = 7. **B** Evaluation of tissue structure, cell morphology, elastin degradation, and collagen fibers analyses with HE, EVG, and Masson’s trichrome, respectively, in aortae of mice. *N* = 3 biologically independent samples. **C** Expression of RIPK1, RIPK3, MLKL, p-RIPK1, p-RIPK3, and p-MLKL, in aortic tissues by Immunofluorescence. *N* (MLKL^−/−^ and MLKL^+/−^) ≥ 3 biologically independent samples. **D** Immunoblotting analyses of the necroptosis signal pathway in the aortic tissues of mice.

Mice were euthanized on day 28 following elastase treatment for aneurysm assessment. In this model, an aneurysm was defined as a ≥100% increase in the maximum external diameter compared with the preperfusion measurement. MLKL gene deletion significantly inhibited aneurysm formation: none of the 7 MLKL^−/−^ mice developed aneurysms, 5 out of 7 MLKL^+/−^ mice (71.4%) formed aneurysms, while all 7 MLKL^+/+^ mice (100%) developed aneurysms (Fig. 3A). Histological staining with HE, EVG, and Masson’s trichrome clearly revealed that MLKL knockout significantly reduced inflammatory cell infiltration in aortic tissues and alleviated the degradation of elastin and collagen (Fig. 3B). Analysis of immunofluorescence staining results showed decreased expressions of RIPK1, RIPK3, MLKL, and their phosphorylated forms in the smooth muscle cell layer of AAA tissues in the MLKL^−/−^ and MLKL^+/−^ groups (Fig. 3C). A lambda phosphatase treatment of sections was performed to ensure phospho stains are specific (Supplementary Fig. 2). Consistent with these findings, Western blot results also demonstrated that MLKL knockout effectively inhibited the activation of necrotic signaling proteins in the aorta (Fig. 3D).

### PTL inhibits necroptosis by suppressing the formation of necrosomes

**Fig. 4:**
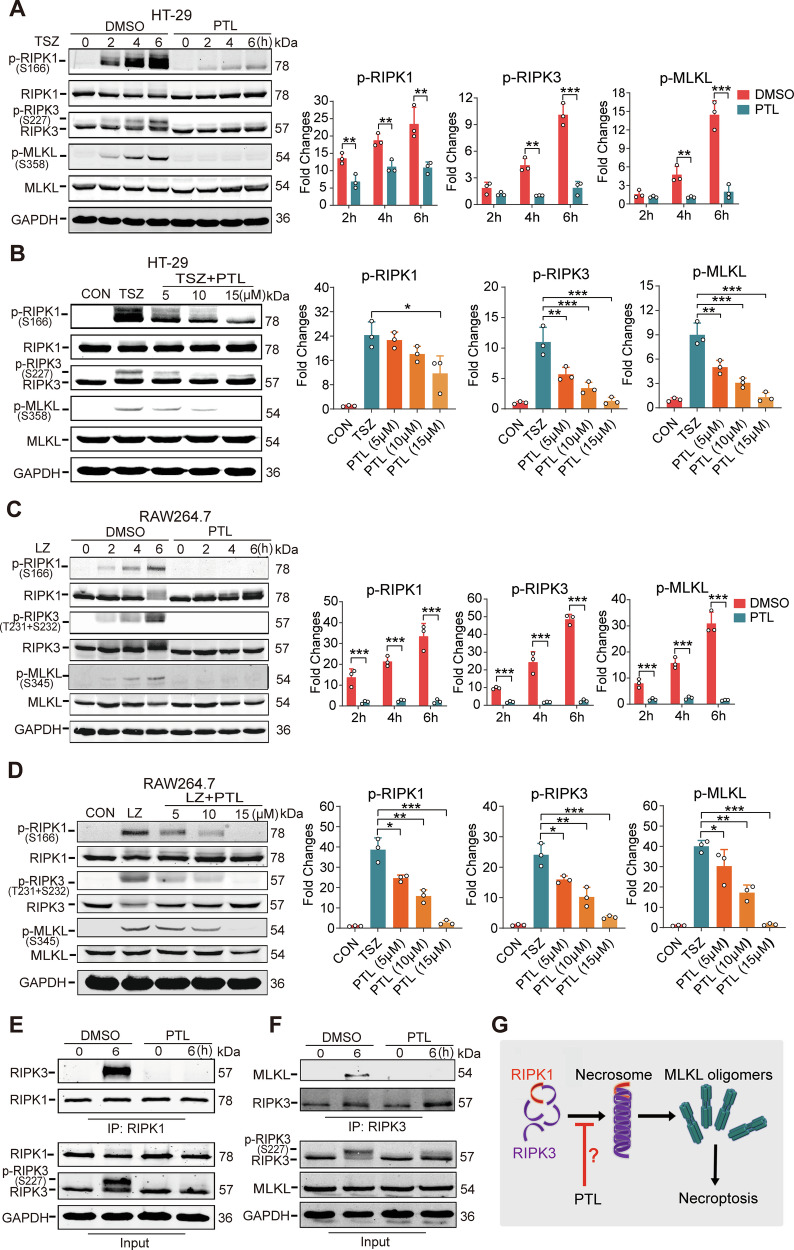
PTL inhibited necroptosis by suppressing the formation of necrosomes. **A** The effect of PTL on the necroptosis pathway at different time points in HT-29 cells. The TSZ model was induced in HT-29 cells with or without PTL (15 μM) for the indicated time. *N* = 3 biologically independent samples. **B** The effect of different concentrations of PTL on the necroptosis pathway in HT-29 cells. The TSZ model was induced in HT-29 cells with indicated concentrations of PTL for 6 h. *N* = 3 biologically independent samples. **C** The effect of PTL on the necroptosis pathway at different time points in RAW264.7 cells. LZ model was induced in RAW264.7 cells with or without PTL (15 μM) for the indicated time. *N* = 3 biologically independent samples. **D** The effect of different concentrations of PTL on the necroptosis pathway in RAW264.7 cells. The LZ model was induced in RAW264.7 cells with indicated concentrations of PTL for 6 h. *N* = 3 biologically independent samples. **E** Interaction between RIPK1 and RIPK3 was detected by co-immunoprecipitation and immunoblotting in cultured HT-29 cells with the indicated treatments. **F** Interaction between RIPK3 and MLKL was detected by co-immunoprecipitation and immunoblotting in cultured HT-29 cells with the indicated treatments. **G** A proposed schematic model of the effects of PTL on the necroptotic pathway.

Based on the results of the cell models, we believed that PTL specifically blocks necroptosis rather than apoptosis. In the TSZ model of HT-29 cells, we examined the impact of PTL on necroptotic pathway proteins across different time periods and found that it was able to inhibit the phosphorylation process of RIPK1 (S166), RIPK3 (S227), and MLKL (S358) (Fig. 4A). This inhibitory effect was dose-dependent. We established three concentrations (5, 10, and 15 μM) of PTL, and our findings revealed a progressive inhibitory effect as the concentration escalated (Fig. 4B). Next, in order to validate the effect of PTL on the necroptotic signaling pathway in murine cells, we employed an LZ model of RAW264.7 cells. The results showed that PTL was also able to significantly inhibit the phosphorylation of RIPK1, RIPK3, and MLKL in murine cells (Fig. 4C). Furthermore, consistent with human cells, this inhibitory effect also exhibited a dose-dependent manner. When the concentration reached 15 μM, PTL was able to almost completely inhibit the phosphorylation of RIPK1 and its downstream proteins (Fig. 4D).

These results indicated that PTL was able to inhibit the activation of necroptosis-related proteins; thus, we believe that PTL can interfere with the formation of the necrosome. Immunoprecipitation confirmed that PTL can significantly block the interaction between RIPK1 and RIPK3 (Fig. 4E), which subsequently prevents the phosphorylation of MLKL (Fig. 4F). In summary, our findings demonstrate that PTL exerts its function by affecting the formation of the necrosome. However, the binding targets in both human and murine are still unclear (Fig. 4G). Applying PTL as a tool compound, we may potentially find novel targets for necroptosis.

### HSPBP1 Cys201 is an upstream target contributing to the anti-necroptotic activity

**Fig. 5:**
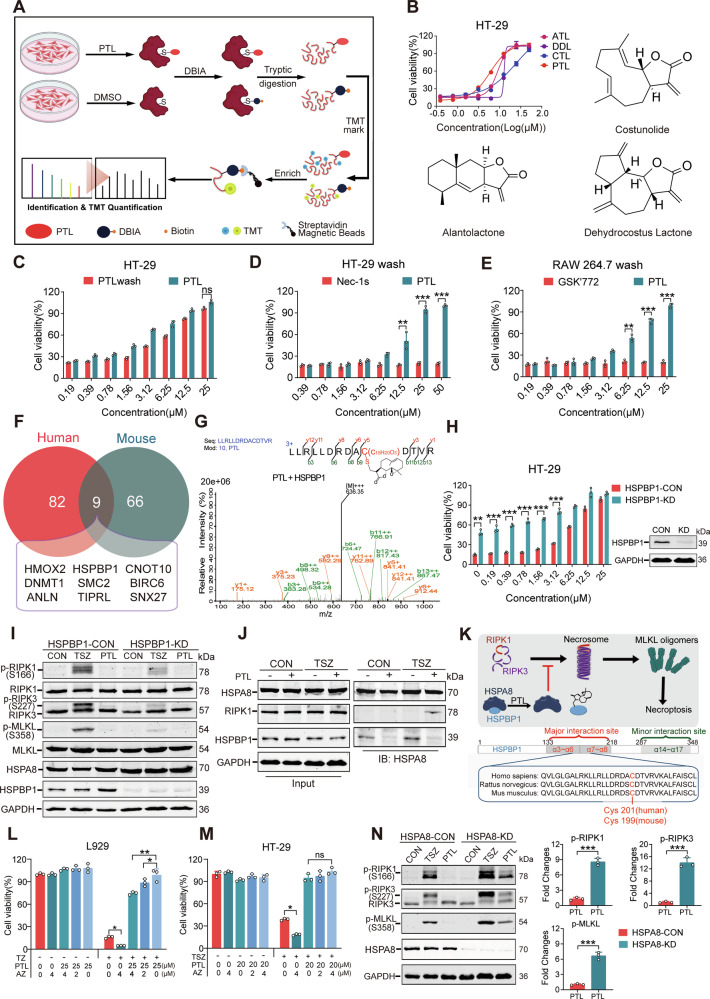
Discovery and validation of HSPBP1 as a target of PTL. **A** A brief schematic diagram of MTRP. Cellular proteins were extracted after the cells were treated with PTL or DMSO for 1 h, and then labeled with desthiobiotin iodoacetamide (DBIA) probe. Subsequently, proteins were digested into peptides, and all the peptides were isotopically labeled with TMT 6plexTM Label Reagent (Thermo). Enrich DBIA-labeled peptides through the click reaction and analyze with LC-MS/MS for identification and quantification. **B** Structures and anti-necroptotic activity evaluations of Alantolactone, Costunolide, and Dehydrocostus Lactone. **C** Evaluation of PTL activity between short-term and normal administration in HT-29 cells. *N* = 3 biologically independent samples. **D** Activity evaluation of PTL and Nec-1s through short-term administration in HT-29 cells. *N* = 3 biologically independent samples. **E** Activity evaluation of PTL and GSK’772 through short-term administration in RAW264.7 cells. *N* = 3 biologically independent samples. **F** Intersection of MTRP experimental results between human and murine cells. **G** Mass spectrometry of covalent modification of PTL with human HSPBP1 Cys201. **H** Activity evaluation of PTL in HSPBP1-KD and HSPBP1-CON cells. *N* = 3 biologically independent samples. **I** Immunoblotting analyses of the necroptosis signal pathway in HSPBP1-KD and HSPBP1-CON cells. **J** Interactions between HSPA8 and HSPBP1, and between HSPA8 and RIPK1, were detected by co-immunoprecipitation and immunoblotting in cultured HT-29 cells with indicated treatments. **K** A proposed schematic model of the effects of PTL on the necroptotic pathway through binding to HSPBP1. **L** The effect of apoptozole on necroptosis and PTL activity in the TZ model of L929 cells. *N* = 3 biologically independent samples. **M** The effect of apoptozole on necroptosis and PTL activity in the TSZ model of HT-29 cells. *N* = 3 biologically independent samples. **N** Immunoblotting analyses of the necroptosis signal pathway in HSPBA8-KD and HSPBA8-CON cells. *N* = 3 biologically independent samples.

NPs commonly contain electrophilic groups, including Michael acceptors and epoxides, leading to potent cytotoxicity and anti-inflammatory/cancer activities because of the extensive covalent modification of cysteinyl thiols on functional proteins [27]. Multiplexed thiol reactivity profiling (MTRP) is a chemoproteomic strategy to discover the target of electrophilic NPs [28]. The α, β-unsaturated γ-lactone contained in PTL is a typical electrophilic group. Thus, we aim to utilize MTRP to identify the targets of PTL (Fig. 5A), as the DiscoverX kinase assay showed that PTL does not directly inhibit the kinase activity of RIPK1 and RIPK3 at 100 μM (Supplementary Fig. 3A, B).

To elucidate the correlation between the activity of PTL and its electrophilic group, we selected three PTL analogs (constunolide, alantolactone, and dehydrocostus lactone), all bearing α, β-unsaturated γ-lactone structures, to assess their anti-necroptotic activity. As we had expected, these analogs all displayed a certain level of activity (Fig. 5B). Meanwhile, we also investigated whether PTL acts through covalent binding through “short-term administration,” in which cells were pretreated with compounds for 30 min, followed by rinsing with PBS three times, and then necroptosis was induced. In both administration modalities, PTL demonstrated stable anti-necroptosis activity (Fig. 5C). On the other hand, Nec-1s and GSK’772, two known non-covalent RIPK1 inhibitors, completely lost their activity (Fig. 5D, E). Based on this, we believed that PTL binds to the targets through covalent interactions.

Human HT-29 cells and murine RAW264.7 cells were selected for MTRP experiments, respectively. Based on 2 criteria (abundance ratio of DMSO/PTL > 2, *p* value < 0.05 in HT-29 cells, and abundance ratio of DMSO/PTL > 4, *p* value < 0.05 in RAW264.7 cells), 92 proteins, containing 96 labeled peptides in HT-29 cells; and 75 proteins, containing 75 labeled peptides, were selected (Supplementary Tables 1 and 2). By intersecting the results from the two MTRP sets, we identified 9 common proteins (Fig. 5F). After conducting a comprehensive analysis of the homology and conservation of these proteins across human and mouse, we also examined their connections to cell death research. Our investigation focused on heme oxygenase 2 (HMOX2) and heat shock protein 70-binding protein 1 (HSPBP1) as the primary candidates for further studies. HMOX2, an essential enzyme in heme catabolism, has been conclusively demonstrated to be a vital anti-inflammatory agent, exerting a crucial protective effect in mitigating both vascular and neural inflammation [29]. The sequence of HMOX2 is highly conserved in human, mouse, and rat species (Supplementary Fig. 3A). Intriguingly, HMOX2 appears to participate in the process of ferroptosis [30], prompting us to first investigate its potential relationship with necroptosis in this context. We generated an HMOX2-KD HT-29 cell line through lentivirus infection and induced a TSZ model. HMOX2-KD cells did not confer resistance to necroptosis, and PTL still retained its anti-necroptotic activity (Supplementary Fig. 3D). Crucially, PTL was still able to inhibit the phosphorylation of RIPK1 and RIPK3 (Supplementary Fig. 3E), indicating that HMOX2 is not a primary target contributing to PTL’s anti-necroptotic activity.

Next, we shifted our focus to explore the relationship between HSPBP1 and necroptosis. HSPBP1 serves as a co-chaperone for HSP70, intimately binding to its ATPase domain and thereby modulating its enzymatic activity [31]. Recently, a particular subtype of HSP70 protein, HSPA8, was unveiled to exert a potent inhibitory effect on suppressing the process of necroptosis through its binding to the RHIM domain [32]. It can bind to RHIM motif-containing proteins through its SBD (substrate-binding domain), preventing fiber formation. Meanwhile, it utilizes the ATP hydrolysis function of its NBD (nucleotide-binding domain) to decompose pre-formed fibers. This discovery prompted us to explore whether PTL had the potential to modulate the activity of HSPA8 through its interaction with human HSPBP1, thereby influencing the progression of necroptosis. Firstly, we co-incubated PTL with purified HSPBP1 and verified through mass spectrometry analysis that the binding site between PTL and HSPBP1 was consistent with the results obtained from MTRP (Fig. 5G). In the truncated HSPBP1 protein, although PTL can covalently modify multiple cysteine sites, the peptide matching number and abundance of the modification at the Cys201 site are the highest (Supplementary Table 3). Afterwards, we knocked down the expression of HSPBP1 in HT-29 cells and induced necroptosis. Upon analysis, we discovered that cells undergoing HSPBP1 knockdown demonstrated a certain level of resilience to necroptosis, accompanied by a moderate enhancement in the activity of PTL (Fig. 5H). Western blot analysis revealed a marked attenuation in p-RIPK1, p-RIPK3, and p-MLKL in the HSPBP1-KD cells (Fig. 5I and Supplementary Fig. 3F). The results of the co-immunoprecipitation revealed that the introduction of PTL disrupted the binding between HSPBP1 and HSPA8, while simultaneously, the interaction between HSPA8 and RIPK1 was observed in the TSZ group (Fig. 5J). According to current research, the Cys201 residue in human HSPBP1 (Cys199 in mouse) might be in the major interaction site that mediates its interaction with HSPA8 [33]. We hypothesized that the binding of PTL to the Cys201 of HSPBP1 diminished the interaction and released HSPA8 to subsequently inhibit the propagation of necroptotic signals (Fig. 5K). Next, we validated our findings by using an HSPA8 inhibitor, apoptozole (AZ), in both HT-29 and L929 cells. In both cellular and murine models, HSPA8 exhibits the capability to depolymerize pre-formed RHIM protein fibrils into non-activated monomers. This process is strictly dependent on its ATPase activity, ultimately contributing to the inhibition of necroptosis [13]. AZ was confirmed to specifically block ATPase activity of HSPA8. Our findings revealed that AZ exacerbated necroptosis in both cell types (Fig. 5L, M) and effectively diminished the protective effect of PTL in L929 cells (Fig. 5L). Surprisingly, AZ did not notably reduce the activity of PTL in HT-29 cells (Fig. 5M). We endeavored to prolong the exposure to necroptosis and discovered that with the extension of time, the protective efficacy of PTL on L929 cells diminished, whereas this decline was absent in HT-29 cells (Supplementary Fig. 3G, H). Finally, we performed a validation experiment using HSPA8-knockdown HT-29 cells, and our results revealed that the activity of PTL remained unaffected (Supplementary Fig. 3I). However, we observed a suppression in the ability of PTL to inhibit the phosphorylation of RIPK1, RIPK3, and MLKL (Fig. 5N). The aforementioned findings imply the existence of a downstream target in the necroptosis signaling pathway, which allows PTL to hinder the execution of necroptosis by phosphorylated MLKL in human HT-29 cells.

### Human MLKL Cys184 is another novel anti-necroptotic target in human HT-29 cells

**Fig. 6:**
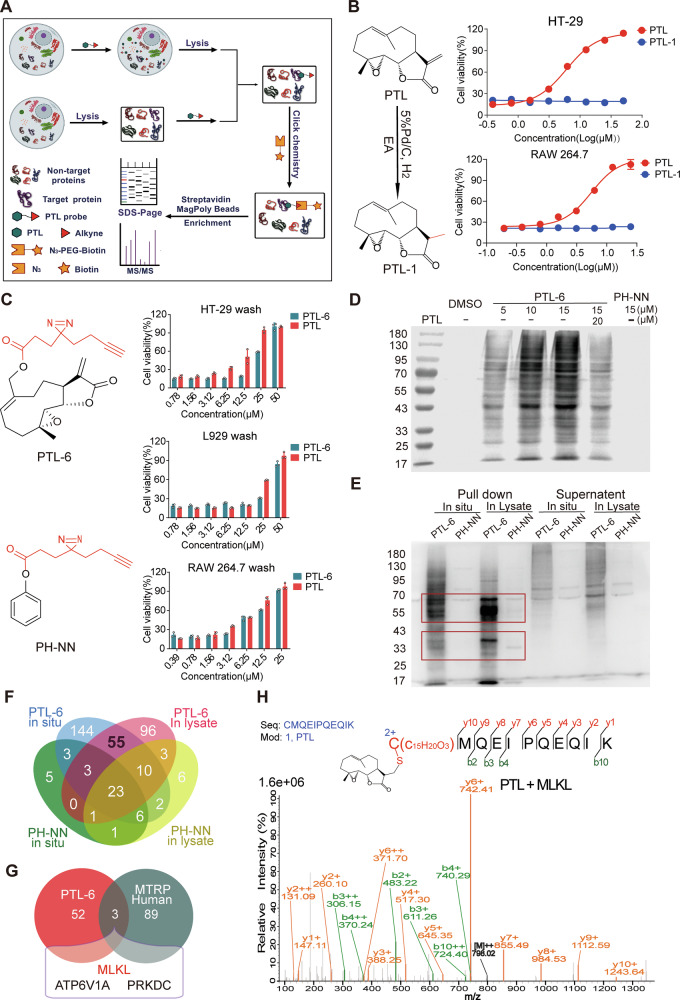
Identification of human MLKL Cys184 as a novel anti-necroptotic target for PTL. **A** A brief schematic diagram of target identification of PTL through bioorthogonal reactions. **B** Preparation and activity evaluation of PTL-1. **C** Probe structures and activity evaluation of PTL-6 with short-term administration. *N* = 3 biologically independent samples. **D** Immunoblotting analyses of probes after click reaction. **E** Immunoblotting analyses of proteins enriched by PTL-6 and PH-NN administrated in situ or in lysate. **F** Intersection of proteins enriched by PTL-6 and PH-NN. **G** Intersection of PTL-6-enriched proteins and MTRP results. **H** Mass spectrometry analysis of covalent modification of human MLKL Cys184 by PTL.

Given the above assessment, we consider adopting another strategy, namely bioorthogonal reactions, to identify potential targets of PTL that may exist in human cells. In brief, we utilized a PTL probe to bind with targets in cells and leverage the click chemistry reaction between the alkyne group of the probe and the azide group to enrich the proteins, followed by mass spectrometry analysis for identification (Fig. 6A). Before synthesizing the probe, we reduced the exocyclic double bond of PTL to obtain PTL-1. The activity of PTL-1 was completely lost (Fig. 6B), highlighting the importance of the exocyclic double bond. The results were consistent with our previous conclusion for further validating the feasibility of MTRP. Therefore, we chose to modify the methyl group, which is located far away from the critical functional groups and has a smaller steric hindrance, to obtain the probe PTL-6 (Fig. 6C). In the short-term administration experiments, PTL-6 retained its anti-necroptotic activity (Fig. 6C). Afterwards, we used PTL-6 to incubate with cells or cell lysates to label potential targets through click reaction between PTL-6 and Biotin-PEG_3_-N_3_, and then visualized the results through SDS-PAGE (Fig. 6D). The proteins labeled by PTL-6, and enriched by streptavidin beads, were used for proteolysis into peptides, followed by the analysis of the specific protein information by LC-MS/MS (Fig. 6E and Supplementary Fig. 4A). We selected the possible target proteins in the intersection of PTL-6 in situ and in lysate, excluding proteins that bind to a negative probe PH-NN, and obtained 55 candidate proteins (Fig. 6F and Supplementary Tables 4–7). Furthermore, by examining the intersection between these proteins and MTRP outcomes, we successfully narrowed down the potential targets to three promising candidate proteins, including MLKL (Fig. 6G and Supplementary Fig. 4B). MLKL, being the pivotal executor of necroptosis, has emerged as our foremost candidate for detailed investigation. The human MTRP results suggested that the possible binding site between PTL and MLKL was Cys184, a precisely non-conserved site in human and mouse MLKL (Supplementary Fig. 4C). Finally, we incubated PTL with MLKL expressed in the eukaryotic system and performed mass spectrometry analysis. It was found that PTL could interact with the sulfhydryl group of MLKL Cys184 through a covalent modification (Fig. 6H and Supplementary Table 8).

### Human MLKL Cys184 is verified by synthesizing PTL derivatives

**Fig. 7:**
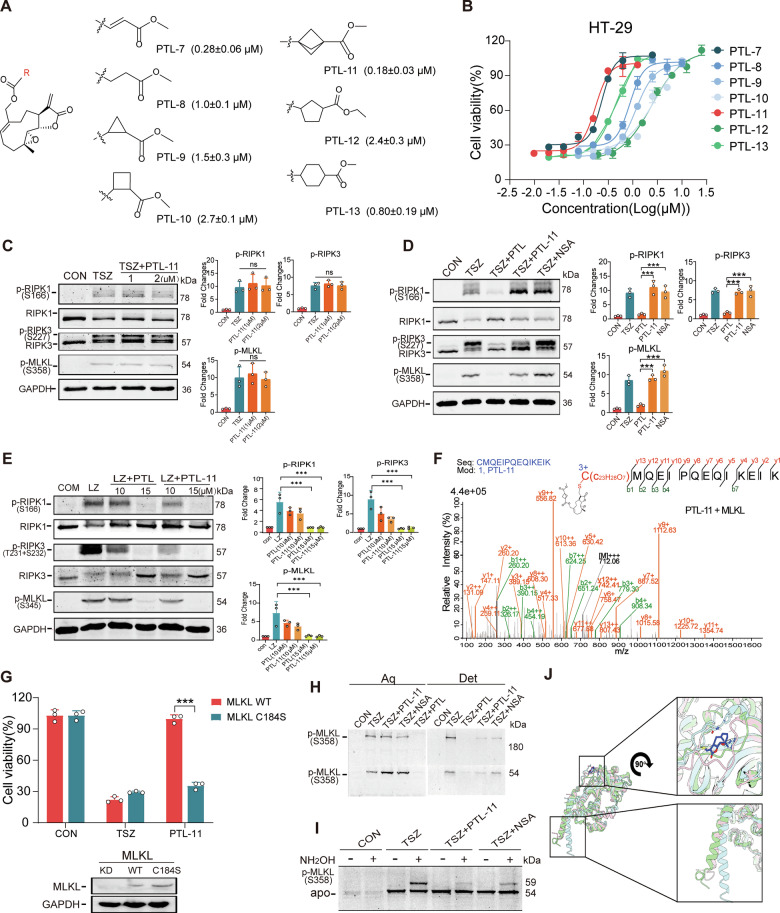
Synthesis and mechanism verification of PTL derivatives targeting MLKL Cys184. **A** Chemical structures of PTL derivatives. **B** Activity evaluation of PTL derivatives in the TSZ model of HT-29 cells. **C** The effect of PTL-11 on the necroptosis pathway in HT-29 cells at the indicated concentrations. *N* = 3 biologically independent samples. **D** The different effects of PTL, PTL-11, and NSA on the necroptosis pathway in HT-29 cells. *N* = 3 biologically independent samples. **E** The effects of PTL and PTL-11 on the necroptosis pathway in RAW264.7 cells at the indicated concentrations. *N* = 3 biologically independent samples. **F** Mass spectrometry analysis of covalent modification of human MLKL Cys184 by PTL-11. **G** Anti-necroptotic activity of PTL-11 (1 μM) in MLKL-WT and MLKL-C184S HT-29 cells. *N* = 3 biologically independent samples. **H** Distribution of p-MLKL and oligomerized p-MLKL under different treatments in HT-29 cells. **I** Acylation modification of p-MLKL under different treatments in HT-29 cells. **J** The alignment of wild-type (WT) MLKL, MLKL C184S and WT MLKL/PTL. The green cartoon represents part of the structure of WT MLKL (147–471), the pink cartoon represents part of the structure of MLKL C184S (147–471), and the blue cartoon represents part of the structure of WT MLKL/PTL, with dark blue representing PTL.

Since PTL effectively inhibited the upstream phosphorylation of RIPK1 and RIPK3, it is not feasible to directly use PTL to validate its effects following its binding to MLKL Cys184. Therefore, we endeavored to modify and synthesize a series of derivatives (PTL-7–13, Supplementary Scheme 1), subsequently screening them with the purpose of discovering a specific covalent inhibitor targeting MLKL (Fig. 7A). Upon evaluating the activity of the derivatives utilizing the HT-29 cell TSZ model, PTL-11 emerged as the most potent candidate, exhibiting an impressive EC_50_ value of 0.18 μM (Fig. 7A, B). In comparison to PTL, the activity of PTL-11 has exhibited a remarkable 33-fold enhancement. The live-cell imaging showed that cells pretreated with PTL-11 (0.5 μM) remained intact compared to those in the TSZ group (Supplementary Fig. 5A, Movies S3 and S4). Hence, we were poised to delve into the impact of PTL-11 on the necroptosis signaling pathway. While it was able to exert full protective effects at a concentration of 0.5 μM, we increased the concentration to 2 μM and found that PTL-11 had no significant effect on the phosphorylation of RIPK1, RIPK3, and MLKL (Fig. 7C). Subsequently, we employed the established covalent MLKL Cys86 inhibitor NSA [9], and discovered that the effects of both were completely different from that of PTL (Fig. 7D). Then, we examined the activity of PTL-11 in the LZ induced necroptosis model of murine RAW264.7 cells. As we predicted, the non-conservation of MLKL in murine cells led to PTL-11 failing to demonstrate a high anti-necroptotic activity as in human cells (Supplementary Fig. 5B). The Western-blotting results suggested that at a high concentration (15 μM), PTL-11, similar to PTL, exerts its activity by inhibiting the phosphorylation of RIPK1 and RIPK3 in murine cells (Fig. 7E). Similarly, we performed mass spectrometry analysis on human MLKL expressed in the eukaryotic system and PTL-11. Like PTL, PTL-11 is covalently bound to Cys184 (Fig. 7F and Supplementary Table 9). Both PTL and PTL-11 could effectively hinder the interaction between PTL-6 and MLKL, suggesting that these molecules share identical binding sites on MLKL (Supplementary Fig. 5C).

Next, to verify the role of MLKL Cys184 in necroptosis, we knocked MLKL down in HT-29 and then expressed the Ser184 mutant, followed by inducing the TSZ model. Consistent with our expectations, HT-29 cells expressing MLKL with Ser184 mutation were not sensitive to PTL-11 (Fig. 7G). Obviously, the Ser184 mutant caused PTL-11 to lose its modification site and thereby became inactive. Furthermore, we explored the impact of PTL-11 binding to MLKL on the latter’s translocation to the membrane, as MLKL’s execution of necroptosis requires its translocation to the plasma membrane [26, 34]. To characterize the MLKL activation process, we took advantage of a unique property of a detergent, Triton X-114, that undergoes phase transition at different temperatures [35]. At a low temperature (0 °C), Triton X-114 is uniformly mixed with an aqueous solution. When the temperature was raised to 30 °C, the detergent phase separated from the aqueous solution and formed a distinguished layer after centrifugation. We used Triton X-114 to extract HT-29 cells undergoing different treatments and subjected the cell extracts to the temperature-dependent phase separation procedure. We observed that PTL-11 blocked p-MLKL’s translocation to the membrane fraction (Fig. 7H*Det*), without affecting the phosphorylation and oligomerization of MLKL (Fig. 7H*Aq*). Currently, the mechanism behind MLKL’s localization to the cell membrane and its subsequent rupture remains elusive. However, research has indicated that the colonization of MLKL to the cell membrane is correlated with the acylation modification of proteins mediated by cysteine sites [36]. Thus, we used acyl-PEG exchange (APE), a method in which NH_2_OH-sensitive thioesters are cleaved and exchanged with a mass tag, to analyze the levels of p-MLKL acylated by endogenous fatty acids in cells [36]. PTL-11 could significantly inhibit the acylation level of p-MLKL (Fig. 7I).

We performed molecular dynamics simulations on the covalent binding between PTL and MLKL monomers to predict and analyze the conformation changes. The simulations revealed that covalent binding of PTL caused significant disruption to the conformational ensemble of MLKL monomers. In the WT-PTL system, PTL binding caused a noticeable displacement of the inactive KLD domain and the portion connected to the linker and its adjacent residues (roughly covering the 175–189 site region), root-mean-square deviation (RMSD) of WT MLKL/PTL to WT MLKL is 2.157 Å. In contrast, the effect of the mutation of cysteine to serine at position 184 is not significant (RMSD of C184S to WT MLKL 0.882 Å). This is because the bound PTL molecule, through steric hindrance, occupies the spatial region that is normally accessible to the proximal portion of the flexible linker (residues 147–179). This steric hindrance within the 175–189 segment directly induces substantial repositioning and alterations in conformational sampling of the adjacent flexible linker region (residues 147–179) throughout the simulation. (Fig. 7J, Movies S5–S7). These changes are specifically associated with PTL binding and were not observed in wild-type monomers or the C184S group in the absence of ligand. Such findings collectively suggest that changes induced by chemical modifications, rather than mutations at specific sites, may affect above discovered MLKL translocation.

Lastly, we performed MLKL mass spectrometry analysis on the previously selected PTL analogs and discovered that this class of compound, featuring similar α, β-unsaturated γ-lactone structures, may potentially all form covalent bonds with MLKL Cys184 (Supplementary Fig. 5D–F and Supplementary Tables 10–12). These structurally similar compounds could provide some insights for the design of novel covalent inhibitors targeting MLKL. In summary, we have demonstrated that the MLKL Cys184 site is a crucial site for PTL to inhibit the final execution phase of necroptosis, and it is highly probable that PTL exerts its effects via electrophilic groups.

## Discussion

The naturally occurring PTL has shown great promise in a variety of applications, including inflammatory diseases, headache, and cancers [21, 37, 38]. Increased evidence reveals that multiple targets associated with PTL, such as IkappaB kinase beta (IKKβ) and Janus kinases (JAKs)/ signal transducer and activator of transcription 3 (STAT3) in inflammation [39, 40], and pyruvate kinase M2 (PKM2) in cancers [41, 42]. Nevertheless, there have been no prior clues or indications suggesting the potential impact of PTL on necroptosis or associated diseases. Emerging evidence suggests that multiple regulated cell death patterns, including necroptosis, are involved in various inflammatory disorders [43]. In this study, we observed that PTL exhibited benefits to diverse cellular models of necroptosis and the in vivo necroptosis-induced SIRS and AAA models. Specifically, PTL exhibited efficacy in mitigating the infiltration of inflammatory cells within aortic tissues, inhibiting the degradation of elastin and collagen. Notably, in contrast to its impact on necroptosis, PTL did not exhibit a discernible effect in apoptosis experiments, suggesting its activity in targeting the necroptosis signaling pathway, exclusive of apoptosis-related targets. Remarkably, we demonstrate that PTL can significantly block RIPK1 and RIPK3 necrosome formation, and subsequently prevent the phosphorylation of MLKL contributing to the inhibition of cell death. Intriguingly, biochemical assays revealed that PTL does not directly inhibit the kinase activity of RIPK1 or RIPK3, hinting at the existence of novel binding targets or binding modes for this compound. Especially, we may use this compound as a tool to identify novel binding targets for the necroptosis pathway.

PTL containing an α, β-unsaturated γ-lactone, a typical electrophilic group, represents the importance of the anti-necroptosis by the reductive compound PTL-1 and several lactone analogs. Furthermore, we discerned that PTL possesses the capability to covalently modify proteins via its electrophilic moiety, a feature ideally suited for the implementation of the MTRP chemoproteomic approach. This strategy facilitates the concurrent quantification of the reactivity exhibited by cysteinyl thiols towards a diverse array of electrophilic NPs [28]. Upon analyzing the activity against both human and murine cells, along with the efficacy demonstrated in mouse models, we identified HSPBP1, rather than HMOX2, as a pivotal upstream target. Remarkably, we demonstrate that PTL covalently binds to Cys201 residue in human HSPBP1 (equivalent to Cys199 in mice), a protein that has been understudied in the context of necroptosis [44, 45]. HspBP1 is a family member of co-chaperones that modulate HSPA8 [44], which is reported recently to function as an amyloidase, inhibiting necroptosis [32]. Our findings unveil a novel mechanism whereby PTL disrupts the HSPBP1-HSPA8 interaction and impedes the HSPA8-RIPK1 interaction during necroptosis. Intriguingly, while chemical inhibition of HSPA8 or its knockdown failed to diminish PTL’s activity in human cells, a lessened protective effect of PTL was observed in murine L929 cells, suggesting the existence of an additional downstream target in human cells within the necroptotic signaling cascade. This discovery underscores the complexity and specificity of the mode of action of PTL, requiring further investigation into the intricacies of necroptosis regulation.

We further corroborated MLKL as a downstream target for PTL modification through a comprehensive approach, employing a sophisticated bioorthogonal strategy with proteomics analysis, and intersecting these findings with MTRP. MLKL is a well-characterized executioner target protein of necroptosis [35, 46, 47]. Our results demonstrated that PTL and the three PTL analogs target MLKL Cys184 in human HT-29 cells. This finding stands in stark contrast to the reported MLKL inhibitor NSA, which modifies Cys86 [9]. MLKL’s structural intricacies include an N-terminal four-helix bundle (4HB) domain vital for membrane binding and disruption [46, 48], a brace domain facilitating oligomerization and linking 4HB to the C-terminal pseudokinase domain [49], and a C-terminal pseudokinase domain [50]. Notably, Cys86 within the 4HB domain is crucial for MLKL oligomerization and necroptosis induction [49], as evidenced by NSA’s inhibitory effects on this process [9, 51]. In contrast, Cys184 resides within the brace helix, suggesting a potential site for acylation [52]. We utilized PTL as the core scaffold for medicinal chemistry optimization, culminating in the development of PTL-11—a next-generation inhibitor that exhibits 40-fold higher potency and enhanced covalent engagement with MLKL. Notably, PTL-11 exhibits unparalleled advantages over PTL in human cells: it exerts anti-necroptotic effects at significantly lower concentrations (nanomolar range). Furthermore, our investigation revealed that the more potent analog PTL-11 did not perturb the phosphorylation status of RIPK1, RIPK3, or MLKL, nor did it impact MLKL oligomerization. Notably, PTL-11 effectively inhibited the acylation level of phosphorylated MLKL by binding to the C184 site. This activity was confirmed through a single-site mutation from Cys184 to Ser184, rendering the mutant non-responsive to PTL-11. Consistently, we also found human MLKL C184S did not compromise necroptosis, similar to C86S [9, 26], which could be affected by small molecules. Molecular dynamics simulations revealed that covalent binding of PTL to MLKL C184 caused significant disruption to the conformational ensemble of MLKL monomers.

In our investigation, there exist a few notable limitations that warrant consideration. Firstly, it is crucial to acknowledge that the comprehensive crystal structure of the full-length human MLKL protein remains uncharacterized to date [26]. While the individual 4HB domain, either standalone or in conjunction with a portion of the brace helix, as well as the pseudokinase domain, have been elucidated, these descriptions exclude the critical Cys184 residue [34, 53, 54]. Furthermore, the brace domain, which serves as a linker between the 4HB and the C-terminal pseudokinase domain, comprises a limited number of amino acids and exhibits a dynamic, flexible nature, potentially hindering the achievement of a well-crystallized structure [26]. It is a challenging direction to explore the structural insight of PTL in complex with either the full-length human MLKL protein or the region encompassing the crucial Cys184 residue. Secondly, while we have prioritized proteins from the intersection based on their ranking, it is important to recognize that our current understanding is limited to these specific proteins. There may be other proteins that are regulated by PTL, which remain undiscovered and unexplored at this stage. Lastly, the relationship between HSPBP1 and MLKL signaling and necroptosis is also warranted.

In conclusion, our study establishes naturally occurring PTL as an inhibitor of necroptosis. Through rigorous chemical biology approaches, we identified HSPBP1 as an upstream target of PTL in both murine and human systems, and MLKL Cys184 as a precise downstream cellular target specifically in human cells. These findings provide valuable insights into the intricate regulatory mechanisms of necroptosis, highlighting the potential of PTL and its derivatives as therapeutic agents for modulating necroptosis-related inflammation.

## Experimental procedures

### Cell culture and treatment

HT-29, EOL-1, U937, L929, and RAW264.7 cells were cultured in high-glucose DMEM medium (BC-M-005, Bio-Channel). containing 10% fetal bovine serum (#BC-SE-FBS08, Bio-Channel) and 1% penicillin-streptomycin (#BC-CE-007, Bio-Channel).

Necroptosis of HT-29, EOL-1, and U937 cells were induced by hTNFα (20 ng/mL, #C008, novoprotein), SM164 (10 nM, #HY-15989, MCE), and z-VAD-fmk (20 mΜ, #HY-16658B, MCE) or TNFα (20 ng/mL), CHX (10 μM, #HY-12320, MCE), and z-VAD-fmk (20 μM). Apoptosis was induced by TNFα (20 ng/mL) and SM164 (10 nM) or TNFα (30 ng/mL) and CHX (10 μg/mL). Necroptosis of L929 cell was induced by mTNFα (20 ng/mL, #CF09, novoprotein), and z-VAD-fmk (20 μM). Necroptosis of RAW264.7 cells was induced by LPS (1 ng/μL, Sigma) and z-VAD-fmk (20 μM).

Cell death was determined using CellTiter-Lumi™ Luminescent Cell Viability Assay Kit (#C0065L, Beyotime), measured by SpectraMax M5 (USA). Cell viability was calculated by comparing each treatment group with the control group.

### Live-cell super-resolution panoramic microscopy

Approximately 1 × 10^5^ HT-29 cells were seeded in a confocal dish and cultured for 24-36 h before inducing the TSZ model. Live-cell super-resolution panoramic microscopy (MH-HoliView, Peking University, China) was maintained at 5% CO_2_ and 37 °C. Start timing from the moment TNF was added. Select the shooting area, set the interval time to 5 min, and the total shooting duration to 12 h. Photos were merged by MH-Merge and edited by ImageJ.

### Animals

Eight-week-old C57BL/6J wild-type mice were obtained from Legen Biotechnology Co., Ltd (Shanghai, China). MLKL^+/+^ and MLKL^+/−^ mice of C57BL/6J background were obtained from GemPharmatech (Nanjing, China). Animals were assigned randomly through a table of random numbers to cohorts by a technician who was blinded to the appearance or other characteristics of the animals. All operations in mice were approved by the Animal Care and Use Committee of Naval Medical University and followed the Principles of Laboratory Animal Care published by the National Institutes of Health (NIH publication 86-23 revised 1985) and ARRIVE guidelines. The mice were bred and housed under specific pathogen-free conditions in the central animal facility. All mice were housed at a temperature of 21–23 °C with relative humidity of 35–65% and a 12 h light/dark cycle in individually ventilated cages with access to water and standard chow diet.

### Mouse systemic inflammatory response syndrome model

The SIRS model was induced by TNFα and z-VAD-FMK. Mice were randomly divided into vehicle groups and treatment groups. In treatment groups (TZ + PTL), the different concentrations of PTL were given by intraperitoneal injection, and the model group (TZ) received an equivalent dose of the control solvent (DMSO, Tween 80, PEG400, PBSZ, accounting for 10%, 5%, 30%, 55%). After 1 h, z-VAD-FMK (200 μg) was injected intraperitoneally in the lower left abdomen, with TNF-α (55 μg/kg) then being injected intravenously 15 min later, and z-VAD-FMK (70 μg) was injected intraperitoneally in the lower right abdomen for the second time after 45 min. Body temperature was monitored with an electric thermometer after shaving the back.

### Mouse experimental abdominal aortic aneurysm model

The abdominal aortic aneurysm model was induced by perivascular application of elastase (#39445-21-1, Sigma-Aldrich, 35 mg/ml) [55]. Animals treated with the same surgical procedure and perivascular application of normal saline were used as controls. The PTL group received daily oral administration of PTL suspension (100 mg/kg, 200 μL), while the AAA group and the control group received the same volume of normal saline. 0.4% 3-Aminopropanenitrile hemifumarate (#2079-89-2, Bidepharm) was added to the drinking water of each group.

### Tissue and blood sampling

The mice were anaesthetized with pentobarbital sodium (40 mg/kg, intraperitoneally), and the blood was drawn. Aortae were dissected carefully and washed in ice-cold PBS solution for two times, and then fixed in 10% formalin or quickly frozen in liquid nitrogen and stored at −80 °C for further examination. Serum was obtained by centrifugation of the blood at 10,000 g for 5 min. Concentration of IL-1β and IL-6 were determined by ELISA Kit (#88-7013-88, #88-7064-88, Invitrogen).

### Transcriptomics study

Transcriptomics study was conducted using bulk RNA-sequencing. The aortae from the CON, AAA, and PTL groups were obtained after 4 weeks of perivascular application of elastase, and the total RNA was extracted from the aorta tissues without tunica intima, tunica adventitia, and perivascular adipose. Total RNA was extracted from the tissue using TRIzol® Reagent according to the manufacturer’s instructions. Then RNA quality was determined by the 5300 Bioanalyser (Agilent) and quantified using the ND-2000 (NanoDrop Technologies). Only high-quality RNA sample (OD260/280 = 1.8–2.2, OD260/23 ≥ 2.0, RIN ≥ 6.5, 28S:18S ≥ 1.0, >1 μg) was used to construct sequencing library. RNA purification, reverse transcription, library construction, and sequencing were performed at Shanghai Majorbio Bio-pharm Biotechnology Co., Ltd. (Shanghai, China) according to the manufacturer’s instructions (Illumina, San Diego, CA).

### Immunoblotting

Tissue samples were lysed with NP40 lysis buffer (#P0013F, Beyotime) containing protease/phosphatase inhibitor cocktails (#WB0122-1, Webio) in a homogenizer at 4 °C. Cultured cells were directly lysed on ice with the buffers. Proteins were run on 8–12% SDS-PAGE gels at 120 V for 1.5 h at room temperature. Then gels were transferred onto nitrocellulose membranes at 200 mA for 1 h in an ice-water bath. Membranes were blocked with 5% non-fat milk dissolved in TBST buffer (0.5% v/v Tween-20 in TBS buffer) for 1 h at room temperature. Membranes were washed (5 min × 3 times) with TBST, and incubated with primary antibodies (anti-RIPK1, #3493S, CST; anti-p-RIPK1 (S166, human), #65746S, CST; anti-p-RIPK1 (S166, mouse), # 53286S, CST; anti-RIPK3/p-RIPK3 (S227, human), #ab209384, Abcam; anti-RIPK3, #17563-1-AP, Proteintech; anti-p-RIPK3 (S227, human), #93654S, CST; anti-p-RIPK3 (T231/S232 mouse), #910702S, CST; anti-MLKL, #1 66675-1-Ig, Proteintech; anti-p-MLKL (human), # 91689S, CST; anti-p-MLKL (mouse), # 37333S, CST; anti-HMOX2, #14817-1-AP, Proteintech; anti-HSPBP1, #10211-1-AP, Proteintech; anti-HSPA8, #10654-1-AP, Proteintech) overnight at 4 °C. The dilution of primary antibodies ranged from 1:1000 to 1:5000. After washing, membranes were incubated with secondary antibodies conjugated with IRDye ®800CW (Li-Cor Biosciences). Images were photographed with the Odyssey system (Li-Cor Biosciences) and then analyzed with ImageJ software (NIH).

### Fluorescent immunohistochemistry

Mouse aorta was fixed in 4% formaldehyde plus 4% sucrose for 20 min and then dehydrated in 30% sucrose. After being embedded in Tissue-Tek® O.C.T. Compound (SAKURA), sections (8 μm) were blocked in 5% normal goat plasma for 3 h at room temperature. The sections were incubated with primary antibodies (anti-IL-1β, #26048-1-AP, Proteintech; anti-IL-6, #26404-1-AP, Proteintech; anti-TNFα, #26405-1-AP, Proteintech; anti-RIPK1, #3493S, CST; anti-RIPK3, #17563-1-AP, Proteintech; anti-MLKL, #1 66675-1-Ig, Proteintech; anti-p-RIPK1 (S166, mouse), # 53286S, CST; anti-p-RIPK3 (T231/S232 mouse), #910702S, CST; anti-p-MLKL (mouse), # 37333S, CST;) overnight at 4 °C and followed by CY3-conjugated secondary antibodies (#A0516/A0521, Beyotime). Nuclei were counterstained in DAPI (Invitrogen). The images were captured by a confocal microscope, FluoView™ FV1000 or digital microscope (Leica Microsystems, Berlin, Germany).

### Immunoprecipitation

HT-29 cells with different treatments were lysed in NP40 lysis buffer with protease inhibitor cocktail, and the crude lysates were cleared of insoluble debris by centrifugation at 12,000 × *g*. The lysates were immunoprecipitated with primary antibodies at 4 °C overnight. The 50 μl protein G-Agarose beads (#101242, Invitrogen) were added to the 400 μl lysates and incubated with gentle agitation at 4 °C overnight. The beads were washed 3 times with the lysis buffer and boiled with 50 μl loading buffer. The beads were removed by centrifugation (5 min at 12,000 × *g*). The supernatant fraction was collected and incubated with the following antibodies (anti-RIPK1, #3493S, CST; anti-RIPK3/p-RIPK3 (S227, human), #ab209384, Abcam; anti-MLKL, #1 66675-1-Ig, Proteintech; anti-HSPBP1, #10211-1-AP, Proteintech; anti-HSPA8, #10654-1-AP, Proteintech) for immunoblotting. The dilution of primary antibodies ranged from 1:1000 to 1:5000. After washing, membranes were incubated with secondary antibodies conjugated with IRDye ®800CW (Li-Cor Biosciences). Images were photographed with the Odyssey system (Li-Cor Biosciences) and then analyzed with ImageJ software (NIH).

### Histology staining

HE staining, Elastica-van-Gieson (EVG), and Masson’s trichrome were used to assess tissue structure and cell morphology, elastin degradation, and collagen-related fibrosis, respectively. The aorta sections (8 μm) were stained with standard procedures. Tissue blocks were fixed in 4% paraformaldehyde (PFA, volume ≥10× tissue size) at room temperature for 24–48 h. After gradient ethanol dehydration, tissues were cleared in xylene (2 × 30 min) until translucent, then infiltrated with molten paraffin (56–58 °C) three times (1 h each) in a thermostatic embedding machine. Tissues were embedded in molds with liquid paraffin, oriented for flat sections, and cooled to form blocks. Eight-micrometer sections were cut, deparaffinized, and hydrated, followed by staining with H&E (Ribiology, RB0002), EVG (Ribiology, R335), and Masson (Ribiology, RB0003) solutions. Stained slides were mounted and analyzed by microscopic examination (Nikon Eclipse E100) and image acquisition.

### Multiplexed thiol reactivity profiling (cell preparation)

Cells were cultured in 10 cm dishes, divided into PTL and DMSO groups with 3 dishes each, and grown until they reached over 90% confluence (approximately 1 × 10^7^ cells). Rinse cells with 10 mL of PBS and 10 mL of serum-free medium, respectively. Prepare 10 mL of serum-free medium containing PTL (15 μM) and an equal volume of serum-free medium containing DMSO with a concentration of ≤1%, separately. Add these media to the corresponding groups of cells and incubate in a culture incubator for 1 h. Aspirate the supernatant from each dish of cells, rinse the cells twice with 10 mL of PBS, and digest the cells with trypsin. Finally, collect the cells in 1.5 mL centrifuge tubes, rinse once with PBS, and freeze the cells in liquid nitrogen. MTRP was completed by ChomiX Biotech (Nanjing, China).

### Recombinant protein expression and purification

A bacmid encoding recombinant full-length human MLKL (residues 1–471) with an N-terminal MBP tag cleavable by TEV protease was expressed in *Sf*9 insect cells using bacmids generated in DH10Bac *E. coli* (Weidi Biotechnology, Shanghai, China) from the pFastBac vector. *Sf*9 insect cells were cultured in SF Vir exp CDM (OPM Biotechnology, Shanghai, China) media. Bacmids were transfected into *Sf*9 cells at 1 × 10^6^ density by Cellfectin^TM^ II (Thermo Fisher Scientific) mediated transfection in six-well plates following the Bac-to-Bac protocol as detailed previously [56]. The resulting P1 baculovirus was harvested after static incubation at 27 °C for 4–5 days and added at 10% v/v to 2 mL *Sf*9 cells at 1 × 10^6^ density in six-well plates, followed by another static incubation at 27 °C for 3 days to harvest the P2 virus supernatant. 200 μL P2 virus was added to *Sf*9 cells at 1 × 10^7^ density in a 10 cm cell culture dish. After 3 days static incubation at 27 °C, cells were harvested at 3000 RPM, pellets snap frozen in liquid N_2_, and either lysed immediately or stored at −80 °C. Cell lysis was performed by sonication in a buffer containing 0.5 M NaCl, 20 mM Tris-HCl (pH 8.0), 1 mM DTT. The purification process was conducted at 4 °C, starting with centrifugation of the lysate at 15000 RPM for 15 min followed by binding to HisTag Ni-NTA resin (Smart-Lesciences Biotechnology, Changzhou, China) pre-equilibrated with a low imidazole buffer [0.5 M NaCl, 20 mM Tris-HCl (pH 8.0), 5 mM imidazole (pH 8.0)], and washed extensively with a low imidazole wash buffer containing 20 mM imidazole. Elution of the protein was carried out with a high imidazole buffer [0.5 M NaCl, 20 mM Tris-HCl (pH 8.0), 250 mM imidazole (pH 8.0)]. The eluted His6-MBP-tagged MLKL protein underwent further purification steps, including cleavage of the tag with TEV protease, dialysis, Ni-pulldown to remove uncleaved material and the TEV protease, and application to a Superdex 200 Increase 10/300 GL column (Cytiva) pre-equilibrated with SEC buffer [0.5 M NaCl, 20 mM Tris-HCl (pH 8.0)]. Purified fractions were assessed by SDS-PAGE gel electrophoresis (Bio-Rad), pooled, concentrated to 2 mg/mL using a 30 kDa MWCO spin concentrator (Millipore), aliquoted, and stored at −80 °C after snap freezing in liquid N_2_.

### Mass spectrometry of covalent modification of proteins and compounds

HSPBP1 (#HY-P701386, MedChemExpress) or MLKL (1 μg/μL) proteins were incubated with PTL/PTL-11 (100 μM) overnight at 4 °C. The samples were then subjected to mass spectrometry analysis (ChomiX biotech, Nanjing, China).

### Establishment of stable cell lines

Lentivirus of HMOX2-KD (NM_002134.4, Target Seq: CAGTTCTACCTGTTTGAGAAT), HSPA8 (NM_006597.6, Target Seq: GGACCTAAATTCGTAGCAAAT), and MLKL C184S were purchased from GeneChem CO. LTD. (Shanghai, China). Lentivirus of MLKL-KD and HSPBP1 was prepared by MLKL-shRNA plasmids (NM_152649, Target Seq: CCCAACATCCTGCGTATATTT, provided by Professor Cai Zhenyu, School of Medicine, Tongji University, Shanghai, China) and HSPBP1-shRNA plasmids (NM_012267.5, Target Seq: TGTGTGAGAACATGGACAATG) using lentiviral packaging kit (#OmL-01, OMIGET), respectively.

HT-29 cells were infected with the corresponding lentiviruses for 16–24 h. After 72 h, HMOX2-KD, HSPBP1-KD, MLKL-KD cell lines were selected using puromycin (1.5 μg /mL) for an additional 72 h. MLKL-KD cells were further infected by MLKL C184S lentivirus and selected by puromycin (1.5 μg /mL) and hygromycin B (50 μg/mL).

### In lysate and in situ proteome labeling

For labeling of HT-29 cell lysates with PTL-6, 300 μg protein in 300 μL lysis buffer was added with different concentrations of probe with or without PTL, and negative probe (PH-NN) as a negative control, then the mixture was incubated for 2 h at 4 °C, click with Biotin-PEG_3_-N_3_ (#762024, Sigma-Aldrich) according standard click chemistry conditions (30 μM Biotin-PEG_3_-N_3_ from 100 mM stock solution in DMSO, 100 μM TBTA from 100 mM freshly prepared stock solution in DMSO, 1 mM TCEP from 1 M freshly prepared stock solution in deionized water, and 1 mM CuSO_4_ from 1 M freshly prepared stock solution in deionized water). After 2 h of click reaction, 4 volumes of prechilled acetone (−20 °C) were added. Then the precipitated proteins were subsequently collected by centrifugation (12,000 × *g*, 20 min, 4 °C), and washed thrice with 1 mL of prechilled methanol (−20 °C). The samples were dissolved in SDS Sample Buffer (#BL502B, Biosharp) (2×) and heated for 10 min at 95 °C. 10 μg proteins for each lane were loaded for the Western blot. Membranes were incubated with Streptavidin/HRP for 1.5 h at room temperature. Images were photographed with the Super ECL Plus kit in the Tanon 4600 system (Tanon).

For in situ proteome labeling, HT-29 cells were grown to 80–90% confluency in 6-well plates. The medium was removed and washed twice with PBS, and then treated with 2 mL medium containing PTL-6, and PH-NN was used as a negative control. After 1 h of treatment, the medium was aspirated, and cells were washed thrice with PBS to remove excessive probes. The cells were lysed with 120 μL NP40 lysis buffer containing protease and phosphatase inhibitor on ice for 30 min. A soluble protein solution was collected by centrifugation for 15 min (12,000 × *g*, 4 °C). Eventually, the protein concentrations were determined by using the BCA Protein Assay Kit (#BL521A, Biosharp) and diluted to 1 mg/mL with PBS. A freshly pre-mixed click chemistry reaction cocktail (30 μM Biotin-PEG_3_-N_3_ from 100 mM stock solution in DMSO, 100 μM TBTA from 100 mM freshly prepared stock solution in DMSO, 1 mM TCEP from 1 M freshly prepared stock solution in deionized water, and 1 mM CuSO_4_ from 1 M freshly prepared stock solution in deionized water) was added to the labeled proteome. The reaction was further incubated for 2 h prior to the addition of prechilled acetone (−20 °C). The precipitated proteins were subsequently collected by centrifugation (12,000 × *g*, 10 min, 4 °C), and washed thrice with 1 mL of prechilled methanol (−20 °C). The samples were dissolved in SDS Sample Buffer (2×) and heated for 10 min at 95 °C. 10 μg proteins for each lane were loaded for the Western blot. Membranes were incubated with Streptavidin/HRP for 1.5 h at room temperature. Images were photographed with the Super ECL Plus kit in the Tanon 4600 system.

### Pull-down and target validation

For pull-down experiments, the strategy of labeling the proteome using the probes, PTL-6 and PH-NN, was described above. After the precipitated proteins were subsequently dissolved by centrifugation, the precipitated proteins were dissolved in PBS containing 1% SDS. Upon incubation with Streptavidin MagPoly Beads (pre-balanced with PBS) for 2 h at room temperature, the beads were washed with PBS (3 × 1 mL). Finally, the enriched proteins were eluted by SDS Sample Buffer (2×) at 95 °C for 10 min and separated by SDS-PAGE (10%). Control pull-down experiments using the negative probe (NP) were carried out at the same time in lysate or in situ as a negative control. For LC-MS/MS protein identification, the SDS-PAGE gels were conducted coomassie brilliant blue staining by eStain® L1 Protein Staining System (Genescript). Each lane was cut into a gel slice and subjected to LC-MS/MS analysis, which was conducted at Shanghai Luming Biological Technology Co., Ltd (Shanghai, China). Immunoblotting experiments were carried out as above using the corresponding antibodies.

### Fractionation by phase separation

The pellets from treated cells were resuspended in 5× volume of Triton X-114 lysis buffer (20 mM HEPES, pH 7.4, 150 mM NaCl, 2% Triton X-114), and complete protease inhibitor and incubated on ice for 30 min. The cell lysate was centrifuged at 15,000 × *g* at 4 °C for 10 min, and then the supernatant was harvested as the detergent soluble fraction. After warming at 30 °C for 3 min, the detergent soluble fraction was centrifuged at 1500 × *g* for 5 min at room temperature. The aqueous layer was collected, then re-centrifuged at 1500 × *g* for 5 min to remove the contamination from the detergent-enriched layer and saved as the aqueous fraction (Aq). The detergent-enriched layer was diluted with basal buffer (20 mM HEPES, pH 7.4, 150 mM NaCl) to the same volume as the detergent soluble fraction and re-centrifuged at 1500 × *g* for 5 min. The washed detergent-enriched layer was diluted with the basal buffer to the same volume as the aqueous fraction and saved as the detergent fraction (Det). Add 4 volumes of prechilled acetone (−20 °C) to precipitate proteins and collect by centrifugation (12,000 × *g*, 10 min, 4 °C). Washed the sample thrice with 1 mL of prechilled methanol (−20 °C) and dissolved in non-reducing SDS Sample Buffer (2x, #BL511B, Biosharp) without heat.

### Acyl-PEG exchange

APE procedure was modified based on the previous report [57]. Pellets were resuspended in 200 μL lysis buffer containing 5 mM EDTA. Protein content was measured using the BCA Assay, and samples were normalized. Approximately 400 μg of proteins (185 μL) were taken for further procedures. Each sample was treated with freshly prepared 200 mM neutralized TCEP (10 μL) to achieve a final concentration of 10 mM. The samples were rotated for 30 min at RT. The cysteines were treated with freshly prepared 1 M NEM (5 μL) in ethanol to achieve a final concentration of 25 mM. The samples were rotated for 2 h at room temperature. The reaction was terminated by methanol-chloroform-water protein precipitations in the ratio of 2:1.5:1. Appropriate volumes of the prechilled solvents were added, and the samples were gently vortexed. They were centrifuged for 10 min at 12,000 rcf at 4 °C. This process was repeated thrice. The solvents were gently decanted every time without disturbing the pellet.

After the last protein precipitation, the pellets were resuspended in 1xTEA buffer with 4% SDS and 5 mM EDTA (120 μL). They were sonicated in a cool water bath and gently vortexed in between till the pellet was solubilized. The samples were then split into two equal fractions (60 μL) for treatment with and without NH_2_OH. The final concentration of NH_2_OH used was 0.75 M. A stock of 1 M in 1xTEA buffer with 0.2% Triton X-100 was freshly prepared and neutralized with NaOH. The portion of the samples undergoing NH_2_OH treatment received an appropriate volume of 1 M stock of NH_2_OH to achieve a final concentration of 0.75 M, and the samples without NH_2_OH treatment received an equal volume of 1xTEA buffer with 0.2% Triton X-100. The samples were rotated for 1 h at room temperature. After hydrolysis with NH_2_OH, the samples were subjected to a methanol-chloroform-water protein precipitation as described before. The samples were gently vortexed and again centrifuged for 10 min at 12,000 rcf at 4 °C. The solvents were decanted without disturbing the protein pellet. The pellets were resuspended in 1xTEA buffer with 4% SDS and 5 mM EDTA. They were sonicated in a cool water bath and gently vortexed till the pellet was solubilized. A 1.33 mM stock solution of mPEG-Mal (5 kDa) was freshly prepared in 1xTEA buffer with 0.2% Triton X-100, and an appropriate amount of volume of the stock was added to the sample to achieve a final concentration of 1 mM. The samples were rotated for 2 h at room temperature. After 2 h, the samples were subjected to a final methanol-chloroform-water protein precipitation. The protein disc obtained after final protein precipitation was resuspended in 50 μL of 2x loading dye containing 5 mM TCEP. 50 mM stock TCEP was prepared in 1xTEA buffer with 4% SDS. The samples were vortexed to disperse the protein disc and were boiled for 5 min at 100 °C for immunoblotting.

### Protein structure modeling, modification, and molecular dynamics simulations

To obtain a structural model of the human MLKL tetramer incorporating its flexible linker region and its C-terminal inactive KLD, residues 147–471 were modeled using AlphaFold-Multimer (accessed via https://cosmic-cryoem.org/tools/alphafold2/). A single chain from the predicted tetrameric assembly was selected as the structural model representing the wild-type (WT) form for subsequent analyses. The selected WT monomer was subjected to covalent docking calculations using Schrödinger Maestro 11.4. to model the binding of the PTL to the cysteine at position 184, resulting in the WT-PTL complex model. The Cysteine-to-Serine point mutation at position 184 (C184S) was computationally introduced into the WT monomer model using PyMOL (version 3.0.0, Schrödinger, LLC) to generate a mutant control model (C184S).

The molecular dynamics simulations were performed on three distinct systems: WT, WT-PTL, and C184S. Each system was solvated in an explicit water box (TIP3P) and neutralized with appropriate counterions using System Builder utilities available in Schrödinger 2022. Energy minimization and equilibration under NPT were performed. Unrestrained molecular dynamics production runs of 20 ns per system were conducted at 300 K temperature and 1.01325 bar pressure using the Desmond utilities. package. Coordinates were saved every Interval 100 ps for analysis. Conformational dynamics were analyzed over the last 1 ns of each trajectory. RMSD and visual inspection using PyMOL were employed to assess structural changes.

### Chemistry

All starting materials were obtained from commercial sources and were analytically pure. The ^1^H NMR and ^13^C NMR spectra were recorded on Bruker Avance 300, 500, and 600 MHz spectrometers (Bruker Company, Germany) using tetramethylsilane as an internal standard and DMSO-d_6_ as solvents. Chemical shifts (*δ* values) and coupling constants (*J* values) are given in ppm and hertz (Hz), respectively. HRMS data were acquired using a quadrupole time-of-flight micro mass spectrometer. Thin-layer chromatography (TLC) analyses were carried out on silica gel plates HGF254 (Qingdao Haiyang Chemical, China). Column chromatography separations were carried out on silica gel 200–300 mesh. The purities of the compounds were analyzed by HPLC (Agilent 1260) using a C18 column (YMC-Pack ODS-A, S-5 μm, 250 × 10.0 mml·D) with 90% methanol/10% water as the mobile phase at a flow rate of 2 mL/min, and all final compounds exhibited purities greater than 95%. Procedures for the preparation and chemical characterizations of the final compounds are described in the Supplemental Information.

### Statistical analysis

All results are shown as mean ± SEM. An unpaired two-tailed *t*-test was used for comparison of two groups; if the normality test was not passed, the non-parametric Mann–Whitney test was used. One-Way ANOVA followed by Tukey’s multiple comparison test was used for comparison of more than two groups; if the normality test was not passed, the non-parametric Kruskal-Wallis test was performed. The statistical significance level was set at 0.05, unless otherwise stated. All statistical analyses were performed with GraphPad Prism 8.

## Supplementary information

Movie S1

Movie S2

Movie S3

Movie S4

Movie S5

Movie S6

Movie S7

Supplemental Materials

Supplementary Information Tables

Supplementary Information for 60 TCM Compounds

Supplementary Information—MLKL Genetic Identification of MLKL Knockout Mice

Biological Experiment Replicate Graph

Original Western Blot Images

## Data Availability

The data underlying this article are available in its online Supplementary Materials. The mRNA sequencing data of mouse abdominal aortic aneurysm were deposited in the Figshare database with the 10.6084/m9.figshare.32052066.
