# Supplementary material for: Identification of human MLKL Cys184 and HSPBP1 Cys201 as novel cellular targets for necroptosis
Source: Cell Death Dis. 2026 Apr 22;17(1):528. doi: 10.1038/s41419-026-08764-4 (PMC13230738; doi:10.1038/s41419-026-08764-4)

**Supplemental materials for**

**Identification of Human MLKL Cys184 and HSPBP1 Cys201 as Novel Cellular Targets for Necroptosis**

Hongming Shao^1,4^, Jiabin Wu^1,4^, Qianyu Han^2,4^, Lijuan Xu^1^, Pengcheng Dai^1^, Rui Wang^1^, Jiao Li^1^, Wenbin Wu^1^, Yanan Hao^1^, Ruilin Hou^1^, Yue Chai^1^, Zhi Cheng^1^, Pei Wang^1^, Lei Xue*^2^, Ting Han*^1^, Chunlin Zhuang^*1,3^

1. The Center for Basic Research and Innovation of Medicine and Pharmacy (MOE), School of Pharmacy, Naval Medical University/Second Military Medical University, Shanghai 200433, China
2. Department of Thoracic Surgery, Second Affiliated Hospital of Naval Medical University, Shanghai 200003, China
3. School of Chemistry and Chemical Engineering, Henan Normal University, Xinxiang, Henan, 453007 China
4. These authors contribute this work equally.

***Correspondence to**

Prof. Dr. Chunlin Zhuang, Email: zhuangcl@smmu.edu.cn

Prof. Dr. Ting Han, Email: hanting@smmu.edu.cn

Prof. Dr. Lei Xue, Email: tommyxuel@smmu.edu.cn

**Supplemental Figure 1**

**
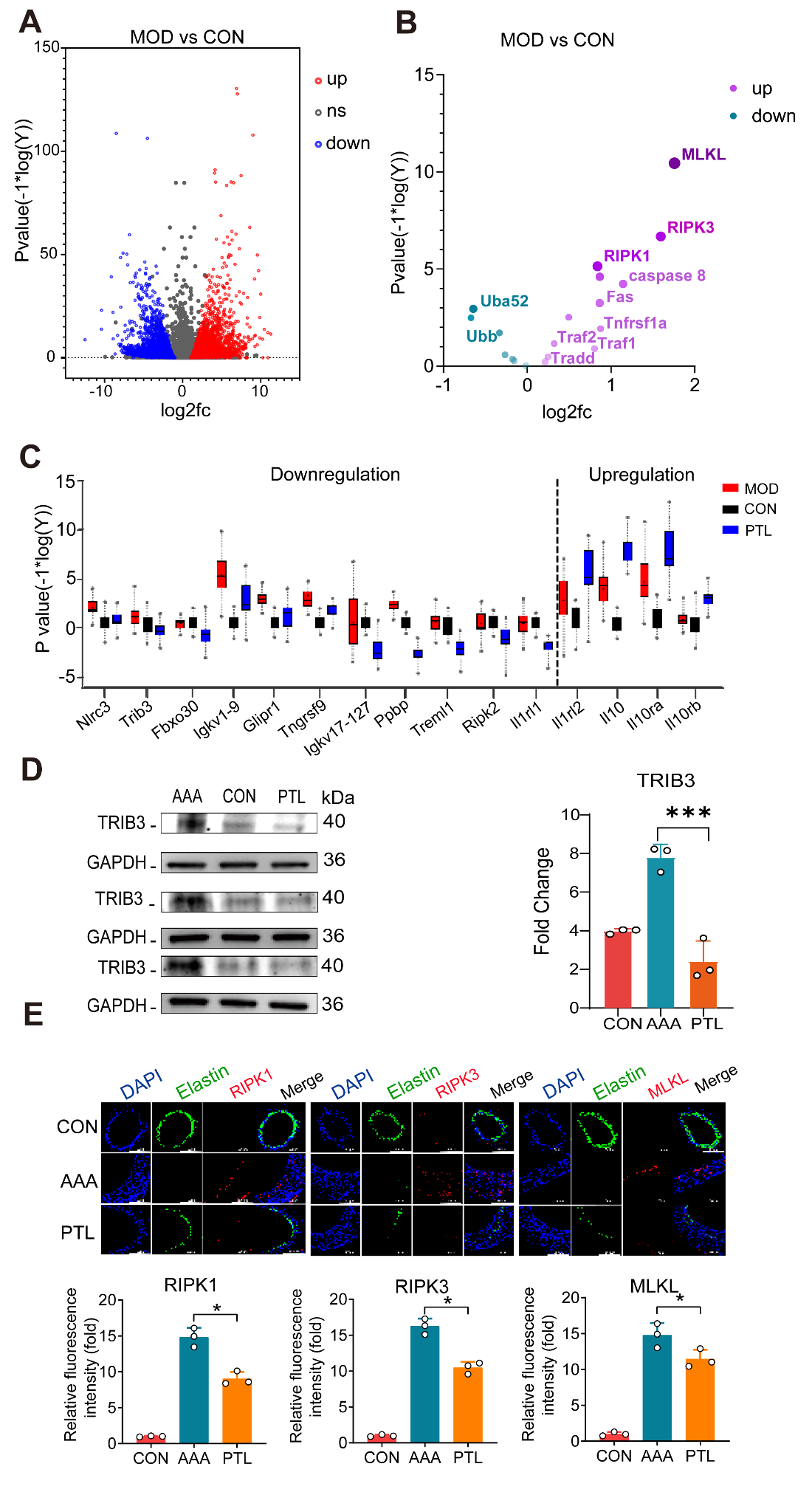
**

**Supplemental Figure 1. Transcriptomics analysis, and immunoblotting of the aortae tissues.**

(**A**) Overall volcano plot of transcriptomics between control and AAA group. N = 3 biologically independent samples.

(**B**) Regulation of genes related to necroptosis pathway in the AAA group compared with control group. N = 3 biologically independent samples.

(**C**) Regulation of genes related to immunoglobulin production and inflammatory signaling among three groups. N = 3 biologically independent samples.

(**D**) Immunoblotting analyses of TRIB3 in aortic tissue of mice.

(**E**) Expression of RIPK1, RIPK3, and MLKL aortic tissue by immunohistochemistry. N (AAA and PTL) ≥ 3 biologically independent samples. Scale bar, 500 μm.

**Supplemental Figure 2**


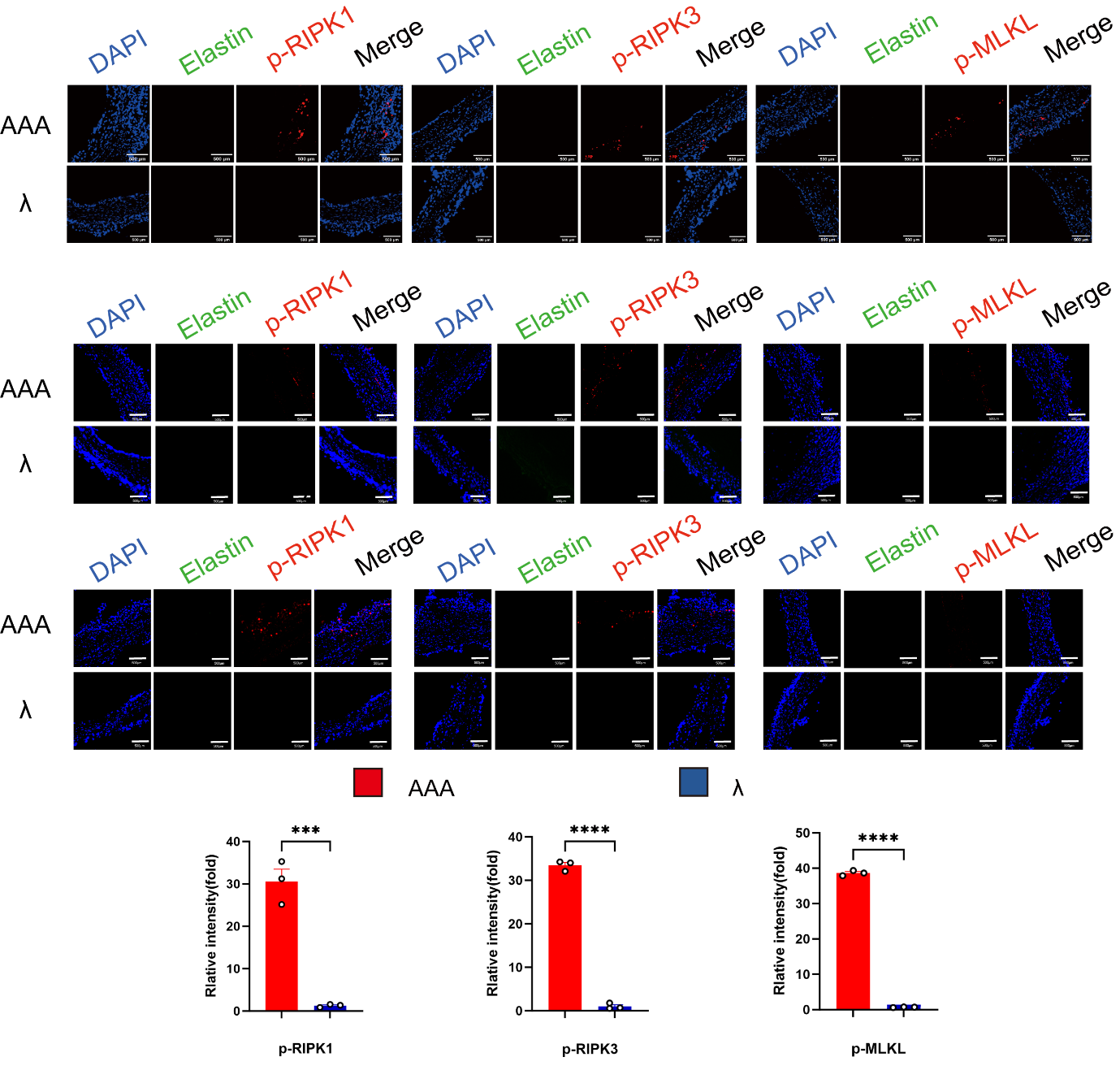


**Supplemental Figure 2. Verification of the specificity of phosphorylated staining using lambda phosphatase**

**Supplemental Figure 3**


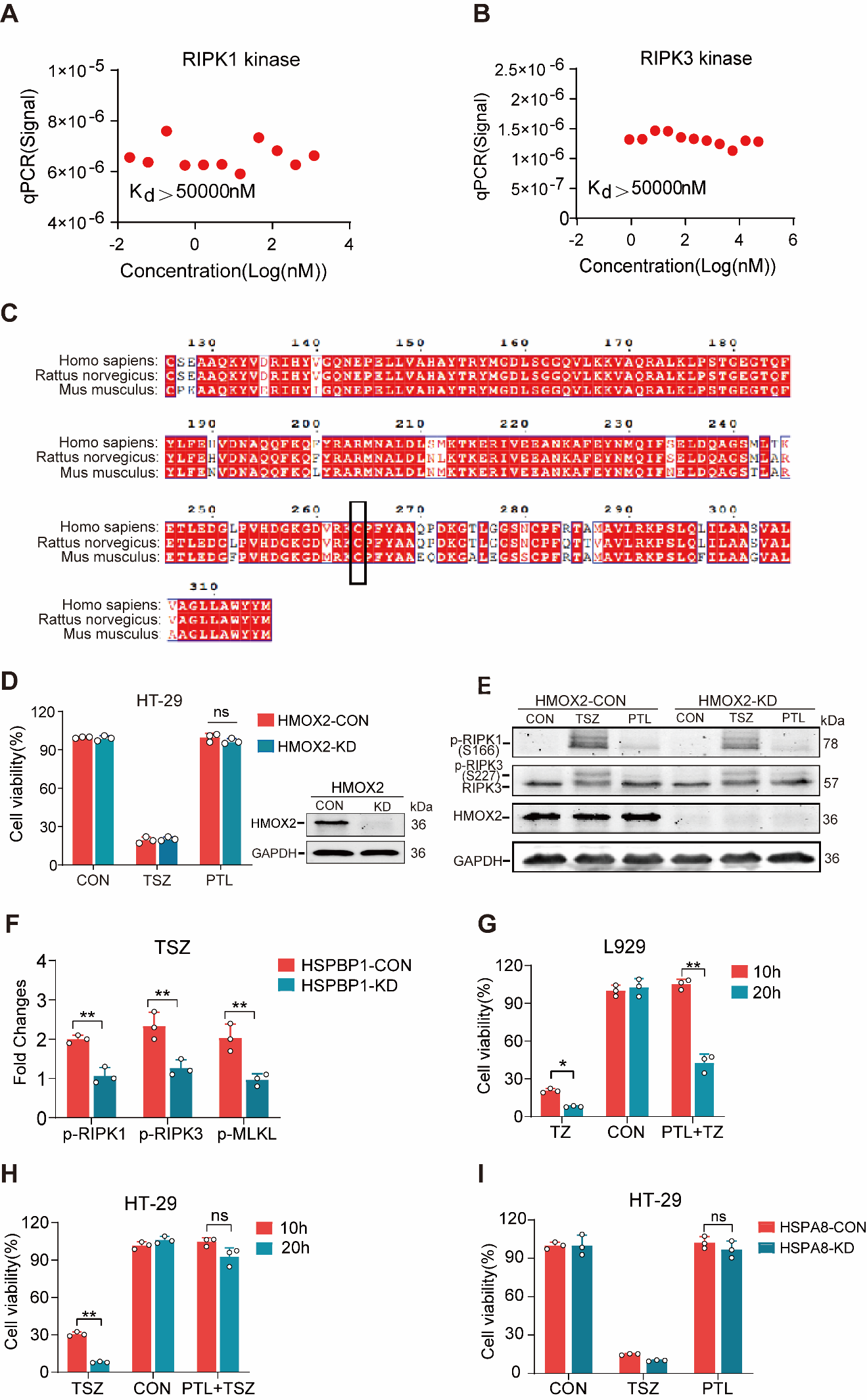


**Supplemental Figure 3. Target Verification of PTL against necroptosis.**

(**A**) RIPK1 kinase activity of PTL.

(**B**) RIPK3 kinase activity of PTL.

(**C**) Sequence conservation analysis of HMOX2 between human, mouse and rat.

(**D**) Activity evaluation of PTL in HMOX2-KD and HMOX2-CON cells.

(**E**) Effect of PTL on necroptosis signal pathway in HMOX2-KD and HMOX2-CON cells by Immunoblotting analyses.

(**F**) Immunoblotting analyses of necroptosis signal pathway in HSPBP1-KD and HSPBP1-CON cells.

(**G**) Evaluation of the activity of PTL in the TZ model of L929 cells under different induction times.

(**H**) Evaluation of the activity of PTL in the TSZ model of HT-29 cells under different induction times.

(**I**) Activity evaluation of PTL in HSPA8-KD and HSPA8-CON cells.


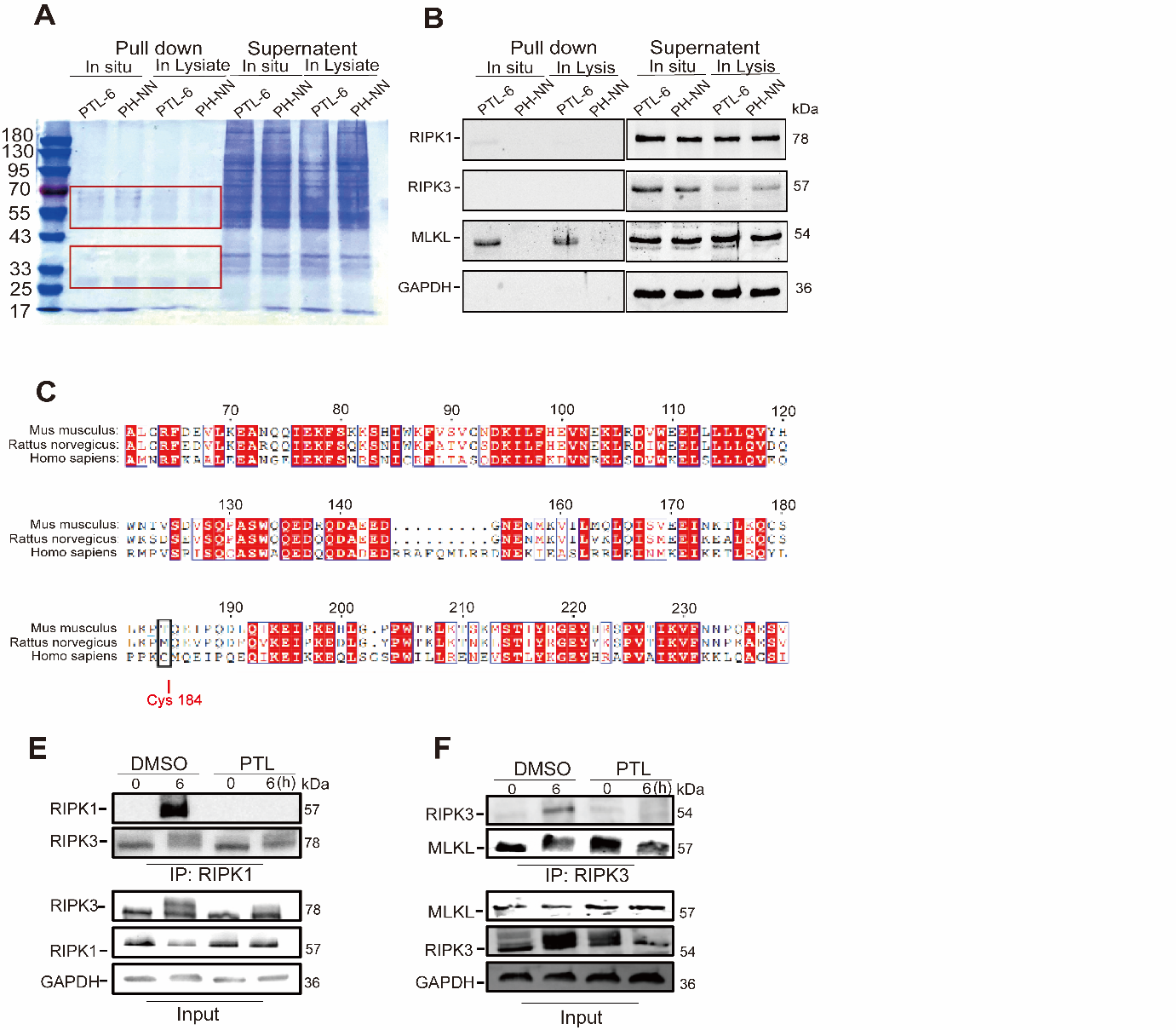


**Supplemental Figure 4. Coomassie staining, and immunoblotting of proteins enriched by PTL-6, and sequence conservation analysis of MLKL between human, mouse and rat.**

(**A**) Coomassie staining of proteins enriched by PTL-6 administrated in situ and in lysate.

(**B**) Immunoblotting of proteins enriched by PTL-6 using indicated antibodies.

(**C**) Sequence conservation analysis of MLKL between human, mouse and rat.

**Supplemental Figure 5**

**
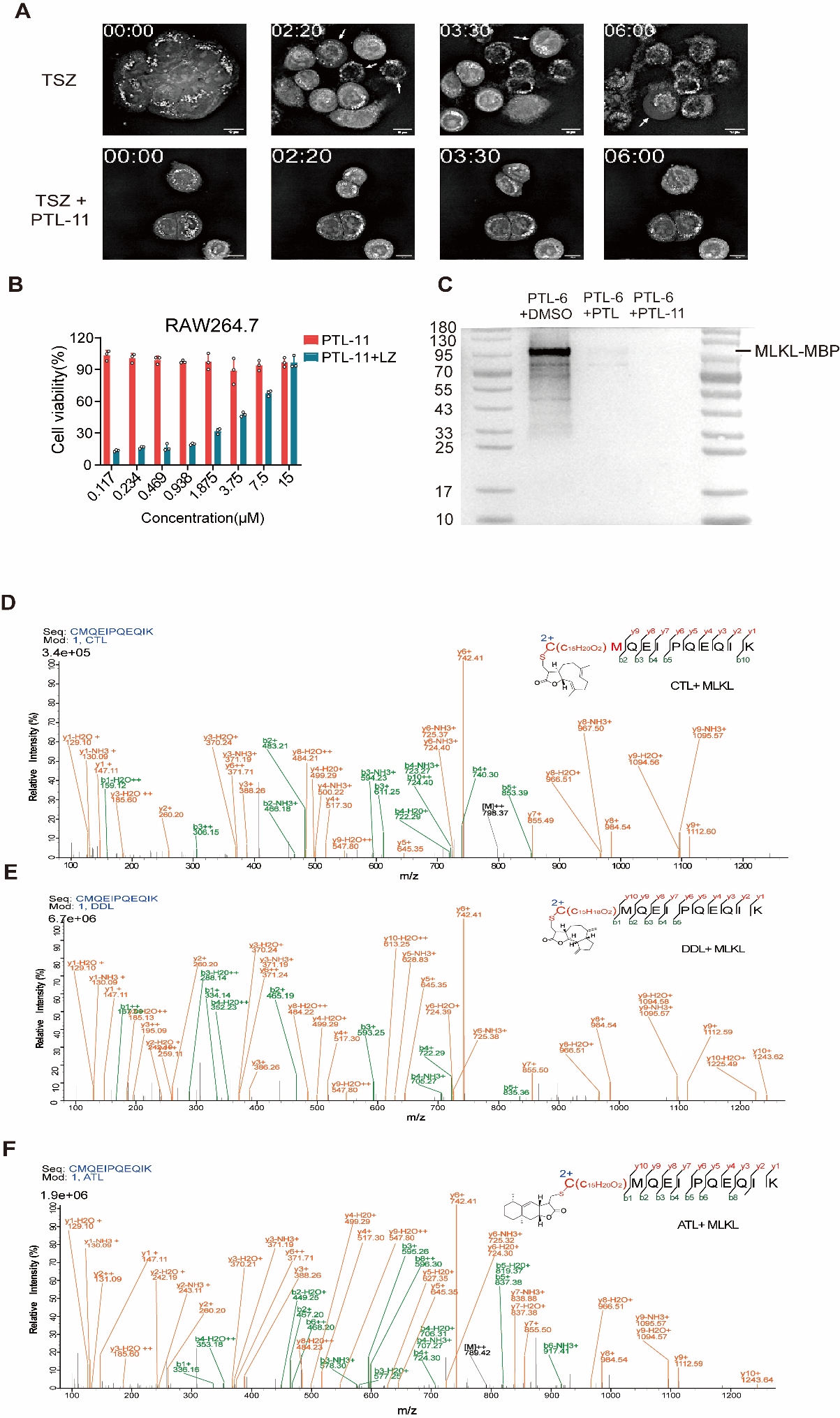
**

**Supplemental Figure 5. Verification of MLKL Cys184 as the target for PTL and its analogues, and the anti-necroptotic activity of PTL-11 in HT29 and RAW264.7 cells.**

**(A)** Live-cell imaging of HT-29 cells treated with TSZ or TSZ+PTL-11 (0.5 μM). The arrows indicated the cellular debris after rupture (02:20), the swollen cell morphology (03:30), and the instant of cell rupture (06:00). Scale bar, 10 μm.

(**B**) Activity evaluation of PTL-11 in LZ model of RAW264.7 cells.

(**C**) PTL and PTL-11 competitively inhibit the binding of PTL-6 to MLKL.

(**D-F**) Mass spectrometry of covalent binding of Costunolide (CTL), Dehydrocostus Lactone (DDL), and Alantolactone (ATL) to MLKL Cys184.

**Supplemental Movies**

**Movie S1** Live-cell super-resolution panoramic microscopy captures the entire process of TSZ-induced necroptosis in HT-29 cells, presenting the temporal dynamics of cellular contraction, rounding, structural disintegration, and eventual rupture.

**Movie S2** Live-cell super-resolution panoramic microscopy reveals that PTL-treated HT-29 cells remain morphologically stable under TSZ induction and do not undergo necroptosis.

**Movie S3** Live-cell super-resolution panoramic microscopy captures the entire process of TSZ-induced necroptosis in HT-29 cells.

**Movie S4** Live-cell super-resolution panoramic microscopy shows that HT-29 cells pretreated with PTL-11 (0.5 μM) maintain their morphology and do not undergo necroptosis upon TSZ induction.

**Movie S5** WT MLKL monomer dynamics. This movie presents the dynamic behavior of the wild-type MLKL monomer during a 20-nanosecond molecular dynamics simulation. The system exhibits its intrinsic conformational flexibility, with continuous fluctuations observed particularly in the linker region (approximately residues 147–179) and adjacent segments, reflecting the natural conformational ensemble and dynamic baseline of the protein in its unbound state.

**Movie S6**  WT-PTL complex stabilized dynamics. This movie illustrates the significant impact of PTL covalent binding at the C184 site on the conformation of the MLKL monomer. The spatial occupancy of PTL restricts the accessible space of the proximal flexible linker region (residues 147–179), directly inducing notable displacement in adjacent segments (approximately residues 175–189) and the inactive KLD domain (RMSD of 2.157 Å relative to the wild type). This reveals the molecular mechanism by which PTL interferes with the natural conformational sampling of MLKL through steric hindrance.

**Movie S7** C184S mutant enhanced dynamics. This video displays the dynamic behavior of the C184S mutant under the same simulation conditions. Compared to the wild type, the mutation does not induce significant structural rearrangement (RMSD of 0.882 Å relative to the wild type). Its conformational fluctuations resemble the baseline dynamics of the wild type, indicating that the point mutation alone is insufficient to produce conformational constraints similar to those induced by PTL binding. This further confirms the critical role of covalent modification at the C184 site in driving structural changes.

**Experimental Procedures for chemistry work**

In this part, 8 new sesquiterpene lactone derivatives and 2 probes were synthesized. The chemical structure of the compounds was identified by ^1^H-NMR, ^13^C-NMR and HRMS data. The purity of all compounds was more than 95% by HPLC analysis. The synthetic route of the new sesquiterpene lactone derivatives and their probes is shown in **Supplemental** **Scheme 1**. Initially, the α-methylene-γ-butyrolactone extracyclic double bond of parthenolide was reduced to PTL-1 by H_2_ and Pd/C. In the presence of mixed oxidant SeO_2_/TBHP, the allyl methyl of parthenolide is oxidized to obtain the intermediate PTL-2 ^1^. Then, the intermediate PTL-2 and m1 are condensed by EDCI and DMAP ester to obtain the target PTL-6~PTL-13 ^2^, and the compound PH-NN is obtained by phenol and m2 in the same way.

**Supplemental Scheme 1.** Synthesis of Parthenolide derivatives and its probe.

**Scheme 1.** Reagents and conditions: (a) SeO_2_, TBHP, DCM, 0℃, 8h, 60%;(b) EDCI, DMAP, 0℃-RT, 8h, 52%;(c) 10%Pd/C, H_2_, MeOH, RT, 4 h, 46%;(d) EDCI, DMAP, Et_3_N, EtOAc, RT, 4h, 10%.

(3S,3aS,9aR,10aS,10bS,E)-3,6,9a-trimethyl-3a,4,5,8,9,9a,10a,10b-octahydrooxireno[2',3':9,10]cyclodeca[1,2-b]furan-2(3H)-one **(PTL-1)**

**Step c:** PTL (50mg) was dissolved in ethyl acetate (3 mL), Pd/C (3 mg) was added and H_2_ was vacuumed. Then, the reaction was performed at room temperature for 1-2 h when TLC monitoring showed the completion. After the reaction, palladium carbon was removed by filtration, ethyl acetate was washed, and the filtrate was spun dry. Colorless oil product PTL-1 was purified by HPLC (20 mg, MeOH/H_2_O=75%/25%, yield 27%). ^1^H NMR (500 MHz, Chloroform-*d*) δ 5.16 (ddd, *J* = 12.6, 3.6, 1.7 Hz, 1H), 3.80 (t, *J* = 9.1 Hz, 1H), 2.69 (d, *J* = 8.9 Hz, 1H), 2.42 – 2.33 (m, 1H), 2.31 – 2.24 (m, 2H), 2.18 – 2.10 (m, 2H), 2.07 – 2.01 (m, 1H), 1.91 – 1.80 (m, 2H), 1.69 (s, 3H), 1.66 – 1.60 (m, 1H), 1.28 – 1.26 (m, 6H), 1.23 – 1.17 (m, 1H). ^13^C NMR (125 MHz, Chloroform-*d*) δ 177.30, 134.40, 125.11, 82.11, 66.32, 61.39, 51.89, 42.43, 41.11, 36.63, 29.72, 24.02, 17.12, 16.82, 13.21. MS (ESI, positive) m/z calcd for C_15_H_22_O_3_ [M+Na]^+^ 273.2 found 273.1. HPLC analysis: retention time = 7.5 min; peak area, >95% (210, 254 nm).

((1aR,7aS,10aS,10bS,E)-1a-methyl-8-methylene-9-oxo-1a,2,3,6,7,7a,8,9,10a,10b-decahydrooxireno[2',3':9,10]cyclodeca[1,2-b]furan-5-yl)methyl3-(3-(but-3-yn-1-yl)-3H-diazirin-3-yl)prop **(PTL-6)**

**
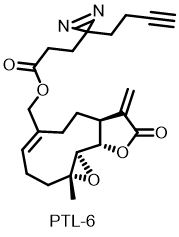
Step a:** Under the condition of ice bath, selenium dioxide (133 mg, 1.2 mmol) was dissolved in dichloromethane (20 mL), and tert-butanol peroxide (70% in H_2_O, 0.69 mL, 5.0 mmol), which was dried by anhydrous sodium sulfate in advance, was added. PTL (500 mg, 2.0 mmo1) was slowly added and dissolved in dichloromethane (10 mL). The mixture was stirred at room temperature overnight. After the reaction was completed, dichloromethane (10 mL) was added for dilution, then sodium saturated thiosulfate was added for quenching the reaction. Then, dichloromethane extraction was performed for three times, organic phase was combined with saturated salt washing for three times. Anhydrous sodium sulfate dried and filtered the mixture, and the crude product was concentrated and obtained under pressure, and purified by silica gel column chromatography. White solid compound PTL-2 (317 mg) was obtained from petroleum ether solution containing 33% ethyl acetate in 60% yield.

**Step b:** 1-ethyl -(3-dimethylaminopropyl) carbodiimide hydrochloride (115 mg, 0.6 mmol), 4-dimethylaminopyridine (1.2mg, 0.01mmol) and 3-(3-(butyl-3-acetylene-1-yl) -3h-diacridine-3-yl) propionic acid (15 mg, 0.3 mmol) were dissolved in anhydrous dichloromethane (10 ml). PTL-2 (50 mg, 0.2 mmol) was slowly added and dissolved in anhydrous dichloromethane (5 ml) at 0 ºC, then triethylamine (60.7 mg, 0.6 mmol) was added. The mixture was stirred overnight at room temperature under nitrogen protection. After the reaction was completed, sodium bicarbonate saturated solution was added, followed by dichloromethane extraction for three times. The organic phase was combined with saturated salt water washing for three times. Then, anhydrous sodium sulfate dried, filtered the mixture, and the crude product was concentrated and obtained under pressure, and purified by silica gel column chromatography (eluent: petroleum ether solution containing 40% ethyl acetate), and further purified by HPLC (mobile phase: methanol/water =60%) to obtain light yellow solid PTL-6 (30 mg) in 70% yield. ^1^H NMR (500 MHz, Chloroform-d) δ 6.23 (d, J = 4.0 Hz, 1H), 5.67 (t, J = 8.7 Hz, 1H), 5.55 (s, 1H), 4.65 (d, J = 12.4 Hz, 1H), 4.44 (d, J = 12.6 Hz, 1H), 3.83 (t, J = 9.3 Hz, 1H), 2.88 – 2.80 (m, 2H), 2.47 – 2.07 (m, 9H), 2.02-1.97(m, 3H), 1.83-1.80 (m, 2H), 1.68 – 1.60 (m, 1H), 1.52 (s, 3H), 1.24 (d, J = 15.3 Hz, 1H), 1.09 (t, J = 13.4 Hz, 1H). ^13^C NMR (125 MHz, CDCl_3_) δ 176.55, 174.07, 143.41, 139.41, 135.58, 125.07, 87.25, 85.70, 74.04, 71.67, 67.96, 64.66, 47.33, 41.28, 36.95, 33.01, 32.49, 32.28, 30.33, 28.99, 28.50, 22.67, 17.93. HRMS (ESI, positive) m/z calcd for. C_23_H_28_N_2_O_5_ [M+Na]^+^ 435.48, found 435.1. HPLC analysis: retention time= 8.70 min, peak purity,＞95%, 90% MeOH, flowrate: 2.0 mL/min.

Methyl(((1aR,7aS,10aS,10bS,E)-1a-methyl-8-methylene-9-oxo-1a,2,3,6,7,7a,8,9,10a,10b-decahydrooxireno[2',3':9,10]cyclodeca[1,2-b]furan-5-yl)methyl) fumarate **(PTL-7)**

The synthetic method refers to PTL-6, white solid compound (35 mg, yield 46.5%).^1^H NMR (600 MHz, Chloroform-*d*) δ 6.83 (s, 2H), 6.22 (d, *J* = 3.4 Hz, 1H), 5.71 (t, *J* = 8.5 Hz, 1H), 5.53 (d, *J* = 3.2 Hz, 1H), 4.75 (d, *J* = 12.5 Hz, 1H), 4.59 (d, *J* = 12.5 Hz, 1H), 3.85 – 3.81 (m, 1H), 3.79 (s, 3H), 2.86-2.81 (m, 2H), 2.45 (td, *J* = 13.8, 13.1, 5.0 Hz, 1H), 2.38 – 2.27 (m, 3H), 2.23 – 2.12 (m, 2H), 1.71 – 1.65 (m, 1H), 1.53 (s, 3H), 1.08 (t, *J* = 13.2 Hz, 1H).^13^C NMR (125 MHz, CDCl_3_) δ 169.26, 165.22, 164.61, 138.59, 134.35, 133.98, 133.15, 131.37, 120.46, 80.94, 67.45, 63.29, 59.93, 52.45, 42.66, 36.56, 25.65, 24.27, 23.85, 18.00. HRMS (ESI, positive) m/z calcd for C_20_H_24_O_7_ [M+Na]^+^ 399.4; found 399.1. HPLC analysis: retention time=8.14min, peak purity, ＞95%, 90% MeOH , flowrate:2.0mL/min.

methyl(((1aR,7aS,10aS,10bS,E)-1a-methyl-8-methylene-9-oxo-1a,2,3,6,7,7a,8,9,10a,10b-decahydrooxireno[2',3':9,10]cyclodeca[1,2-b]furan-5-yl)methyl) succinate **(PTL-8)**

The synthetic method refers to the compound PTL-6, yellow liquid compound (28mg, yield 29.8%). ^1^H NMR (500 MHz, Chloroform-*d*) δ 6.22 (d, *J* = 3.4 Hz, 1H), 5.68 (t, *J* = 8.4 Hz, 1H), 5.56 (d, *J* = 3.1 Hz, 1H), 4.67 (d, *J* = 12.4 Hz, 1H), 4.46 (d, *J* = 12.5 Hz, 1H), 3.84 (t, *J* = 9.3 Hz, 1H), 3.67 (s, 3H), 2.91-2.87(m, 1H), 2.84 (d, *J* = 9.4 Hz, 1H), 2.64 – 2.58 (m, 4H), 2.47 – 2.36 (m, 2H), 2.32 – 2.24 (m, 2H), 2.20 (q, *J* = 6.7 Hz, 1H), 2.17 – 2.12 (m, 1H), 1.69 – 1.62 (m, 1H), 1.53 (s, 3H), 1.09 (t, *J* = 13.1 Hz, 1H). ^13^C NMR (125 MHz, CDCl_3_) δ 172.77, 172.14, 169.49, 138.83, 134.84, 130.77, 120.35, 81.08, 67.16, 63.30, 59.99, 51.95, 42.69, 36.63, 29.04, 28.78, 25.71, 24.47, 23.85, 18.03. MS (ESI, positive) m/z calcd for C_20_H_26_O_7_ [M+Na]^+^ 401.4; found 401.2. HPLC analysis: retention time=7.81 min, peak area, >95%, 90% MeOH, flowrate: 2.0 mL/min.

1-methyl2-(((1aR,7aS,10aS,10bS,E)-1a-methyl-8-methylene-9-oxo-1a,2,3,6,7,7a,8,9,10a,10b-decahydrooxireno[2',3':9,10]cyclodeca[1,2-b]furan-5-yl)methyl)cyclopropane-1,2-dicarboxylate **(PTL-9)**

The synthetic method refers to the compound PTL-6, white solid (36mg, yield 48.6%). ^1^H NMR (500 MHz, Chloroform-*d*) δ 6.23 (d, *J* = 2.2 Hz, 1H), 5.69 (t, *J* = 8.4 Hz, 1H), 5.54 (d, *J* = 11.2 Hz, 1H), 4.64 (d, *J* = 18.9 Hz, 1H), 4.50 (d, *J* = 17.2 Hz, 1H), 3.84 (t, *J* = 9.3 Hz, 1H), 3.69 (s, 3H), 2.87 –2.81 (m, 2H), 2.49 – 2.11 (m, 8H), 1.72 –1.64 (m, 1H), 1.53 (s, 3H), 1.49 – 1.38 (m, 2H), 1.09 (t, *J* = 13.1 Hz, 1H). ^13^C NMR (125 MHz, CDCl_3_) δ 172.02, 171.62, 169.37, 138.72, 134.66, 131.25, 120.38, 81.03, 67.43, 63.34, 60.01, 52.33, 42.70, 36.64, 25.76, 24.45, 23.90, 22.46, 22.22, 18.05, 15.61. MS (ESI, positive) m/z calcd for C_21_H_26_O_7_ [M+Na]^+^ 413.4; found 413.1. HPLC analysis: retention time=8.25 min, peak area, >95%, 90% MeOH, flowrate: 2.0 mL/min.

1-methyl2-(((1aR,7aS,10aS,10bS,E)-1a-methyl-8-methylene-9-oxo-1a,2,3,6,7,7a,8,9,10a,10b-decahydrooxireno[2',3':9,10]cyclodeca[1,2-b]furan-5-yl)methyl)(1S,2R)-cyclobutane-1,2-dicarboxylae **(PTL-10)**

**The synthetic method refers to the compound PTL-6**, white solid (24mg, yield 36.9%) ^1^H NMR (500 MHz, Chloroform-*d*) δ 6.23 (d, *J* = 3.0 Hz, 1H), 5.68 (t, *J* = 8.5 Hz, 1H), 5.60 (d, *J* = 9.6 Hz, 1H), 4.68 (d, *J* = 12.5 Hz, 1H), 4.41 (d, *J* = 20.3 Hz, 1H), 3.84 (t, *J* = 9.3 Hz,1H), 3.66-3.63 (m, 3H), 3.44 – 3.33 (m, 2H), 2.92 – 2.82 (m, 2H), 2.45-2.40 (m, 2H), 2.38 – 2.25 (m, 4H), 2.24 – 2.12 (m, 4H), 1.68-1.61 (m, 1H), 1.53 (s, 3H), 1.10 (t, *J* = 13.1 Hz, 1H).^13^C NMR (125 MHz, CDCl_3_) δ 173.85, 173.22, 169.57, 138.92, 135.08, 130.98, 120.49, 81.15, 67.32, 63.39, 60.04, 51.88, 42.84, 40.70, 40.42, 36.68, 25.70, 24.54, 23.92, 22.32, 22.21, 18.07. MS (ESI, positive) m/z calcd for C_22_H_28_O_7_[M+Na]^+^ 427.5; found 427.2. HPLC analysis: retention time=8.07 min, peak area, >95%, 90% MeOH, flowrate: 2.0 mL/min.

1-methyl3-(((1aR,7aS,10aS,10bS,E)-1a-methyl-8-methylene-9-oxo-1a,2,3,6,7,7a,8,9,10a,10b-decahydrooxireno[2',3':9,10]cyclodeca[1,2-b]furan-5-yl)methyl)bicyclo[1.1.1]pentane-1,3-dicarboxylate **(PTL-11)**

The synthetic method refers to the compound PTL-6, white solid (42mg, yield 52.1%).^1^H NMR (600 MHz, Chloroform-*d*) δ 6.25 (d, *J* = 3.5 Hz, 1H), 5.67 (t, *J* = 8.7 Hz, 1H), 5.54 (d, *J* = 3.2 Hz, 1H), 4.66 (d, *J* = 12.5 Hz, 1H), 4.46 (d, *J* = 12.5 Hz, 1H), 3.84 (t, *J* = 9.3 Hz, 1H), 3.68 (s, 3H), 2.87-2.82 (m, 2H), 2.44 (td, *J* = 13.8, 5.0 Hz, 1H), 2.39 – 2.33 (m, 1H), 2.30 (s, 7H), 2.27 – 2.14 (m, 3H), 1.69 – 1.64 (m, 1H), 1.54 (s, 3H), 1.10 ( t, *J* = 13.1 Hz,1H).^13^C NMR (125 MHz, CDCl_3_) δ 169.52, 169.26, 169.02, 138.71, 134.56, 130.98, 120.42, 80.93, 66.72, 63.25, 59.95, 52.87, 51.92, 42.69, 37.65, 37.63, 36.59, 25.66, 24.43, 23.86, 18.00. HRMS (ESI,positive) m/z calcd for C_23_H_28_O_7_ [M+Na]^+^ 439.5; found 439.2. HPLC analysis: retention time=7.98 min, peak purity, ＞95%, 90% MeOH, flowrate: 2.0mL/min.

1-ethyl3-(((1aR,7aS,10aS,10bS,E)-1a-methyl-8-methylene-9-oxo-1a,2,3,6,7,7a,8,9,10a,10b-decahydrooxireno[2',3':9,10]cyclodeca[1,2-b]furan-5-yl)methyl)cyclopentane-1,3-dicarboxylate **(PTL-12)**

The synthetic method refers to the compound PTL-6, white solid (39 mg, yield 49.2%).^1^H NMR (500 MHz, Chloroform-d) δ 6.19 (d, J = 3.3 Hz, 1H), 5.63 (t, J = 8.7 Hz, 1H), 5.53 (d, J = 5.0 Hz, 1H), 4.64 (d, J = 12.4 Hz, 1H), 4.41 (d, J = 12.6 Hz, 1H), 4.12 – 4.05 (m, 2H), 3.82 (t, J = 9.3 Hz, 1H), 2.87 – 2.71 (m, 4H), 2.44 – 2.22 (m, 4H), 2.21 – 2.02 (m, 4H), 1.96-1.84 (m, 4H), 1.64 (t, J = 12.2 Hz, 1H), 1.50 (s, 3H), 1.23-1.18 (m, 3H), 1.05 (t, J = 13.2 Hz, 1H). ^13^C NMR (125 MHz, CDCl_3_) δ 175.21, 174.96, 169.41, 138.75, 134.98, 130.45, 120.33, 81.01, 66.71, 63.25, 60.52, 59.99, 43.81, 43.77, 42.65, 36.60, 33.26, 29.20, 29.02, 25.60, 24.32, 23.79, 17.97, 14.21. HRMS (ESI, positive) m/z calcd for C_24_H_32_O_7_ [M+Na]^+^ 455.5; found 455.2. HPLC analysis: retention time=8.70 min, peak purity, ＞95%, 90% MeOH, flowrate: 2.0mL/min.

1-methyl4-(((1aR,7aS,10aS,10bS,E)-1a-methyl-8-methylene-9-oxo-1a,2,3,6,7,7a,8,9,10a,10b-decahydrooxireno[2',3':9,10]cyclodeca[1,2-b]furan-5-yl)methyl)cyclohexane-1,4-dicarboxylate **(PTL-13)**

The synthetic method refers to the compound PTL-6, yellow solid (21mg, yield 31.5%). ^1^H NMR (500 MHz, Chloroform-*d*) δ 6.23 (d, *J* = 3.8 Hz, 1H), 5.66 (t, *J* = 8.5 Hz, 1H), 5.54 (d, *J* = 3.8 Hz, 1H), 4.65 (d, *J* = 12.3 Hz, 1H), 4.42 (d, *J* = 12.5 Hz, 1H), 3.84 (t, *J* = 9.3 Hz, 1H), 3.66 (s, 3H), 2.89–2.82 (m, 2H), 2.51 – 2.39 (m, 2H), 2.38 – 2.24 (m, 4H), 2.22–2.12 (m, 2H), 2.06 – 2.00 (m, 2H), 1.93 – 1.82 (m, 2H), 1.71–1.63 (m, 3H), 1.53 (s, 3H), 1.45–1.41 (m, 2H), 1.08 (t, *J* = 13.0 Hz, 1H). ^13^C NMR (125 MHz, CDCl_3_) δ 177.22, 176.11, 170.74, 140.12, 136.35, 131.80, 121.74, 82.41, 67.84, 64.70, 61.39, 53.09, 44.10, 43.73, 42.24, 38.05, 29.51, 29.35, 27.42, 27.39, 27.06, 25.72, 25.23, 19.41. HRMS (ESI, positive) m/z calcd for C_24_H_32_O_7_ [M+Na]^+^ 455.5；found 455.2. HPLC analysis: retention time=8.69min,peak purity,＞95%，90% MeOH, flowrate: 2.0mL/min.

phenyl 3-(3-(but-3-yn-1-yl)-3H-diazirin-3-yl)propanoate **(PH-NN)**

**Step d:** 1-ethyl -(3-dimethylaminopropyl) carbodiimide hydrochloride (115 mg, 0.6 mmol), 4-dimethylaminopyridine (1.2 mg, 0.01 mmol) and 3-(3-(butyl-3-acetylene-1-yl) -3h-diacridine-3-yl] propionic acid (15 mg, 0.092 mmol) was dissolved in anhydrous dichloromethane (10 ml). Then, phenol (9 mg, 0.092 mmol) dissolved in anhydrous dichloromethane (5 ml) was slowly added at 0 ºC, triethylamine (60.7 mg, 0.6 mmol) was added. The mixture was stirred overnight at room temperature under nitrogen protection. After the reaction was completed, sodium bicarbonate saturated solution was added. Then, the organic phase was extracted using dichloromethane for three times. The organic phase was combined with saturated salt washing for three times, then dried by anhydrous sodium sulfate and filtered. The crude product was concentrated under pressure, and purified by silica gel column chromatography column. The transparent liquid compound PH-NN (10 mg) was obtained from petroleum ether solution containing 3.5% ethyl acetate in a yield of 43%. ^1^H NMR (500 MHz, Chloroform-*d*) δ 7.42 – 7.33 (m, 2H), 7.24 (t, *J* = 7.4 Hz, 1H), 7.15 – 7.06 (m, 2H), 2.39 (t, *J* = 7.6 Hz, 2H), 2.09-2.02 (m, 2H), 2.01 (t, *J* = 2.6 Hz, 1H), 1.94 (t, *J* = 7.6 Hz, 2H), 1.71 (t, *J* = 7.4 Hz, 2H). ^13^C NMR (125 MHz, CDCl_3_) δ 175.49, 155.25, 134.19, 130.68, 126.17, 87.28, 74.05, 36.96, 33.32, 32.74, 32.26, 17.98. HRMS (ESI, positive) m/z calcd for C_14_H_14_N_2_O_2_ [M+Na]^+^ 265.27; found 265.0. HPLC analysis: retention time=9.20min,peak purity,＞95%, 90% MeOH, flowrate: 2.0mL/min.

**References**

(1) Wang, P.; Yang, H.; Lin, W.; Zhou, J.; Liu, Y.; Ma, L.; Li, M.; Hu, Y.; Yu, C.; Zhang, Y.et al. Discovery of Novel Sesquiterpene Lactone Derivatives as Potent PKM2 Activators for the Treatment of Ulcerative Colitis. *J Med Chem* **2023,** *66* (8), 5500.

(2) Liu, X.; Wang, X. Recent advances on the structural modification of parthenolide and its derivatives as anticancer agents. *Chin J Nat Med* **2022,** *20* (11), 814.

**Chemical characterizations of the target compounds**

***^1^H NMR spectra of compounds***

**
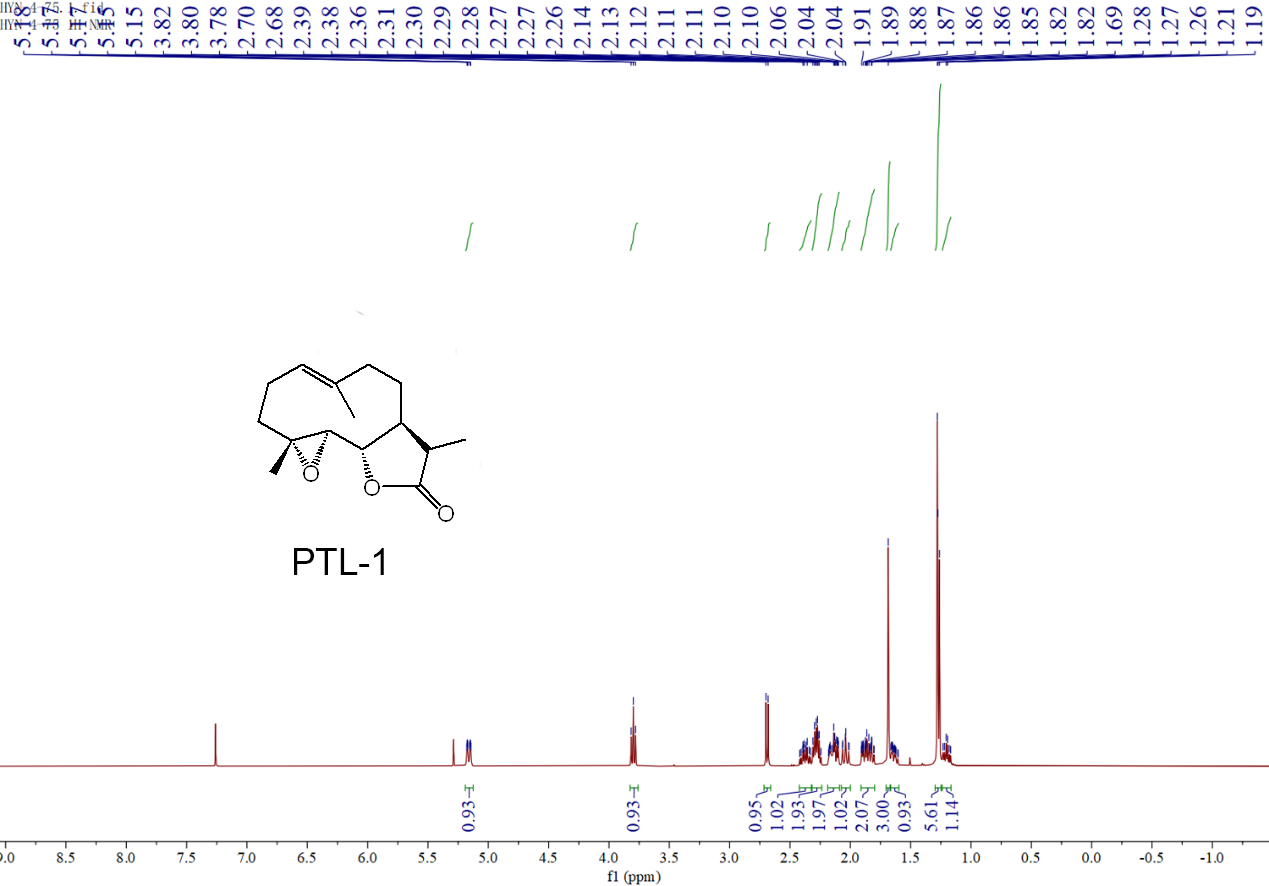
**


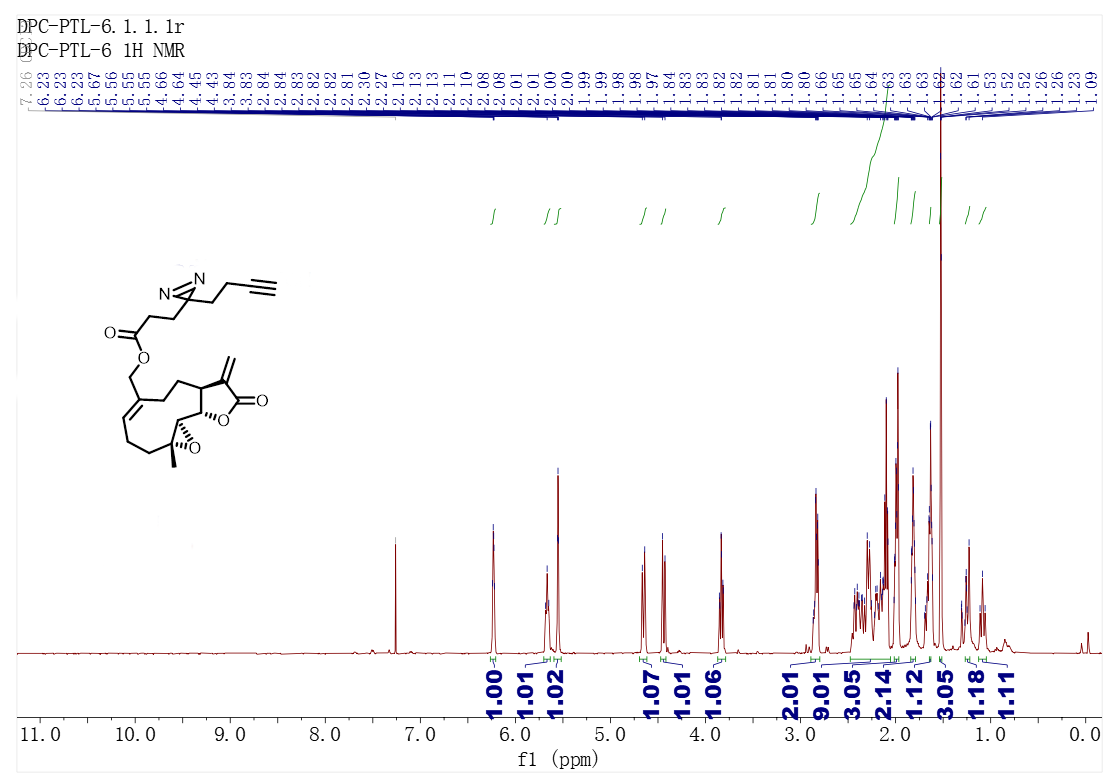


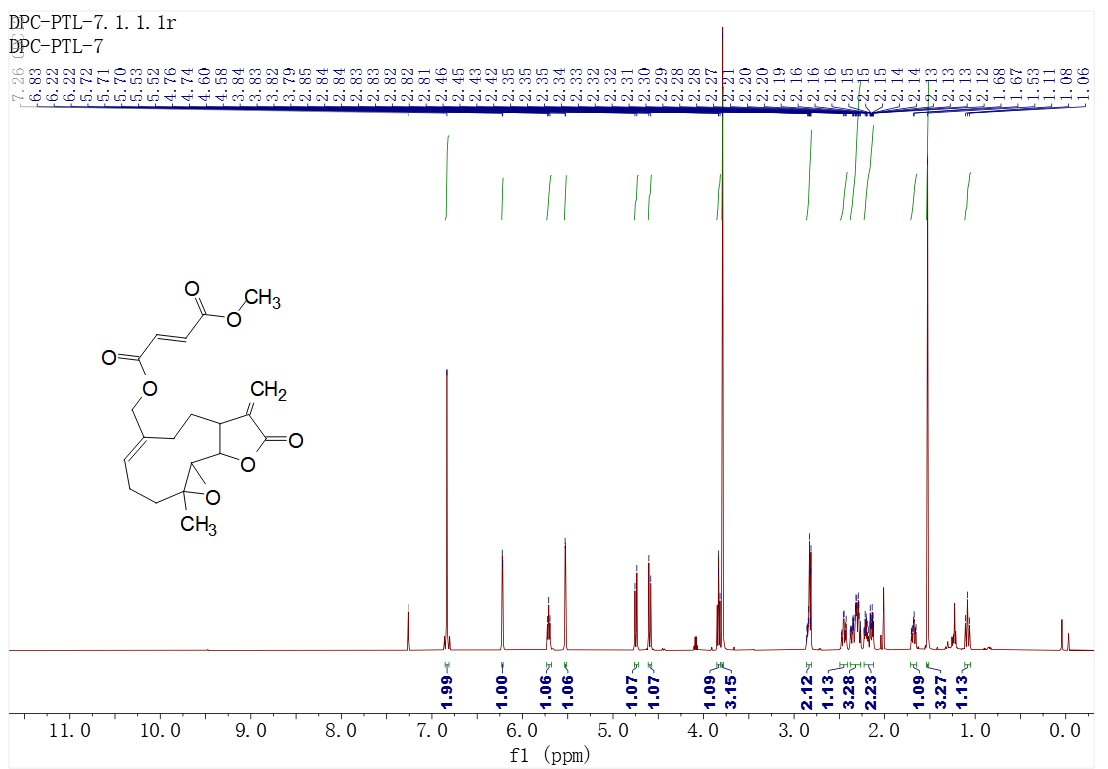


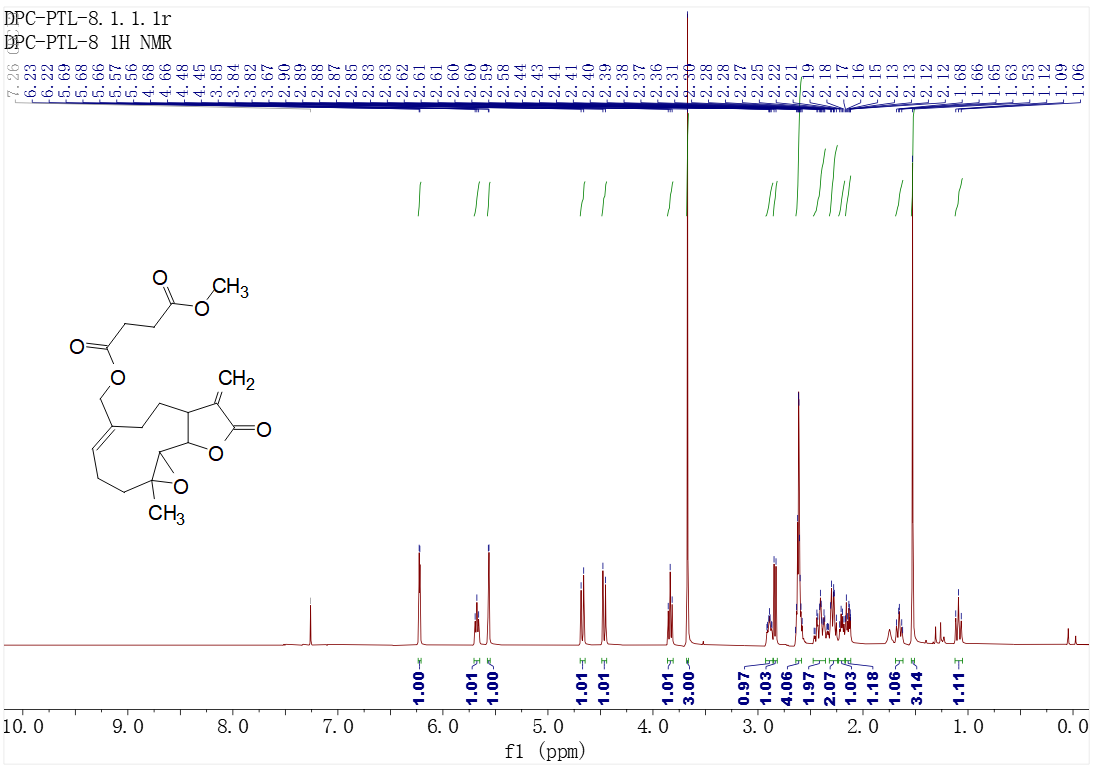


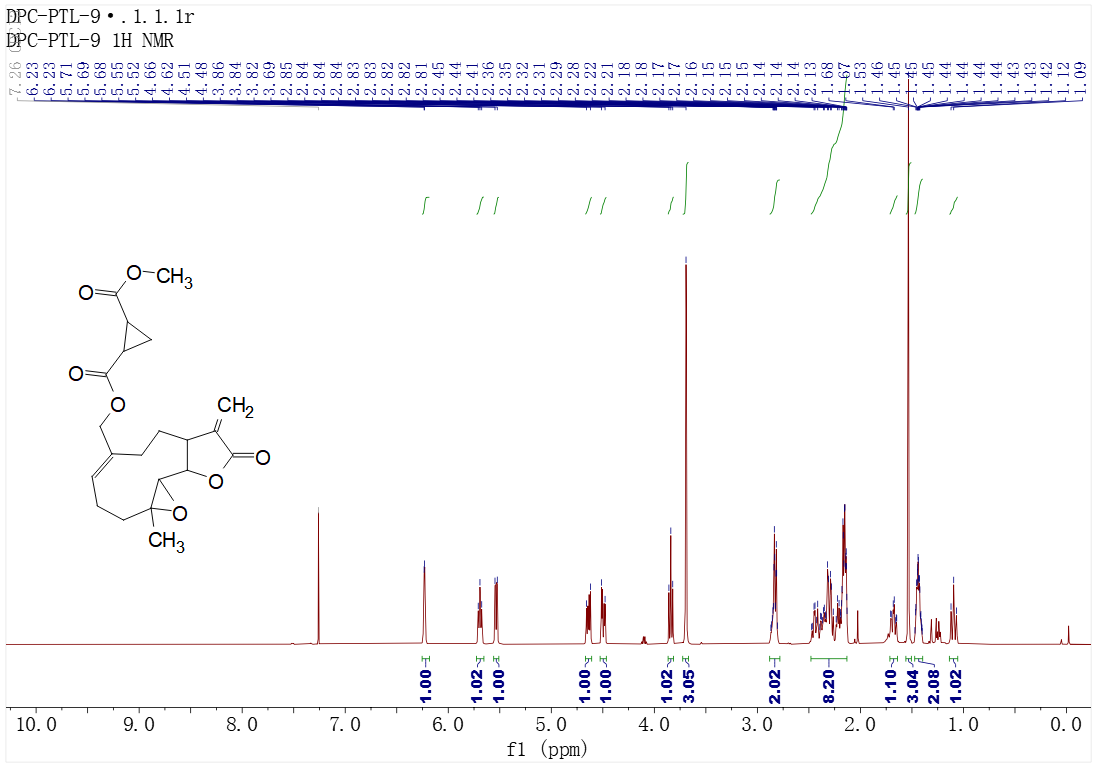


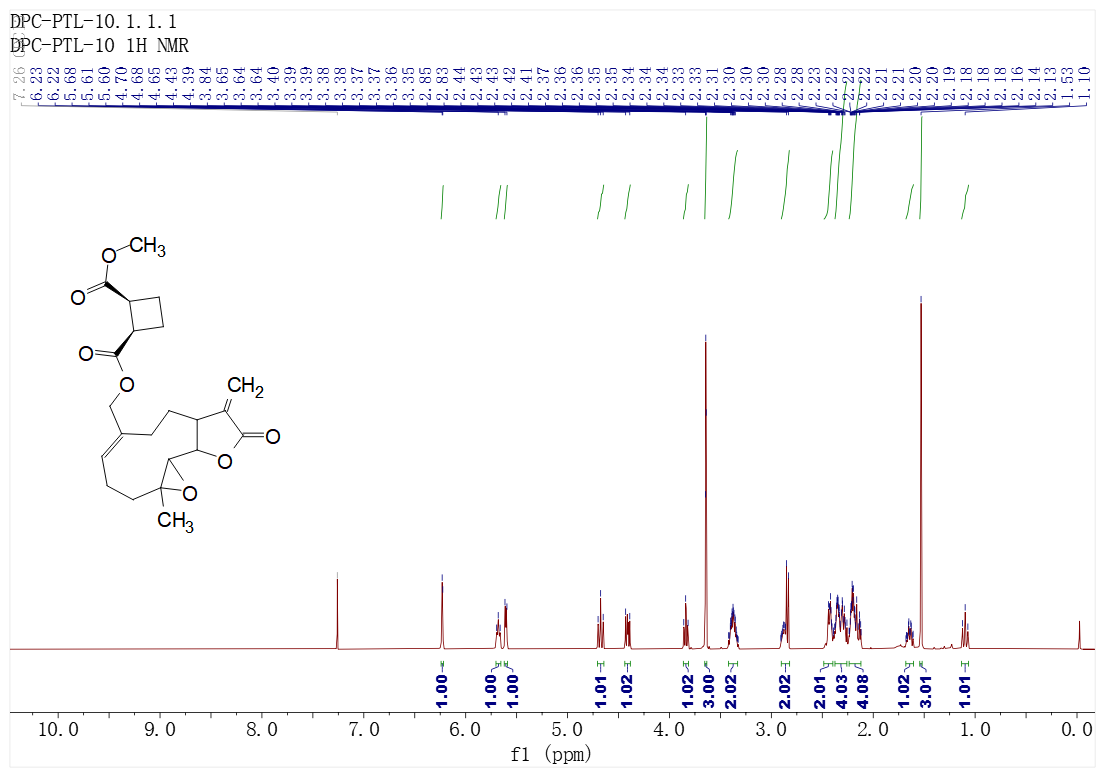


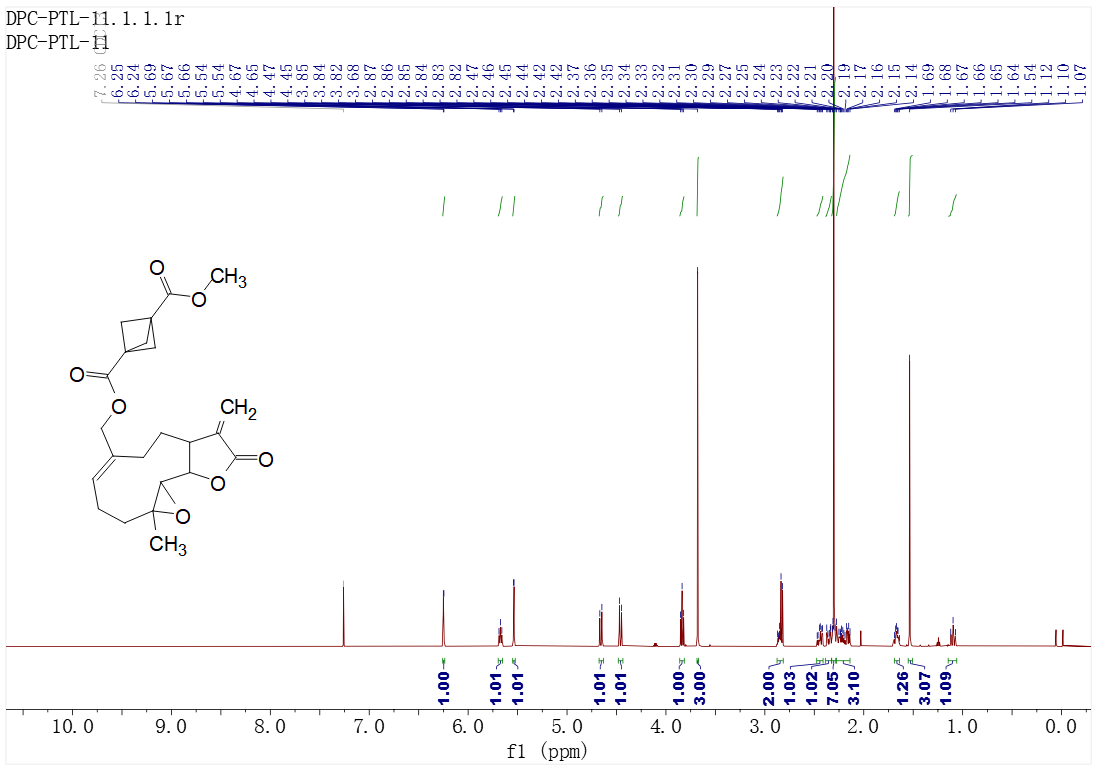


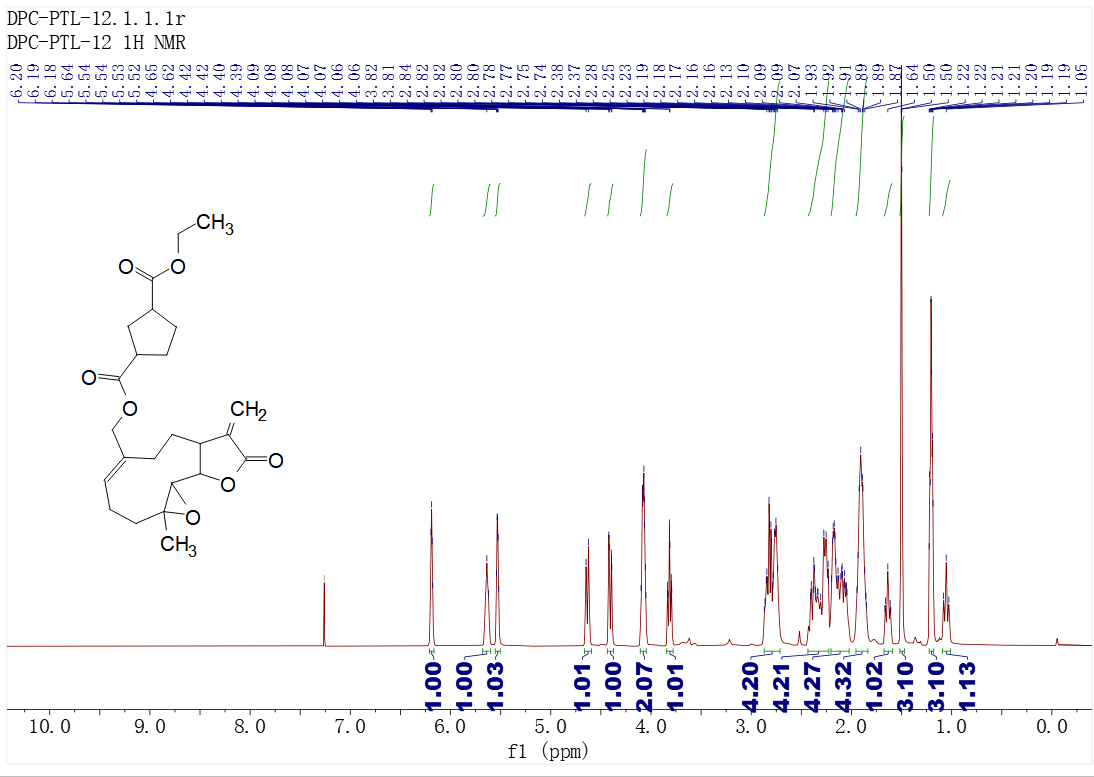


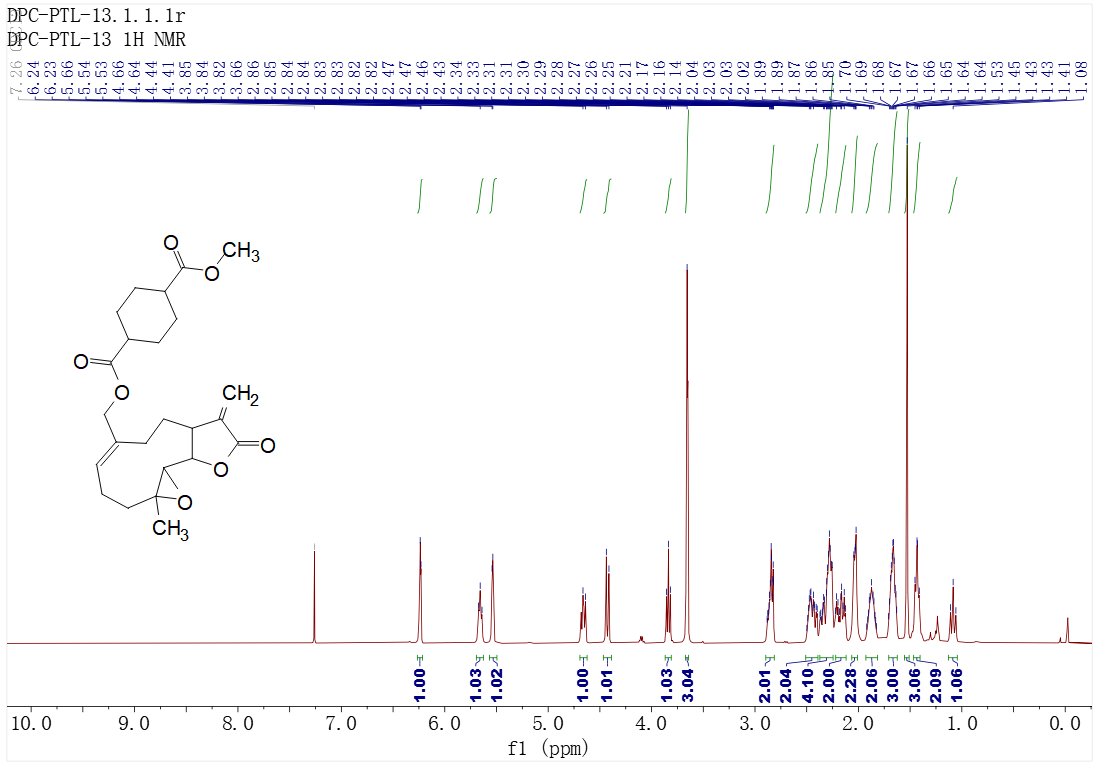


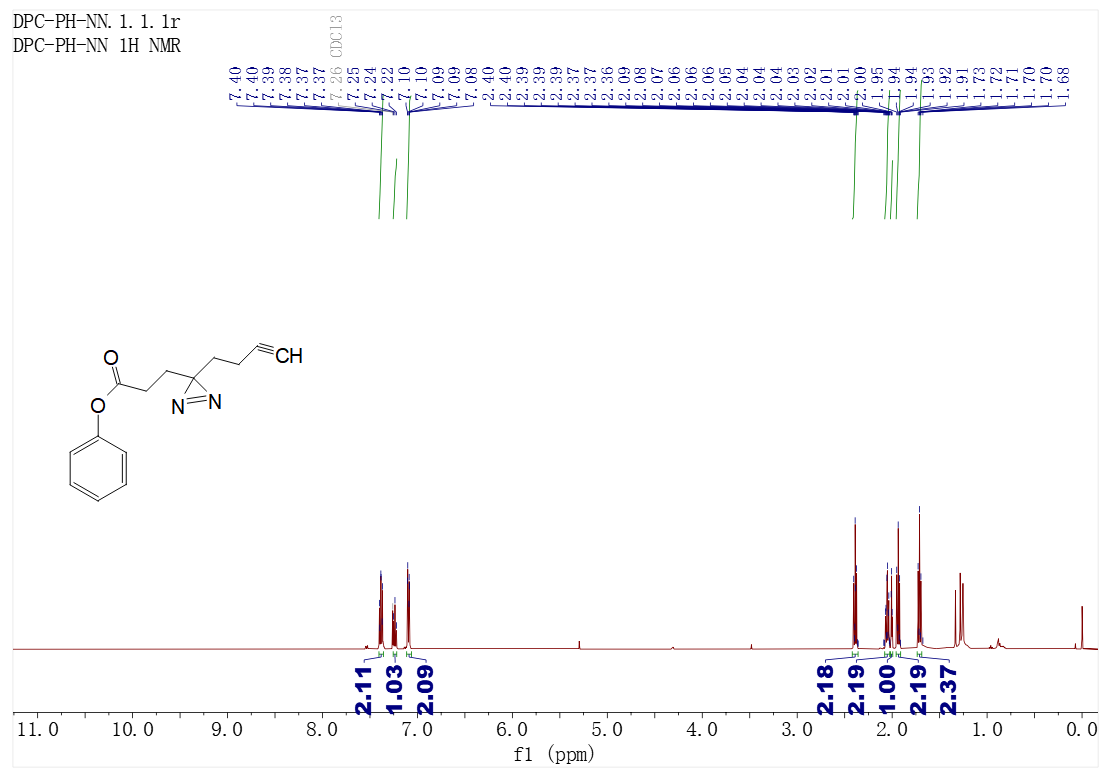


***^13^C NMR spectra of compounds***

**
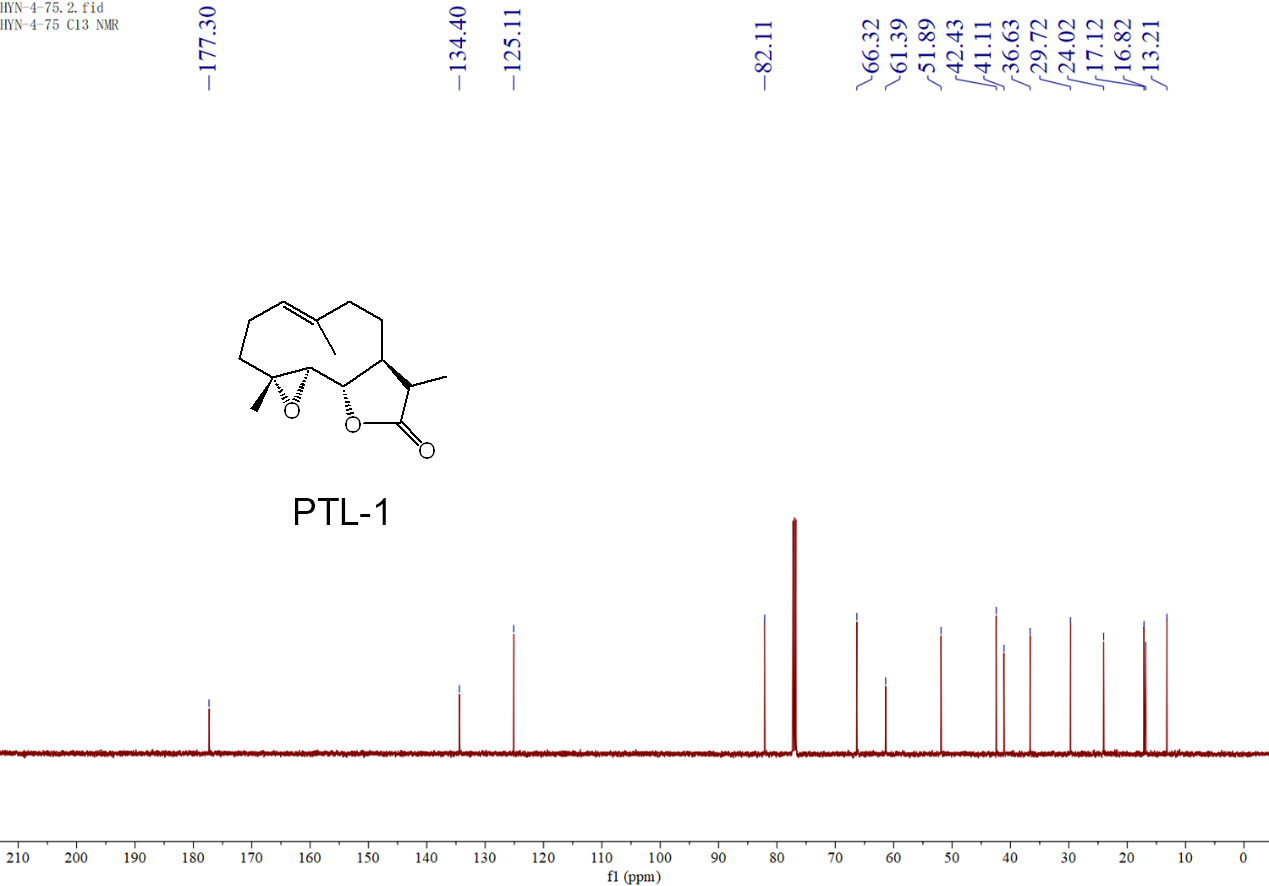
**


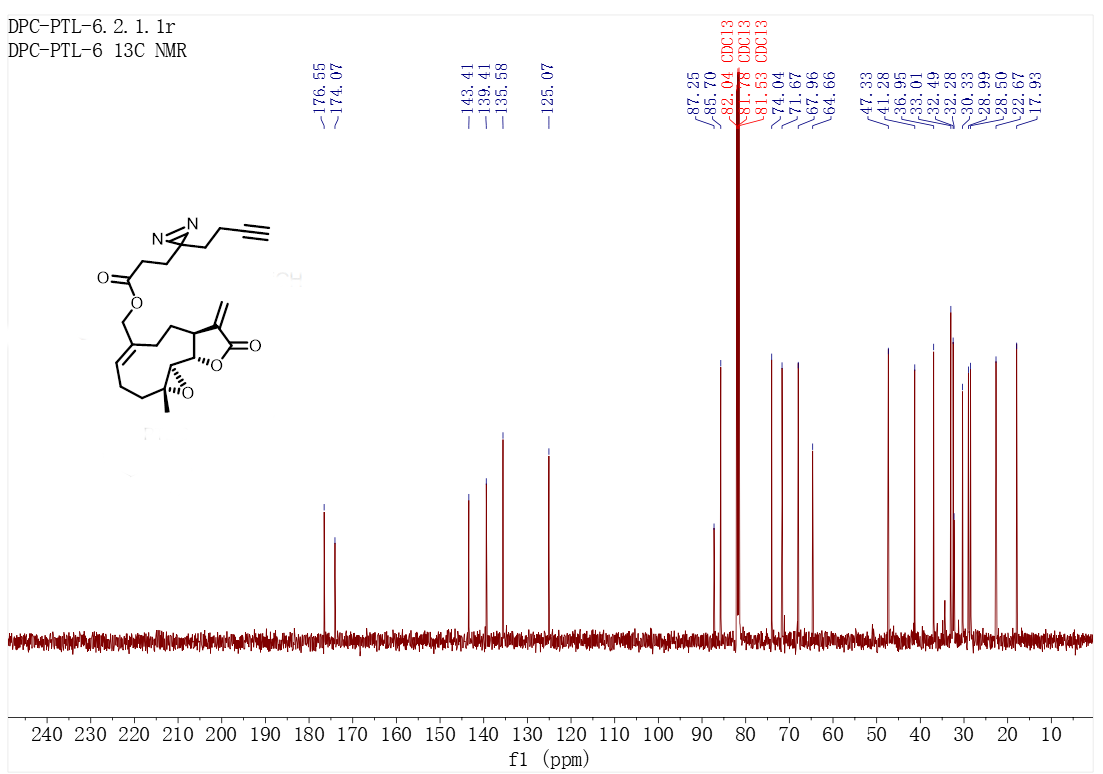


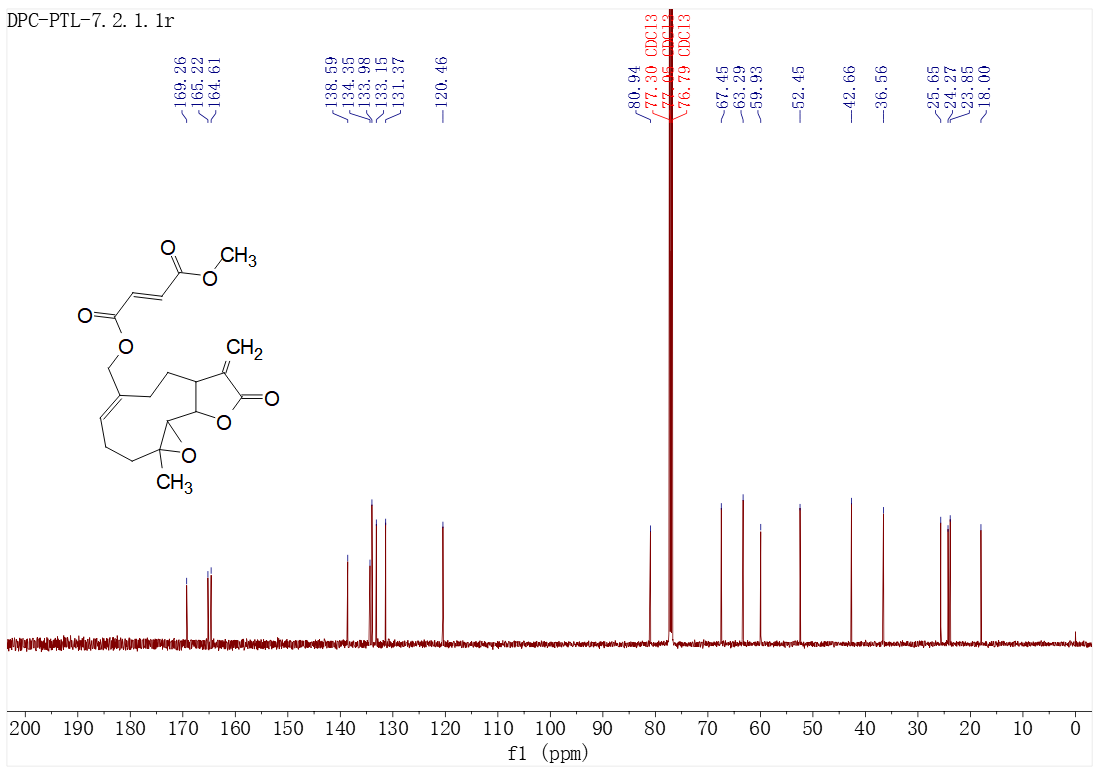


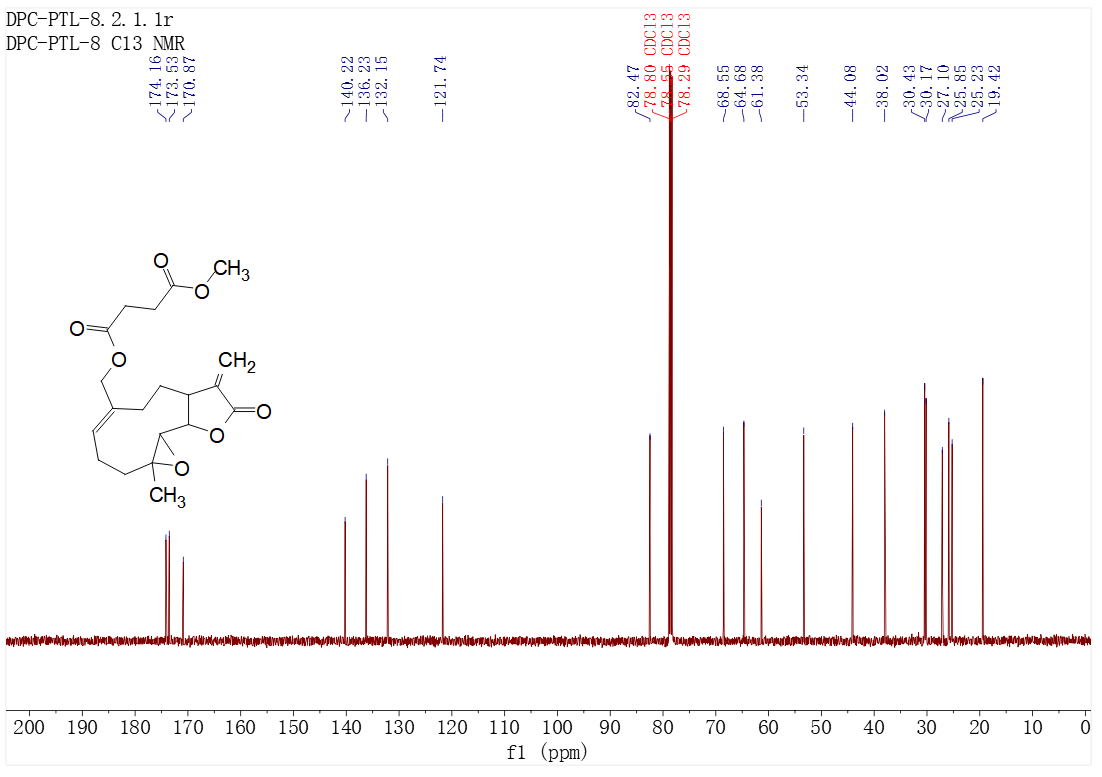


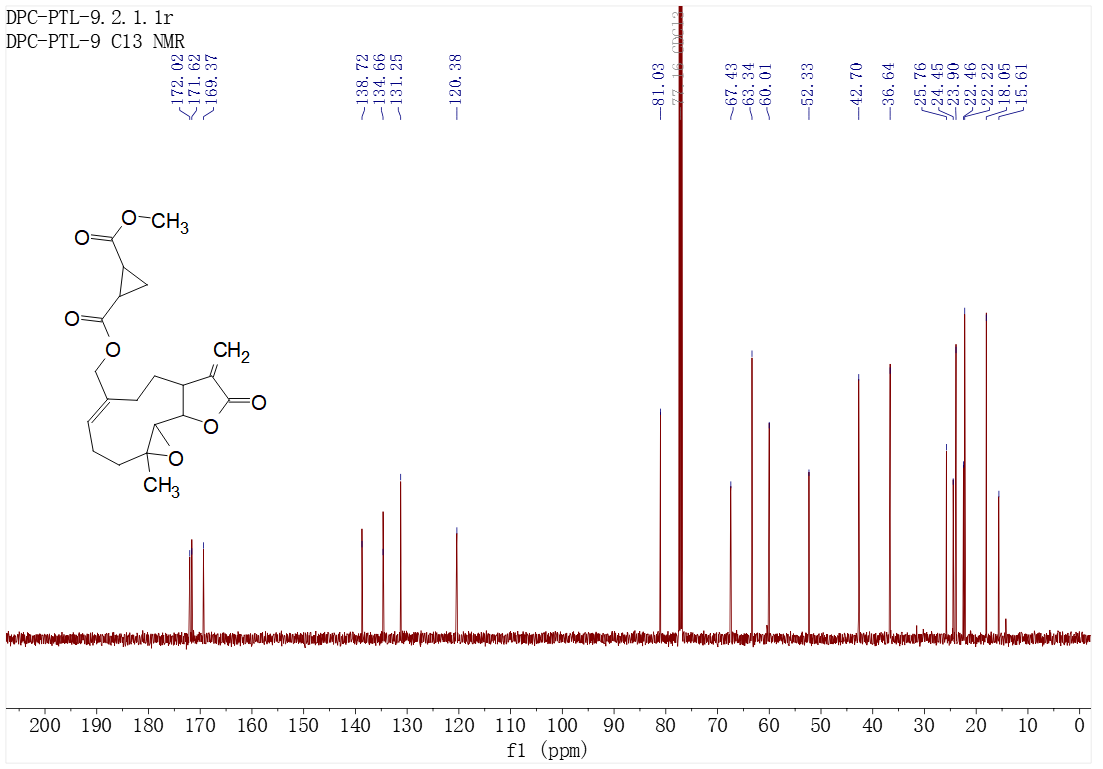


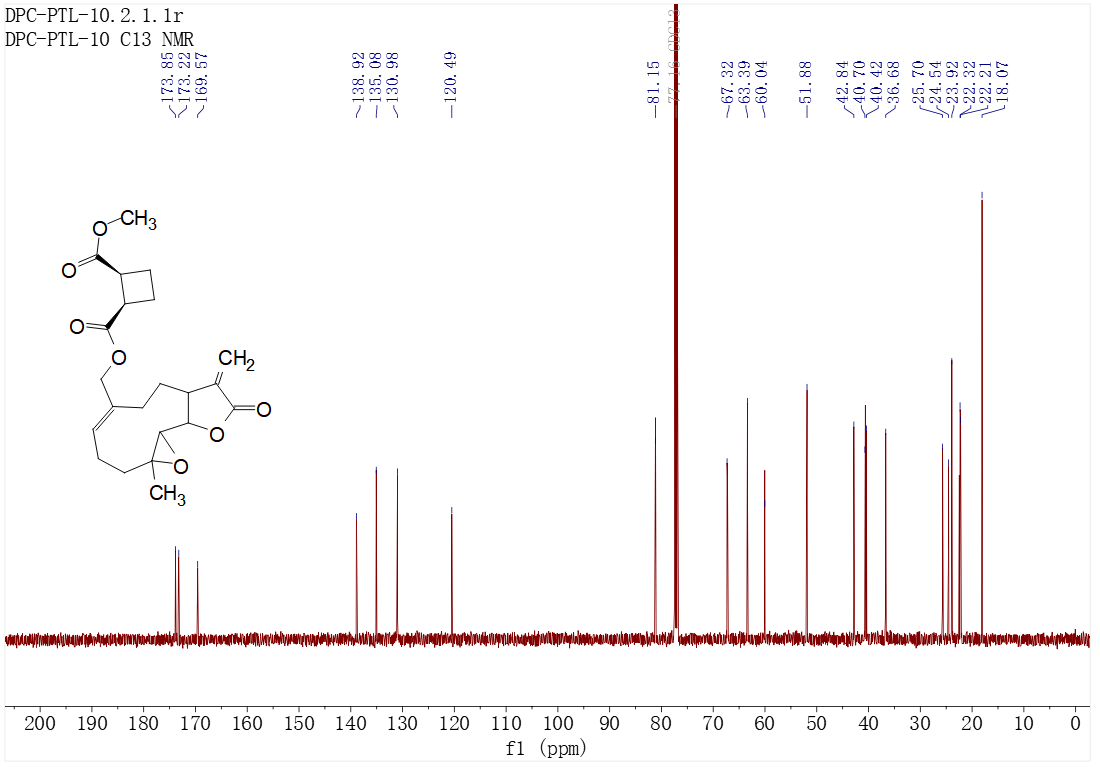


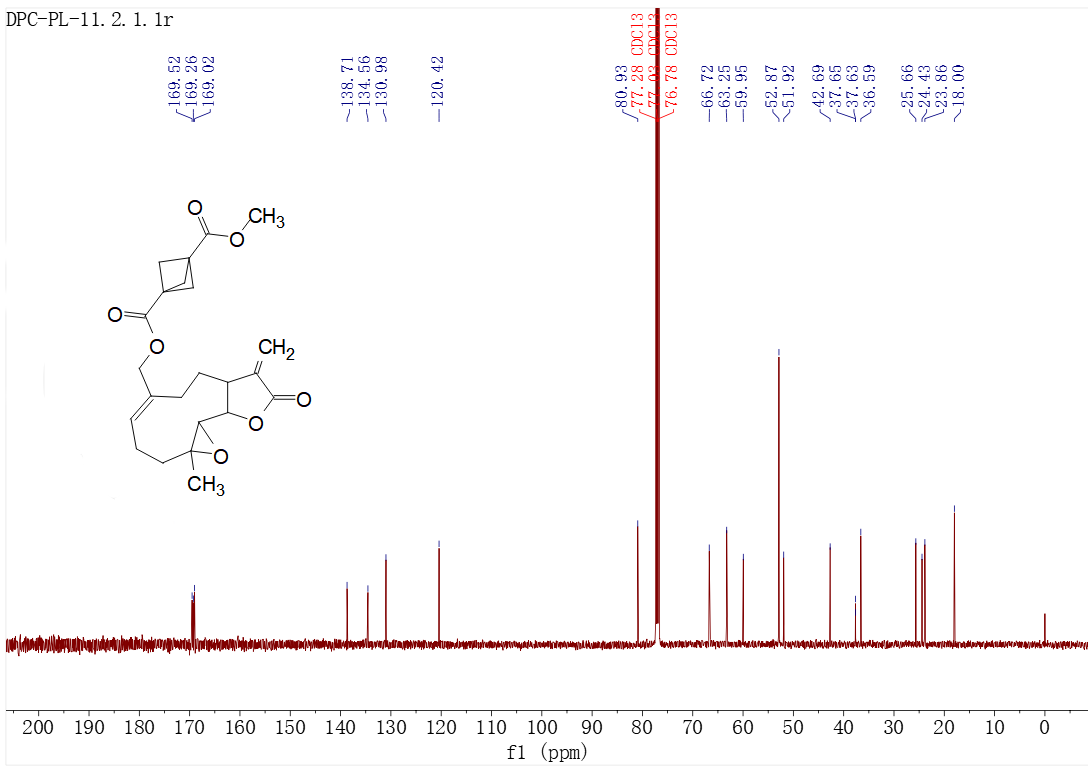


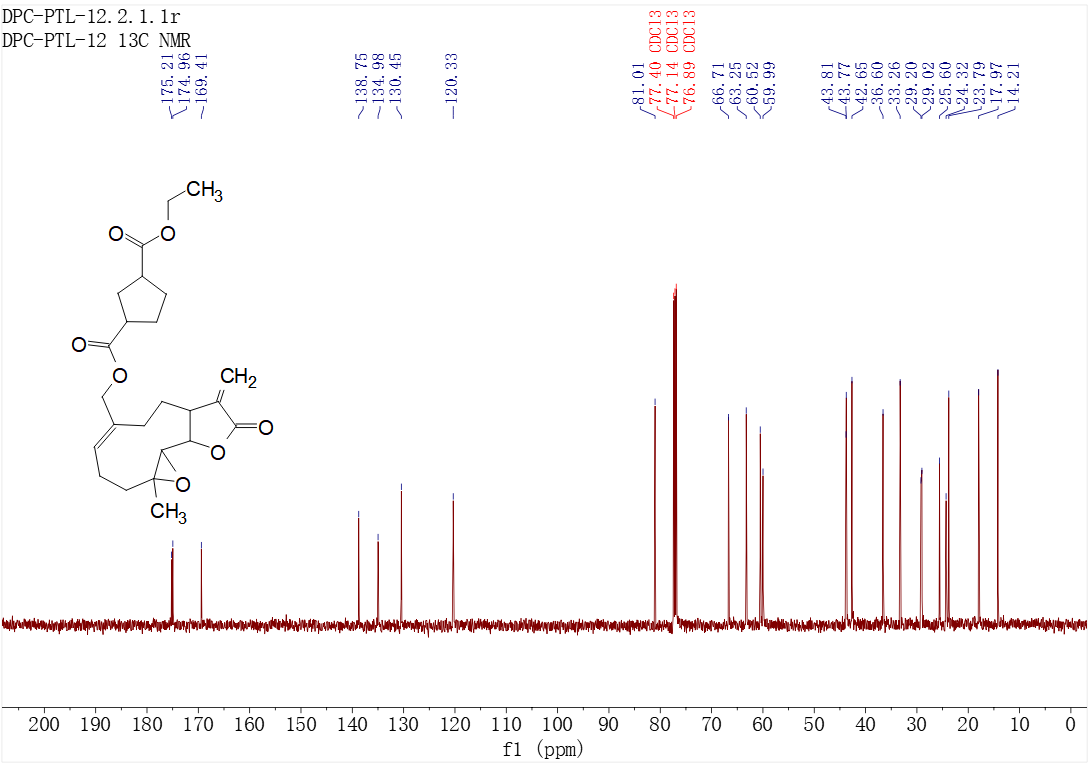


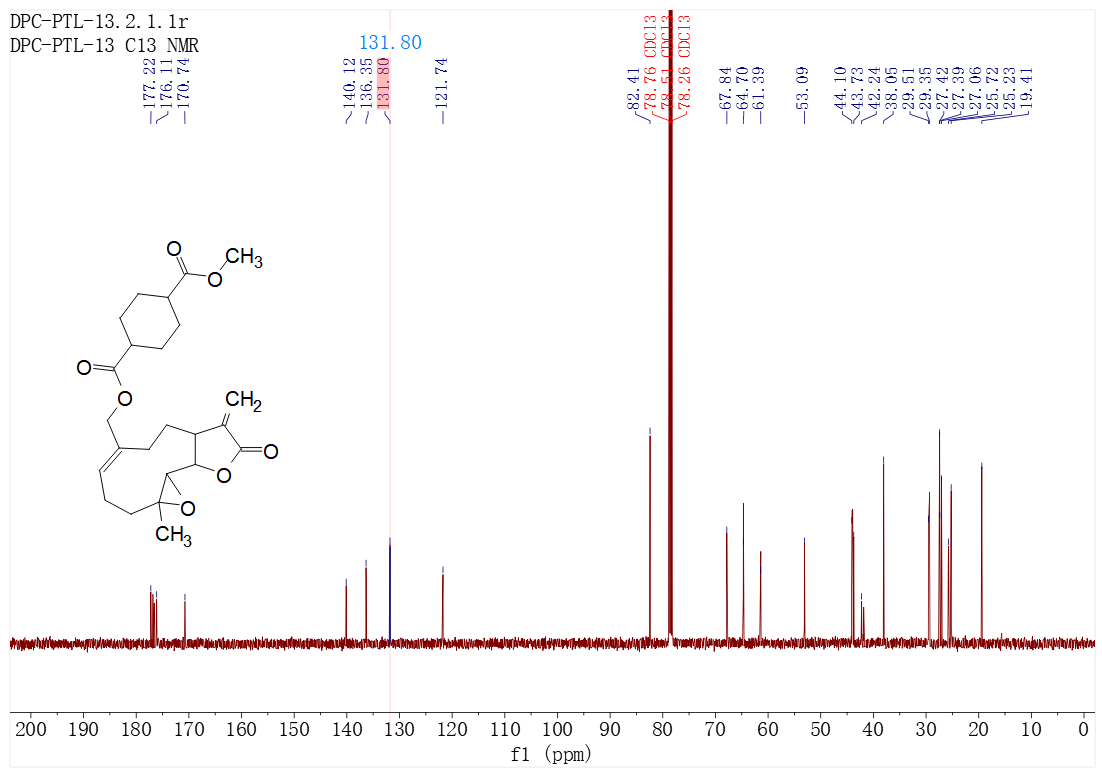


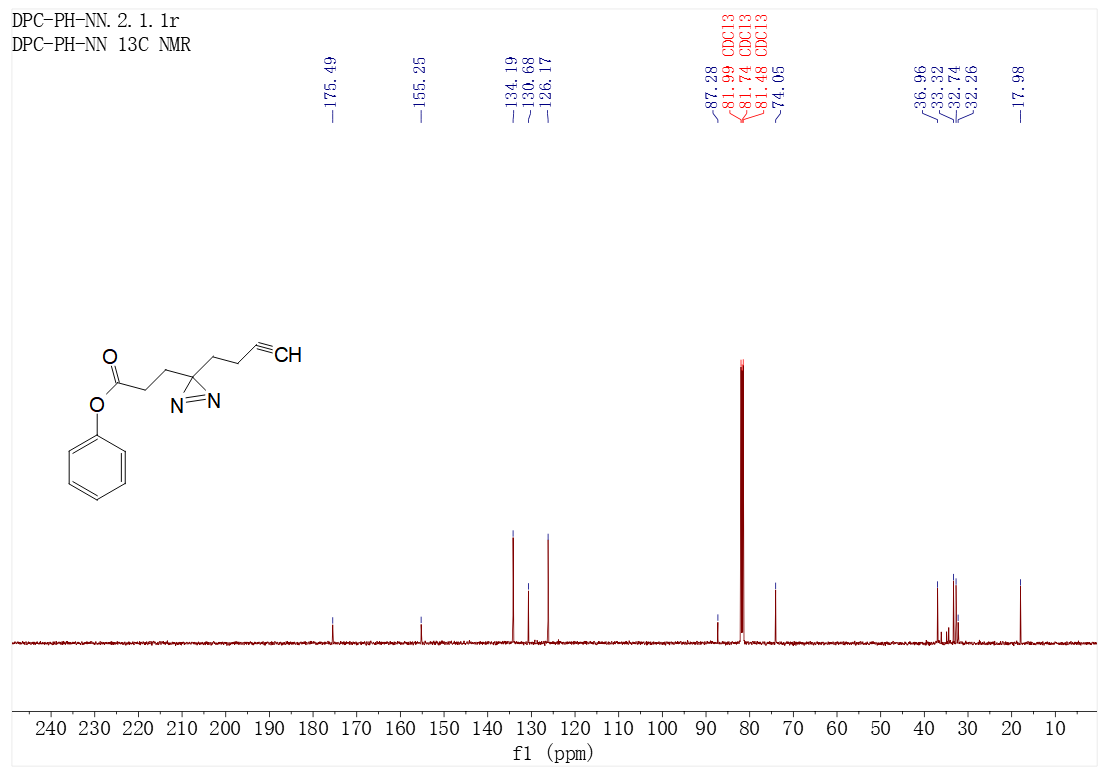


Qualitative analysis report

***HPLC purity spectra of compounds***

**PTL-1**


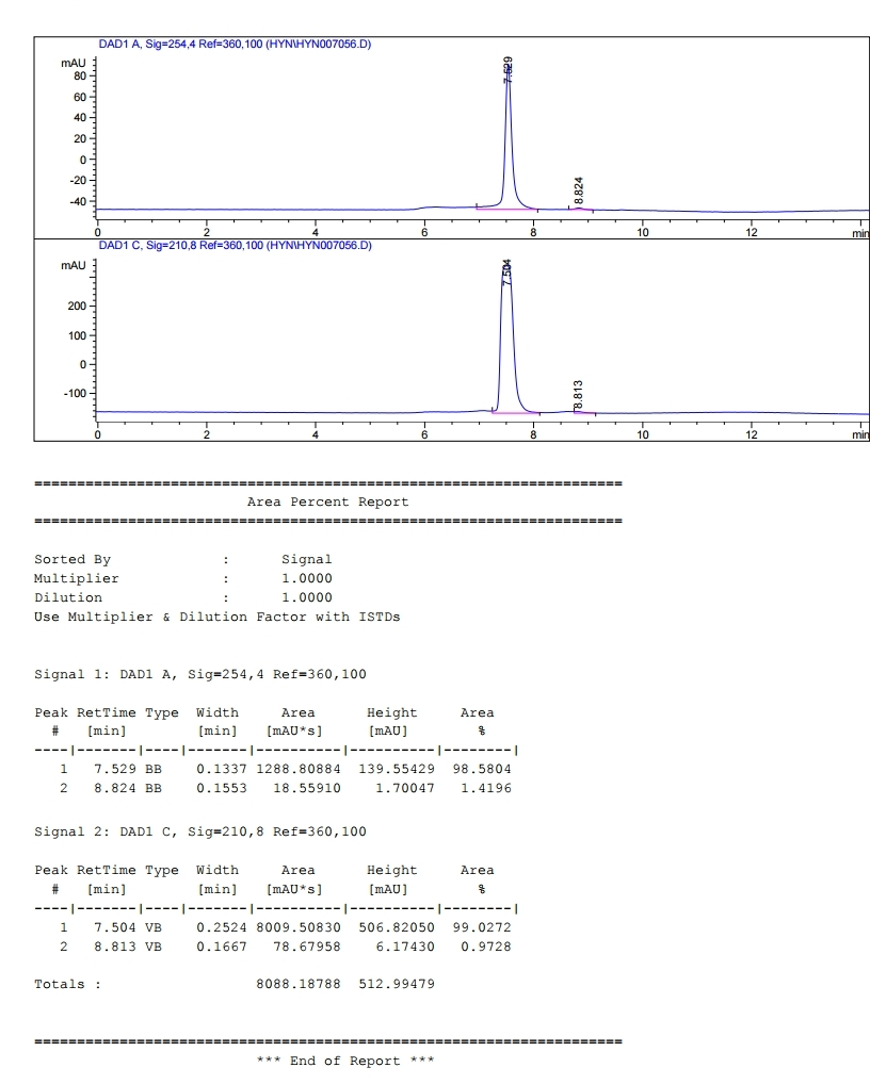


**PTL-6**


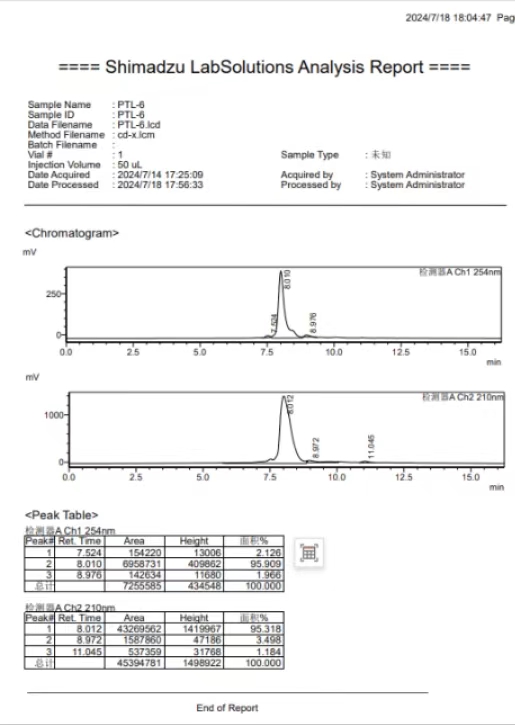


**PTL-7**


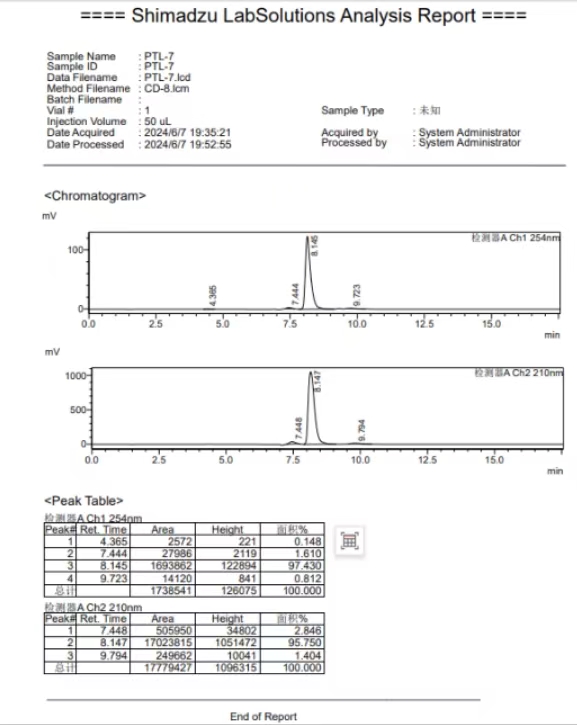


**PTL-8**

**
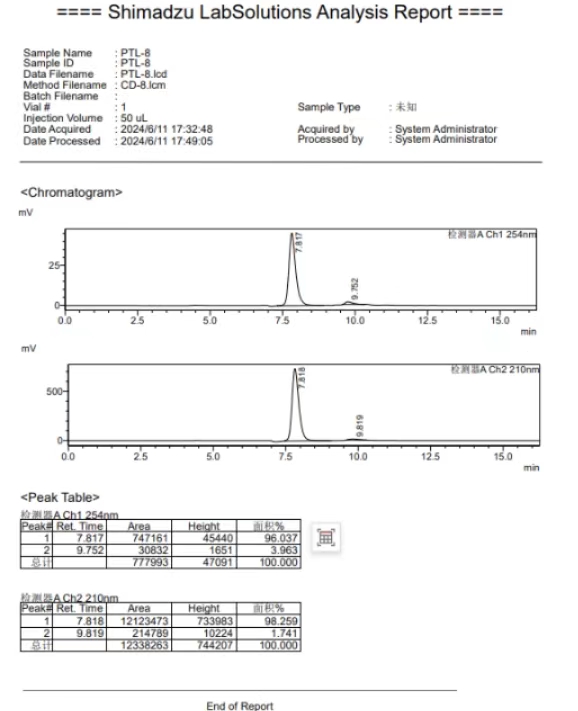
**

**PTL-9**


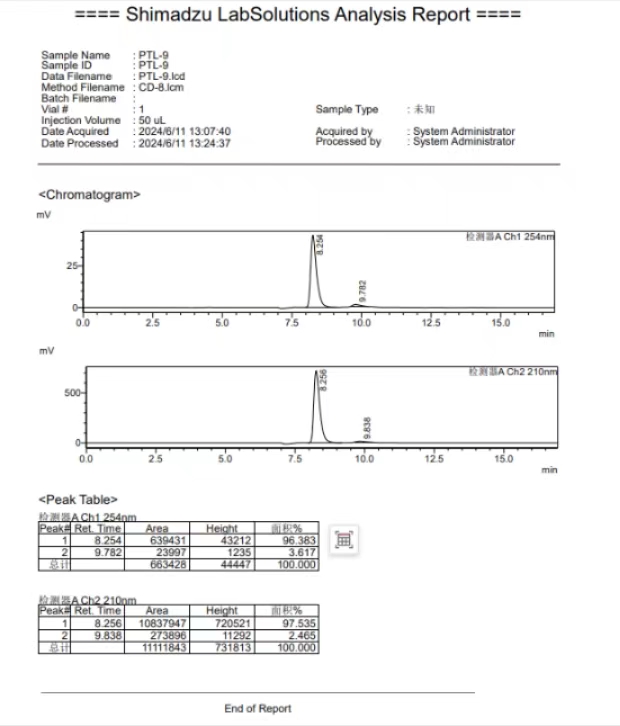


**PTL-10**


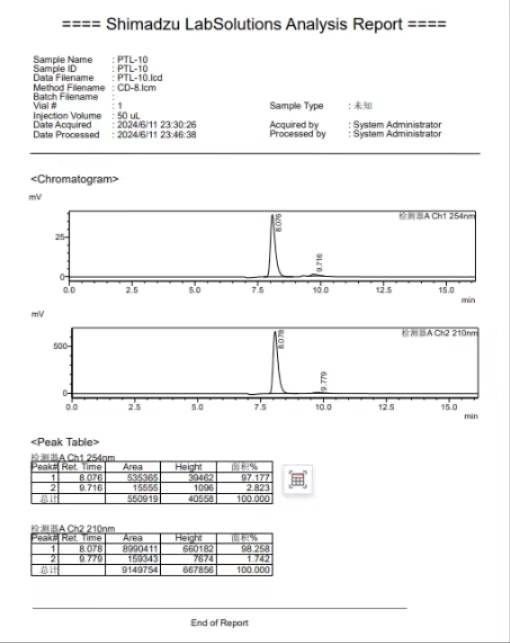


**PTL-11**


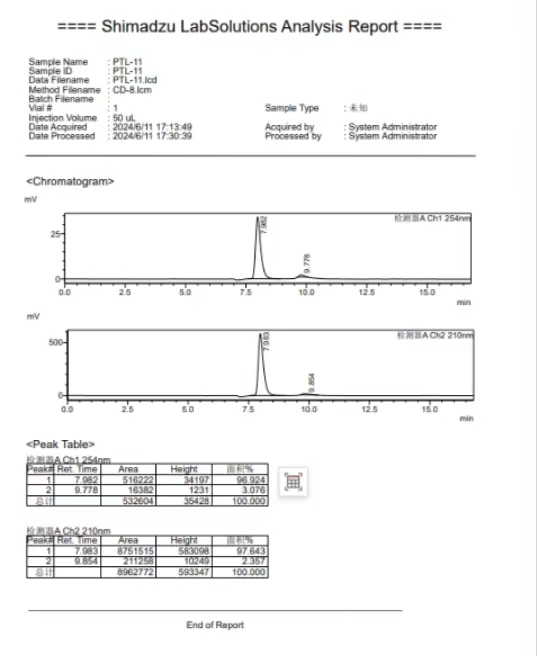


**PTL-12**


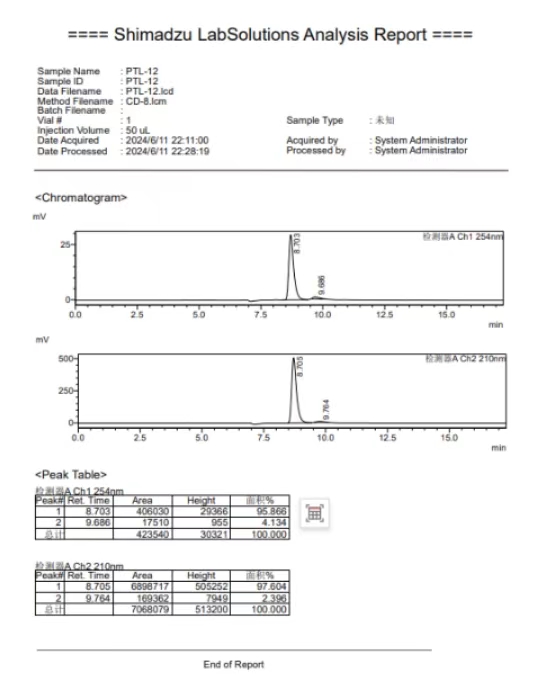


**PTL-13**


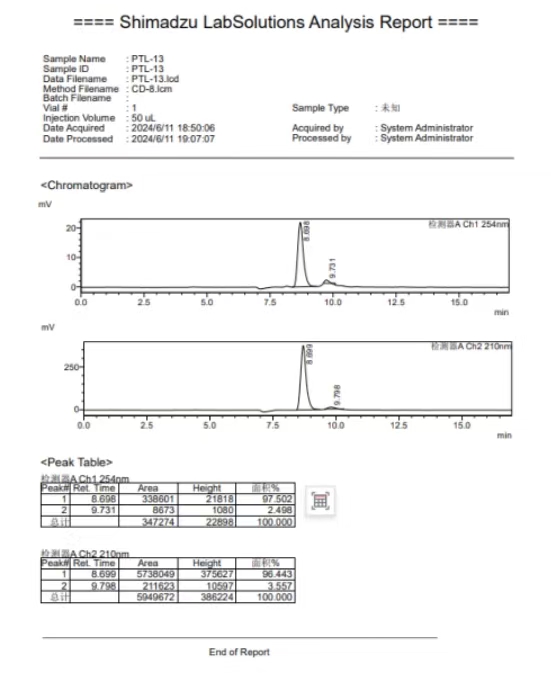


**PH-NN**


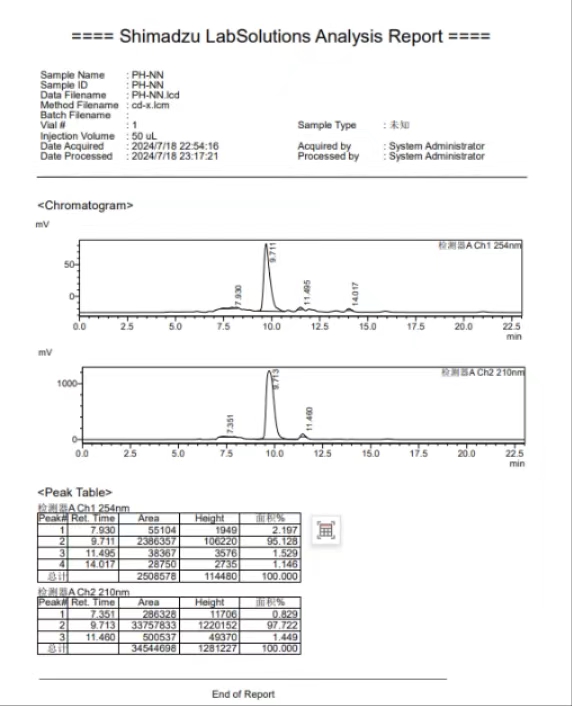

Supplement: Supplementary file 8 — Supplemental Materials [file 41419_2026_8764_MOESM8_ESM.docx]
