# Supplementary material for: Identification of human MLKL Cys184 and HSPBP1 Cys201 as novel cellular targets for necroptosis
Source: Cell Death Dis. 2026 Apr 22;17(1):528. doi: 10.1038/s41419-026-08764-4 (PMC13230738; doi:10.1038/s41419-026-08764-4)
Supplement: Supplementary file 9 — Supplementary Information Tables [file 41419_2026_8764_MOESM9_ESM.docx]

**Supplemental Table 1 Candidate covalent protein targets and labeled peptides of PTL identified by MTRP in HT-29 cells**

| **Annotated Sequence** | **Modifications** | **# PSMs** | **Master Protein Accessions** | **Modifications in Master Proteins** | **Abundance: DMSO** | | | **Abundance: PTL** | | | **Abundance Ratio: (DMSO) / (PTL)** | **Abundance Ratio P-Value: (DMSO) / (PTL)** |
| --- | --- | --- | --- | --- | --- | --- | --- | --- | --- | --- | --- | --- |
|  |  |  |  |  | **1** | **2** | **3** | **1** | **2** | **3** |  |  |
| **[R].VVC*SSGAVGNYSGGLAVK.[E]** | **1xDBIA_C [C3]** | **2** | **P16455** | **P16455_C150** | **714.4** | **691.3** | **718.7** | **99.3** | **124.3** | **105.1** | **6.7973359** | **7.81E-07** |
| **[R].AAC*CLAGAR.[A]** | **1xDBIA_C [C3]** | **2** | **O95336** | **O95336_C32** | **573** | **570.4** | **577.1** | **228.8** | **244.8** | **271.3** | **2.3406863** | **1.32E-05** |
| **[-].MVLC*PVIGK.[L]** | **1xDBIA_C [C4]** | **2** | **O95671** | **O95671_C4** | **1961.9** | **1956.8** | **1839.8** | **670.1** | **780.1** | **741.9** | **2.6375522** | **2.05E-05** |
| **[K].WDFTPC*K.[N]** | **1xDBIA_C [C6]** | **1** | **P38606** | **P38606_C138** | **458.5** | **462.8** | **420.4** | **102.1** | **134.5** | **105.8** | **4.1175318** | **3.93E-05** |
| **[K].RVPC*AYDK.[T]** | **1xDBIA_C [C4]** | **4** | **P46109** | **P46109_C249** | **1317.9** | **1272.1** | **1226.4** | **478.7** | **562.6** | **569.9** | **2.312511** | **4.87E-05** |
| **[K].LFTNDC*IFLK.[K]** | **1xDBIA_C [C6]** | **1** | **Q8IV50** | **Q8IV50_C106** | **530** | **484.2** | **507.2** | **228.4** | **239.3** | **254.3** | **2.1195153** | **6.22E-05** |
| **[K].VYSC*LNNK.[L]** | **1xDBIA_C [C4]** | **2** | **Q5UIP0** | **Q5UIP0_C880** | **836.3** | **759** | **781.6** | **147.8** | **182.8** | **238.2** | **4.2757112** | **6.61E-05** |
| **[R].EGSC*SDFQFK.[L]** | **1xDBIA_C [C4]** | **2** | **Q8WVV4** | **Q8WVV4_C496** | **1211.2** | **1102.9** | **1085.8** | **286.2** | **376.4** | **301.7** | **3.6556182** | **7.24E-05** |
| **[K].QYTTTTC*VQLQK.[Q]** | **1xDBIA_C [C7]** | **1** | **P42858** | **P42858_C1441** | **348.4** | **377.9** | **345.7** | **125.7** | **145.4** | **156.1** | **2.420884** | **9.43E-05** |
| **[K].LLAPDC*EIIQEVGK.[L]** | **1xDBIA_C [C6]** | **1** | **Q9NQT5** | **Q9NQT5_C215** | **592.6** | **598.9** | **570.1** | **205** | **268.1** | **262.3** | **2.2592451** | **0.000099** |
| **[K].YSAADC*PLSFLISEK.[D]** | **1xDBIA_C [C6]** | **1** | **O14867** | **O14867_C646** | **332.3** | **339.4** | **309.4** | **137.9** | **161.2** | **155.8** | **2.1328626** | **0.000107** |
| **[R].VFC*ELK.[W]** | **1xDBIA_C [C3]** | **1** | **Q96L92** | **Q96L92_C519** | **1450.7** | **1364.5** | **1305.2** | **354.1** | **476.2** | **504.7** | **2.8743808** | **0.00012** |
| **[R].LTC*EISTR.[G]** | **1xDBIA_C [C3]** | **1** | **Q9BPY3** | **Q9BPY3_C319** | **471.4** | **467** | **413.6** | **157.5** | **174.4** | **139** | **2.9650794** | **0.000157** |
| **[K].GALEGSSC*PFR.[T]** | **1xDBIA_C [C8]** | **3** | **P30519** | **P30519_C282** | **766.5** | **725.2** | **790.4** | **185.5** | **216.3** | **308.4** | **3.5436893** | **0.000227** |
| **[R].VAMEEQLGTC*QDMEK.[R]** | **1xDBIA_C [C10]** | **1** | **P25054** | **P25054_C207** | **153.6** | **152.4** | **142.8** | **58.5** | **79.2** | **69** | **2.2086957** | **0.000303** |
| **[R].WLSC*TATPQIEEEVSLTQK.[N]** | **1xDBIA_C [C4]** | **1** | **P52948** | **P52948_C1312** | **395.6** | **400.2** | **374.3** | **184.6** | **224.7** | **176.2** | **2.1242906** | **0.000327** |
| **[R].RDDLSFC*QEMK.[F]** | **1xDBIA_C [C7]** | **2** | **P21359** | **P21359_C1032** | **935.5** | **965.1** | **823.1** | **338.2** | **396.9** | **397.6** | **2.4273139** | **0.000367** |
| **[R].MQVSETC*TGPMLQAK.[M]** | **1xDBIA_C [C7]** | **1** | **Q8WVV4** | **Q8WVV4_C444** | **172.1** | **160.7** | **157.3** | **62.7** | **83.5** | **60.8** | **2.5629984** | **0.000381** |
| **[R].EQQVYC*EEMR.[E]** | **1xDBIA_C [C6]** | **1** | **O95140** | **O95140_C390** | **316.5** | **300.7** | **319.8** | **110** | **130.5** | **163.3** | **2.4252874** | **0.000432** |
| **[K].SIQTIC*SGLLTDVEDQAAK.[G]** | **1xDBIA_C [C6]** | **1** | **Q6PCB5** | **Q6PCB5_C280** | **658.5** | **611.9** | **547.9** | **241.5** | **277.2** | **244.3** | **2.3755411** | **0.000495** |
| **[K].SVYLGTGC*GK.[S]** | **1xDBIA_C [C8]** | **1** | **Q9NXV6** | **Q9NXV6_C516** | **685.3** | **688.3** | **577.6** | **226.2** | **279.4** | **256** | **2.5534925** | **0.000555** |
| **[R].KPNVGC*QQDSEELLK.[L]** | **1xDBIA_C [C6]** | **3** | **A0AVT1** | **A0AVT1_C347** | **1483** | **1377.4** | **1382** | **666.8** | **844.8** | **647.8** | **2.0725855** | **0.000632** |
| **[R].VGGLC*LK.[C]** | **1xDBIA_C [C5]** | **1** | **Q86SQ7** | **Q86SQ7_C283** | **475.9** | **482.6** | **413.6** | **219.8** | **242.6** | **198.9** | **2.0794369** | **0.000728** |
| **[K].VACC*PLER.[C]** | **1xDBIA_C [C4]** | **2** | **Q15021** | **Q15021_C767** | **1397.8** | **1352.1** | **1170.6** | **529.4** | **631.3** | **619.1** | **2.2111825** | **0.000734** |
| **[R].SC*PQEEGPR.[G]** | **1xDBIA_C [C2]** | **1** | **P53814** | **P53814_C410** | **590.8** | **591.9** | **510.7** | **230.6** | **296.3** | **255.9** | **2.2146574** | **0.000776** |
| **[R].YVVAVGLEC*GK.[I]** | **1xDBIA_C [C9]** | **1** | **Q6IA86** | **Q6IA86_C746** | **260.8** | **259.4** | **254.3** | **95.5** | **116.8** | **147.5** | **2.2208904** | **0.000815** |
| **[R].QC*LPSLDLSCK.[Q]** | **1xDBIA_C [C2]** | **1** | **P78527** | **P78527_C1499** | **414.1** | **407.8** | **329.3** | **42** | **51** | **114.2** | **7.8404762** | **0.000894** |
| **[K].C*VAYTGNNMR.[K]** | **1xDBIA_C [C1]** | **1** | **Q9Y496** | **Q9Y496_C621** | **352.6** | **367.9** | **307.1** | **166.4** | **184.7** | **152.5** | **2.0137705** | **0.001041** |
| **[K].QVQFPVIQLQELMDDC*SAVLENEK.[L]** | **1xDBIA_C [C16]** | **1** | **Q9H0R6** | **Q9H0R6_C512** | **94.5** | **82.5** | **89.6** | **32.7** | **42** | **48.6** | **2.1333333** | **0.00117** |
| **[K].GC*SNKPTTVNK.[R]** | **1xDBIA_C [C2]** | **2** | **Q8NHV4** | **Q8NHV4_C314** | **713.3** | **631.2** | **626.3** | **248.8** | **313.8** | **366** | **2.0114723** | **0.001405** |
| **[R].SGC*SSQSISPMR.[S]** | **1xDBIA_C [C3]** | **1** | **Q14449** | **Q14449_C360** | **261.4** | **289.8** | **225.8** | **113.9** | **116.4** | **99** | **2.2949956** | **0.001505** |
| **[R].C*VEDAK.[I]** | **1xDBIA_C [C1]** | **1** | **Q8TAA5** | **Q8TAA5_C97** | **685.2** | **693.5** | **534.3** | **218.7** | **254.1** | **222** | **2.7292405** | **0.001555** |
| **[R].AQELC*PENFIHFNNK.[L]** | **1xDBIA_C [C5]** | **1** | **Q8IY21** | **Q8IY21_C1051** | **514** | **463.5** | **509** | **198** | **247.5** | **302.7** | **2.0565657** | **0.001985** |
| **[-].MPC*GEDWLSHPLGIVQGFFAQNGVNPDWEK.[K]** | **1xDBIA_C [C3]** | **1** | **Q9BTE3** | **Q9BTE3_C3** | **193.9** | **186.1** | **157** | **90.8** | **94.7** | **73.7** | **2.0495595** | **0.002023** |
| **[R].TPTC*SSNK.[I]** | **1xDBIA_C [C4]** | **1** | **Q8NDZ2** | **Q8NDZ2_C683** | **301.7** | **281.2** | **260.6** | **139.9** | **176.1** | **125.5** | **2.0100071** | **0.002202** |
| **[R].SSC*SPGGR.[T]** | **1xDBIA_C [C3]** | **2** | **Q6UB35** | **Q6UB35_C61** | **529.1** | **547.9** | **429.8** | **236.2** | **258.1** | **214.7** | **2.1228206** | **0.002354** |
| **[R].ELAHC*QK.[V]** | **1xDBIA_C [C5]** | **1** | **O00411** | **O00411_C765** | **738.6** | **701.6** | **542.8** | **239.4** | **270.5** | **233** | **2.7304991** | **0.0025** |
| **[K].QAC*EIQK.[C]** | **1xDBIA_C [C3]** | **1** | **P56277** | **P56277_C12** | **366.8** | **375.1** | **478.1** | **151.4** | **187.5** | **175.4** | **2.4227213** | **0.003244** |
| **[K].DESATNSGTGATASC*GLGSEFSTDK.[G]** | **1xDBIA_C [C15]** | **1** | **Q9UKJ3** | **Q9UKJ3_C247** | **215.2** | **221.7** | **171.1** | **106** | **104.3** | **87.8** | **2.063279** | **0.003643** |
| **[R].DAC*DTVR.[V]** | **1xDBIA_C [C3]** | **3** | **Q9NZL4** | **Q9NZL4_C201** | **110.9** | **124** | **100.4** | **53.9** | **68.3** | **48.1** | **2.0575139** | **0.003767** |
| **[R].YC*NLQK.[Y]** | **1xDBIA_C [C2]** | **1** | **O14879** | **O14879_C365** | **837.7** | **903.8** | **731.5** | **415** | **485.5** | **317.6** | **2.0185542** | **0.003923** |
| **[K].C*KPVPLLELAEGQK.[Q]** | **1xDBIA_C [C1]** | **2** | **Q8NHV4** | **Q8NHV4_C66** | **757.1** | **838.2** | **575.1** | **268.6** | **289.9** | **228.2** | **2.8186895** | **0.004464** |
| **[K].C*SSLYEER.[Y]** | **1xDBIA_C [C1]** | **1** | **Q9Y4D7** | **Q9Y4D7_C1363** | **364.5** | **425.4** | **277.7** | **80.3** | **102.9** | **124.4** | **3.4582814** | **0.004783** |
| **[K].C*MQEIPQEQIK.[E]** | **1xDBIA_C [C1]** | **2** | **Q8NB16** | **Q8NB16_C184** | **568.9** | **618.6** | **459.4** | **257.5** | **299.1** | **239** | **2.0682046** | **0.00485** |
| **[K].HLFC*LLSK.[S]** | **1xDBIA_C [C4]** | **1** | **Q6YHU6** | **Q6YHU6_C1853** | **316.4** | **307.5** | **266.6** | **153.1** | **189.6** | **120.1** | **2.0084912** | **0.004851** |
| **[K].C*MLGLLLGK.[I]** | **1xDBIA_C [C1]** | **1** | **Q92564** | **Q92564_C219** | **887** | **997.5** | **707.9** | **356.2** | **399.7** | **265.1** | **2.4956217** | **0.004934** |
| **[K].QAC*EGNLPK.[G]** | **1xDBIA_C [C3]** | **1** | **Q9H1A4** | **Q9H1A4_C988** | **1283.8** | **1345.3** | **988.4** | **603.1** | **666** | **534.9** | **2.01997** | **0.006562** |
| **[-].MAGC*AAR.[A]** | **1xDBIA_C [C4]** | **2** | **O60725** | **O60725_C4** | **242.6** | **247.3** | **241.5** | **69.9** | **93.8** | **158.6** | **2.5863539** | **0.006815** |
| **[R].HHVPQQC*NK.[M]** | **1xDBIA_C [C7]** | **2** | **Q9NR09** | **Q9NR09_C3673** | **114.1** | **119.4** | **83.9** | **41.2** | **53.3** | **35.6** | **2.3567416** | **0.006955** |
| **[K].QEAQNEQTSEPSNMDGNSGDADC*FQPAVK.[R]** | **1xDBIA_C [C23]** | **1** | **P32780** | **P32780_C344** | **223.6** | **265** | **184.7** | **83.5** | **100.7** | **112** | **2.2204568** | **0.006956** |
| **[R].TASEDSC*K.[R]** | **1xDBIA_C [C7]** | **1** | **P15924** | **P15924_C1589** | **433.6** | **516** | **350.4** | **180.9** | **196.8** | **124.7** | **2.6219512** | **0.007194** |
| **[K].NLAC*EESK.[R]** | **1xDBIA_C [C4]** | **2** | **O95347** | **O95347_C326** | **808.7** | **945.3** | **564.1** | **214.3** | **243.1** | **151.9** | **3.7736818** | **0.007675** |
| **[R].C*ANVNNSSTTSQR.[I]** | **1xDBIA_C [C1]** | **2** | **Q5VZ89** | **Q5VZ89_C126** | **227** | **275.7** | **197.4** | **77.9** | **90.2** | **128.6** | **2.5166297** | **0.008065** |
| **[R].C*QYQER.[L]** | **1xDBIA_C [C1]** | **1** | **Q13439** | **Q13439_C1771** | **645.1** | **738.9** | **471.7** | **225** | **248.1** | **229** | **2.8170306** | **0.008085** |
| **[R].C*VNNYQGMLK.[V]** | **1xDBIA_C [C1]** | **1** | **O75663** | **O75663_C75** | **446.5** | **523.5** | **366** | **218.5** | **238.8** | **180.9** | **2.0434783** | **0.008693** |
| **[R].TC*AGESK.[D]** | **1xDBIA_C [C2]** | **1** | **O95425** | **O95425_C178** | **505.2** | **587.2** | **410.6** | **244.7** | **274.4** | **232.8** | **2.0645689** | **0.008848** |
| **[K].C*PFYAAEQDK.[G]** | **1xDBIA_C [C1]** | **3** | **P30519** | **P30519_C265** | **820** | **976.7** | **628.7** | **319.3** | **362** | **311.8** | **2.5681178** | **0.009388** |
| **[K].ESDQAC*GK.[M]** | **1xDBIA_C [C6]** | **1** | **Q9H930** | **Q9H930_C191** | **347.4** | **414.8** | **284.6** | **152.3** | **186** | **164.7** | **2.1092896** | **0.009557** |
| **[K].C*EMETEYQPK.[R]** | **1xDBIA_C [C1]** | **1** | **Q96E09** | **Q96E09_C206** | **210.2** | **246.4** | **168.8** | **100.5** | **110.3** | **77.1** | **2.1893645** | **0.010073** |
| **[K].NQLC*DLETK.[L]** | **1xDBIA_C [C4]** | **2** | **P26358** | **P26358_C62** | **1659.5** | **2043.3** | **1154.1** | **538** | **580.4** | **348.7** | **3.3097218** | **0.013376** |
| **[R].NC*LDLYEEILTEEGTAK.[E]** | **1xDBIA_C [C2]** | **1** | **Q9UKL3** | **Q9UKL3_C54** | **33.7** | **41.5** | **27.2** | **17.6** | **16.8** | **13.3** | **2.0451128** | **0.013677** |
| **[R].FQC*VFQVLPK.[C]** | **1xDBIA_C [C3]** | **1** | **Q8WXE1** | **Q8WXE1_C585** | **313.9** | **331.9** | **229.7** | **157.1** | **163** | **114.3** | **2.0096238** | **0.013734** |
| **[K].C*PLASTNK.[R]** | **1xDBIA_C [C1]** | **2** | **P0DPB5** | **P0DPB5_C37** | **770** | **893.3** | **569.8** | **332.5** | **390** | **268.4** | **2.2905128** | **0.014657** |
| **[K].TEQDHC*NVK.[A]** | **1xDBIA_C [C6]** | **1** | **Q8IVU3** | **Q8IVU3_C557** | **462.3** | **522.8** | **414.9** | **163.9** | **209.4** | **328.4** | **2.2077364** | **0.016081** |
| **[K].TC*GSTASMK.[V]** | **1xDBIA_C [C2]** | **1** | **A7KAX9** | **A7KAX9_C93** | **416.9** | **473.1** | **323.1** | **224.9** | **224.6** | **131.5** | **2.1036016** | **0.017163** |
| **[K].C*NEQPNRVEIYEK.[T]** | **1xDBIA_C [C1]** | **3** | **Q7L576** | **Q7L576_C98** | **476.5** | **639.6** | **389.9** | **216** | **232** | **162.7** | **2.3964352** | **0.017254** |
| **[K].C*NEQPNRVEIYEK.[T]** | **1xDBIA_C [C1]** | **3** | **Q96F07** | **Q96F07_C98** | **476.5** | **639.6** | **389.9** | **216** | **232** | **162.7** | **2.3964352** | **0.017254** |
| **[K].C*QALEENNLSLR.[H]** | **1xDBIA_C [C1]** | **2** | **Q8WVV4** | **Q8WVV4_C398** | **553** | **690.4** | **432.8** | **259.5** | **290.5** | **231.6** | **2.1310212** | **0.017448** |
| **[K].ASEQIYGTPSSSPYEC*LR.[Q]** | **1xDBIA_C [C16]** | **1** | **Q14203** | **Q14203_C888** | **1272.3** | **1496.3** | **983.9** | **612.9** | **738.3** | **611.1** | **2.0266829** | **0.017983** |
| **[R].C*YGGLWEK.[D]** | **1xDBIA_C [C1]** | **1** | **Q9BT40** | **Q9BT40_C213** | **1257.2** | **1445.7** | **937.8** | **647.4** | **659.4** | **453.9** | **2.0660939** | **0.018204** |
| **[K].C*QLLFALK.[V]** | **1xDBIA_C [C1]** | **1** | **Q9BRJ7** | **Q9BRJ7_C171** | **821.7** | **1026.5** | **653.6** | **426.9** | **446.7** | **312.1** | **2.0942006** | **0.019239** |
| **[K].NNQESDC*VSK.[K]** | **1xDBIA_C [C7]** | **3** | **A6NDG6** | **A6NDG6_C297** | **838.8** | **1081.9** | **588.2** | **307.6** | **322.7** | **152.2** | **3.3526495** | **0.01958** |
| **[K].NC*QQGAAYK.[T]** | **1xDBIA_C [C2]** | **1** | **Q9Y4C1** | **Q9Y4C1_C695** | **328.6** | **404.6** | **214.6** | **116.4** | **118.6** | **63.4** | **3.384858** | **0.020357** |
| **[R].C*GCLDEDTQR.[Q]** | **1xDBIA_C [C1]** | **1** | **P58107** | **P58107_C421** | **200.4** | **288.2** | **184.6** | **105.3** | **114.9** | **90** | **2.0511111** | **0.021517** |
| **[K].NQEPSSC*K.[S]** | **1xDBIA_C [C7]** | **3** | **Q9GZU8** | **Q9GZU8_C187** | **660.9** | **767.8** | **485.2** | **325.7** | **363.9** | **247.2** | **2.0291679** | **0.02179** |
| **[K].C*DFEFK.[A]** | **1xDBIA_C [C1]** | **1** | **O94763** | **O94763_C167** | **956.9** | **1150** | **688.7** | **461.1** | **464.9** | **298.3** | **2.3087496** | **0.022307** |
| **[K].C*ALGWDHQEK.[L]** | **1xDBIA_C [C1]** | **5** | **Q14247** | **Q14247_C246** | **1484.4** | **1723.4** | **1098** | **767.4** | **809.2** | **498.6** | **2.1297578** | **0.022767** |
| **[K].C*EILQSDSR.[C]** | **1xDBIA_C [C1]** | **1** | **Q14145** | **Q14145_C288** | **347.8** | **436.3** | **266** | **171.8** | **187.8** | **145.5** | **2.024447** | **0.023097** |
| **[K].TTC*MSSQGSDDEQIK.[R]** | **1xDBIA_C [C3]** | **3** | **Q9P0V9** | **Q9P0V9_C22** | **824.7** | **921** | **595.7** | **439.3** | **443.3** | **249.9** | **2.0775998** | **0.025265** |
| **[K].C*NFTGDGK.[T]** | **1xDBIA_C [C1]** | **2** | **Q7Z6K5** | **Q7Z6K5_C183** | **1289** | **1665.2** | **943.6** | **585.7** | **621.8** | **405.1** | **2.3293014** | **0.025352** |
| **[R].C*SILNYLK.[L]** | **1xDBIA_C [C1]** | **1** | **P48506** | **P48506_C553** | **1642.6** | **1969.8** | **1179.3** | **821.8** | **829.1** | **516.6** | **2.2828107** | **0.025358** |
| **[K].C*ASQVGMTAPGTR.[R]** | **1xDBIA_C [C1]** | **4** | **Q99439** | **Q99439_C215** | **1012.7** | **1188.2** | **751.6** | **566.6** | **567.1** | **365.1** | **2.0586141** | **0.027908** |
| **[R].C*SGPNRPQNSWR.[T]** | **1xDBIA_C [C1]** | **1** | **Q2TAZ0** | **Q2TAZ0_C1458** | **260.4** | **345.9** | **195** | **130.9** | **131.4** | **92.4** | **2.1103896** | **0.030861** |
| **[K].NSNQLGGNTESSESSETC*SSK.[S]** | **1xDBIA_C [C18]** | **1** | **Q9H9A5** | **Q9H9A5_C504** | **210.4** | **246.1** | **139** | **97** | **99.8** | **44.6** | **2.4659319** | **0.031176** |
| **[K].C*SSSASSK.[Q]** | **1xDBIA_C [C1]** | **1** | **Q9Y2K1** | **Q9Y2K1_C126** | **192.7** | **251.3** | **161.3** | **112.2** | **125.2** | **73.4** | **2.0071885** | **0.032718** |
| **[K].C*TGGEESK.[A]** | **1xDBIA_C [C1]** | **1** | **Q96GV9** | **Q96GV9_C77** | **669.3** | **837.2** | **465.1** | **315.2** | **337.2** | **173.5** | **2.4827995** | **0.032771** |
| **[K].NALGC*ENK.[G]** | **1xDBIA_C [C5]** | **1** | **Q8N954** | **Q8N954_C95** | **573.4** | **715.7** | **441.2** | **329** | **344.2** | **217.7** | **2.0266422** | **0.034396** |
| **[RK].TSC*SPQK.[AT]** | **1xDBIA_C [C3]** | **1** | **Q8IV63** | **Q8IV63_C128** | **699.2** | **809.1** | **491.7** | **403.4** | **392.8** | **213.5** | **2.0057015** | **0.041647** |
| **[RK].TSC*SPQK.[AT]** | **1xDBIA_C [C3]** | **1** | **Q8IY33** | **Q8IY33_C271** | **699.2** | **809.1** | **491.7** | **403.4** | **392.8** | **213.5** | **2.0057015** | **0.041647** |
| **[R].C*SDNTEVEVSNLENK.[Q]** | **1xDBIA_C [C1]** | **1** | **Q9NQW6** | **Q9NQW6_C71** | **244.9** | **287.3** | **169.6** | **134.1** | **137.8** | **70.9** | **2.0849057** | **0.042355** |
| **[K].TSSNC*NGEEK.[K]** | **1xDBIA_C [C5]** | **1** | **P46100** | **P46100_C324** | **369.2** | **491** | **294.5** | **222.9** | **226.2** | **103.5** | **2.1706454** | **0.045712** |
| **[K].NASC*GTR.[S]** | **1xDBIA_C [C4]** | **2** | **P60468** | **P60468_C39** | **279.4** | **394** | **209.5** | **148.5** | **152.5** | **96.8** | **2.1642562** | **0.046289** |
| **[K].C*YQEDSSEK.[A]** | **1xDBIA_C [C1]** | **1** | **Q9Y2K7** | **Q9Y2K7_C675** | **330.8** | **482.1** | **289.3** | **209.2** | **211** | **117.3** | **2.2848341** | **0.046818** |
| **[K].NEMNC*K.[E]** | **1xDBIA_C [C5]** | **2** | **P53618** | **P53618_C684** | **1909.8** | **2303.1** | **1382** | **1144.3** | **1136.7** | **644.8** | **2.0126715** | **0.047122** |
| **[R].NSC*NVGGGGGGFK.[H]** | **1xDBIA_C [C3]** | **1** | **Q7L2J0** | **Q7L2J0_C153** | **775.8** | **1028.8** | **544.6** | **412.4** | **419.4** | **220.2** | **2.4530281** | **0.0487** |
| **[R].NC*SEEK.[S]** | **1xDBIA_C [C2]** | **1** | **Q8ND24** | **Q8ND24_C149** | **868.6** | **1093.9** | **614.2** | **501.3** | **486** | **269.3** | **2.1821265** | **0.049133** |

**Supplemental Table 2 Candidate covalent protein targets and labeled peptides of PTL identified by MTRP in RAW264.7 cells**

| **Master Protein Accessions** | **Annotated Sequence** | **Modifications** | **Modifications in Master Proteins** | **# PSMs** | **Abundance: DMSO** | | | **Abundance: PTL** | | | **Abundance Ratio: (DMSO) / (PTL)** | **Abundance Ratio P-Value: (DMSO) / (PTL)** |
| --- | --- | --- | --- | --- | --- | --- | --- | --- | --- | --- | --- | --- |
|  |  |  |  |  | **1** | **2** | **3** | **1** | **2** | **3** |  |  |
| **Q6PIP5** | **[R].DPAQC*AAIAER.[L]** | **1xDBIA [C5(100)]** | **Q6PIP5_C375** | **2** | **271.1** | **276.2** | **268.4** | **47.1** | **52.9** | **58.1** | **5.124763705** | **6.08938E-07** |
| **Q8BH58** | **[K].VAC*AEEWQESR.[T]** | **1xDBIA [C3(100)]** | **Q8BH58_C87** | **3** | **1321.8** | **1366.3** | **1353.5** | **230.9** | **283.2** | **275.8** | **4.907541697** | **8.46927E-07** |
| **Q8BKZ9** | **[K].DVSAPPPVSKPPAPTQPSPQPQIPC*PAR.[K]** | **1xDBIA [C25(100)]** | **Q8BKZ9_C170** | **1** | **216** | **228.8** | **216.9** | **28.7** | **31.2** | **27.4** | **7.557491289** | **1.48332E-06** |
| **Q8K4Q0** | **[R].DSPC*TPR.[L]** | **1xDBIA [C4(100)]** | **Q8K4Q0_C713** | **1** | **157.5** | **161.2** | **151.6** | **26.4** | **32.2** | **31.4** | **5.015923567** | **2.85356E-06** |
| **Q8C9B9** | **[R].REEDSAETGSVQIGSAEQDRPLC*K.[Q]** | **1xDBIA [C23(100)]** | **Q8C9B9_C212** | **1** | **144.9** | **147.4** | **156.7** | **31.1** | **30.2** | **32.4** | **4.798013245** | **5.35709E-06** |
| **Q99P88** | **[R].YVENPSLVLNC*ER.[R]** | **1xDBIA [C11(100)]** | **Q99P88_C1344** | **1** | **107.8** | **114.3** | **110.6** | **24.2** | **28.3** | **20.4** | **4.570247934** | **8.09833E-06** |
| **Q8CG48** | **[K].KLDC*AK.[T]** | **1xDBIA [C4(100)]** | **Q8CG48_C800** | **3** | **2293.9** | **2430.7** | **2525.7** | **499.1** | **525.1** | **531.9** | **4.629023043** | **9.80875E-06** |
| **Q60953** | **[K].ALC*SLR.[Q]** | **1xDBIA [C3(100)]** | **Q60953_C361** | **1** | **822.5** | **822.4** | **906.4** | **105.8** | **121.6** | **103.3** | **7.774102079** | **1.3174E-05** |
| **A2AWP8** | **[R].SRPC*AR.[D]** | **1xDBIA [C4(100)]** | **A2AWP8_C1226** | **1** | **125.4** | **132.1** | **137.6** | **27.8** | **24.2** | **32.3** | **4.751798561** | **1.65658E-05** |
| **Q9D1H7** | **[K].EQNYC*ESR.[Y]** | **1xDBIA [C5(100)]** | **Q9D1H7_C160** | **1** | **211.3** | **237.7** | **232.8** | **51.6** | **56.1** | **58.7** | **4.09496124** | **3.3255E-05** |
| **Q5SFM8** | **[K].LC*SGSK.[S]** | **1xDBIA [C2(100)]** | **Q5SFM8_C802** | **1** | **1354.3** | **1326.2** | **1193.1** | **320.6** | **308.3** | **297.3** | **4.224266999** | **4.00988E-05** |
| **Q80WC7** | **[R].ELGGC*SQAGNR.[H]** | **1xDBIA [C5(100)]** | **Q80WC7_C39** | **2** | **393.3** | **409.7** | **462.4** | **16.1** | **16.7** | **19.2** | **24.42857143** | **4.1758E-05** |
| **Q8CB96** | **[R].ILHGPC*EK.[I]** | **1xDBIA [C6(100)]** | **Q8CB96_C250** | **1** | **375.9** | **375.5** | **423.2** | **85.3** | **87** | **82.6** | **4.546004843** | **4.24933E-05** |
| **Q8BQM4** | **[K].RPQFSPIESC*QAEAAAASNGTGDEEDDGPAAELLEK.[L]** | **1xDBIA [C10(100)]** | **Q8BQM4_C20** | **1** | **82.7** | **92.4** | **82.2** | **17.4** | **21** | **17.6** | **4.698863636** | **4.4583E-05** |
| **P35601** | **[K].EASTC*PR.[G]** | **1xDBIA [C5(100)]** | **P35601_C547** | **2** | **490** | **544.1** | **573.5** | **73.5** | **69.3** | **76.7** | **7.402721088** | **4.66495E-05** |
| **B2RX14** | **[R].DQSHC*K.[M]** | **1xDBIA [C5(100)]** | **B2RX14_C94** | **1** | **437.7** | **480.5** | **418.7** | **105.7** | **109.3** | **114.5** | **4.004574565** | **5.36344E-05** |
| **Q3UHD6** | **[R].VFC*ELK.[W]** | **1xDBIA [C3(100)]** | **Q3UHD6_C517** | **3** | **998.1** | **1006.4** | **869.3** | **156.6** | **164.9** | **154.9** | **6.103092784** | **5.64619E-05** |
| **Q99P31** | **[R].DSC*DTVR.[V]** | **1xDBIA [C3(100)]** | **Q99P31_C199** | **2** | **569.9** | **586.2** | **500.2** | **78.1** | **87.4** | **84.9** | **6.707093822** | **6.02339E-05** |
| **Q8BVE8** | **[K].ESC*PNTGR.[D]** | **1xDBIA [C3(100)]** | **Q8BVE8_C207** | **2** | **308.9** | **361.9** | **354.3** | **61.4** | **63.8** | **67.1** | **5.393442623** | **7.54321E-05** |
| **Q8K1E6** | **[K].EQQQC*DR.[Q]** | **1xDBIA [C5(100)]** | **Q8K1E6_C50** | **1** | **155** | **177.1** | **184.6** | **20.3** | **19.5** | **27.5** | **7.948717949** | **8.47494E-05** |
| **Q9QWT9** | **[K].GQLC*DLNEELK.[R]** | **1xDBIA [C4(100)]** | **Q9QWT9_C145** | **1** | **458.3** | **465.5** | **530** | **103.7** | **101.3** | **116.6** | **4.524185587** | **8.47791E-05** |
| **Q9DB85** | **[K].LQPC*LYK.[R]** | **1xDBIA [C4(100)]** | **Q9DB85_C452** | **1** | **444.7** | **506.6** | **527.5** | **106.2** | **113.6** | **114.9** | **4.459507042** | **0.000107817** |
| **Q920Q8** | **[K].QISGSSTGC*LSSPNASMQSPK.[H]** | **1xDBIA [C9(100)]** | **Q920Q8_C274** | **1** | **299.9** | **317** | **260.7** | **33.7** | **47** | **48** | **6.604166667** | **0.000133776** |
| **Q7TSG2** | **[R].AASGGC*VR.[A]** | **1xDBIA [C6(100)]** | **Q7TSG2_C73** | **2** | **490.3** | **501.7** | **573.9** | **119.7** | **153.7** | **116.5** | **4.191311612** | **0.000167632** |
| **Q9QZN4** | **[K].AEGYC*EELMDTFRPDIVVK.[D]** | **1xDBIA [C5(100)]** | **Q9QZN4_C145** | **1** | **248.3** | **250** | **302.4** | **26.4** | **34.5** | **33.5** | **8.765217391** | **0.000194643** |
| **Q810U5** | **[K].HDPEC*K.[L]** | **1xDBIA [C5(100)]** | **Q810U5_C237** | **4** | **1414.5** | **1336.1** | **1133.9** | **215.4** | **217.8** | **219.2** | **6.134527089** | **0.000209031** |
| **Q8K387** | **[K].GC*TVPSVK.[G]** | **1xDBIA [C2(100)]** | **Q8K387_C185** | **3** | **1247.1** | **1214.8** | **1481.5** | **263.3** | **292.4** | **268.5** | **4.644692737** | **0.000250752** |
| **Q3UHX0** | **[K].DSETNGPQNDSHC*K.[F]** | **1xDBIA [C13(100)]** | **Q3UHX0_C494** | **1** | **148** | **169.9** | **135.4** | **23.4** | **27.7** | **28.8** | **5.786324786** | **0.000260195** |
| **Q8BZA9** | **[K].AAGEEC*PMFTPPGGETVEQVK.[M]** | **1xDBIA [C6(100)]** | **Q8BZA9_C114** | **1** | **231.1** | **250.5** | **291.3** | **42.2** | **43.8** | **46.6** | **5.719178082** | **0.000276483** |
| **Q6VN19** | **[R].QLC*GGNQAATER.[I]** | **1xDBIA [C3(100)]** | **Q6VN19_C501** | **1** | **143** | **133.7** | **109.4** | **6.5** | **7.6** | **10** | **16.83076923** | **0.000277946** |
| **Q2EMV9** | **[K].ASYSAC*DTYSRPDTNGR.[K]** | **1xDBIA [C6(100)]** | **Q2EMV9_C1740** | **1** | **158.6** | **179.3** | **206.9** | **16** | **20.9** | **17** | **9.9125** | **0.000312414** |
| **Q80YR5** | **[R].ASC*SSGR.[N]** | **1xDBIA [C3(100)]** | **Q80YR5_C447** | **1** | **188.3** | **214.3** | **245.8** | **24.9** | **22.6** | **25** | **8.606425703** | **0.000322609** |
| **E9Q634** | **[R].AAPAPPGC*HQNGVIR.[N]** | **1xDBIA [C8(100)]** | **E9Q634_C960** | **1** | **115.2** | **130.4** | **145.5** | **31.3** | **29.1** | **26.9** | **4.481099656** | **0.000331193** |
| **Q61093** | **[R].GSSACC*STR.[I]** | **1xDBIA [C6(100)]** | **Q61093_C86** | **1** | **51.2** | **60.9** | **64.2** | **11.9** | **11.9** | **15.2** | **4.302521008** | **0.000350679** |
| **Q9CYU6** | **[K].DC*SSSSVK.[T]** | **1xDBIA [C2(100)]** | **Q9CYU6_C417** | **1** | **141.4** | **144.5** | **114.4** | **27.4** | **29.3** | **31.9** | **4.529780564** | **0.000421444** |
| **Q91WU5** | **[R].HAPEGTGGCC*GK.[R]** | **1xDBIA [C10(100)]** | **Q91WU5_C370** | **3** | **761.2** | **676.8** | **604.7** | **124.2** | **191.2** | **121** | **4.997520661** | **0.000454293** |
| **Q8K1R7** | **[R].LDPAVPC*VGK.[A]** | **1xDBIA [C7(100)]** | **Q8K1R7_C885** | **1** | **269.7** | **303.8** | **348.9** | **68.5** | **73.2** | **70** | **4.34** | **0.000499347** |
| **Q9D1J1** | **[K].QQC*EFAK.[Q]** | **1xDBIA [C3(100)]** | **Q9D1J1_C133** | **2** | **1253.2** | **1110** | **965.2** | **256.3** | **301.8** | **260.8** | **4.15241882** | **0.000581481** |
| **Q9QXL8** | **[R].AC*QQGR.[S]** | **1xDBIA [C2(100)]** | **Q9QXL8_C4** | **2** | **304.2** | **290.6** | **376.5** | **60.4** | **62.3** | **54.6** | **5.322344322** | **0.000586306** |
| **Q64701** | **[R].LC*QENDDVLLK.[R]** | **1xDBIA [C2(100)]** | **Q64701_C1042** | **1** | **631.4** | **557.8** | **481.6** | **132.7** | **138.1** | **146** | **4.0391021** | **0.000650891** |
| **Q6P549** | **[R].KPASTETSC*PLSK.[L]** | **1xDBIA [C9(100)]** | **Q6P549_C927** | **1** | **507.5** | **559.9** | **682.8** | **88.6** | **96.6** | **93.1** | **6.01396348** | **0.000703681** |
| **E9Q2M9** | **[R].QIC*LDGAQDPSRPAGSQTSGK.[A]** | **1xDBIA [C3(100)]** | **E9Q2M9_C1562** | **1** | **377.3** | **404** | **298.5** | **50.6** | **62.2** | **66.3** | **6.065916399** | **0.000720373** |
| **Q80XQ2** | **[R].APAC*SPLLFSDPLMGPASASASSSNPSSSPDDDSSK.[E]** | **1xDBIA [C4(100)]** | **Q80XQ2_C771** | **1** | **77.7** | **100.4** | **101.3** | **19.5** | **24.2** | **25.2** | **4.01984127** | **0.000895759** |
| **P17225** | **[R].GSDELFSTC*VSNGPFIMSSSASAANGNDSK.[K]** | **1xDBIA [C9(100)]** | **P17225_C23** | **1** | **77.9** | **87.7** | **107.4** | **10** | **14.6** | **14.4** | **7.356164384** | **0.000896047** |
| **Q9JHU9** | **[K].EPTPATNGC*TGDANGHPQAPTPK.[L]** | **1xDBIA [C9(100)]** | **Q9JHU9_C539** | **2** | **338.4** | **403.2** | **460.3** | **67.9** | **85.5** | **99.4** | **4.715789474** | **0.00096202** |
| **P61759** | **[K].DGC*GLETAAGNGR.[R]** | **1xDBIA [C3(100)]** | **P61759_C8** | **1** | **192.6** | **177.7** | **141.7** | **29.4** | **36.5** | **42.3** | **4.819727891** | **0.000982903** |
| **Q61510** | **[K].QQC*VQDSMK.[R]** | **1xDBIA [C3(100)]** | **Q61510_C228** | **3** | **568.3** | **502.5** | **702.9** | **97.9** | **95.1** | **107.7** | **5.804902962** | **0.001146403** |
| **O70252** | **[K].C*PFYAAQPDK.[G]** | **1xDBIA [C1(100)]** | **O70252_C264** | **2** | **781.4** | **758.3** | **581.8** | **171.1** | **191.7** | **181.1** | **4.076160668** | **0.001147835** |
| **Q6P9L6** | **[-].MAPGC*K.[S]** | **1xDBIA [C5(100)]** | **Q6P9L6_C5** | **1** | **569.1** | **668.3** | **817.6** | **117.1** | **134** | **128.5** | **5.20077821** | **0.001519714** |
| **Q8K4P0** | **[R].GGQDC*R.[G]** | **1xDBIA [C5(100)]** | **Q8K4P0_C990** | **2** | **502.9** | **545.6** | **754.6** | **91.9** | **99.5** | **102.1** | **5.483417085** | **0.002947364** |
| **Q69ZS7** | **[R].AVC*AGQPSK.[G]** | **1xDBIA [C3(100)]** | **Q69ZS7_C133** | **2** | **260.1** | **292** | **393.5** | **52.6** | **60.9** | **60.2** | **4.94486692** | **0.003090087** |
| **Q3UJP5** | **[K].SSSNTC*VR.[A]** | **1xDBIA [C6(100)]** | **Q3UJP5_C94** | **1** | **230.8** | **203.6** | **137.8** | **16.2** | **19.5** | **21.7** | **10.44102564** | **0.003430633** |
| **Q8N9S3** | **[K].GVPVC*K.[E]** | **1xDBIA [C5(100)]** | **Q8N9S3_C303** | **1** | **344.7** | **439** | **532.3** | **109.4** | **102.9** | **104.7** | **4.192932187** | **0.00355656** |
| **Q3TWF6** | **[R].SQSSC*R.[D]** | **1xDBIA [C5(100)]** | **Q3TWF6_C98** | **2** | **136** | **132.7** | **88.7** | **27** | **33.8** | **33.1** | **4.009063444** | **0.004669909** |
| **Q9Z148** | **[K].GGAC*PSR.[A]** | **1xDBIA [C4(100)]** | **Q9Z148_C179** | **1** | **122.1** | **130.6** | **196.7** | **21.9** | **21.8** | **19** | **6.426315789** | **0.005461156** |
| **O88738** | **[R].HHVPQHC*NK.[M]** | **1xDBIA [C7(100)]** | **O88738_C3696** | **2** | **322** | **248.5** | **181.6** | **35.1** | **35** | **37.7** | **7.07977208** | **0.006104696** |
| **Q8BH15** | **[K].SSSQLGGNTESSESSETC*SSK.[S]** | **1xDBIA [C18(100)]** | **Q8BH15_C504** | **1** | **108.7** | **78** | **58.5** | **8.7** | **6.8** | **8.1** | **9.62962963** | **0.007222177** |
| **P53569** | **[K].TVAEQRPESC*PVSK.[A]** | **1xDBIA [C10(100)]** | **P53569_C153** | **2** | **961.6** | **773.7** | **556.1** | **171.3** | **200.3** | **181.5** | **4.262809917** | **0.007858142** |
| **Q8K305** | **[K].APDC*AQR.[K]** | **1xDBIA [C4(100)]** | **Q8K305_C264** | **2** | **263.8** | **306.5** | **458.6** | **70.8** | **70.3** | **76.7** | **4.329096045** | **0.01025513** |
| **P13864** | **[K].SQLC*DLETK.[L]** | **1xDBIA [C4(100)]** | **P13864_C62** | **3** | **1255.8** | **1068.6** | **670.7** | **213.4** | **262.3** | **238.5** | **4.480503145** | **0.011760131** |
| **P97329** | **[K].AAC*IAEQYHTVLK.[L]** | **1xDBIA [C3(100)]** | **P97329_C807** | **1** | **583** | **679.7** | **1056.1** | **132.5** | **172** | **142.5** | **4.769824561** | **0.01255523** |
| **Q9CYN2** | **[R].SGGGGGSSGAGGGPSC*GTSSSR.[S]** | **1xDBIA [C16(100)]** | **Q9CYN2_C26** | **4** | **230.7** | **164.4** | **121.8** | **33.2** | **39.9** | **42.9** | **4.120300752** | **0.013689876** |
| **G5E870** | **[K].TTGSC*ASTSR.[R]** | **1xDBIA [C5(100)]** | **G5E870_C332** | **1** | **120.5** | **93.8** | **58** | **18** | **20** | **20.8** | **4.69** | **0.017154292** |
| **Q61263** | **[K].SASLDNGGC*ALTTFSILEEMK.[K]** | **1xDBIA [C9(100)]** | **Q61263_C82** | **3** | **677.5** | **596.2** | **329.1** | **102.5** | **138.9** | **117.8** | **4.877609791** | **0.017254804** |
| **P25799** | **[R].YVC*EGPSHGGLPGASSEK.[N]** | **1xDBIA [C3(100)]** | **P25799_C59** | **1** | **390** | **691** | **808.5** | **142.9** | **177** | **146.7** | **4.56779661** | **0.019240018** |
| **Q7TMB8** | **[K].C*NEQPNR.[V]** | **1xDBIA [C1(100)]** | **Q7TMB8_C98** | **2** | **289** | **201.6** | **116.5** | **15.5** | **17.1** | **13.2** | **13.00645161** | **0.019839874** |
| **Q6Q899** | **[K].C*VLEAFR.[A]** | **1xDBIA [C1(100)]** | **Q6Q899_C681** | **4** | **1643.1** | **1172.2** | **787.7** | **247.5** | **302.2** | **263.5** | **4.44857685** | **0.019921538** |
| **Q8C156** | **[K].QDATSC*TEHK.[K]** | **1xDBIA [C6(100)]** | **Q8C156_C451** | **2** | **349.6** | **605** | **755.9** | **123.1** | **123.1** | **143.3** | **4.914703493** | **0.020697824** |
| **A2BE28** | **[K].C*SNLPLK.[Y]** | **1xDBIA [C1(100)]** | **A2BE28_C118** | **1** | **717.7** | **439.2** | **330.8** | **69.9** | **82.4** | **77.2** | **5.689119171** | **0.022006116** |
| **Q9CU65** | **[K].GPENLHYDQGC*QTSR.[T]** | **1xDBIA [C11(100)]** | **Q9CU65_C822** | **1** | **110.2** | **141.9** | **233.7** | **35.3** | **32.9** | **38.9** | **4.019830028** | **0.027151777** |
| **Q9D2L9** | **[K].C*QENFQAR.[E]** | **1xDBIA [C1(100)]** | **Q9D2L9_C495** | **1** | **333.2** | **220.8** | **141.4** | **35.1** | **50.3** | **43.5** | **5.075862069** | **0.027701498** |
| **Q8BY02** | **[K].SSQC*HSGSSPK.[G]** | **1xDBIA [C4(100)]** | **Q8BY02_C424** | **1** | **152.1** | **307.1** | **340.9** | **76.8** | **69.6** | **76.7** | **4.003911343** | **0.029760287** |
| **Q8K298** | **[K].QNTC*SSTTHLAQQLK.[Q]** | **1xDBIA [C4(100)]** | **Q8K298_C412** | **1** | **125.8** | **269.7** | **305.5** | **49.8** | **48.4** | **59.5** | **5.134453782** | **0.030181195** |
| **O88351** | **[R].NNSC*LSK.[M]** | **1xDBIA [C4(100)]** | **O88351_C464** | **1** | **593.9** | **372.3** | **233.4** | **70.3** | **86.4** | **82.4** | **4.518203883** | **0.038152609** |
| **Q60710** | **[K].TSSC*LQEVSK.[V]** | **1xDBIA [C4(100)]** | **Q60710_C645** | **2** | **900.5** | **620** | **365.4** | **139.7** | **188.1** | **152.4** | **4.06824147** | **0.039203358** |

**Supplemental Table 3 Mass Spectrometry-Based Analysis of Covalent Modification: Identification and Quantification of Modification Sites on Truncated HSPBP1 by PTL (Cys201 Shown as the Primary Site)**

| **Annotated Sequence** | **Modifications** | **# PSMs** | **Master Protein Accessions** | **Positions in Master Proteins** | **Modifications in Master Proteins** | **Sequence Length** | **Abundance: F2: Sample, P** | **Charge (by Search Engine): Sequest HT** | **RT [min] (by Search Engine): Sequest HT** |
| --- | --- | --- | --- | --- | --- | --- | --- | --- | --- |
| **[R].EGALELLADLCENMDNAADFCQLSGMHLLVGR.[Y]** | **2xSHM-P [C11; C21]** | **1** | **Q9NZL4** | **Q9NZL4 [121-152]** | **Q9NZL4 2xSHM-P [C131; C141]** | **32** |  | **3** | **58.368** |
| **[R].LLDRDACDTVR.[V]** | **1xSHM-P [C7]** | **11** | **Q9NZL4** | **Q9NZL4 [195-205]** | **Q9NZL4 1xSHM-P [C201]** | **11** | **3.937E+09** | **3** | **35.078** |
| **[K].LLQTCFSSPADDSMDR.[-]** | **1xSHM-P [C5]** | **4** | **Q9NZL4** | **Q9NZL4 [344-359]** | **Q9NZL4 1xSHM-P [C348]** | **16** | **156865838** | **2** | **56.1174** |
| **[K].GTLCSMGMVQQLVALVR.[T]** | **1xSHM-P [C4]** | **1** | **Q9NZL4** | **Q9NZL4 [266-282]** | **Q9NZL4 1xSHM-P [C269]** | **17** |  | **2** | **57.9352** |
| **[R].ECREPELGLEELLR.[H]** | **1xSHM-P [C2]** | **3** | **Q9NZL4** | **Q9NZL4 [309-322]** | **Q9NZL4 1xSHM-P [C310]** | **14** | **35686928** | **3** | **57.0157** |
| **[R].EGALELLADLCENMDNAADFCQLSGMHLLVGR.[Y]** | **1xSHM-P [C21]** | **1** | **Q9NZL4** | **Q9NZL4 [121-152]** | **Q9NZL4 1xSHM-P [C141]** | **32** |  | **3** | **58.2404** |
| **[R].CQLLQQHEEYQEELEFCEK.[L]** | **1xSHM-P [C1]** | **1** | **Q9NZL4** | **Q9NZL4 [325-343]** | **Q9NZL4 1xSHM-P [C325]** | **19** | **11366058** | **3** | **54.9112** |
| **[R].TEHSPFHEHVLGALCSLVTDFPQGVR.[E]** | **1xSHM-P [C15]** | **1** | **Q9NZL4** | **Q9NZL4 [283-308]** | **Q9NZL4 1xSHM-P [C297]** | **26** |  | **3** | **57.7791** |
| **[K].LLRLLDRDACDTVR.[V]** | **1xSHM-P [C10]** | **1** | **Q9NZL4** | **Q9NZL4 [192-205]** | **Q9NZL4 1xSHM-P [C201]** | **14** | **2958650** | **3** | **53.4853** |
| **[R].AAQLIGTCSQNVAAIQEQVLGLGALRK.[L]** |  | **1** | **Q9NZL4** | **Q9NZL4 [165-191]** |  | **27** | **4781032** | **3** | **55.8999** |
| **[K].ALFAISCLVR.[E]** |  | **4** | **Q9NZL4** | **Q9NZL4 [208-217]** |  | **10** | **1E+08** | **2** | **55.4385** |
| **[R].AMQQQVQK.[L]** |  | **18** | **Q9NZL4** | **Q9NZL4 [238-245]** |  | **8** | **4.05E+09** | **2** | **10.7608** |
| **[R].AMQQQVQKLK.[V]** |  | **1** | **Q9NZL4** | **Q9NZL4 [238-247]** |  | **10** | **14625193** | **3** | **20.6684** |
| **[R].DACDTVR.[V]** |  | **5** | **Q9NZL4** | **Q9NZL4 [199-205]** |  | **7** | **33215562** | **2** | **12.58** |
| **[R].ECREPELGLEELLR.[H]** |  | **2** | **Q9NZL4** | **Q9NZL4 [309-322]** |  | **14** | **2222490** | **3** | **53.2688** |
| **[R].EPELGLEELLR.[H]** |  | **11** | **Q9NZL4** | **Q9NZL4 [312-322]** |  | **11** | **1.92E+09** | **2** | **55.5673** |
| **[R].EQEAGLLQFLR.[L]** |  | **10** | **Q9NZL4** | **Q9NZL4 [218-228]** |  | **11** | **3.68E+09** | **2** | **54.5884** |
| **[R].LDGFSVLMR.[A]** |  | **20** | **Q9NZL4** | **Q9NZL4 [229-237]** |  | **9** | **3.19E+09** | **2** | **51.0592** |
| **[K].LKVKSAFLLQNLLVGHPEHK.[G]** |  | **1** | **Q9NZL4** | **Q9NZL4 [246-265]** |  | **20** | **2598345** | **4** | **48.2961** |
| **[R].LLDRDACDTVR.[V]** |  | **42** | **Q9NZL4** | **Q9NZL4 [195-205]** |  | **11** | **7.08E+08** | **3** | **29.9678** |
| **[K].LLQTCFSSPADDSMDR.[-]** |  | **5** | **Q9NZL4** | **Q9NZL4 [344-359]** |  | **16** | **3.03E+08** | **2** | **45.146** |
| **[K].SAFLLQNLLVGHPEHK.[G]** |  | **57** | **Q9NZL4** | **Q9NZL4 [250-265]** |  | **16** | **8.38E+09** | **2** | **51.4871** |
| **[R].TEHSPFHEHVLGALCSLVTDFPQGVR.[E]** |  | **5** | **Q9NZL4** | **Q9NZL4 [283-308]** |  | **26** | **21759623** | **4** | **55.4563** |
| **[K].VKSAFLLQNLLVGHPEHK.[G]** |  | **2** | **Q9NZL4** | **Q9NZL4 [248-265]** |  | **18** | **20176322** | **3** | **48.5133** |
| **[R].VLSQPMPPTAGEAEQAADQQER.[E]** |  | **70** | **Q9NZL4** | **Q9NZL4 [99-120]** |  | **22** | **5.58E+09** | **3** | **39.5565** |
| **[R].YLEAGAAGLR.[W]** |  | **128** | **Q9NZL4** | **Q9NZL4 [153-162]** |  | **10** | **4.87E+09** | **2** | **33.6898** |
| **[R].YLEAGAAGLRWR.[A]** |  | **2** | **Q9NZL4** | **Q9NZL4 [153-164]** |  | **12** | **70938064** | **3** | **41.9597** |

**Supplemental Table 4 Mass spectrometry profile of proteins enriched by PTL-6 in situ**

| **Accession** | **Coverage [%]** | **# Peptides** | **# PSMs** | **# Unique Peptides** | **# AAs** | **MW [kDa]** | **calc. pI** | **Score Sequest HT: Sequest HT** | **Abundances (Normalized): F1: Sample** |
| --- | --- | --- | --- | --- | --- | --- | --- | --- | --- |
| **P63104** | **3** | **1** | **1** | **1** | **245** | **27.7** | **4.79** | **1.75** | **7905327** |
| **Q15233** | **6** | **3** | **3** | **3** | **471** | **54.2** | **8.95** | **5.97** | **7538172.25** |
| **Q96L21** | **9** | **2** | **2** | **2** | **214** | **24.5** | **10.01** | **2.78** | **5567507.5** |
| **Q96P63** | **31** | **11** | **12** | **11** | **405** | **46.2** | **5.53** | **41.67** | **132075783.8** |
| **P19224** | **3** | **2** | **2** | **1** | **532** | **60.7** | **8.41** | **2.55** | **5088613** |
| **P00558** | **7** | **3** | **3** | **3** | **417** | **44.6** | **8.1** | **7.1** | **10328508.5** |
| **P07437** | **18** | **7** | **9** | **2** | **444** | **49.6** | **4.89** | **20.86** | **92673174.75** |
| **Q15366** | **9** | **2** | **2** | **1** | **365** | **38.6** | **6.79** | **6.7** | **2228903.5** |
| **P06733** | **49** | **15** | **18** | **15** | **434** | **47.1** | **7.39** | **51.75** | **92997767.5** |
| **P61978** | **13** | **4** | **4** | **4** | **463** | **50.9** | **5.54** | **11.41** | **2900614.75** |
| **P57088** | **3** | **1** | **1** | **1** | **247** | **28** | **9.7** | **0** | **1749762** |
| **Q14534** | **15** | **5** | **5** | **5** | **574** | **63.9** | **8.63** | **14.78** | **13439335.25** |
| **P23526** | **2** | **1** | **1** | **1** | **432** | **47.7** | **6.34** | **2.41** | **1766320.5** |
| **P08238** | **6** | **4** | **4** | **4** | **724** | **83.2** | **5.03** | **11.92** | **39341454** |
| **O75369** | **2** | **5** | **5** | **5** | **2602** | **278** | **5.73** | **5.62** | **12912472** |
| **Q8N1N4** | **31** | **14** | **17** | **12** | **520** | **56.8** | **6.02** | **45.13** | **85686366.25** |
| **O43175** | **4** | **2** | **2** | **2** | **533** | **56.6** | **6.71** | **4.57** | **6897340.75** |
| **P45880** | **32** | **8** | **9** | **8** | **294** | **31.5** | **7.56** | **29.45** | **74094561.5** |
| **Q06830** | **30** | **6** | **9** | **5** | **199** | **22.1** | **8.13** | **23.53** | **85525522.5** |
| **P02100** | **7** | **1** | **1** | **1** | **147** | **16.2** | **8.63** | **2.45** | **10904528** |
| **P62987** | **30** | **3** | **7** | **3** | **128** | **14.7** | **9.83** | **19.62** | **178876726.3** |
| **P78527** | **2** | **9** | **9** | **9** | **4128** | **468.8** | **7.12** | **18.85** | **20032126.63** |
| **P55060** | **2** | **2** | **2** | **2** | **971** | **110.3** | **5.77** | **4.08** | **7673405** |
| **Q04695** | **61** | **32** | **49** | **16** | **432** | **48.1** | **5.02** | **159.16** | **391601058.3** |
| **Q15758** | **4** | **2** | **2** | **2** | **541** | **56.6** | **5.48** | **4.45** | **4036015.75** |
| **Q15365** | **28** | **6** | **6** | **5** | **356** | **37.5** | **7.09** | **19.7** | **103622999.5** |
| **P14618** | **34** | **11** | **12** | **11** | **531** | **57.9** | **7.84** | **33.88** | **72580836.88** |
| **P05783** | **23** | **8** | **8** | **7** | **430** | **48** | **5.45** | **21.57** | **40748916.75** |
| **P11413** | **7** | **4** | **4** | **4** | **515** | **59.2** | **6.84** | **7.01** | **7577555.75** |
| **P04843** | **3** | **1** | **1** | **1** | **607** | **68.5** | **6.38** | **2.04** | **1171263.5** |
| **P30740** | **11** | **4** | **4** | **4** | **379** | **42.7** | **6.28** | **9.36** | **7792373.25** |
| **Q9HAW8** | **4** | **2** | **2** | **1** | **530** | **59.8** | **7.3** | **5.21** | **2632790.25** |
| **P36578** | **3** | **1** | **1** | **1** | **427** | **47.7** | **11.06** | **3.11** |  |
| **O60218** | **7** | **2** | **2** | **2** | **316** | **36** | **7.84** | **2.19** | **5427779.875** |
| **P26641** | **2** | **1** | **1** | **1** | **437** | **50.1** | **6.67** | **1.99** | **2830049.25** |
| **O95197** | **2** | **2** | **2** | **2** | **1032** | **112.5** | **4.96** | **5.53** | **34278787** |
| **P38646** | **1** | **1** | **1** | **1** | **679** | **73.6** | **6.16** | **2.06** | **9229196** |
| **P61204** | **6** | **1** | **1** | **1** | **181** | **20.6** | **7.43** | **1.95** | **4156715.5** |
| **Q92945** | **3** | **2** | **2** | **2** | **711** | **73.1** | **7.3** | **5.33** | **5476160.5** |
| **P38606** | **2** | **1** | **1** | **1** | **617** | **68.3** | **5.52** | **1.99** |  |
| **P46977** | **1** | **1** | **1** | **1** | **705** | **80.5** | **8.07** | **1.67** |  |
| **P40926** | **3** | **1** | **1** | **1** | **338** | **35.5** | **8.68** | **2.16** | **2784035.75** |
| **P11216** | **16** | **10** | **10** | **10** | **843** | **96.6** | **6.86** | **30.35** | **45097417.75** |
| **P56470** | **19** | **5** | **6** | **5** | **323** | **35.9** | **9.16** | **19.04** | **43045449.5** |
| **P12236** | **9** | **3** | **4** | **3** | **298** | **32.8** | **9.74** | **6.63** | **15226227.5** |
| **P12268** | **2** | **1** | **1** | **1** | **514** | **55.8** | **6.9** | **2.37** | **4088786.5** |
| **P13639** | **16** | **11** | **12** | **11** | **858** | **95.3** | **6.83** | **31.43** | **57349073** |
| **Q00325** | **3** | **1** | **1** | **1** | **362** | **40.1** | **9.38** | **2.2** | **5085575** |
| **P07195** | **5** | **2** | **2** | **2** | **334** | **36.6** | **6.05** | **2.23** | **2285363** |
| **P16615** | **18** | **15** | **16** | **15** | **1042** | **114.7** | **5.34** | **43.8** | **98675959.63** |
| **P50995** | **8** | **3** | **3** | **3** | **505** | **54.4** | **7.65** | **7.3** | **12843509** |
| **P00352** | **11** | **5** | **5** | **5** | **501** | **54.8** | **6.73** | **12.66** | **26281070** |
| **O00410** | **2** | **2** | **2** | **2** | **1097** | **123.6** | **4.94** | **3.16** | **3968509** |
| **P68371** | **15** | **6** | **8** | **1** | **445** | **49.8** | **4.89** | **18.01** | **4357786.5** |
| **Q9NSB4** | **4** | **2** | **2** | **1** | **513** | **56.6** | **6.74** | **4.44** | **218731008** |
| **P04264** | **64** | **57** | **332** | **49** | **644** | **66** | **8.12** | **1188.46** | **80383168902** |
| **P35527** | **91** | **48** | **331** | **47** | **623** | **62** | **5.24** | **1020.73** | **44296454998** |
| **P15924** | **47** | **124** | **165** | **124** | **2871** | **331.6** | **6.81** | **458.96** | **2223144024** |
| **P13645** | **59** | **37** | **141** | **30** | **584** | **58.8** | **5.21** | **437.07** | **17682571615** |
| **P35908** | **85** | **52** | **115** | **41** | **639** | **65.4** | **8** | **363.03** | **5815729686** |
| **P02533** | **72** | **40** | **89** | **16** | **472** | **51.5** | **5.16** | **314.63** | **11090284923** |
| **P02538** | **61** | **45** | **94** | **3** | **564** | **60** | **8** | **288.69** | **3543269295** |
| **P04259** | **60** | **41** | **90** | **2** | **564** | **60** | **8** | **268.99** | **48377758.5** |
| **P48668** | **58** | **42** | **87** | **1** | **564** | **60** | **8** | **264.52** | **86715592.75** |
| **P08779** | **71** | **39** | **77** | **22** | **473** | **51.2** | **5.05** | **263.14** | **1213812627** |
| **P13647** | **56** | **44** | **86** | **27** | **590** | **62.3** | **7.74** | **259.59** | **2608161388** |
| **P14923** | **50** | **30** | **46** | **30** | **745** | **81.7** | **6.14** | **146.87** | **830109253.7** |
| **Q86YZ3** | **21** | **13** | **41** | **13** | **2850** | **282.2** | **10.04** | **141.21** | **891823373.8** |
| **Q02413** | **33** | **25** | **36** | **25** | **1049** | **113.7** | **5.03** | **121.23** | **445749025** |
| **P05787** | **21** | **12** | **39** | **6** | **483** | **53.7** | **5.59** | **105.95** | **34865303** |
| **Q7Z794** | **10** | **6** | **33** | **3** | **578** | **61.9** | **5.99** | **84.24** | **2717735624** |
| **O95678** | **17** | **14** | **25** | **1** | **551** | **59.5** | **7.74** | **75.79** | **18660138** |
| **Q6KB66** | **24** | **10** | **24** | **9** | **452** | **50.5** | **5.67** | **67.33** | **54239071** |
| **Q08554** | **21** | **13** | **19** | **13** | **894** | **99.9** | **5.43** | **62.74** | **174361791.8** |
| **Q01469** | **70** | **12** | **16** | **12** | **135** | **15.2** | **7.01** | **56.42** | **229800158** |
| **P04406** | **36** | **9** | **16** | **9** | **335** | **36** | **8.46** | **47.55** | **143682732.3** |
| **Q13835** | **25** | **14** | **18** | **14** | **747** | **82.8** | **9.13** | **44.47** | **105921859.6** |
| **P60709** | **48** | **12** | **17** | **6** | **375** | **41.7** | **5.48** | **44.31** | **190489684.6** |
| **P13646** | **11** | **7** | **13** | **1** | **458** | **49.6** | **4.96** | **39.06** |  |
| **Q5D862** | **13** | **12** | **16** | **12** | **2391** | **247.9** | **8.31** | **37.29** | **49733870.25** |
| **P19012** | **15** | **9** | **14** | **1** | **456** | **49.2** | **4.77** | **35.2** | **1644230.25** |
| **Q5T749** | **26** | **10** | **12** | **10** | **579** | **64.1** | **8.27** | **32.08** | **107449056** |
| **P07355** | **38** | **11** | **12** | **11** | **339** | **38.6** | **7.75** | **31.05** | **77915239.5** |
| **Q7Z3Y8** | **6** | **3** | **11** | **1** | **459** | **49.8** | **5.05** | **29.4** | **2213486.25** |
| **P68104** | **21** | **8** | **10** | **8** | **462** | **50.1** | **9.01** | **27.59** | **115929698** |
| **P11498** | **13** | **11** | **11** | **11** | **1178** | **129.6** | **6.84** | **24.89** | **39668986.38** |
| **Q00796** | **27** | **9** | **10** | **9** | **357** | **38.3** | **7.97** | **23.77** | **84328979** |
| **P08243** | **23** | **10** | **10** | **10** | **561** | **64.3** | **6.86** | **23.39** | **73237632.75** |
| **Q15323** | **22** | **9** | **9** | **8** | **416** | **47.2** | **4.88** | **21.28** | **70744693** |
| **P11142** | **16** | **9** | **9** | **8** | **646** | **70.9** | **5.52** | **21.27** | **45881691.75** |
| **P49327** | **4** | **8** | **8** | **8** | **2511** | **273.3** | **6.44** | **21.18** | **15686092.75** |
| **P20930** | **1** | **6** | **10** | **6** | **4061** | **434.9** | **9.25** | **19.61** | **95381701.5** |
| **Q86Y46** | **10** | **5** | **10** | **1** | **540** | **58.9** | **7.23** | **19.11** | **4190090.25** |
| **P05089** | **22** | **6** | **8** | **6** | **322** | **34.7** | **7.21** | **19.07** | **37308025** |
| **P02768** | **8** | **6** | **7** | **6** | **609** | **69.3** | **6.28** | **18.85** | **49241799.75** |
| **Q71U36** | **21** | **7** | **7** | **7** | **451** | **50.1** | **5.06** | **17.36** | **44234104.63** |
| **P30519** | **22** | **6** | **6** | **6** | **316** | **36** | **5.41** | **16.83** | **25536815.38** |
| **P63267** | **23** | **7** | **8** | **1** | **376** | **41.9** | **5.48** | **15.19** |  |
| **Q08188** | **7** | **4** | **5** | **4** | **693** | **76.6** | **5.86** | **14.95** | **21820933.5** |
| **P29508** | **18** | **5** | **5** | **5** | **390** | **44.5** | **6.81** | **13.83** | **26893634.5** |
| **P01040** | **33** | **3** | **4** | **3** | **98** | **11** | **5.5** | **12.99** | **35867649** |
| **P69905** | **24** | **3** | **4** | **3** | **142** | **15.2** | **8.68** | **12.4** | **41894249.75** |
| **Q2M2I5** | **5** | **3** | **4** | **1** | **525** | **55.1** | **4.96** | **12.11** | **3170911.25** |
| **Q15517** | **17** | **4** | **5** | **4** | **529** | **51.5** | **8.35** | **11.98** | **62777942.5** |
| **P22735** | **7** | **5** | **5** | **5** | **817** | **89.7** | **6.04** | **11.87** | **18624842.75** |
| **Q8IUC4** | **8** | **6** | **6** | **6** | **686** | **76.9** | **6.8** | **11.8** | **13365788.31** |
| **P78386** | **9** | **5** | **5** | **1** | **507** | **55.8** | **6.55** | **11.71** | **38357776** |
| **Q14533** | **9** | **5** | **6** | **1** | **505** | **54.9** | **5.47** | **11.43** | **102646039.9** |
| **P58107** | **6** | **4** | **4** | **4** | **5088** | **555.3** | **5.62** | **11** | **19272184.25** |
| **P11021** | **8** | **4** | **4** | **3** | **654** | **72.3** | **5.16** | **10.5** | **11424872.25** |
| **P04040** | **9** | **4** | **4** | **4** | **527** | **59.7** | **7.39** | **10.15** | **10809258.5** |
| **P32119** | **19** | **3** | **4** | **2** | **198** | **21.9** | **5.97** | **10** | **2711981** |
| **Q14574** | **6** | **3** | **3** | **3** | **896** | **99.9** | **6.1** | **9.77** | **7356728.313** |
| **Q9NQC3** | **3** | **2** | **3** | **2** | **1192** | **129.9** | **4.5** | **9.68** | **26978056.75** |
| **P54886** | **3** | **2** | **3** | **2** | **795** | **87.2** | **7.12** | **9.65** | **3022389** |
| **P10809** | **12** | **5** | **5** | **5** | **573** | **61** | **5.87** | **9.37** | **16481625.06** |
| **O43143** | **6** | **4** | **4** | **4** | **795** | **90.9** | **7.46** | **9.29** | **3989995** |
| **P26599** | **17** | **4** | **7** | **4** | **557** | **59.6** | **9.16** | **9.05** | **7255627.875** |
| **Q6ZVX7** | **11** | **3** | **3** | **3** | **275** | **30.8** | **6.62** | **8.65** | **30215680.5** |
| **P81605** | **23** | **3** | **3** | **3** | **110** | **11.3** | **6.54** | **8.64** | **15877604.75** |
| **Q13085** | **2** | **4** | **4** | **4** | **2346** | **265.4** | **6.37** | **8.41** | **6082254.125** |
| **P53597** | **4** | **1** | **2** | **1** | **346** | **36.2** | **8.79** | **8.29** | **18219799** |
| **Q16881** | **6** | **3** | **3** | **3** | **649** | **70.9** | **7.39** | **7.92** | **13580636** |
| **P25705** | **4** | **2** | **3** | **2** | **553** | **59.7** | **9.13** | **7.11** | **41956210.5** |
| **Q6UWP8** | **21** | **4** | **5** | **4** | **590** | **60.5** | **7.01** | **6.88** | **19475866.25** |
| **P27708** | **2** | **4** | **4** | **4** | **2225** | **242.8** | **6.46** | **6.78** | **2558788.75** |
| **Q9HCY8** | **32** | **3** | **3** | **3** | **104** | **11.7** | **5.24** | **6.68** | **15364455** |
| **P10599** | **24** | **2** | **3** | **2** | **105** | **11.7** | **4.92** | **6.57** | **23784679.5** |
| **P35321** | **37** | **2** | **3** | **1** | **89** | **9.9** | **8.48** | **6.51** | **4108957.25** |
| **Q9H4A4** | **4** | **3** | **3** | **3** | **650** | **72.5** | **5.74** | **6.49** | **10273261** |
| **Q13867** | **7** | **2** | **2** | **2** | **455** | **52.5** | **6.27** | **6.43** | **4361203.5** |
| **P50395** | **8** | **3** | **3** | **3** | **445** | **50.6** | **6.47** | **6.41** | **6765168.5** |
| **Q9NZT1** | **22** | **2** | **2** | **2** | **146** | **15.9** | **4.44** | **6.22** | **9126955** |
| **P04792** | **12** | **2** | **2** | **2** | **205** | **22.8** | **6.4** | **6.05** | **11031144.63** |
| **P47929** | **15** | **2** | **2** | **2** | **136** | **15.1** | **7.62** | **5.83** | **19850454** |
| **P09211** | **17** | **2** | **2** | **2** | **210** | **23.3** | **5.64** | **5.66** | **1739900.5** |
| **Q04828** | **6** | **2** | **2** | **2** | **323** | **36.8** | **7.88** | **5.34** | **12687318.5** |
| **P05109** | **12** | **1** | **2** | **1** | **93** | **10.8** | **7.03** | **5.04** | **8257689.75** |
| **P30101** | **5** | **2** | **2** | **2** | **505** | **56.7** | **6.35** | **5** |  |
| **P30048** | **10** | **2** | **2** | **2** | **256** | **27.7** | **7.78** | **4.82** | **2154209.5** |
| **Q5T750** | **4** | **2** | **2** | **2** | **250** | **26.2** | **7.97** | **4.81** | **27473610** |
| **Q9HCC0** | **7** | **3** | **3** | **3** | **563** | **61.3** | **7.68** | **4.75** | **3388947.5** |
| **Q92598** | **4** | **3** | **3** | **3** | **858** | **96.8** | **5.39** | **4.47** | **13168729.88** |
| **P78371** | **4** | **2** | **2** | **2** | **535** | **57.5** | **6.46** | **4.4** | **6557140.5** |
| **Q96QA5** | **4** | **2** | **2** | **2** | **445** | **49.3** | **5.29** | **4.39** | **7215853** |
| **Q8WUM4** | **2** | **2** | **2** | **2** | **868** | **96** | **6.52** | **4.33** | **4132774.375** |
| **P48506** | **3** | **2** | **2** | **2** | **637** | **72.7** | **6.09** | **4.27** | **2070956.875** |
| **P22528** | **28** | **2** | **2** | **1** | **89** | **9.9** | **8.48** | **4.23** | **71480028** |
| **Q8N2K0** | **5** | **2** | **2** | **2** | **398** | **45.1** | **8.65** | **4.22** | **2457900.75** |
| **P03915** | **3** | **1** | **1** | **1** | **603** | **67** | **9.03** | **4.13** | **2506065.25** |
| **Q9UHG3** | **3** | **2** | **2** | **2** | **505** | **56.6** | **6.18** | **4.13** | **2069138.25** |
| **P02786** | **3** | **2** | **2** | **2** | **760** | **84.8** | **6.61** | **3.81** | **3208125.063** |
| **O14880** | **9** | **1** | **1** | **1** | **152** | **16.5** | **9.38** | **3.32** | **710482.5** |
| **Q92685** | **2** | **1** | **1** | **1** | **438** | **50.1** | **9.44** | **3.19** | **3649932.75** |
| **P04183** | **8** | **1** | **1** | **1** | **234** | **25.5** | **8.51** | **3.05** | **2188907** |
| **Q15417** | **3** | **1** | **1** | **1** | **329** | **36.4** | **6.05** | **2.96** | **3249680.5** |
| **O00571** | **2** | **1** | **1** | **1** | **662** | **73.2** | **7.18** | **2.92** | **1682248.5** |
| **P52597** | **8** | **2** | **2** | **2** | **415** | **45.6** | **5.58** | **2.9** | **4887438.5** |
| **P00338** | **5** | **2** | **2** | **2** | **332** | **36.7** | **8.27** | **2.85** | **4137001.75** |
| **Q99873** | **3** | **1** | **1** | **1** | **371** | **42.4** | **5.35** | **2.78** | **2833639.75** |
| **P00390** | **2** | **1** | **1** | **1** | **522** | **56.2** | **8.5** | **2.75** |  |
| **Q8WVV4** | **2** | **1** | **1** | **1** | **589** | **68** | **6.32** | **2.74** | **2839408.75** |
| **Q13310** | **2** | **1** | **1** | **1** | **644** | **70.7** | **9.26** | **2.7** | **1553698.25** |
| **P13804** | **6** | **1** | **1** | **1** | **333** | **35.1** | **8.38** | **2.68** | **5122015.5** |
| **Q7Z434** | **3** | **1** | **1** | **1** | **540** | **56.5** | **5.52** | **2.63** |  |
| **P22314** | **1** | **1** | **1** | **1** | **1058** | **117.8** | **5.76** | **2.62** | **2833518.25** |
| **O95816** | **3** | **1** | **1** | **1** | **211** | **23.8** | **6.7** | **2.62** |  |
| **P12814** | **1** | **1** | **1** | **1** | **892** | **103** | **5.41** | **2.61** | **934455.4375** |
| **P25311** | **4** | **1** | **1** | **1** | **298** | **34.2** | **6.05** | **2.61** | **2250138.75** |
| **P49915** | **2** | **1** | **1** | **1** | **693** | **76.7** | **6.87** | **2.54** | **2282123.5** |
| **P29401** | **2** | **1** | **1** | **1** | **623** | **67.8** | **7.66** | **2.52** | **2951875.25** |
| **Q9H6S3** | **2** | **1** | **1** | **1** | **715** | **80.6** | **6.84** | **2.48** | **2614577.75** |
| **O94919** | **3** | **1** | **1** | **1** | **500** | **55** | **5.71** | **2.48** |  |
| **A5YKK6** | **0** | **1** | **1** | **1** | **2376** | **266.8** | **7.11** | **2.46** | **1017868.438** |
| **Q9NZB2** | **2** | **1** | **1** | **1** | **1118** | **121.8** | **8.88** | **2.46** | **1372248.625** |
| **P55884** | **1** | **1** | **1** | **1** | **814** | **92.4** | **5** | **2.42** | **5825769.5** |
| **P21796** | **4** | **1** | **1** | **1** | **283** | **30.8** | **8.54** | **2.34** |  |
| **P00491** | **8** | **1** | **1** | **1** | **289** | **32.1** | **6.95** | **2.33** | **13699045** |
| **Q7Z2K6** | **2** | **2** | **2** | **2** | **904** | **100.2** | **7.52** | **2.32** | **1681952.25** |
| **Q9UL46** | **13** | **2** | **2** | **2** | **239** | **27.4** | **5.73** | **2.31** | **3620723.5** |
| **Q96P70** | **3** | **2** | **2** | **2** | **1041** | **115.9** | **4.81** | **2.28** | **4167970.25** |
| **P15531** | **6** | **1** | **1** | **1** | **152** | **17.1** | **6.19** | **2.28** |  |
| **Q06323** | **5** | **1** | **1** | **1** | **249** | **28.7** | **6.02** | **2.26** | **817651.5625** |
| **P35579** | **1** | **1** | **1** | **1** | **1960** | **226.4** | **5.6** | **2.24** | **3655949** |
| **O95210** | **5** | **1** | **1** | **1** | **358** | **39** | **5.73** | **2.22** | **9470605** |
| **Q9BT22** | **3** | **1** | **1** | **1** | **464** | **52.5** | **7.23** | **2.18** | **1053305.75** |
| **P50552** | **3** | **1** | **1** | **1** | **380** | **39.8** | **8.94** | **2.15** | **5733211** |
| **A6NEC2** | **2** | **1** | **1** | **1** | **478** | **53.7** | **5.34** | **2.14** | **2588876.5** |
| **Q8NB16** | **2** | **1** | **1** | **1** | **471** | **54.4** | **8.82** | **2.1** | **2263425.25** |
| **A0FGR8** | **1** | **1** | **1** | **1** | **921** | **102.3** | **9.26** | **2.09** | **5022275.5** |
| **P60174** | **17** | **2** | **2** | **2** | **249** | **26.7** | **6.9** | **2.07** | **2897110.375** |
| **O75153** | **1** | **1** | **1** | **1** | **1309** | **146.6** | **6.13** | **2.04** | **794474.375** |
| **P45974** | **1** | **1** | **1** | **1** | **858** | **95.7** | **5.03** | **2.01** | **1701681.125** |
| **P54136** | **3** | **2** | **2** | **2** | **660** | **75.3** | **6.68** | **2.01** | **4514250** |
| **Q16650** | **2** | **1** | **1** | **1** | **682** | **74** | **7.33** | **1.94** | **2355668** |
| **O75449** | **2** | **1** | **1** | **1** | **491** | **55.9** | **6.9** | **1.9** |  |
| **Q12906** | **1** | **1** | **1** | **1** | **894** | **95.3** | **8.76** | **1.89** | **1420726.375** |
| **Q9Y446** | **1** | **1** | **1** | **1** | **797** | **87** | **9.32** | **1.86** | **1969855.5** |
| **Q6PIU2** | **3** | **1** | **1** | **1** | **408** | **45.8** | **7.23** | **1.8** |  |
| **P47755** | **3** | **1** | **1** | **1** | **286** | **32.9** | **5.85** | **1.79** |  |
| **P29144** | **1** | **1** | **1** | **1** | **1249** | **138.3** | **6.32** | **1.74** | **1200684.375** |
| **Q7L5N7** | **1** | **1** | **1** | **1** | **544** | **60.2** | **6.55** | **1.65** | **1830313.375** |
| **P30041** | **3** | **1** | **1** | **1** | **224** | **25** | **6.38** | **1.62** | **2068637.25** |
| **Q96DT5** | **1** | **1** | **2** | **1** | **4516** | **520** | **6.44** | **0** |  |
| **Q96RQ3** | **2** | **1** | **1** | **1** | **725** | **80.4** | **7.78** | **0** |  |
| **Q96HC4** | **2** | **1** | **1** | **1** | **596** | **63.9** | **8.21** | **0** | **1806488.375** |
| **P22570** | **1** | **1** | **1** | **1** | **491** | **53.8** | **8.44** | **0** | **16975862** |
| **P04083** | **4** | **1** | **1** | **1** | **346** | **38.7** | **7.02** | **0** | **3577851.75** |
| **Q9H0E2** | **3** | **1** | **1** | **1** | **274** | **30.3** | **5.97** | **0** | **3209169.25** |
| **P50990** | **2** | **1** | **1** | **1** | **548** | **59.6** | **5.6** | **0** | **1387576.75** |
| **P62701** | **3** | **1** | **1** | **1** | **263** | **29.6** | **10.15** | **0** |  |
| **Q99714** | **5** | **1** | **1** | **1** | **261** | **26.9** | **7.78** | **2.14** | **5151268.5** |
| **P57768** | **2** | **1** | **1** | **1** | **344** | **39.1** | **4.65** | **2.96** | **10988605** |
| **P61626** | **6** | **1** | **1** | **1** | **148** | **16.5** | **9.16** | **2.46** | **1311885.75** |
| **P15170** | **2** | **1** | **1** | **1** | **499** | **55.7** | **5.62** | **2.36** |  |
| **O43490** | **1** | **1** | **1** | **1** | **865** | **97.1** | **7.27** | **2.31** | **4320899.5** |
| **Q7Z7A1** | **0** | **1** | **1** | **1** | **2325** | **268.7** | **5.55** | **2.21** | **25630518** |
| **P17931** | **4** | **1** | **1** | **1** | **250** | **26.1** | **8.56** | **2.18** | **5857148.5** |
| **Q9Y6U7** | **2** | **1** | **1** | **1** | **377** | **41.1** | **8.65** | **2.14** | **3321968.5** |
| **Q9Y5I4** | **1** | **1** | **1** | **1** | **1007** | **109.4** | **5.41** | **2.13** | **22188748** |
| **P06703** | **9** | **1** | **1** | **1** | **90** | **10.2** | **5.48** | **2.13** | **4015627.25** |
| **P47756** | **4** | **1** | **1** | **1** | **272** | **30.6** | **6** | **2.12** | **2542078.5** |
| **O96008** | **4** | **1** | **1** | **1** | **361** | **37.9** | **7.25** | **2.06** | **1162144.875** |
| **O43150** | **1** | **1** | **1** | **1** | **1006** | **111.6** | **6.68** | **2.04** | **2289668** |
| **P50402** | **4** | **1** | **1** | **1** | **254** | **29** | **5.5** | **2.03** |  |
| **Q9P281** | **0** | **1** | **1** | **1** | **2639** | **279.8** | **8.78** | **1.99** |  |
| **Q99497** | **4** | **1** | **1** | **1** | **189** | **19.9** | **6.79** | **1.92** | **2039549.875** |
| **P19338** | **1** | **1** | **1** | **1** | **710** | **76.6** | **4.7** | **1.87** | **1910642.25** |
| **P17031** | **2** | **1** | **1** | **1** | **533** | **61.2** | **8.84** | **1.85** | **1730111.75** |
| **Q5BJF2** | **5** | **1** | **1** | **1** | **176** | **20.8** | **9.38** | **1.81** | **3181311.75** |
| **P14735** | **1** | **1** | **1** | **1** | **1019** | **117.9** | **6.61** | **1.69** |  |
| **Q8WUY1** | **4** | **1** | **1** | **1** | **208** | **23.9** | **9.55** | **1.63** | **921924.1875** |
| **P62873** | **2** | **1** | **1** | **1** | **340** | **37.4** | **6** | **1.62** | **3545346.75** |
| **P01857** | **2** | **1** | **1** | **1** | **330** | **36.1** | **8.19** | **1.6** | **4846335** |
| **P46939** | **1** | **1** | **1** | **1** | **3433** | **394.2** | **5.33** | **0** | **2221852.75** |
| **Q04721** | **1** | **1** | **1** | **1** | **2471** | **265.2** | **5.14** | **0** | **7355081.5** |
| **Q96BY6** | **2** | **1** | **1** | **1** | **2186** | **249.4** | **7.14** | **0** |  |
| **Q7Z3U7** | **2** | **1** | **3** | **1** | **1717** | **190.2** | **6.06** | **0** |  |
| **P46940** | **2** | **1** | **1** | **1** | **1657** | **189.1** | **6.48** | **0** | **1995519.875** |
| **P07814** | **1** | **1** | **1** | **1** | **1512** | **170.5** | **7.33** | **0** |  |
| **Q8TEQ6** | **1** | **1** | **1** | **1** | **1508** | **168.5** | **6.62** | **0** | **4033521.5** |
| **Q7Z2D5** | **1** | **1** | **1** | **1** | **763** | **82.9** | **8.73** | **0** | **11930779** |
| **O15270** | **4** | **1** | **2** | **1** | **562** | **62.9** | **7.78** | **0** |  |
| **Q8WTV0** | **1** | **1** | **1** | **1** | **552** | **60.8** | **8.24** | **0** | **2826377.5** |
| **Q8WTX9** | **5** | **1** | **1** | **1** | **485** | **54.8** | **10.32** | **0** | **5287039.5** |
| **P53567** | **27** | **1** | **1** | **1** | **150** | **16.4** | **9.77** | **0** | **1316066.5** |

**Supplemental Table 5 Mass spectrometry profile of proteins enriched by PTL-6 in lysate**

| **Accession** | **Coverage [%]** | **# Peptides** | **# PSMs** | **# Unique Peptides** | **# AAs** | **MW [kDa]** | **calc. pI** | **Score Sequest HT: Sequest HT** | **Abundances (Normalized): F3: Sample** |
| --- | --- | --- | --- | --- | --- | --- | --- | --- | --- |
| **P04264** | **55** | **40** | **83** | **34** | **644** | **66** | **8.12** | **258.65** | **3198568852** |
| **P35908** | **72** | **39** | **67** | **31** | **639** | **65.4** | **8** | **188.78** | **1279362788** |
| **P13645** | **55** | **29** | **54** | **24** | **584** | **58.8** | **5.21** | **152.34** | **1850518159** |
| **P68363** | **45** | **16** | **31** | **3** | **451** | **50.1** | **5.06** | **99.62** | **681709455.2** |
| **P35527** | **44** | **24** | **35** | **23** | **623** | **62** | **5.24** | **98.36** | **680462907.6** |
| **P68366** | **43** | **15** | **30** | **2** | **448** | **49.9** | **5.06** | **94.85** | **6145487** |
| **P07437** | **47** | **18** | **29** | **4** | **444** | **49.6** | **4.89** | **81.67** | **94669431.13** |
| **P08238** | **32** | **23** | **29** | **23** | **724** | **83.2** | **5.03** | **79.79** | **401459276.5** |
| **P10809** | **41** | **21** | **26** | **21** | **573** | **61** | **5.87** | **79.18** | **253723336.5** |
| **P68371** | **44** | **18** | **27** | **4** | **445** | **49.8** | **4.89** | **74.53** | **463170398.3** |
| **P68104** | **39** | **13** | **25** | **13** | **462** | **50.1** | **9.01** | **69.66** | **665354492.4** |
| **P02533** | **38** | **16** | **24** | **6** | **472** | **51.5** | **5.16** | **60.6** | **49984927.63** |
| **P13647** | **30** | **19** | **22** | **10** | **590** | **62.3** | **7.74** | **57.94** | **112296988** |
| **P08779** | **40** | **17** | **24** | **8** | **473** | **51.2** | **5.05** | **56.11** | **92079568.63** |
| **P02538** | **32** | **17** | **20** | **1** | **564** | **60** | **8** | **51.48** | **40432614** |
| **P04259** | **31** | **16** | **19** | **1** | **564** | **60** | **8** | **48.45** | **8128895.5** |
| **P07355** | **41** | **12** | **14** | **12** | **339** | **38.6** | **7.75** | **41.08** | **100296751** |
| **P05787** | **25** | **12** | **16** | **7** | **483** | **53.7** | **5.59** | **40.01** | **30000207** |
| **P78527** | **5** | **17** | **17** | **17** | **4128** | **468.8** | **7.12** | **38.51** | **52462578** |
| **Q15323** | **25** | **10** | **14** | **4** | **416** | **47.2** | **4.88** | **36.48** | **231017921** |
| **Q9BUF5** | **15** | **6** | **12** | **1** | **446** | **49.8** | **4.88** | **36.41** | **12910959** |
| **P04406** | **36** | **8** | **12** | **8** | **335** | **36** | **8.46** | **33.7** | **97723765.13** |
| **P13646** | **9** | **5** | **10** | **1** | **458** | **49.6** | **4.96** | **31.14** | **3738631.75** |
| **P60709** | **32** | **9** | **10** | **9** | **375** | **41.7** | **5.48** | **30.48** | **143783955.5** |
| **P78385** | **15** | **9** | **11** | **1** | **493** | **54.2** | **5.64** | **29.57** | **186703829** |
| **O95678** | **14** | **9** | **11** | **2** | **551** | **59.5** | **7.74** | **29.33** | **33436204** |
| **P23396** | **60** | **11** | **11** | **11** | **243** | **26.7** | **9.66** | **29.09** | **82129045.13** |
| **A6NCN2** | **26** | **9** | **12** | **3** | **255** | **29.1** | **5.8** | **28.83** | **4337127** |
| **P78386** | **14** | **8** | **10** | **1** | **507** | **55.8** | **6.55** | **27.78** | **16031794** |
| **P05141** | **31** | **9** | **12** | **3** | **298** | **32.8** | **9.69** | **27.78** | **197430307.9** |
| **O14980** | **12** | **9** | **10** | **9** | **1071** | **123.3** | **6.06** | **26.24** | **22203602.94** |
| **O60701** | **23** | **9** | **10** | **9** | **494** | **55** | **7.12** | **25.62** | **47991307.38** |
| **P55060** | **13** | **10** | **10** | **10** | **971** | **110.3** | **5.77** | **25.15** | **48083692** |
| **Q92764** | **11** | **7** | **9** | **2** | **455** | **50.3** | **4.91** | **23.69** | **10077316.38** |
| **Q92616** | **4** | **10** | **10** | **10** | **2671** | **292.5** | **7.43** | **21.94** | **22400675.75** |
| **P56470** | **24** | **6** | **8** | **6** | **323** | **35.9** | **9.16** | **21.58** | **40633852.63** |
| **P25705** | **17** | **8** | **8** | **8** | **553** | **59.7** | **9.13** | **20.85** | **27320449** |
| **Q04695** | **11** | **6** | **8** | **1** | **432** | **48.1** | **5.02** | **20.69** | **3918000** |
| **P05023** | **10** | **8** | **8** | **8** | **1023** | **112.8** | **5.49** | **20.25** | **23431375.81** |
| **P12236** | **27** | **8** | **10** | **2** | **298** | **32.8** | **9.74** | **20.19** | **6534069** |
| **Q15758** | **16** | **8** | **8** | **8** | **541** | **56.6** | **5.48** | **20.11** | **40769657.13** |
| **Q86VP6** | **8** | **8** | **8** | **8** | **1230** | **136.3** | **5.78** | **19.85** | **23085080.13** |
| **Q15365** | **39** | **8** | **8** | **6** | **356** | **37.5** | **7.09** | **18.91** | **81089252.25** |
| **P45880** | **20** | **6** | **6** | **6** | **294** | **31.5** | **7.56** | **17.79** | **25509247.13** |
| **P11142** | **12** | **6** | **6** | **6** | **646** | **70.9** | **5.52** | **16.74** | **11829838.13** |
| **Q99832** | **13** | **7** | **7** | **7** | **543** | **59.3** | **7.65** | **15.93** | **19562273.13** |
| **P13639** | **9** | **7** | **7** | **7** | **858** | **95.3** | **6.83** | **15.2** | **23817531.63** |
| **O76013** | **11** | **5** | **6** | **3** | **467** | **52.2** | **4.94** | **14.78** | **25656614** |
| **P00558** | **22** | **6** | **7** | **6** | **417** | **44.6** | **8.1** | **14.71** | **15616270.69** |
| **P49368** | **12** | **6** | **6** | **6** | **545** | **60.5** | **6.49** | **14.64** | **24689389.88** |
| **P56192** | **8** | **6** | **6** | **6** | **900** | **101.1** | **6.16** | **13.82** | **9497366.625** |
| **Q9Y678** | **7** | **6** | **6** | **6** | **874** | **97.7** | **5.47** | **13.63** | **14441898.75** |
| **P49411** | **15** | **5** | **5** | **5** | **455** | **49.8** | **7.61** | **13.63** | **11337740** |
| **Q06830** | **22** | **4** | **5** | **4** | **199** | **22.1** | **8.13** | **13.33** | **25150833** |
| **O00410** | **8** | **6** | **6** | **6** | **1097** | **123.6** | **4.94** | **13.16** | **25521969.94** |
| **P02768** | **5** | **4** | **5** | **4** | **609** | **69.3** | **6.28** | **13** | **51824373.88** |
| **O43175** | **11** | **5** | **5** | **5** | **533** | **56.6** | **6.71** | **12.75** | **16542366.25** |
| **P14618** | **16** | **6** | **6** | **6** | **531** | **57.9** | **7.84** | **12.51** | **20417823.25** |
| **P50990** | **10** | **5** | **5** | **5** | **548** | **59.6** | **5.6** | **12.5** | **16700802.25** |
| **P31930** | **15** | **4** | **4** | **4** | **480** | **52.6** | **6.37** | **12.14** | **10224375.13** |
| **Q15366** | **14** | **4** | **4** | **2** | **365** | **38.6** | **6.79** | **11.7** | **8544177.5** |
| **P69905** | **25** | **3** | **4** | **3** | **142** | **15.2** | **8.68** | **11.7** | **18859437.38** |
| **P17987** | **12** | **5** | **5** | **5** | **556** | **60.3** | **6.11** | **11.48** | **14020023.75** |
| **Q92973** | **5** | **4** | **4** | **4** | **898** | **102.3** | **4.98** | **11.36** | **20245144.25** |
| **P09110** | **16** | **5** | **5** | **5** | **424** | **44.3** | **8.44** | **11.36** | **15023216.38** |
| **P52292** | **10** | **4** | **4** | **4** | **529** | **57.8** | **5.4** | **11.09** | **18931520.75** |
| **P22695** | **12** | **4** | **4** | **4** | **453** | **48.4** | **8.63** | **9.97** | **13050561.25** |
| **Q8NB16** | **8** | **3** | **4** | **3** | **471** | **54.4** | **8.82** | **6.51** | **5372418.75** |
| **Q14974** | **5** | **4** | **4** | **4** | **876** | **97.1** | **4.78** | **9.68** | **15139415.5** |
| **Q9NSB4** | **8** | **4** | **4** | **2** | **513** | **56.6** | **6.74** | **9.6** | **80721708** |
| **P62701** | **16** | **4** | **4** | **4** | **263** | **29.6** | **10.15** | **9.45** | **20759713.75** |
| **P60842** | **11** | **4** | **4** | **4** | **406** | **46.1** | **5.48** | **9.12** | **23710506.63** |
| **P62987** | **27** | **3** | **3** | **3** | **128** | **14.7** | **9.83** | **8.6** | **30081376** |
| **P05783** | **12** | **4** | **4** | **3** | **430** | **48** | **5.45** | **8.58** | **10301315.25** |
| **P27348** | **12** | **3** | **3** | **1** | **245** | **27.7** | **4.78** | **8.48** | **9772106** |
| **P11413** | **9** | **4** | **4** | **4** | **515** | **59.2** | **6.84** | **8.46** | **6931413.5** |
| **P11166** | **4** | **3** | **3** | **3** | **492** | **54** | **8.72** | **8.41** | **15459103.25** |
| **P61619** | **7** | **3** | **4** | **3** | **476** | **52.2** | **8.06** | **8.38** | **20958481.75** |
| **P08195** | **7** | **3** | **3** | **3** | **630** | **68** | **5.01** | **8.22** | **4623193** |
| **P31946** | **12** | **3** | **3** | **1** | **246** | **28.1** | **4.83** | **8.19** | **38353740.25** |
| **P04843** | **5** | **3** | **3** | **3** | **607** | **68.5** | **6.38** | **7.58** | **7178699** |
| **P42704** | **2** | **3** | **3** | **3** | **1394** | **157.8** | **6.13** | **7.37** | **8530839.375** |
| **O95373** | **4** | **4** | **4** | **4** | **1038** | **119.4** | **4.82** | **7.24** | **11583972.75** |
| **P16615** | **4** | **3** | **3** | **3** | **1042** | **114.7** | **5.34** | **7.15** | **4894470** |
| **P50991** | **7** | **3** | **3** | **3** | **539** | **57.9** | **7.83** | **7.15** | **6088259.375** |
| **P53597** | **4** | **1** | **2** | **1** | **346** | **36.2** | **8.79** | **6.99** | **16351499.75** |
| **P30740** | **9** | **3** | **3** | **3** | **379** | **42.7** | **6.28** | **6.94** | **6549077.375** |
| **Q9HAW8** | **6** | **3** | **3** | **2** | **530** | **59.8** | **7.3** | **6.74** | **4856285.75** |
| **P36578** | **5** | **2** | **2** | **2** | **427** | **47.7** | **11.06** | **6.61** | **9018095.75** |
| **P63104** | **9** | **2** | **2** | **1** | **245** | **27.7** | **4.79** | **5.69** | **2814337.75** |
| **P54920** | **8** | **2** | **2** | **2** | **295** | **33.2** | **5.36** | **5.59** | **5130735.75** |
| **P57088** | **9** | **2** | **2** | **2** | **247** | **28** | **9.7** | **5.54** | **5453073.5** |
| **P00352** | **9** | **3** | **3** | **3** | **501** | **54.8** | **6.73** | **5.35** | **6944957.375** |
| **P06733** | **5** | **2** | **2** | **2** | **434** | **47.1** | **7.39** | **5.34** | **5137825.125** |
| **Q04721** | **1** | **1** | **2** | **1** | **2471** | **265.2** | **5.14** | **5.26** | **6496276192** |
| **O60218** | **8** | **2** | **2** | **2** | **316** | **36** | **7.84** | **5.24** | **7645597.25** |
| **O75746** | **3** | **2** | **2** | **2** | **678** | **74.7** | **8.38** | **5.1** | **3056855.875** |
| **P30153** | **6** | **3** | **3** | **3** | **589** | **65.3** | **5.11** | **5.08** | **9153979.875** |
| **O75874** | **8** | **3** | **3** | **3** | **414** | **46.6** | **7.01** | **5.02** | **12150951.75** |
| **P53618** | **4** | **2** | **2** | **2** | **953** | **107.1** | **6.05** | **4.99** | **1097563.75** |
| **Q9Y5L0** | **1** | **1** | **2** | **1** | **923** | **104.1** | **5.57** | **4.95** | **3217778.25** |
| **P26641** | **7** | **3** | **3** | **3** | **437** | **50.1** | **6.67** | **4.95** | **6825619.063** |
| **Q15233** | **4** | **2** | **2** | **2** | **471** | **54.2** | **8.95** | **4.76** | **13410511** |
| **O95197** | **2** | **2** | **2** | **2** | **1032** | **112.5** | **4.96** | **4.69** | **11448447** |
| **Q96L21** | **9** | **2** | **2** | **2** | **214** | **24.5** | **10.01** | **4.63** | **11773008** |
| **P38646** | **3** | **2** | **2** | **2** | **679** | **73.6** | **6.16** | **4.59** | **5245092.375** |
| **P61204** | **6** | **1** | **2** | **1** | **181** | **20.6** | **7.43** | **4.56** | **2023540.75** |
| **Q00325** | **6** | **2** | **2** | **2** | **362** | **40.1** | **9.38** | **4.45** | **16445152** |
| **Q9ULC5** | **3** | **2** | **2** | **2** | **683** | **75.9** | **6.92** | **4.31** | **5920112** |
| **Q8N1N4** | **4** | **2** | **2** | **1** | **520** | **56.8** | **6.02** | **4.19** | **967807.5** |
| **P62753** | **8** | **2** | **2** | **2** | **249** | **28.7** | **10.84** | **4.19** | **11752811.25** |
| **Q9Y6C9** | **6** | **2** | **2** | **2** | **303** | **33.3** | **7.97** | **4.17** | **6297090** |
| **P61247** | **6** | **2** | **2** | **2** | **264** | **29.9** | **9.73** | **4.11** | **12736501.5** |
| **P19224** | **3** | **2** | **2** | **1** | **532** | **60.7** | **8.41** | **4.03** | **944456.4375** |
| **Q9Y6E2** | **4** | **2** | **2** | **2** | **419** | **48.1** | **6.68** | **3.97** | **13266792** |
| **Q9UBM7** | **4** | **2** | **2** | **2** | **475** | **54.5** | **8.7** | **3.93** | **10229347.5** |
| **Q14534** | **4** | **1** | **1** | **1** | **574** | **63.9** | **8.63** | **3.65** | **1165619.125** |
| **Q06210** | **4** | **2** | **2** | **2** | **699** | **78.8** | **7.11** | **3.53** | **2418254.25** |
| **A0A087WW87** | **11** | **1** | **1** | **1** | **121** | **13.3** | **4.61** | **3.38** | **6361205.5** |
| **P46782** | **4** | **1** | **1** | **1** | **204** | **22.9** | **9.72** | **3.29** | **4542220** |
| **P62917** | **4** | **1** | **1** | **1** | **257** | **28** | **11.03** | **3.16** | **8229955.5** |
| **P00338** | **3** | **1** | **1** | **1** | **332** | **36.7** | **8.27** | **2.92** | **4580631** |
| **P50995** | **2** | **1** | **1** | **1** | **505** | **54.4** | **7.65** | **2.83** | **2967352** |
| **P49327** | **0** | **1** | **1** | **1** | **2511** | **273.3** | **6.44** | **2.79** | **1880019** |
| **P83731** | **8** | **1** | **1** | **1** | **157** | **17.8** | **11.25** | **2.75** | **3508625.5** |
| **Q96T76** | **2** | **2** | **2** | **2** | **1030** | **113.2** | **6.35** | **2.72** | **2663399.875** |
| **O95573** | **3** | **2** | **2** | **2** | **720** | **80.4** | **8.38** | **2.69** | **3942759.125** |
| **P36542** | **4** | **1** | **1** | **1** | **298** | **33** | **9.22** | **2.67** | **2102685** |
| **O43592** | **1** | **1** | **1** | **1** | **962** | **109.9** | **5.39** | **2.63** | **1165708.75** |
| **P46776** | **7** | **1** | **1** | **1** | **148** | **16.6** | **11** | **2.59** | **2196032.75** |
| **Q92945** | **2** | **1** | **1** | **1** | **711** | **73.1** | **7.3** | **2.57** | **1669956.875** |
| **Q13011** | **5** | **1** | **1** | **1** | **328** | **35.8** | **8** | **2.54** | **1508786.125** |
| **Q9NZL4** | **3** | **1** | **1** | **1** | **359** | **39.3** | **5.21** | **2.53** | **1117635** |
| **P14923** | **2** | **1** | **1** | **1** | **745** | **81.7** | **6.14** | **2.52** | **867618.3125** |
| **Q8TB61** | **5** | **2** | **2** | **2** | **432** | **47.5** | **9.16** | **2.51** | **31381640.5** |
| **P52895** | **2** | **1** | **1** | **1** | **323** | **36.7** | **7.49** | **2.46** | **1896435.5** |
| **Q96N66** | **3** | **1** | **1** | **1** | **472** | **52.7** | **8.97** | **2.45** | **1453388.75** |
| **P07195** | **3** | **1** | **1** | **1** | **334** | **36.6** | **6.05** | **2.44** | **3619230** |
| **Q13813** | **0** | **1** | **1** | **1** | **2472** | **284.4** | **5.35** | **2.39** | **1030949.625** |
| **A8MTJ3** | **3** | **1** | **1** | **1** | **354** | **40.3** | **6.01** | **2.35** | **4643708.5** |
| **P48047** | **7** | **1** | **1** | **1** | **213** | **23.3** | **9.96** | **2.35** | **661026.25** |
| **P02100** | **7** | **1** | **1** | **1** | **147** | **16.2** | **8.63** | **2.34** | **11912032** |
| **P63244** | **6** | **2** | **2** | **2** | **317** | **35.1** | **7.69** | **2.33** | **4236875.063** |
| **Q9UIA9** | **1** | **1** | **1** | **1** | **1087** | **123.8** | **6.32** | **2.32** | **1063243.125** |
| **P38606** | **2** | **1** | **1** | **1** | **617** | **68.3** | **5.52** | **2.32** | **1578538.25** |
| **P62241** | **7** | **1** | **1** | **1** | **208** | **24.2** | **10.32** | **2.31** | **885505.875** |
| **P55786** | **1** | **1** | **1** | **1** | **919** | **103.2** | **5.72** | **2.3** | **954749** |
| **P42167** | **4** | **1** | **1** | **1** | **454** | **50.6** | **9.38** | **2.3** | **1188971.375** |
| **P62851** | **7** | **1** | **1** | **1** | **125** | **13.7** | **10.11** | **2.28** | **2230787** |
| **Q14728** | **2** | **1** | **1** | **1** | **455** | **48.3** | **9.6** | **2.26** | **3081829.75** |
| **P46977** | **2** | **1** | **1** | **1** | **705** | **80.5** | **8.07** | **2.24** | **1947652.5** |
| **Q6P996** | **1** | **1** | **1** | **1** | **788** | **86.7** | **5.38** | **2.22** | **1885264.625** |
| **P13804** | **6** | **1** | **1** | **1** | **333** | **35.1** | **8.38** | **2.22** | **2649860.25** |
| **P46060** | **2** | **1** | **1** | **1** | **587** | **63.5** | **4.68** | **2.2** | **1017975.188** |
| **P98172** | **4** | **1** | **1** | **1** | **346** | **38** | **8.94** | **2.2** | **1597393.375** |
| **Q02413** | **2** | **1** | **1** | **1** | **1049** | **113.7** | **5.03** | **2.19** | **819527.3125** |
| **Q9P035** | **6** | **1** | **1** | **1** | **362** | **43.1** | **8.94** | **2.19** | **2295877** |
| **P11498** | **1** | **1** | **1** | **1** | **1178** | **129.6** | **6.84** | **2.18** | **3426618** |
| **P54578** | **2** | **1** | **1** | **1** | **494** | **56** | **5.3** | **2.18** | **1190219** |
| **Q16186** | **4** | **1** | **1** | **1** | **407** | **42.1** | **5.07** | **2.16** | **1701235** |
| **P61978** | **4** | **1** | **1** | **1** | **463** | **50.9** | **5.54** | **2.15** | **1846000.25** |
| **Q99714** | **10** | **1** | **1** | **1** | **261** | **26.9** | **7.78** | **2.15** | **805057.75** |
| **O75369** | **0** | **1** | **1** | **1** | **2602** | **278** | **5.73** | **2.08** | **1838566.25** |
| **Q9HD45** | **3** | **2** | **2** | **2** | **589** | **67.8** | **7.21** | **2.06** | **1907159.5** |
| **P40616** | **5** | **1** | **1** | **1** | **181** | **20.4** | **5.72** | **2.06** | **955046.625** |
| **P62249** | **7** | **1** | **1** | **1** | **146** | **16.4** | **10.21** | **2.06** | **2309426.75** |
| **Q96I24** | **2** | **1** | **1** | **1** | **572** | **61.6** | **8.38** | **2.02** | **3040633.75** |
| **P36957** | **2** | **1** | **1** | **1** | **453** | **48.7** | **8.95** | **1.99** | **12775168** |
| **P40926** | **3** | **1** | **1** | **1** | **338** | **35.5** | **8.68** | **1.92** | **2503532** |
| **P50454** | **3** | **1** | **1** | **1** | **418** | **46.4** | **8.69** | **1.88** | **1493392.625** |
| **P23526** | **2** | **1** | **1** | **1** | **432** | **47.7** | **6.34** | **1.87** | **1612028** |
| **Q96P63** | **2** | **1** | **1** | **1** | **405** | **46.2** | **5.53** | **1.87** | **2199058.5** |
| **Q7Z739** | **2** | **1** | **1** | **1** | **585** | **63.8** | **9.04** | **1.84** | **996470.125** |
| **Q9HAV4** | **1** | **1** | **1** | **1** | **1204** | **136.2** | **5.8** | **1.82** | **1286600** |
| **Q5D862** | **0** | **1** | **1** | **1** | **2391** | **247.9** | **8.31** | **1.81** | **1525268.375** |
| **P11216** | **1** | **1** | **1** | **1** | **843** | **96.6** | **6.86** | **1.8** | **2147493** |
| **Q04760** | **9** | **2** | **2** | **2** | **184** | **20.8** | **5.31** | **1.8** | **1379901.063** |
| **P12268** | **4** | **2** | **2** | **2** | **514** | **55.8** | **6.9** | **1.79** | **4681907.75** |
| **P41252** | **1** | **1** | **1** | **1** | **1262** | **144.4** | **6.15** | **1.76** | **1338545.875** |
| **Q6NUQ4** | **1** | **1** | **1** | **1** | **689** | **77.1** | **9.14** | **1.76** | **1383394.75** |
| **Q02543** | **6** | **1** | **1** | **1** | **176** | **20.7** | **10.71** | **1.74** | **2263679.25** |
| **P22234** | **3** | **1** | **1** | **1** | **425** | **47** | **7.23** | **1.72** | **923966.375** |
| **P11172** | **3** | **1** | **1** | **1** | **480** | **52.2** | **7.24** | **1.6** | **1363546.625** |
| **Q92621** | **1** | **1** | **1** | **1** | **2012** | **227.8** | **6.19** | **0** | **957159.5625** |
| **Q5VZK9** | **2** | **1** | **1** | **1** | **1371** | **151.5** | **7.85** | **0** |  |
| **O60716** | **1** | **1** | **1** | **1** | **968** | **108.1** | **6.23** | **0** | **852164.125** |
| **Q9UEW8** | **2** | **1** | **1** | **1** | **545** | **59.4** | **6.29** | **0** | **1954228** |
| **Q6NUK1** | **2** | **1** | **1** | **1** | **477** | **53.3** | **6.33** | **0** | **5751163.5** |
| **Q9NVF9** | **2** | **1** | **1** | **1** | **386** | **44.8** | **7.33** | **0** |  |
| **P04075** | **4** | **1** | **1** | **1** | **364** | **39.4** | **8.09** | **0** | **3802100** |
| **P60903** | **18** | **1** | **1** | **1** | **97** | **11.2** | **7.37** | **0** | **2331631.75** |

**Supplemental Table 6 Mass spectrometry profile of proteins enriched by PH-NN in situ**

| **Accession** | **Coverage [%]** | **# Peptides** | **# PSMs** | **# Unique Peptides** | **# AAs** | **MW [kDa]** | **calc. pI** | **Score Sequest HT: Sequest HT** | **Abundances (Normalized): F2: Sample** |
| --- | --- | --- | --- | --- | --- | --- | --- | --- | --- |
| **P04264** | **48** | **38** | **99** | **33** | **644** | **66** | **8.12** | **293.94** | **3.1E+09** |
| **P35527** | **64** | **26** | **55** | **26** | **623** | **62** | **5.24** | **183.12** | **1.1E+09** |
| **P35908** | **72** | **36** | **51** | **27** | **639** | **65.4** | **8** | **136.41** | **5.68E+08** |
| **P13645** | **49** | **25** | **50** | **20** | **584** | **58.8** | **5.21** | **153.63** | **1.59E+09** |
| **P04259** | **33** | **19** | **29** | **2** | **564** | **60** | **8** | **78.13** | **32272092** |
| **P02538** | **30** | **18** | **22** | **2** | **564** | **60** | **8** | **63.4** | **17578638** |
| **P02533** | **43** | **16** | **21** | **8** | **472** | **51.5** | **5.16** | **51.34** | **1.04E+08** |
| **P08779** | **37** | **14** | **19** | **6** | **473** | **51.2** | **5.05** | **49.8** | **13910237** |
| **P13647** | **28** | **20** | **22** | **9** | **590** | **62.3** | **7.74** | **59.75** | **1.36E+08** |
| **P11498** | **10** | **8** | **8** | **8** | **1178** | **129.6** | **6.84** | **20.87** | **23674423** |
| **Q7Z794** | **7** | **4** | **16** | **1** | **578** | **61.9** | **5.99** | **35.9** | **3.03E+08** |
| **Q96RQ3** | **9** | **4** | **4** | **4** | **725** | **80.4** | **7.78** | **10.26** | **8865299** |
| **P60709** | **21** | **6** | **7** | **6** | **375** | **41.7** | **5.48** | **14.56** | **34752067** |
| **P13646** | **7** | **4** | **9** | **1** | **458** | **49.6** | **4.96** | **26.17** | **4658923** |
| **Q13085** | **3** | **5** | **5** | **5** | **2346** | **265.4** | **6.37** | **13.63** | **10427394** |
| **P05787** | **8** | **5** | **14** | **1** | **483** | **53.7** | **5.59** | **37.54** | **2210711** |
| **P14923** | **4** | **2** | **2** | **2** | **745** | **81.7** | **6.14** | **7.15** | **3196235** |
| **P69905** | **19** | **2** | **3** | **2** | **142** | **15.2** | **8.68** | **9.93** | **20443461** |
| **P81605** | **13** | **2** | **3** | **2** | **110** | **11.3** | **6.54** | **7.66** | **3646510** |
| **P68104** | **13** | **4** | **4** | **4** | **462** | **50.1** | **9.01** | **11.17** | **16337412** |
| **Q02413** | **5** | **3** | **3** | **3** | **1049** | **113.7** | **5.03** | **4.42** | **5903006** |
| **P05109** | **12** | **1** | **2** | **1** | **93** | **10.8** | **7.03** | **4.78** | **3256220** |
| **Q13011** | **5** | **1** | **1** | **1** | **328** | **35.8** | **8** | **3.39** | **1092044** |
| **O76009** | **5** | **2** | **2** | **2** | **404** | **45.9** | **4.82** | **4.66** | **7380088** |
| **P15924** | **1** | **2** | **2** | **2** | **2871** | **331.6** | **6.81** | **5.44** | **2350948** |
| **P25705** | **2** | **1** | **1** | **1** | **553** | **59.7** | **9.13** | **2.21** | **1600343** |
| **P07355** | **4** | **1** | **1** | **1** | **339** | **38.6** | **7.75** | **2** | **1121063** |
| **Q86YZ3** | **2** | **2** | **2** | **2** | **2850** | **282.2** | **10.04** | **5.9** | **17712790** |
| **P02768** | **4** | **2** | **2** | **2** | **609** | **69.3** | **6.28** | **5.54** | **5117245** |
| **P02788** | **3** | **2** | **2** | **2** | **710** | **78.1** | **8.12** | **1.64** | **3041294** |
| **Q08188** | **2** | **1** | **1** | **1** | **693** | **76.6** | **5.86** | **2.5** | **708847** |
| **P53597** | **4** | **1** | **1** | **1** | **346** | **36.2** | **8.79** | **4.12** | **3515495** |
| **P12273** | **8** | **1** | **1** | **1** | **146** | **16.6** | **8.05** | **3.15** | **1422663** |
| **P05165** | **2** | **1** | **1** | **1** | **728** | **80** | **7.52** | **2.52** | **1867712** |
| **Q5D862** | **0** | **1** | **1** | **1** | **2391** | **247.9** | **8.31** | **2.97** | **2748424** |
| **P02100** | **7** | **1** | **1** | **1** | **147** | **16.2** | **8.63** | **2.35** | **18712562** |
| **P31025** | **6** | **1** | **1** | **1** | **176** | **19.2** | **5.58** | **2.08** | **1711583** |
| **P01857** | **3** | **1** | **1** | **1** | **330** | **36.1** | **8.19** | **1.91** | **963138.1** |
| **P01876** | **3** | **1** | **1** | **1** | **353** | **37.6** | **6.51** | **2.15** | **1967667** |
| **Q06830** | **6** | **1** | **1** | **1** | **199** | **22.1** | **8.13** | **2.91** | **1765876** |
| **P04406** | **4** | **1** | **1** | **1** | **335** | **36** | **8.46** | **3.07** | **3531732** |
| **P13804** | **6** | **1** | **1** | **1** | **333** | **35.1** | **8.38** | **1.78** | **2350197** |

**Supplemental Table 7 Mass spectrometry profile of proteins enriched by PH-NN in lysate**

| **Accession** | **Coverage [%]** | **# Peptides** | **# PSMs** | **# Unique Peptides** | **# AAs** | **MW [kDa]** | **calc. pI** | **Score Sequest HT: Sequest HT** | **Abundances (Normalized): F4: Sample** |
| --- | --- | --- | --- | --- | --- | --- | --- | --- | --- |
| **Q86YZ3** | **2** | **1** | **1** | **1** | **2850** | **282.2** | **10.04** | **3.32** | **6219459** |
| **P49327** | **5** | **9** | **9** | **9** | **2511** | **273.3** | **6.44** | **21.75** | **16095608** |
| **Q13085** | **2** | **4** | **4** | **4** | **2346** | **265.4** | **6.37** | **4.23** | **5575879** |
| **Q04721** | **1** | **1** | **1** | **1** | **2471** | **265.2** | **5.14** | **0** | **3064910** |
| **Q5D862** | **0** | **1** | **1** | **1** | **2391** | **247.9** | **8.31** | **2.64** | **1481354** |
| **P11498** | **12** | **10** | **10** | **10** | **1178** | **129.6** | **6.84** | **26.63** | **24100113** |
| **Q02413** | **2** | **1** | **1** | **1** | **1049** | **113.7** | **5.03** | **2.28** | **1858933** |
| **P07900** | **4** | **2** | **2** | **2** | **732** | **84.6** | **5.02** | **5.31** | **3912660** |
| **Q96RQ3** | **9** | **5** | **5** | **5** | **725** | **80.4** | **7.78** | **12.57** | **10320341** |
| **P11142** | **8** | **4** | **4** | **4** | **646** | **70.9** | **5.52** | **10.19** | **5077538** |
| **P02768** | **2** | **2** | **3** | **2** | **609** | **69.3** | **6.28** | **7.01** | **55025201** |
| **P04264** | **42** | **30** | **54** | **26** | **644** | **66** | **8.12** | **177.89** | **1.7E+09** |
| **P35908** | **62** | **30** | **41** | **24** | **639** | **65.4** | **8** | **129.93** | **8.47E+08** |
| **P13647** | **22** | **12** | **16** | **5** | **590** | **62.3** | **7.74** | **47.88** | **45616893** |
| **P35527** | **50** | **20** | **31** | **20** | **623** | **62** | **5.24** | **88.57** | **4.57E+08** |
| **Q7Z794** | **5** | **3** | **6** | **1** | **578** | **61.9** | **5.99** | **16.47** | **1.58E+08** |
| **P10809** | **5** | **2** | **2** | **2** | **573** | **61** | **5.87** | **6.19** | **3674508** |
| **P02538** | **24** | **13** | **17** | **1** | **564** | **60** | **8** | **49.04** | **59177459** |
| **P48668** | **24** | **13** | **17** | **1** | **564** | **60** | **8** | **49.51** | **5852945** |
| **P25705** | **2** | **1** | **1** | **1** | **553** | **59.7** | **9.13** | **2.41** | **4232165** |
| **O95678** | **10** | **5** | **8** | **1** | **551** | **59.5** | **7.74** | **22.71** | **19591642** |
| **P13645** | **47** | **23** | **39** | **22** | **584** | **58.8** | **5.21** | **122.17** | **1.13E+09** |
| **O75449** | **2** | **1** | **1** | **1** | **491** | **55.9** | **6.9** | **2.24** | **2194495** |
| **P78386** | **21** | **9** | **11** | **4** | **507** | **55.8** | **6.55** | **32.91** | **96439431** |
| **P78385** | **23** | **9** | **11** | **1** | **493** | **54.2** | **5.64** | **32.81** | **13530419** |
| **P05787** | **13** | **6** | **10** | **3** | **483** | **53.7** | **5.59** | **28.61** | **8039336** |
| **O43790** | **22** | **8** | **11** | **1** | **486** | **53.5** | **5.66** | **34.79** | **17764556** |
| **P02533** | **37** | **14** | **14** | **6** | **472** | **51.5** | **5.16** | **39.59** | **23860538** |
| **P08779** | **34** | **14** | **14** | **6** | **473** | **51.2** | **5.05** | **37.22** | **81632692** |
| **O76015** | **4** | **2** | **2** | **1** | **456** | **50.4** | **4.84** | **4.48** | **2072742** |
| **P68104** | **15** | **5** | **6** | **5** | **462** | **50.1** | **9.01** | **17.43** | **26436205** |
| **P68363** | **12** | **4** | **4** | **4** | **451** | **50.1** | **5.06** | **10.4** | **15028806** |
| **Q13885** | **3** | **1** | **1** | **1** | **445** | **49.9** | **4.89** | **4.38** | **2322677** |
| **O76011** | **14** | **6** | **9** | **1** | **436** | **49.4** | **5.06** | **29.02** | **2701028** |
| **Q15323** | **35** | **13** | **17** | **2** | **416** | **47.2** | **4.88** | **50.3** | **1.26E+08** |
| **Q14525** | **27** | **10** | **13** | **2** | **404** | **46.2** | **4.84** | **39.08** | **9721465** |
| **O76009** | **33** | **12** | **16** | **1** | **404** | **45.9** | **4.82** | **48.54** | **4001581** |
| **P60709** | **29** | **8** | **8** | **8** | **375** | **41.7** | **5.48** | **19.34** | **49717720** |
| **P07355** | **10** | **3** | **3** | **3** | **339** | **38.6** | **7.75** | **7.85** | **9715691** |
| **P00338** | **3** | **1** | **1** | **1** | **332** | **36.7** | **8.27** | **2.21** | **1405532** |
| **P53597** | **4** | **1** | **1** | **1** | **346** | **36.2** | **8.79** | **3** | **11113178** |
| **P01857** | **3** | **1** | **1** | **1** | **330** | **36.1** | **8.19** | **2.07** | **1285074** |
| **P04406** | **4** | **1** | **1** | **1** | **335** | **36** | **8.46** | **1.99** | **1133974** |
| **Q13011** | **23** | **7** | **8** | **7** | **328** | **35.8** | **8** | **21.95** | **41105116** |
| **P13804** | **6** | **1** | **1** | **1** | **333** | **35.1** | **8.38** | **2.89** | **1115480** |
| **P45880** | **7** | **1** | **1** | **1** | **294** | **31.5** | **7.56** | **2.85** | **800686.2** |
| **Q06830** | **6** | **1** | **1** | **1** | **199** | **22.1** | **8.13** | **2.83** | **1724815** |
| **P02100** | **7** | **1** | **1** | **1** | **147** | **16.2** | **8.63** | **2.38** | **13042980** |
| **P69905** | **19** | **2** | **2** | **2** | **142** | **15.2** | **8.68** | **6.91** | **18232812** |
| **P62987** | **13** | **1** | **1** | **1** | **128** | **14.7** | **9.83** | **2.47** | **1605703** |
| **A0A087WW87** | **11** | **1** | **1** | **1** | **121** | **13.3** | **4.61** | **3.06** | **9562366** |
| **P81605** | **10** | **1** | **1** | **1** | **110** | **11.3** | **6.54** | **1.94** | **1452014** |

**Supplemental Table 8 Mass spectrometry analysis of the covalent modification site on recombinant MLKL by PTL**

| **Annotated Sequence** | **Modifications** | **# PSMs** | **Master Protein Accessions** | **Positions in Master Proteins** | **Modifications in Master Proteins** | **# Missed Cleavages** | **Theo. MH+ [Da]** | **Abundance: F2: Sample, P** | **Charge (by Search Engine): Sequest HT** | **XCorr (by Search Engine): Sequest HT** |
| --- | --- | --- | --- | --- | --- | --- | --- | --- | --- | --- |
| **[K].CMQEIPQEQIK.[E]** | **1xSHM-P [C1(100)]** | **1** | **Q8NB16** | **Q8NB16 [184-194]** | **Q8NB16 1xSHM-P [C184(100)]** | **0** | **1594.786** | **9243059** | **2** | **2.01** |
| **[K].CMQEIPQEQIK.[E]** | **1xOxidation [M2]; 1xSHM-P [C1(100)]** | **1** | **Q8NB16** | **Q8NB16 [184-194]** | **Q8NB16 1xSHM-P [C184(100)]** | **0** | **1610.78** | **2638265** | **2** | **2.01** |
| **[R].QQEPLGEDCPSELREIIDECR.[A]** | **2xCarbamidomethyl [C9; C20]** | **3** | **Q8NB16** | **Q8NB16 [429-449]** |  | **1** | **2573.161** | **79342429** | **3** | **3.35** |
| **[K].RQQEPLGEDCPSELREIIDECR.[A]** | **2xCarbamidomethyl [C10; C21]** | **3** | **Q8NB16** | **Q8NB16 [428-449]** |  | **2** | **2729.262** | **57587902** | **4** | **3.69** |
| **[R].KTQTSMSLGTTR.[E]** | **1xOxidation [M6]** | **1** | **Q8NB16** | **Q8NB16 [354-365]** |  | **1** | **1326.668** | **23645020** | **3** | **2.38** |
| **[K].TQTSMSLGTTR.[E]** | **1xOxidation [M5]** | **4** | **Q8NB16** | **Q8NB16 [355-365]** |  | **0** | **1198.573** | **8906777** | **2** | **2.32** |
| **[R].MPVSPISQGASWAQEDQQDADEDR.[R]** | **1xOxidation [M1]** | **3** | **Q8NB16** | **Q8NB16 [122-145]** |  | **0** | **2676.148** | **2262970** | **3** | **4.44** |
| **[R].MPVSPISQGASWAQEDQQDADEDRR.[A]** | **1xOxidation [M1]** | **5** | **Q8NB16** | **Q8NB16 [122-146]** |  | **1** | **2832.249** | **67118169** | **3** | **4.78** |
| **[R].MVLVLGAAR.[G]** | **1xOxidation [M1]** | **3** | **Q8NB16** | **Q8NB16 [307-315]** |  | **0** | **945.555** | **1.3E+08** | **2** | **2.3** |
| **[R].VLGLIKPLEMLQDQGK.[R]** | **1xOxidation [M10]** | **4** | **Q8NB16** | **Q8NB16 [35-50]** |  | **0** | **1798.014** | **70373596** | **3** | **3.32** |
| **[R].VLGLIKPLEMLQDQGKR.[S]** | **1xOxidation [M10]** | **5** | **Q8NB16** | **Q8NB16 [35-51]** |  | **1** | **1954.115** | **4630132** | **3** | **3.27** |
| **[R].QQEPLGEDCPSELR.[E]** | **1xCarbamidomethyl [C9]** | **3** | **Q8NB16** | **Q8NB16 [429-442]** |  | **0** | **1657.749** | **44854304** | **2** | **2.24** |
| **[K].SEIYSFGIVLWEIATGDIPFQGCNSEK.[I]** | **1xCarbamidomethyl [C23]** | **1** | **Q8NB16** | **Q8NB16 [393-419]** |  | **0** | **3060.466** |  | **2** | **3.87** |
| **[K].CMQEIPQEQIK.[E]** | **1xCarbamidomethyl [C1]; 1xOxidation [M2]** | **8** | **Q8NB16** | **Q8NB16 [184-194]** |  | **0** | **1419.661** | **50815295** | **2** | **3.16** |
| **[K].CMQEIPQEQIKEIK.[K]** | **1xCarbamidomethyl [C1]; 1xOxidation [M2]** | **7** | **Q8NB16** | **Q8NB16 [184-197]** |  | **1** | **1789.882** | **9886100** | **3** | **3.55** |
| **[K].CMQEIPQEQIK.[E]** | **1xCarbamidomethyl [C1]** | **5** | **Q8NB16** | **Q8NB16 [184-194]** |  | **0** | **1403.666** | **1.62E+08** | **2** | **3** |
| **[K].CMQEIPQEQIKEIK.[K]** | **1xCarbamidomethyl [C1]** | **6** | **Q8NB16** | **Q8NB16 [184-197]** |  | **1** | **1773.887** | **41947700** | **3** | **2.83** |
| **[K].RQQEPLGEDCPSELR.[E]** | **1xCarbamidomethyl [C10]** | **3** | **Q8NB16** | **Q8NB16 [428-442]** |  | **1** | **1813.85** | **18248120** | **3** | **3.54** |
| **[K].AALEEANGEIEK.[F]** |  | **5** | **Q8NB16** | **Q8NB16 [67-78]** |  | **0** | **1273.627** | **4.61E+08** | **2** | **3.05** |
| **[K].AALEEANGEIEKFSNR.[S]** |  | **3** | **Q8NB16** | **Q8NB16 [67-82]** |  | **1** | **1777.872** | **10792058** | **3** | **2.88** |
| **[R].AHDPSVRPSVDEILK.[K]** |  | **2** | **Q8NB16** | **Q8NB16 [450-464]** |  | **0** | **1662.881** | **3.03E+08** | **3** | **2.31** |
| **[R].AHDPSVRPSVDEILKK.[L]** |  | **1** | **Q8NB16** | **Q8NB16 [450-465]** |  | **1** | **1790.976** | **9564263** | **4** | **3.04** |
| **[R].DNEKIEASLR.[R]** |  | **4** | **Q8NB16** | **Q8NB16 [154-163]** |  | **1** | **1174.606** | **41365548** | **2** | **2.7** |
| **[R].ELLDREK.[D]** |  | **1** | **Q8NB16** | **Q8NB16 [293-299]** |  | **1** | **902.4942** | **1.05E+08** | **2** | **2.06** |
| **[K].ELSLLLQVEQR.[M]** |  | **15** | **Q8NB16** | **Q8NB16 [111-121]** |  | **0** | **1327.758** | **2.67E+08** | **2** | **2.68** |
| **[R].ENEVSTLYK.[G]** |  | **7** | **Q8NB16** | **Q8NB16 [211-219]** |  | **0** | **1082.536** | **3.91E+08** | **2** | **2.34** |
| **[R].ENEVSTLYKGEYHR.[A]** |  | **3** | **Q8NB16** | **Q8NB16 [211-224]** |  | **1** | **1724.824** | **10663718** | **2** | **3.02** |
| **[K].EQLSGSPWILLR.[E]** |  | **3** | **Q8NB16** | **Q8NB16 [199-210]** |  | **0** | **1398.774** | **1.48E+08** | **2** | **2.29** |
| **[K].EQLSGSPWILLRENEVSTLYK.[G]** |  | **1** | **Q8NB16** | **Q8NB16 [199-219]** |  | **1** | **2462.293** | **2783683** | **3** | **3.34** |
| **[K].EQLSGSPWILLRENEVSTLYKGEYHR.[A]** |  | **1** | **Q8NB16** | **Q8NB16 [199-224]** |  | **2** | **3104.58** |  | **4** | **3.57** |
| **[K].FESPNILR.[I]** |  | **1** | **Q8NB16** | **Q8NB16 [257-264]** |  | **0** | **975.5258** | **1.01E+08** | **2** | **1.92** |
| **[R].FKAALEEANGEIEK.[F]** |  | **2** | **Q8NB16** | **Q8NB16 [65-78]** |  | **1** | **1548.79** | **32094081** | **2** | **3.55** |
| **[R].FLTASQDKILFK.[D]** |  | **2** | **Q8NB16** | **Q8NB16 [88-99]** |  | **1** | **1410.799** | **6105738** | **3** | **2.84** |
| **[R].FLTASQDKILFKDVNR.[K]** |  | **1** | **Q8NB16** | **Q8NB16 [88-103]** |  | **2** | **1895.039** | **1444827** | **3** | **2.62** |
| **[K].HIITLGQVIHK.[R]** |  | **6** | **Q8NB16** | **Q8NB16 [6-16]** |  | **0** | **1258.763** | **62264128** | **3** | **3.85** |
| **[K].KEQLSGSPWILLR.[E]** |  | **3** | **Q8NB16** | **Q8NB16 [198-210]** |  | **1** | **1526.869** | **1.28E+08** | **3** | **2.83** |
| **[K].KEQLSGSPWILLRENEVSTLYK.[G]** |  | **1** | **Q8NB16** | **Q8NB16 [198-219]** |  | **2** | **2590.388** | **5457982** | **3** | **4.51** |
| **[K].KFESPNILR.[I]** |  | **5** | **Q8NB16** | **Q8NB16 [256-264]** |  | **1** | **1103.621** | **2.84E+08** | **2** | **2.53** |
| **[K].KLQAGSIAIVR.[Q]** |  | **6** | **Q8NB16** | **Q8NB16 [234-244]** |  | **1** | **1155.721** | **3.64E+08** | **2** | **3.61** |
| **[R].KLSDVWK.[E]** |  | **6** | **Q8NB16** | **Q8NB16 [104-110]** |  | **1** | **875.4985** | **2.64E+08** | **2** | **2.7** |
| **[K].KLSTFSK.[-]** |  | **4** | **Q8NB16** | **Q8NB16 [465-471]** |  | **1** | **810.472** | **1.86E+08** | **2** | **2.48** |
| **[R].KTQTSMSLGTTR.[E]** |  | **3** | **Q8NB16** | **Q8NB16 [354-365]** |  | **1** | **1310.673** | **2.59E+08** | **3** | **2.99** |
| **[K].LAGFELR.[K]** |  | **3** | **Q8NB16** | **Q8NB16 [347-353]** |  | **0** | **805.4567** | **5.24E+08** | **2** | **2.23** |
| **[R].LEINMKEIK.[E]** |  | **1** | **Q8NB16** | **Q8NB16 [165-173]** |  | **1** | **1117.629** | **460428.8** | **2** | **2.41** |
| **[R].LHHSEAPELHGK.[I]** |  | **3** | **Q8NB16** | **Q8NB16 [320-331]** |  | **0** | **1354.686** | **99500256** | **3** | **4.53** |
| **[K].LQAGSIAIVR.[Q]** |  | **2** | **Q8NB16** | **Q8NB16 [235-244]** |  | **0** | **1027.626** | **3.86E+08** | **2** | **2.33** |
| **[K].LTTAMNR.[F]** |  | **1** | **Q8NB16** | **Q8NB16 [58-64]** |  | **0** | **806.4189** | **3.09E+08** | **2** | **2.09** |
| **[R].MPVSPISQGASWAQEDQQDADEDR.[R]** |  | **3** | **Q8NB16** | **Q8NB16 [122-145]** |  | **0** | **2660.153** | **8968832** | **3** | **5.4** |
| **[R].MPVSPISQGASWAQEDQQDADEDRR.[A]** |  | **9** | **Q8NB16** | **Q8NB16 [122-146]** |  | **1** | **2816.254** | **1.79E+08** | **3** | **4.9** |
| **[R].MPVSPISQGASWAQEDQQDADEDRRAFQMLR.[R]** |  | **1** | **Q8NB16** | **Q8NB16 [122-152]** |  | **2** | **3562.644** | **3597761** | **4** | **3.85** |
| **[R].MVLVLGAAR.[G]** |  | **9** | **Q8NB16** | **Q8NB16 [307-315]** |  | **0** | **929.5601** | **6.7E+08** | **2** | **2.87** |
| **[R].QYLPPK.[C]** |  | **6** | **Q8NB16** | **Q8NB16 [178-183]** |  | **0** | **745.4243** | **1.01E+08** | **2** | **2.03** |
| **[R].RAFQMLR.[R]** |  | **4** | **Q8NB16** | **Q8NB16 [146-152]** |  | **1** | **921.5087** | **37978824** | **2** | **2.7** |
| **[R].RDNEKIEASLR.[R]** |  | **3** | **Q8NB16** | **Q8NB16 [153-163]** |  | **2** | **1330.707** | **67232978** | **2** | **2.96** |
| **[R].RLEINMK.[E]** |  | **9** | **Q8NB16** | **Q8NB16 [164-170]** |  | **1** | **903.5081** | **5.31E+08** | **2** | **2.34** |
| **[R].RLEINMKEIK.[E]** |  | **1** | **Q8NB16** | **Q8NB16 [164-173]** |  | **2** | **1273.73** | **2841020** | **3** | **2.62** |
| **[K].RMVLVLGAAR.[G]** |  | **3** | **Q8NB16** | **Q8NB16 [306-315]** |  | **1** | **1085.661** | **33736620** | **2** | **2.58** |
| **[K].RSVPSEK.[L]** |  | **1** | **Q8NB16** | **Q8NB16 [51-57]** |  | **1** | **802.4417** | **11016188** | **2** | **2.04** |
| **[R].SSNFLVTQGYQVK.[L]** |  | **12** | **Q8NB16** | **Q8NB16 [334-346]** |  | **0** | **1470.759** | **6.91E+08** | **2** | **3.53** |
| **[R].SSNFLVTQGYQVKLAGFELR.[K]** |  | **1** | **Q8NB16** | **Q8NB16 [334-353]** |  | **1** | **2257.198** | **10438391** | **3** | **5.27** |
| **[R].SSNFLVTQGYQVKLAGFELRK.[T]** |  | **2** | **Q8NB16** | **Q8NB16 [334-354]** |  | **2** | **2385.293** | **1362464** | **3** | **3.38** |
| **[K].STAYLSPQELEDVFYQYDVK.[S]** |  | **4** | **Q8NB16** | **Q8NB16 [373-392]** |  | **0** | **2395.134** | **1.84E+08** | **2** | **4.05** |
| **[K].TQTSMSLGTTR.[E]** |  | **2** | **Q8NB16** | **Q8NB16 [355-365]** |  | **0** | **1182.578** | **3.63E+08** | **2** | **2.56** |
| **[R].VKSTAYLSPQELEDVFYQYDVK.[S]** |  | **1** | **Q8NB16** | **Q8NB16 [371-392]** |  | **1** | **2622.297** | **2277842** | **3** | **2.74** |
| **[R].VLGLIKPLEMLQDQGK.[R]** |  | **4** | **Q8NB16** | **Q8NB16 [35-50]** |  | **0** | **1782.019** | **1.24E+08** | **3** | **4.25** |
| **[R].VLGLIKPLEMLQDQGKR.[S]** |  | **3** | **Q8NB16** | **Q8NB16 [35-51]** |  | **1** | **1938.12** | **28833849** | **3** | **4.7** |

**Supplemental Table 9 Mass spectrometry analysis of the covalent modification site on recombinant MLKL by PTL-11**

| **Annotated Sequence** | **Modifications** | **# PSMs** | **Master Protein Accessions** | **Positions in Master Proteins** | **Modifications in Master Proteins** | **# Missed Cleavages** | **Theo. MH+ [Da]** | **Abundance: F2: Sample, P11** | **Charge (by Search Engine): Sequest HT** | **XCorr (by Search Engine): Sequest HT** |
| --- | --- | --- | --- | --- | --- | --- | --- | --- | --- | --- |
| **[K].CMQEIPQEQIK.[E]** | **1xSHM_P11 [C1(100)]** | **1** | **Q8NB16** | **Q8NB16 [184-194]** | **Q8NB16 1xSHM_P11 [C184(100)]** | **0** | **1762.828** | **1646438** | **2** | **2.43** |
| **[K].CMQEIPQEQIKEIK.[K]** | **1xSHM_P11 [C1(100)]** | **1** | **Q8NB16** | **Q8NB16 [184-197]** | **Q8NB16 1xSHM_P11 [C184(100)]** | **1** | **2133.049** | **3548798** | **3** | **3.48** |
| **[R].MPVSPISQGASWAQEDQQDADEDRRAFQMLR.[R]** | **2xOxidation [M1; M29]** | **2** | **Q8NB16** | **Q8NB16 [122-152]** |  | **2** | **3594.634** | **2172270** | **4** | **3.88** |
| **[R].QQEPLGEDCPSELREIIDECR.[A]** | **2xCarbamidomethyl [C9; C20]** | **3** | **Q8NB16** | **Q8NB16 [429-449]** |  | **1** | **2573.161** | **57118843** | **3** | **3.48** |
| **[R].QQEPLGEDCPSELREIIDECRAHDPSVRPSVDEILK.[K]** | **2xCarbamidomethyl [C9; C20]** | **1** | **Q8NB16** | **Q8NB16 [429-464]** |  | **2** | **4217.024** | **2594195** | **5** | **2.96** |
| **[K].RQQEPLGEDCPSELREIIDECR.[A]** | **2xCarbamidomethyl [C10; C21]** | **2** | **Q8NB16** | **Q8NB16 [428-449]** |  | **2** | **2729.262** | **23507024** | **4** | **5.07** |
| **[R].MPVSPISQGASWAQEDQQDADEDRRAFQMLR.[R]** | **1xOxidation [M]** | **3** | **Q8NB16** | **Q8NB16 [122-152]** |  | **2** | **3578.639** | **14844888** | **4** | **4.86** |
| **[K].TQTSMSLGTTR.[E]** | **1xOxidation [M5]** | **3** | **Q8NB16** | **Q8NB16 [355-365]** |  | **0** | **1198.573** | **17389288** | **2** | **2.53** |
| **[R].MPVSPISQGASWAQEDQQDADEDR.[R]** | **1xOxidation [M1]** | **1** | **Q8NB16** | **Q8NB16 [122-145]** |  | **0** | **2676.148** | **862589.2** | **3** | **3.78** |
| **[R].MPVSPISQGASWAQEDQQDADEDRR.[A]** | **1xOxidation [M1]** | **7** | **Q8NB16** | **Q8NB16 [122-146]** |  | **1** | **2832.249** | **51502300** | **3** | **4.78** |
| **[R].MVLVLGAAR.[G]** | **1xOxidation [M1]** | **4** | **Q8NB16** | **Q8NB16 [307-315]** |  | **0** | **945.555** | **74422690** | **2** | **2.25** |
| **[R].VLGLIKPLEMLQDQGK.[R]** | **1xOxidation [M10]** | **6** | **Q8NB16** | **Q8NB16 [35-50]** |  | **0** | **1798.014** | **41406027** | **3** | **3.07** |
| **[R].VLGLIKPLEMLQDQGKR.[S]** | **1xOxidation [M10]** | **7** | **Q8NB16** | **Q8NB16 [35-51]** |  | **1** | **1954.115** | **15070757** | **3** | **4.83** |
| **[R].QQEPLGEDCPSELR.[E]** | **1xCarbamidomethyl [C9]** | **3** | **Q8NB16** | **Q8NB16 [429-442]** |  | **0** | **1657.749** | **10684613** | **2** | **2.46** |
| **[R].EIIDECR.[A]** | **1xCarbamidomethyl [C6]** | **1** | **Q8NB16** | **Q8NB16 [443-449]** |  | **0** | **934.4299** | **13438199** | **2** | **1.99** |
| **[K].CMQEIPQEQIK.[E]** | **1xCarbamidomethyl [C1]; 1xOxidation [M2]** | **5** | **Q8NB16** | **Q8NB16 [184-194]** |  | **0** | **1419.661** | **14225962** | **2** | **3.21** |
| **[K].CMQEIPQEQIKEIK.[K]** | **1xCarbamidomethyl [C1]; 1xOxidation [M2]** | **8** | **Q8NB16** | **Q8NB16 [184-197]** |  | **1** | **1789.882** | **11006950** | **3** | **3.56** |
| **[K].CMQEIPQEQIK.[E]** | **1xCarbamidomethyl [C1]** | **3** | **Q8NB16** | **Q8NB16 [184-194]** |  | **0** | **1403.666** | **54366990** | **2** | **2.68** |
| **[K].CMQEIPQEQIKEIK.[K]** | **1xCarbamidomethyl [C1]** | **4** | **Q8NB16** | **Q8NB16 [184-197]** |  | **1** | **1773.887** | **55863025** | **3** | **3.22** |
| **[K].RQQEPLGEDCPSELR.[E]** | **1xCarbamidomethyl [C10]** | **2** | **Q8NB16** | **Q8NB16 [428-442]** |  | **1** | **1813.85** | **6795182** | **3** | **3.75** |
| **[K].AALEEANGEIEK.[F]** |  | **6** | **Q8NB16** | **Q8NB16 [67-78]** |  | **0** | **1273.627** | **1.72E+08** | **2** | **3.24** |
| **[K].AALEEANGEIEKFSNR.[S]** |  | **3** | **Q8NB16** | **Q8NB16 [67-82]** |  | **1** | **1777.872** | **37171048** | **3** | **4.13** |
| **[R].AHDPSVRPSVDEILK.[K]** |  | **2** | **Q8NB16** | **Q8NB16 [450-464]** |  | **0** | **1662.881** | **1.82E+08** | **3** | **2.55** |
| **[R].AHDPSVRPSVDEILKK.[L]** |  | **3** | **Q8NB16** | **Q8NB16 [450-465]** |  | **1** | **1790.976** | **27386680** | **4** | **4.05** |
| **[R].DNEKIEASLR.[R]** |  | **2** | **Q8NB16** | **Q8NB16 [154-163]** |  | **1** | **1174.606** | **90721156** | **2** | **2.61** |
| **[R].ELLDREKDLTLGK.[R]** |  | **3** | **Q8NB16** | **Q8NB16 [293-305]** |  | **2** | **1529.853** | **50719430** | **3** | **2.5** |
| **[K].ELSLLLQVEQR.[M]** |  | **11** | **Q8NB16** | **Q8NB16 [111-121]** |  | **0** | **1327.758** | **65090748** | **2** | **2.76** |
| **[R].ENEVSTLYK.[G]** |  | **3** | **Q8NB16** | **Q8NB16 [211-219]** |  | **0** | **1082.536** | **1.73E+08** | **2** | **1.96** |
| **[R].ENEVSTLYKGEYHR.[A]** |  | **3** | **Q8NB16** | **Q8NB16 [211-224]** |  | **1** | **1724.824** | **35075113** | **4** | **3.42** |
| **[K].EQLSGSPWILLR.[E]** |  | **2** | **Q8NB16** | **Q8NB16 [199-210]** |  | **0** | **1398.774** | **51581956** | **2** | **2.45** |
| **[K].EQLSGSPWILLRENEVSTLYK.[G]** |  | **2** | **Q8NB16** | **Q8NB16 [199-219]** |  | **1** | **2462.293** | **12127325** | **3** | **4.43** |
| **[K].EQLSGSPWILLRENEVSTLYKGEYHR.[A]** |  | **1** | **Q8NB16** | **Q8NB16 [199-224]** |  | **2** | **3104.58** |  | **4** | **4.77** |
| **[R].FKAALEEANGEIEK.[F]** |  | **2** | **Q8NB16** | **Q8NB16 [65-78]** |  | **1** | **1548.79** | **50409250** | **2** | **4.05** |
| **[R].FKAALEEANGEIEKFSNR.[S]** |  | **1** | **Q8NB16** | **Q8NB16 [65-82]** |  | **2** | **2053.035** | **4071935** | **3** | **5.21** |
| **[R].FLTASQDK.[I]** |  | **1** | **Q8NB16** | **Q8NB16 [88-95]** |  | **0** | **909.4676** | **4586709** | **2** | **2.07** |
| **[R].FLTASQDKILFK.[D]** |  | **1** | **Q8NB16** | **Q8NB16 [88-99]** |  | **1** | **1410.799** | **4761295** | **3** | **3.04** |
| **[K].HIITLGQVIHK.[R]** |  | **7** | **Q8NB16** | **Q8NB16 [6-16]** |  | **0** | **1258.763** | **56145192** | **3** | **3.76** |
| **[K].HIITLGQVIHKR.[C]** |  | **1** | **Q8NB16** | **Q8NB16 [6-17]** |  | **1** | **1414.864** | **1932075** | **3** | **4.09** |
| **[K].KEQLSGSPWILLR.[E]** |  | **3** | **Q8NB16** | **Q8NB16 [198-210]** |  | **1** | **1526.869** | **78519232** | **3** | **3.37** |
| **[K].KEQLSGSPWILLRENEVSTLYK.[G]** |  | **1** | **Q8NB16** | **Q8NB16 [198-219]** |  | **2** | **2590.388** | **19843958** | **3** | **4.47** |
| **[K].KFESPNILR.[I]** |  | **7** | **Q8NB16** | **Q8NB16 [256-264]** |  | **1** | **1103.621** | **1.36E+08** | **2** | **3.28** |
| **[K].KLQAGSIAIVR.[Q]** |  | **5** | **Q8NB16** | **Q8NB16 [234-244]** |  | **1** | **1155.721** | **1.23E+08** | **2** | **3.43** |
| **[R].KLSDVWK.[E]** |  | **4** | **Q8NB16** | **Q8NB16 [104-110]** |  | **1** | **875.4985** | **1.29E+08** | **2** | **2.75** |
| **[K].KLSTFSK.[-]** |  | **3** | **Q8NB16** | **Q8NB16 [465-471]** |  | **1** | **810.472** | **1.46E+08** | **2** | **2.47** |
| **[R].KTQTSMSLGTTR.[E]** |  | **2** | **Q8NB16** | **Q8NB16 [354-365]** |  | **1** | **1310.673** | **1.1E+08** | **3** | **2.52** |
| **[K].LAGFELR.[K]** |  | **4** | **Q8NB16** | **Q8NB16 [347-353]** |  | **0** | **805.4567** | **2.78E+08** | **2** | **2.13** |
| **[R].LHHSEAPELHGK.[I]** |  | **4** | **Q8NB16** | **Q8NB16 [320-331]** |  | **0** | **1354.686** | **49513620** | **3** | **3.55** |
| **[K].LQAGSIAIVR.[Q]** |  | **4** | **Q8NB16** | **Q8NB16 [235-244]** |  | **0** | **1027.626** | **1.89E+08** | **2** | **2.52** |
| **[K].IRSSNFLVTQGYQVKLAGFELR.[K]** |  | **1** | **Q8NB16** | **Q8NB16 [332-353]** |  | **2** | **2526.383** | **337104.9** | **4** | **3.91** |
| **[K].LTTAMNR.[F]** |  | **1** | **Q8NB16** | **Q8NB16 [58-64]** |  | **0** | **806.4189** | **2.74E+08** | **2** | **1.92** |
| **[R].MPVSPISQGASWAQEDQQDADEDR.[R]** |  | **2** | **Q8NB16** | **Q8NB16 [122-145]** |  | **0** | **2660.153** | **3119038** | **3** | **4.69** |
| **[R].MPVSPISQGASWAQEDQQDADEDRR.[A]** |  | **7** | **Q8NB16** | **Q8NB16 [122-146]** |  | **1** | **2816.254** | **1.26E+08** | **3** | **4.45** |
| **[R].MPVSPISQGASWAQEDQQDADEDRRAFQMLR.[R]** |  | **2** | **Q8NB16** | **Q8NB16 [122-152]** |  | **2** | **3562.644** | **26425782** | **4** | **4.2** |
| **[R].MVLVLGAAR.[G]** |  | **7** | **Q8NB16** | **Q8NB16 [307-315]** |  | **0** | **929.5601** | **2.89E+08** | **2** | **2.66** |
| **[R].QTFNKEIK.[T]** |  | **2** | **Q8NB16** | **Q8NB16 [245-252]** |  | **1** | **1007.552** | **26954194** | **2** | **2.38** |
| **[R].QYLPPK.[C]** |  | **2** | **Q8NB16** | **Q8NB16 [178-183]** |  | **0** | **745.4243** | **27286923** | **2** | **2.05** |
| **[R].RAFQMLR.[R]** |  | **3** | **Q8NB16** | **Q8NB16 [146-152]** |  | **1** | **921.5087** | **20133532** | **2** | **2.45** |
| **[R].RDNEKIEASLR.[R]** |  | **4** | **Q8NB16** | **Q8NB16 [153-163]** |  | **2** | **1330.707** | **2.08E+08** | **3** | **3.05** |
| **[R].RLEINMK.[E]** |  | **4** | **Q8NB16** | **Q8NB16 [164-170]** |  | **1** | **903.5081** | **3.5E+08** | **2** | **2.31** |
| **[R].RLEINMKEIK.[E]** |  | **1** | **Q8NB16** | **Q8NB16 [164-173]** |  | **2** | **1273.73** | **5874859** | **3** | **2.86** |
| **[K].RMVLVLGAAR.[G]** |  | **3** | **Q8NB16** | **Q8NB16 [306-315]** |  | **1** | **1085.661** | **27810736** | **2** | **2.52** |
| **[R].SSNFLVTQGYQVK.[L]** |  | **9** | **Q8NB16** | **Q8NB16 [334-346]** |  | **0** | **1470.759** | **4.05E+08** | **2** | **3.58** |
| **[R].SSNFLVTQGYQVKLAGFELR.[K]** |  | **1** | **Q8NB16** | **Q8NB16 [334-353]** |  | **1** | **2257.198** |  | **3** | **5.43** |
| **[K].STAYLSPQELEDVFYQYDVK.[S]** |  | **3** | **Q8NB16** | **Q8NB16 [373-392]** |  | **0** | **2395.134** | **94532544** | **2** | **4.76** |
| **[R].SVPSEKLTTAMNR.[F]** |  | **1** | **Q8NB16** | **Q8NB16 [52-64]** |  | **1** | **1433.742** | **1209589** | **2** | **1.95** |
| **[K].TQTSMSLGTTR.[E]** |  | **3** | **Q8NB16** | **Q8NB16 [355-365]** |  | **0** | **1182.578** | **1.55E+08** | **2** | **2.45** |
| **[R].VKSTAYLSPQELEDVFYQYDVK.[S]** |  | **1** | **Q8NB16** | **Q8NB16 [371-392]** |  | **1** | **2622.297** | **3902537** | **3** | **4.64** |
| **[R].VLGLIKPLEMLQDQGK.[R]** |  | **3** | **Q8NB16** | **Q8NB16 [35-50]** |  | **0** | **1782.019** | **78620360** | **2** | **4.25** |
| **[R].VLGLIKPLEMLQDQGKR.[S]** |  | **5** | **Q8NB16** | **Q8NB16 [35-51]** |  | **1** | **1938.12** | **88763132** | **3** | **6.21** |

**Supplemental Table 10 Mass spectrometry analysis of the covalent modification site on recombinant MLKL by CTL**

| **Annotated Sequence** | **Modifications** | **# PSMs** | **Master Protein Accessions** | **Positions in Master Proteins** | **Modifications in Master Proteins** | **# Missed Cleavages** | **Theo. MH+ [Da]** | **Abundance: F2: Sample, CTL** | **Charge (by Search Engine): Sequest HT** | **XCorr (by Search Engine): Sequest HT** |
| --- | --- | --- | --- | --- | --- | --- | --- | --- | --- | --- |
| **[K].CMQEIPQEQIK.[E]** | **1xOxidation [M2]; 1xSHM_CTL [C1(100)]** | **1** | **Q8NB16** | **Q8NB16 [184-194]** | **Q8NB16 1xSHM_CTL [C184(100)]** | **0** | **1594.7855** | **2436489** | **2** | **2.53** |
| **[R].KTQTSMSLGTTR.[E]** | **1xOxidation [M6]** | **2** | **Q8NB16** | **Q8NB16 [354-365]** |  | **1** | **1326.668** | **16346961** | **3** | **3** |
| **[K].TQTSMSLGTTR.[E]** | **1xOxidation [M5]** | **6** | **Q8NB16** | **Q8NB16 [355-365]** |  | **0** | **1198.573** | **5.02E+08** | **2** | **2.43** |
| **[R].MVLVLGAAR.[G]** | **1xOxidation [M1]** | **12** | **Q8NB16** | **Q8NB16 [307-315]** |  | **0** | **945.555** | **7.18E+08** | **2** | **2.4** |
| **[R].VLGLIKPLEMLQDQGK.[R]** | **1xOxidation [M10]** | **5** | **Q8NB16** | **Q8NB16 [35-50]** |  | **0** | **1798.014** | **52748254** | **3** | **3.31** |
| **[R].QQEPLGEDCPSELR.[E]** | **1xCarbamidomethyl [C9]** | **2** | **Q8NB16** | **Q8NB16 [429-442]** |  | **0** | **1657.749** | **1.13E+08** | **2** | **2.81** |
| **[R].EIIDECR.[A]** | **1xCarbamidomethyl [C6]** | **1** | **Q8NB16** | **Q8NB16 [443-449]** |  | **0** | **934.4299** | **2.41E+08** | **2** | **2.01** |
| **[K].CMQEIPQEQIK.[E]** | **1xCarbamidomethyl [C1]; 1xOxidation [M2]** | **8** | **Q8NB16** | **Q8NB16 [184-194]** |  | **0** | **1419.661** | **89977285** | **2** | **3.37** |
| **[K].CMQEIPQEQIK.[E]** | **1xCarbamidomethyl [C1]** | **6** | **Q8NB16** | **Q8NB16 [184-194]** |  | **0** | **1403.666** | **1.49E+08** | **2** | **2.69** |
| **[K].RQQEPLGEDCPSELR.[E]** | **1xCarbamidomethyl [C10]** | **3** | **Q8NB16** | **Q8NB16 [428-442]** |  | **1** | **1813.85** | **30230162** | **3** | **4.12** |
| **[K].AALEEANGEIEK.[F]** |  | **13** | **Q8NB16** | **Q8NB16 [67-78]** |  | **0** | **1273.627** | **2.42E+08** | **2** | **3.33** |
| **[R].AHDPSVRPSVDEILK.[K]** |  | **1** | **Q8NB16** | **Q8NB16 [450-464]** |  | **0** | **1662.881** | **3.88E+08** | **3** | **2.31** |
| **[R].AHDPSVRPSVDEILKK.[L]** |  | **1** | **Q8NB16** | **Q8NB16 [450-465]** |  | **1** | **1790.976** | **1598364** | **4** | **2.86** |
| **[R].APVAIKVFK.[K]** |  | **2** | **Q8NB16** | **Q8NB16 [225-233]** |  | **1** | **972.6241** | **10789240** | **2** | **2.71** |
| **[R].APVAIKVFKK.[L]** |  | **1** | **Q8NB16** | **Q8NB16 [225-234]** |  | **2** | **1100.719** | **683989.9** | **3** | **2.37** |
| **[R].EKDLTLGK.[R]** |  | **1** | **Q8NB16** | **Q8NB16 [298-305]** |  | **1** | **903.5146** | **79314880** | **2** | **1.95** |
| **[R].ELLDREKDLTLGK.[R]** |  | **1** | **Q8NB16** | **Q8NB16 [293-305]** |  | **2** | **1529.853** | **2188024** | **3** | **2.38** |
| **[K].ELSLLLQVEQR.[M]** |  | **13** | **Q8NB16** | **Q8NB16 [111-121]** |  | **0** | **1327.758** | **71150907** | **2** | **3** |
| **[R].ENEVSTLYK.[G]** |  | **10** | **Q8NB16** | **Q8NB16 [211-219]** |  | **0** | **1082.536** | **8.6E+08** | **2** | **2.49** |
| **[R].ENEVSTLYKGEYHR.[A]** |  | **2** | **Q8NB16** | **Q8NB16 [211-224]** |  | **1** | **1724.824** | **6696245** | **3** | **4.2** |
| **[K].EQLSGSPWILLR.[E]** |  | **4** | **Q8NB16** | **Q8NB16 [199-210]** |  | **0** | **1398.774** | **4.14E+08** | **2** | **2.54** |
| **[K].FESPNILR.[I]** |  | **4** | **Q8NB16** | **Q8NB16 [257-264]** |  | **0** | **975.5258** | **3.79E+08** | **2** | **2.13** |
| **[R].FLTASQDK.[I]** |  | **4** | **Q8NB16** | **Q8NB16 [88-95]** |  | **0** | **909.4676** | **1.03E+09** | **2** | **2.34** |
| **[R].FLTASQDKILFK.[D]** |  | **2** | **Q8NB16** | **Q8NB16 [88-99]** |  | **1** | **1410.799** | **11539745** | **3** | **2.8** |
| **[K].HIITLGQVIHK.[R]** |  | **4** | **Q8NB16** | **Q8NB16 [6-16]** |  | **0** | **1258.763** | **1.34E+08** | **3** | **3.91** |
| **[K].KEQLSGSPWILLR.[E]** |  | **3** | **Q8NB16** | **Q8NB16 [198-210]** |  | **1** | **1526.869** | **1.26E+08** | **2** | **4.63** |
| **[K].KFESPNILR.[I]** |  | **7** | **Q8NB16** | **Q8NB16 [256-264]** |  | **1** | **1103.621** | **4.42E+08** | **2** | **2.63** |
| **[K].KLQAGSIAIVR.[Q]** |  | **3** | **Q8NB16** | **Q8NB16 [234-244]** |  | **1** | **1155.721** | **1.12E+08** | **3** | **3.18** |
| **[R].KLSDVWK.[E]** |  | **14** | **Q8NB16** | **Q8NB16 [104-110]** |  | **1** | **875.4985** | **4.41E+08** | **2** | **2.91** |
| **[K].KLSTFSK.[-]** |  | **6** | **Q8NB16** | **Q8NB16 [465-471]** |  | **1** | **810.472** | **86979910** | **2** | **2.52** |
| **[R].KTQTSMSLGTTR.[E]** |  | **3** | **Q8NB16** | **Q8NB16 [354-365]** |  | **1** | **1310.673** | **45132116** | **3** | **2.9** |
| **[K].LAGFELR.[K]** |  | **3** | **Q8NB16** | **Q8NB16 [347-353]** |  | **0** | **805.4567** | **1.34E+09** | **2** | **2.15** |
| **[R].LEINMKEIK.[E]** |  | **1** | **Q8NB16** | **Q8NB16 [165-173]** |  | **1** | **1117.629** | **1093006** | **2** | **1.96** |
| **[R].LHHSEAPELHGK.[I]** |  | **3** | **Q8NB16** | **Q8NB16 [320-331]** |  | **0** | **1354.686** | **93360576** | **3** | **4.08** |
| **[R].LHHSEAPELHGKIR.[S]** |  | **1** | **Q8NB16** | **Q8NB16 [320-333]** |  | **1** | **1623.871** | **1855268** | **4** | **3.48** |
| **[K].LQAGSIAIVR.[Q]** |  | **5** | **Q8NB16** | **Q8NB16 [235-244]** |  | **0** | **1027.626** | **1.57E+09** | **2** | **2.57** |
| **[K].IRSSNFLVTQGYQVK.[L]** |  | **1** | **Q8NB16** | **Q8NB16 [332-346]** |  | **1** | **1739.944** | **1213699** | **3** | **2.38** |
| **[K].LTTAMNR.[F]** |  | **1** | **Q8NB16** | **Q8NB16 [58-64]** |  | **0** | **806.4189** | **5660281** | **2** | **1.93** |
| **[R].MVLVLGAAR.[G]** |  | **9** | **Q8NB16** | **Q8NB16 [307-315]** |  | **0** | **929.5601** | **1.35E+09** | **2** | **2.7** |
| **[R].QTFNKEIK.[T]** |  | **1** | **Q8NB16** | **Q8NB16 [245-252]** |  | **1** | **1007.552** | **7223632** | **2** | **2.13** |
| **[R].QYLPPK.[C]** |  | **4** | **Q8NB16** | **Q8NB16 [178-183]** |  | **0** | **745.4243** | **1.38E+08** | **2** | **2.05** |
| **[R].RAFQMLR.[R]** |  | **1** | **Q8NB16** | **Q8NB16 [146-152]** |  | **1** | **921.5087** | **4534981** | **2** | **2.17** |
| **[R].RLEINMK.[E]** |  | **11** | **Q8NB16** | **Q8NB16 [164-170]** |  | **1** | **903.5081** | **5.2E+08** | **2** | **2.55** |
| **[R].RLEINMKEIK.[E]** |  | **2** | **Q8NB16** | **Q8NB16 [164-173]** |  | **2** | **1273.73** | **2178458** | **3** | **3.83** |
| **[K].RMVLVLGAAR.[G]** |  | **2** | **Q8NB16** | **Q8NB16 [306-315]** |  | **1** | **1085.661** | **1928203** | **2** | **2.46** |
| **[K].RSVPSEK.[L]** |  | **1** | **Q8NB16** | **Q8NB16 [51-57]** |  | **1** | **802.4417** | **4169240** | **2** | **1.98** |
| **[R].SSNFLVTQGYQVK.[L]** |  | **11** | **Q8NB16** | **Q8NB16 [334-346]** |  | **0** | **1470.759** | **1.28E+09** | **2** | **3.25** |
| **[R].SSNFLVTQGYQVKLAGFELR.[K]** |  | **1** | **Q8NB16** | **Q8NB16 [334-353]** |  | **1** | **2257.198** | **5004587** | **3** | **5.04** |
| **[R].SSNFLVTQGYQVKLAGFELRK.[T]** |  | **1** | **Q8NB16** | **Q8NB16 [334-354]** |  | **2** | **2385.293** | **1408382** | **4** | **4.27** |
| **[K].STAYLSPQELEDVFYQYDVK.[S]** |  | **3** | **Q8NB16** | **Q8NB16 [373-392]** |  | **0** | **2395.134** | **45262304** | **2** | **4.37** |
| **[K].TQTSMSLGTTR.[E]** |  | **1** | **Q8NB16** | **Q8NB16 [355-365]** |  | **0** | **1182.578** | **9.46E+08** | **2** | **2.83** |
| **[R].VLGLIKPLEMLQDQGK.[R]** |  | **3** | **Q8NB16** | **Q8NB16 [35-50]** |  | **0** | **1782.019** | **40786888** | **3** | **3.38** |

**Supplemental Table 11 Mass spectrometry analysis of the covalent modification site on recombinant MLKL by DDL**

| **Annotated Sequence** | **Modifications** | **# PSMs** | **Master Protein Accessions** | **Positions in Master Proteins** | **Modifications in Master Proteins** | **# Missed Cleavages** | **Theo. MH+ [Da]** | **Abundance: F2: Sample, DDL** | **Charge (by Search Engine): Sequest HT** | **XCorr (by Search Engine): Sequest HT** |
| --- | --- | --- | --- | --- | --- | --- | --- | --- | --- | --- |
| **[R].QQEPLGEDCPSELR.[E]** | **1xSHM_DDL [C9(100)]** | **1** | **Q8NB16** | **Q8NB16 [429-442]** | **Q8NB16 1xSHM_DDL [C437(100)]** | **0** | **1830.8579** | **26550078** | **2** | **2.71** |
| **[K].RQQEPLGEDCPSELR.[E]** | **1xSHM_DDL [C10(100)]** | **2** | **Q8NB16** | **Q8NB16 [428-442]** | **Q8NB16 1xSHM_DDL [C437(100)]** | **1** | **1986.959** | **6534180.5** | **3** | **3.98** |
| **[K].CMQEIPQEQIK.[E]** | **1xSHM_DDL [C1(100)]** | **2** | **Q8NB16** | **Q8NB16 [184-194]** | **Q8NB16 1xSHM_DDL [C184(100)]** | **0** | **1576.775** | **318272632** | **2** | **2.79** |
| **[K].CMQEIPQEQIK.[E]** | **1xOxidation [M2]; 1xSHM_DDL [C1(100)]** | **1** | **Q8NB16** | **Q8NB16 [184-194]** | **Q8NB16 1xSHM_DDL [C184(100)]** | **0** | **1592.7699** | **3966785.8** | **2** | **2.25** |
| **[R].QQEPLGEDCPSELREIIDECR.[A]** | **2xCarbamidomethyl [C9; C20]** | **24** | **Q8NB16** | **Q8NB16 [429-449]** |  | **1** | **2573.161** | **6.35E+08** | **3** | **3.95** |
| **[K].RQQEPLGEDCPSELREIIDECR.[A]** | **2xCarbamidomethyl [C10; C21]** | **8** | **Q8NB16** | **Q8NB16 [428-449]** |  | **2** | **2729.262** | **1.51E+08** | **4** | **5.54** |
| **[R].KTQTSMSLGTTR.[E]** | **1xOxidation [M6]** | **2** | **Q8NB16** | **Q8NB16 [354-365]** |  | **1** | **1326.668** | **28487324** | **3** | **2.79** |
| **[R].RLEINMK.[E]** | **1xOxidation [M6]** | **1** | **Q8NB16** | **Q8NB16 [164-170]** |  | **1** | **919.503** | **6544842** | **2** | **2.06** |
| **[K].TQTSMSLGTTR.[E]** | **1xOxidation [M5]** | **11** | **Q8NB16** | **Q8NB16 [355-365]** |  | **0** | **1198.573** | **1.01E+09** | **2** | **2.44** |
| **[R].MPVSPISQGASWAQEDQQDADEDR.[R]** | **1xOxidation [M1]** | **11** | **Q8NB16** | **Q8NB16 [122-145]** |  | **0** | **2676.148** | **7315739** | **3** | **6.21** |
| **[R].MPVSPISQGASWAQEDQQDADEDRR.[A]** | **1xOxidation [M1]** | **105** | **Q8NB16** | **Q8NB16 [122-146]** |  | **1** | **2832.249** | **4.24E+08** | **3** | **5.14** |
| **[R].MVLVLGAAR.[G]** | **1xOxidation [M1]** | **14** | **Q8NB16** | **Q8NB16 [307-315]** |  | **0** | **945.555** | **1.05E+09** | **2** | **2.29** |
| **[R].VLGLIKPLEMLQDQGK.[R]** | **1xOxidation [M10]** | **13** | **Q8NB16** | **Q8NB16 [35-50]** |  | **0** | **1798.014** | **6.94E+08** | **3** | **4.28** |
| **[R].VLGLIKPLEMLQDQGKR.[S]** | **1xOxidation [M10]** | **1** | **Q8NB16** | **Q8NB16 [35-51]** |  | **1** | **1954.115** |  | **3** | **3.01** |
| **[R].QQEPLGEDCPSELR.[E]** | **1xCarbamidomethyl [C9]** | **17** | **Q8NB16** | **Q8NB16 [429-442]** |  | **0** | **1657.749** | **1.09E+09** | **2** | **3** |
| **[R].QYLPPKCMQEIPQEQIK.[E]** | **1xCarbamidomethyl [C7]** | **1** | **Q8NB16** | **Q8NB16 [178-194]** |  | **1** | **2130.072** |  | **3** | **2.64** |
| **[R].EIIDECR.[A]** | **1xCarbamidomethyl [C6]** | **3** | **Q8NB16** | **Q8NB16 [443-449]** |  | **0** | **934.4299** | **1.19E+09** | **2** | **2.07** |
| **[K].CMQEIPQEQIK.[E]** | **1xCarbamidomethyl [C1]; 1xOxidation [M2]** | **7** | **Q8NB16** | **Q8NB16 [184-194]** |  | **0** | **1419.661** | **3.96E+08** | **2** | **3.05** |
| **[K].CMQEIPQEQIK.[E]** | **1xCarbamidomethyl [C1]** | **8** | **Q8NB16** | **Q8NB16 [184-194]** |  | **0** | **1403.666** | **1.22E+09** | **2** | **2.79** |
| **[K].RQQEPLGEDCPSELR.[E]** | **1xCarbamidomethyl [C10]** | **5** | **Q8NB16** | **Q8NB16 [428-442]** |  | **1** | **1813.85** | **4.03E+08** | **3** | **5.01** |
| **[K].AALEEANGEIEK.[F]** |  | **30** | **Q8NB16** | **Q8NB16 [67-78]** |  | **0** | **1273.627** | **2.88E+09** | **2** | **3.35** |
| **[K].AALEEANGEIEKFSNR.[S]** |  | **2** | **Q8NB16** | **Q8NB16 [67-82]** |  | **1** | **1777.872** | **20387133** | **2** | **3.63** |
| **[R].AHDPSVRPSVDEILK.[K]** |  | **4** | **Q8NB16** | **Q8NB16 [450-464]** |  | **0** | **1662.881** | **3.12E+09** | **3** | **2.62** |
| **[R].AHDPSVRPSVDEILKK.[L]** |  | **1** | **Q8NB16** | **Q8NB16 [450-465]** |  | **1** | **1790.976** | **12545391** | **4** | **3.44** |
| **[R].APVAIKVFK.[K]** |  | **4** | **Q8NB16** | **Q8NB16 [225-233]** |  | **1** | **972.6241** | **17436344** | **2** | **2.6** |
| **[R].APVAIKVFKK.[L]** |  | **1** | **Q8NB16** | **Q8NB16 [225-234]** |  | **2** | **1100.719** | **902121.4** | **2** | **2.25** |
| **[R].EKDLTLGK.[R]** |  | **4** | **Q8NB16** | **Q8NB16 [298-305]** |  | **1** | **903.5146** | **4.51E+08** | **2** | **2.08** |
| **[R].ELLDREK.[D]** |  | **2** | **Q8NB16** | **Q8NB16 [293-299]** |  | **1** | **902.4942** | **5.59E+08** | **2** | **2.1** |
| **[K].ELSLLLQVEQR.[M]** |  | **16** | **Q8NB16** | **Q8NB16 [111-121]** |  | **0** | **1327.758** | **2.67E+08** | **2** | **3.13** |
| **[R].ENEVSTLYK.[G]** |  | **12** | **Q8NB16** | **Q8NB16 [211-219]** |  | **0** | **1082.536** | **2.52E+09** | **2** | **2.29** |
| **[R].ENEVSTLYKGEYHR.[A]** |  | **4** | **Q8NB16** | **Q8NB16 [211-224]** |  | **1** | **1724.824** | **30541929** | **3** | **3.66** |
| **[K].EQLSGSPWILLR.[E]** |  | **7** | **Q8NB16** | **Q8NB16 [199-210]** |  | **0** | **1398.774** | **1.93E+09** | **2** | **2.56** |
| **[K].EQLSGSPWILLRENEVSTLYK.[G]** |  | **1** | **Q8NB16** | **Q8NB16 [199-219]** |  | **1** | **2462.293** | **1822719** | **3** | **4.15** |
| **[K].EQLSGSPWILLRENEVSTLYKGEYHR.[A]** |  | **1** | **Q8NB16** | **Q8NB16 [199-224]** |  | **2** | **3104.58** |  | **4** | **4.67** |
| **[K].FESPNILR.[I]** |  | **2** | **Q8NB16** | **Q8NB16 [257-264]** |  | **0** | **975.5258** | **9.09E+08** | **2** | **2.06** |
| **[R].FKAALEEANGEIEK.[F]** |  | **3** | **Q8NB16** | **Q8NB16 [65-78]** |  | **1** | **1548.79** | **18568438** | **2** | **3.37** |
| **[R].FLTASQDK.[I]** |  | **6** | **Q8NB16** | **Q8NB16 [88-95]** |  | **0** | **909.4676** | **1.73E+09** | **2** | **2.31** |
| **[R].FLTASQDKILFK.[D]** |  | **2** | **Q8NB16** | **Q8NB16 [88-99]** |  | **1** | **1410.799** | **21885250** | **3** | **3.47** |
| **[R].FLTASQDKILFKDVNR.[K]** |  | **1** | **Q8NB16** | **Q8NB16 [88-103]** |  | **2** | **1895.039** | **945949.1** | **3** | **3.02** |
| **[K].HIITLGQVIHK.[R]** |  | **21** | **Q8NB16** | **Q8NB16 [6-16]** |  | **0** | **1258.763** | **1.64E+09** | **3** | **4.08** |
| **[K].HIITLGQVIHKR.[C]** |  | **1** | **Q8NB16** | **Q8NB16 [6-17]** |  | **1** | **1414.864** |  | **3** | **3.51** |
| **[K].KEQLSGSPWILLR.[E]** |  | **7** | **Q8NB16** | **Q8NB16 [198-210]** |  | **1** | **1526.869** | **8.09E+08** | **3** | **2.96** |
| **[K].KEQLSGSPWILLRENEVSTLYK.[G]** |  | **1** | **Q8NB16** | **Q8NB16 [198-219]** |  | **2** | **2590.388** |  | **4** | **3.84** |
| **[K].KFESPNILR.[I]** |  | **17** | **Q8NB16** | **Q8NB16 [256-264]** |  | **1** | **1103.621** | **2.94E+09** | **2** | **2.91** |
| **[K].KLQAGSIAIVR.[Q]** |  | **9** | **Q8NB16** | **Q8NB16 [234-244]** |  | **1** | **1155.721** | **6.47E+08** | **3** | **3.42** |
| **[R].KLSDVWK.[E]** |  | **25** | **Q8NB16** | **Q8NB16 [104-110]** |  | **1** | **875.4985** | **1.55E+09** | **2** | **2.9** |
| **[K].KLSTFSK.[-]** |  | **9** | **Q8NB16** | **Q8NB16 [465-471]** |  | **1** | **810.472** | **5.68E+08** | **2** | **2.66** |
| **[R].KLVAVKR.[Q]** |  | **3** | **Q8NB16** | **Q8NB16 [422-428]** |  | **2** | **813.5669** | **3772606** | **2** | **2.15** |
| **[R].KTQTSMSLGTTR.[E]** |  | **2** | **Q8NB16** | **Q8NB16 [354-365]** |  | **1** | **1310.673** | **1.26E+08** | **3** | **2.92** |
| **[K].LAGFELR.[K]** |  | **6** | **Q8NB16** | **Q8NB16 [347-353]** |  | **0** | **805.4567** | **3E+09** | **2** | **2.25** |
| **[R].LHHSEAPELHGK.[I]** |  | **20** | **Q8NB16** | **Q8NB16 [320-331]** |  | **0** | **1354.686** | **1.13E+09** | **3** | **3.95** |
| **[R].LHHSEAPELHGKIR.[S]** |  | **1** | **Q8NB16** | **Q8NB16 [320-333]** |  | **1** | **1623.871** | **15705511** | **4** | **4.61** |
| **[K].LQAGSIAIVR.[Q]** |  | **10** | **Q8NB16** | **Q8NB16 [235-244]** |  | **0** | **1027.626** | **3.32E+09** | **2** | **2.51** |
| **[K].LTTAMNR.[F]** |  | **3** | **Q8NB16** | **Q8NB16 [58-64]** |  | **0** | **806.4189** | **2.49E+09** | **2** | **1.95** |
| **[R].MPVSPISQGASWAQEDQQDADEDR.[R]** |  | **32** | **Q8NB16** | **Q8NB16 [122-145]** |  | **0** | **2660.153** | **81243088** | **3** | **4.97** |
| **[R].MPVSPISQGASWAQEDQQDADEDRR.[A]** |  | **122** | **Q8NB16** | **Q8NB16 [122-146]** |  | **1** | **2816.254** | **1.11E+09** | **3** | **5.29** |
| **[R].MVLVLGAAR.[G]** |  | **19** | **Q8NB16** | **Q8NB16 [307-315]** |  | **0** | **929.5601** | **2.92E+09** | **2** | **2.94** |
| **[R].QTFNKEIK.[T]** |  | **1** | **Q8NB16** | **Q8NB16 [245-252]** |  | **1** | **1007.552** | **16539412** | **2** | **2.13** |
| **[R].QYLPPK.[C]** |  | **5** | **Q8NB16** | **Q8NB16 [178-183]** |  | **0** | **745.4243** | **3E+08** | **2** | **2.1** |
| **[R].RAFQMLR.[R]** |  | **2** | **Q8NB16** | **Q8NB16 [146-152]** |  | **1** | **921.5087** | **31160052** | **2** | **2.59** |
| **[R].RDNEKIEASLR.[R]** |  | **1** | **Q8NB16** | **Q8NB16 [153-163]** |  | **2** | **1330.707** | **17695658** | **3** | **2.87** |
| **[R].RLEINMK.[E]** |  | **21** | **Q8NB16** | **Q8NB16 [164-170]** |  | **1** | **903.5081** | **2.27E+09** | **2** | **2.48** |
| **[R].RLEINMKEIK.[E]** |  | **2** | **Q8NB16** | **Q8NB16 [164-173]** |  | **2** | **1273.73** | **9007539** | **3** | **3.52** |
| **[K].RMVLVLGAAR.[G]** |  | **2** | **Q8NB16** | **Q8NB16 [306-315]** |  | **1** | **1085.661** | **3935320** | **2** | **2.43** |
| **[K].RSVPSEK.[L]** |  | **1** | **Q8NB16** | **Q8NB16 [51-57]** |  | **1** | **802.4417** | **34873248** | **2** | **2.08** |
| **[R].SSNFLVTQGYQVK.[L]** |  | **20** | **Q8NB16** | **Q8NB16 [334-346]** |  | **0** | **1470.759** | **2.73E+09** | **2** | **3.71** |
| **[R].SSNFLVTQGYQVKLAGFELR.[K]** |  | **1** | **Q8NB16** | **Q8NB16 [334-353]** |  | **1** | **2257.198** | **18855578** | **3** | **5.7** |
| **[R].SSNFLVTQGYQVKLAGFELRK.[T]** |  | **1** | **Q8NB16** | **Q8NB16 [334-354]** |  | **2** | **2385.293** | **3682352** | **4** | **4.48** |
| **[K].STAYLSPQELEDVFYQYDVK.[S]** |  | **11** | **Q8NB16** | **Q8NB16 [373-392]** |  | **0** | **2395.134** | **9.7E+08** | **2** | **4.52** |
| **[R].SVPSEKLTTAMNR.[F]** |  | **1** | **Q8NB16** | **Q8NB16 [52-64]** |  | **1** | **1433.742** | **13086646** | **3** | **3.18** |
| **[K].TQTSMSLGTTR.[E]** |  | **3** | **Q8NB16** | **Q8NB16 [355-365]** |  | **0** | **1182.578** | **2.72E+09** | **2** | **2.54** |
| **[R].VKSTAYLSPQELEDVFYQYDVK.[S]** |  | **1** | **Q8NB16** | **Q8NB16 [371-392]** |  | **1** | **2622.297** | **13331816** | **3** | **4.45** |
| **[R].VLGLIKPLEMLQDQGK.[R]** |  | **6** | **Q8NB16** | **Q8NB16 [35-50]** |  | **0** | **1782.019** | **1.22E+09** | **3** | **4.37** |
| **[R].VLGLIKPLEMLQDQGKR.[S]** |  | **1** | **Q8NB16** | **Q8NB16 [35-51]** |  | **1** | **1938.12** | **2819708** | **3** | **5.4** |

**Supplemental Table 12 Mass spectrometry analysis of the covalent modification site on recombinant MLKL by ATL**

| **ATL with MLKL** | | | | | | | | | |
| --- | --- | --- | --- | --- | --- | --- | --- | --- | --- |
| **Annotated Sequence** | **Modifications** | **# PSMs** | **Master Protein Accessions** | **Positions in Master Proteins** | **Modifications in Master Proteins** | **# Missed Cleavages** | **Abundance: F2: Sample** | **Charge (by Search Engine): Sequest HT** | **XCorr (by Search Engine): Sequest HT** |
| **[R].QQEPLGEDCPSELR.[E]** | **1xSHM_ATL [C9(100)]** | **1** | **Q8NB16** | **Q8NB16 [429-442]** | **Q8NB16 1xSHM_ATL [C437(100)]** | **0** |  | **2** | **2.9** |
| **[K].CMQEIPQEQIK.[E]** | **1xSHM_ATL [C1(100)]** | **2** | **Q8NB16** | **Q8NB16 [184-194]** | **Q8NB16 1xSHM_ATL [C184(100)]** | **0** | **51416318** | **2** | **2.6** |
| **[K].CMQEIPQEQIK.[E]** | **1xOxidation [M2]; 1xSHM_ATL [C1(100)]** | **2** | **Q8NB16** | **Q8NB16 [184-194]** | **Q8NB16 1xSHM_ATL [C184(100)]** | **0** | **32273502** | **2** | **2.84** |
| **[R].QQEPLGEDCPSELREIIDECR.[A]** | **2xCarbamidomethyl [C9; C20]** | **2** | **Q8NB16** | **Q8NB16 [429-449]** |  | **1** | **2551777.5** | **3** | **3.2** |
| **[R].KTQTSMSLGTTR.[E]** | **1xOxidation [M6]** | **4** | **Q8NB16** | **Q8NB16 [354-365]** |  | **1** | **28384073** | **3** | **3.08** |
| **[K].TQTSMSLGTTR.[E]** | **1xOxidation [M5]** | **10** | **Q8NB16** | **Q8NB16 [355-365]** |  | **0** | **3.19E+08** | **2** | **2.37** |
| **[R].MVLVLGAAR.[G]** | **1xOxidation [M1]** | **12** | **Q8NB16** | **Q8NB16 [307-315]** |  | **0** | **7.72E+08** | **2** | **2.37** |
| **[R].SVPSEKLTTAMNR.[F]** | **1xOxidation [M11]** | **1** | **Q8NB16** | **Q8NB16 [52-64]** |  | **1** | **2281600** | **3** | **2.3** |
| **[R].VLGLIKPLEMLQDQGK.[R]** | **1xOxidation [M10]** | **9** | **Q8NB16** | **Q8NB16 [35-50]** |  | **0** | **96022265** | **3** | **3.69** |
| **[R].QQEPLGEDCPSELR.[E]** | **1xCarbamidomethyl [C9]** | **7** | **Q8NB16** | **Q8NB16 [429-442]** |  | **0** | **1.88E+08** | **2** | **2.95** |
| **[R].EIIDECR.[A]** | **1xCarbamidomethyl [C6]** | **3** | **Q8NB16** | **Q8NB16 [443-449]** |  | **0** | **3.01E+08** | **2** | **1.94** |
| **[K].CMQEIPQEQIK.[E]** | **1xCarbamidomethyl [C1]; 1xOxidation [M2]** | **6** | **Q8NB16** | **Q8NB16 [184-194]** |  | **0** | **1.61E+08** | **2** | **2.99** |
| **[K].CMQEIPQEQIK.[E]** | **1xCarbamidomethyl [C1]** | **6** | **Q8NB16** | **Q8NB16 [184-194]** |  | **0** | **2.87E+08** | **2** | **2.87** |
| **[K].CMQEIPQEQIKEIK.[K]** | **1xCarbamidomethyl [C1]** | **1** | **Q8NB16** | **Q8NB16 [184-197]** |  | **1** | **1809598** | **3** | **2.89** |
| **[K].RQQEPLGEDCPSELR.[E]** | **1xCarbamidomethyl [C10]** | **4** | **Q8NB16** | **Q8NB16 [428-442]** |  | **1** | **56345802** | **3** | **4.81** |
| **[K].AALEEANGEIEK.[F]** |  | **24** | **Q8NB16** | **Q8NB16 [67-78]** |  | **0** | **6.64E+08** | **2** | **3.21** |
| **[K].AALEEANGEIEKFSNR.[S]** |  | **1** | **Q8NB16** | **Q8NB16 [67-82]** |  | **1** | **6015714** | **3** | **4.37** |
| **[R].AHDPSVRPSVDEILK.[K]** |  | **3** | **Q8NB16** | **Q8NB16 [450-464]** |  | **0** | **9.06E+08** | **4** | **2.93** |
| **[R].AHDPSVRPSVDEILKK.[L]** |  | **2** | **Q8NB16** | **Q8NB16 [450-465]** |  | **1** | **3290832** | **4** | **3.57** |
| **[R].APVAIKVFK.[K]** |  | **2** | **Q8NB16** | **Q8NB16 [225-233]** |  | **1** | **12849776** | **2** | **2.57** |
| **[K].DLTLGKRMVLVLGAAR.[G]** |  | **1** | **Q8NB16** | **Q8NB16 [300-315]** |  | **2** |  | **3** | **2.35** |
| **[R].ELLDREK.[D]** |  | **1** | **Q8NB16** | **Q8NB16 [293-299]** |  | **1** | **1.1E+08** | **2** | **2.07** |
| **[K].ELSLLLQVEQR.[M]** |  | **13** | **Q8NB16** | **Q8NB16 [111-121]** |  | **0** | **80654178** | **2** | **3.21** |
| **[R].ENEVSTLYK.[G]** |  | **11** | **Q8NB16** | **Q8NB16 [211-219]** |  | **0** | **1.39E+09** | **2** | **2.4** |
| **[R].ENEVSTLYKGEYHR.[A]** |  | **1** | **Q8NB16** | **Q8NB16 [211-224]** |  | **1** | **10071161** | **3** | **3.11** |
| **[K].EQLSGSPWILLR.[E]** |  | **5** | **Q8NB16** | **Q8NB16 [199-210]** |  | **0** | **6.2E+08** | **2** | **2.58** |
| **[K].FESPNILR.[I]** |  | **3** | **Q8NB16** | **Q8NB16 [257-264]** |  | **0** | **4.09E+08** | **2** | **2.13** |
| **[R].FKAALEEANGEIEK.[F]** |  | **1** | **Q8NB16** | **Q8NB16 [65-78]** |  | **1** | **3931509** | **3** | **2.43** |
| **[R].FLTASQDK.[I]** |  | **5** | **Q8NB16** | **Q8NB16 [88-95]** |  | **0** | **1.24E+09** | **2** | **2.29** |
| **[R].FLTASQDKILFK.[D]** |  | **2** | **Q8NB16** | **Q8NB16 [88-99]** |  | **1** | **10864545** | **3** | **3.44** |
| **[R].FLTASQDKILFKDVNR.[K]** |  | **1** | **Q8NB16** | **Q8NB16 [88-103]** |  | **2** | **787724.4** | **3** | **3.16** |
| **[K].HIITLGQVIHK.[R]** |  | **9** | **Q8NB16** | **Q8NB16 [6-16]** |  | **0** | **2.31E+08** | **3** | **4.07** |
| **[K].KEQLSGSPWILLR.[E]** |  | **5** | **Q8NB16** | **Q8NB16 [198-210]** |  | **1** | **1.79E+08** | **3** | **3.42** |
| **[K].KFESPNILR.[I]** |  | **6** | **Q8NB16** | **Q8NB16 [256-264]** |  | **1** | **1.13E+09** | **2** | **2.67** |
| **[K].KLQAGSIAIVR.[Q]** |  | **5** | **Q8NB16** | **Q8NB16 [234-244]** |  | **1** | **3.13E+08** | **2** | **3.92** |
| **[R].KLSDVWK.[E]** |  | **13** | **Q8NB16** | **Q8NB16 [104-110]** |  | **1** | **6.46E+08** | **2** | **2.82** |
| **[K].KLSTFSK.[-]** |  | **6** | **Q8NB16** | **Q8NB16 [465-471]** |  | **1** | **1.66E+08** | **2** | **2.62** |
| **[R].KLVAVKR.[Q]** |  | **1** | **Q8NB16** | **Q8NB16 [422-428]** |  | **2** | **1129318** | **2** | **1.9** |
| **[R].KTQTSMSLGTTR.[E]** |  | **2** | **Q8NB16** | **Q8NB16 [354-365]** |  | **1** | **54190188** | **3** | **2.46** |
| **[K].LAGFELR.[K]** |  | **2** | **Q8NB16** | **Q8NB16 [347-353]** |  | **0** | **1.64E+09** | **2** | **2.01** |
| **[K].LAGFELRKTQTSMSLGTTR.[E]** |  | **1** | **Q8NB16** | **Q8NB16 [347-365]** |  | **2** | **374088.2** | **3** | **2.48** |
| **[R].LHHSEAPELHGK.[I]** |  | **3** | **Q8NB16** | **Q8NB16 [320-331]** |  | **0** | **1.76E+08** | **3** | **3.82** |
| **[R].LHHSEAPELHGKIR.[S]** |  | **1** | **Q8NB16** | **Q8NB16 [320-333]** |  | **1** | **4376094** | **4** | **4.5** |
| **[K].LQAGSIAIVR.[Q]** |  | **3** | **Q8NB16** | **Q8NB16 [235-244]** |  | **0** | **1.65E+09** | **2** | **2.59** |
| **[K].LTTAMNR.[F]** |  | **2** | **Q8NB16** | **Q8NB16 [58-64]** |  | **0** | **1.19E+09** | **2** | **1.95** |
| **[R].MVLVLGAAR.[G]** |  | **14** | **Q8NB16** | **Q8NB16 [307-315]** |  | **0** | **1.72E+09** | **2** | **2.81** |
| **[R].QYLPPK.[C]** |  | **3** | **Q8NB16** | **Q8NB16 [178-183]** |  | **0** | **87639757** | **2** | **2.19** |
| **[R].RAFQMLR.[R]** |  | **2** | **Q8NB16** | **Q8NB16 [146-152]** |  | **1** | **7395220** | **2** | **2.12** |
| **[R].RDNEKIEASLR.[R]** |  | **1** | **Q8NB16** | **Q8NB16 [153-163]** |  | **2** | **8561159** | **3** | **2.92** |
| **[R].RLEINMK.[E]** |  | **10** | **Q8NB16** | **Q8NB16 [164-170]** |  | **1** | **7.98E+08** | **2** | **2.55** |
| **[R].RLEINMKEIK.[E]** |  | **2** | **Q8NB16** | **Q8NB16 [164-173]** |  | **2** | **3585418** | **3** | **3.77** |
| **[K].RMVLVLGAAR.[G]** |  | **1** | **Q8NB16** | **Q8NB16 [306-315]** |  | **1** | **2256642** | **2** | **2.8** |
| **[R].SSNFLVTQGYQVK.[L]** |  | **14** | **Q8NB16** | **Q8NB16 [334-346]** |  | **0** | **1.64E+09** | **2** | **3.28** |
| **[R].SSNFLVTQGYQVKLAGFELR.[K]** |  | **1** | **Q8NB16** | **Q8NB16 [334-353]** |  | **1** |  | **3** | **5.43** |
| **[R].SSNFLVTQGYQVKLAGFELRK.[T]** |  | **1** | **Q8NB16** | **Q8NB16 [334-354]** |  | **2** | **2856948** | **4** | **3.94** |
| **[K].STAYLSPQELEDVFYQYDVK.[S]** |  | **4** | **Q8NB16** | **Q8NB16 [373-392]** |  | **0** | **74294272** | **2** | **4.39** |
| **[R].SVPSEKLTTAMNR.[F]** |  | **2** | **Q8NB16** | **Q8NB16 [52-64]** |  | **1** | **5136628** | **3** | **3.1** |
| **[K].TMKKFESPNILR.[I]** |  | **1** | **Q8NB16** | **Q8NB16 [253-264]** |  | **2** | **912542** | **3** | **2.3** |
| **[R].VKSTAYLSPQELEDVFYQYDVK.[S]** |  | **1** | **Q8NB16** | **Q8NB16 [371-392]** |  | **1** |  | **3** | **2.44** |
| **[R].VLGLIKPLEMLQDQGK.[R]** |  | **3** | **Q8NB16** | **Q8NB16 [35-50]** |  | **0** | **1.2E+08** | **3** | **4.01** |
| **[R].VLGLIKPLEMLQDQGKR.[S]** |  | **1** | **Q8NB16** | **Q8NB16 [35-51]** |  | **1** | **1540786** | **3** | **4.3** |
