# Supplementary material for: Identification of human MLKL Cys184 and HSPBP1 Cys201 as novel cellular targets for necroptosis
Source: Cell Death Dis. 2026 Apr 22;17(1):528. doi: 10.1038/s41419-026-08764-4 (PMC13230738; doi:10.1038/s41419-026-08764-4)
Supplement: Supplementary file 10 — Supplementary Information for 60 TCM Compounds [file 41419_2026_8764_MOESM10_ESM.docx]

| Serial Number | Name | Chemical Formula | Molecular  Weight | Structural  Formula | EC_50_ |
| --- | --- | --- | --- | --- | --- |

|  |  |  | |  | 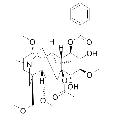 |  |
| --- | --- | --- | --- | --- | --- | --- |
|  |  |  | |  |  |  |
| 1 | Hypaconitine | C33H45NO10 | 615.711 | |  | ＞10μM |
|  |  |  |  | |  |  |
|  |  |  |  | |  |  |
|  |  |  |  | | 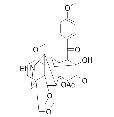 |  |
|  |  |  |  | |  |  |
| 2 | Bulleyaconitine A | C35H49NO9 | 627.765 | |  | ＞10μM |
|  |  |  |  | |  |  |
|  |  |  |  | |  |  |
|  |  |  |  | |  |  |
|  |  |  |  | | 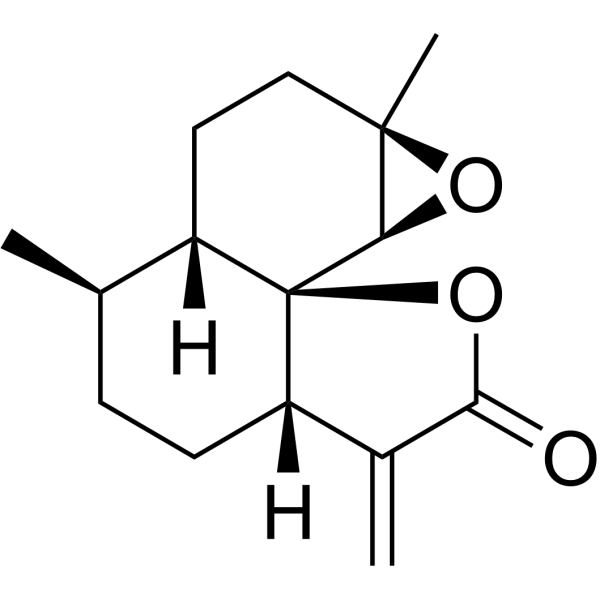 |  |
| 3 | Arteannuin B | C_15_H_20_O_3_ | 248.32 | |  | ＞10μM |
|  |  |  |  | |  |  |
|  |  |  |  | |  |  |
|  |  |  |  | | 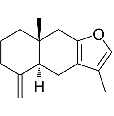 |  |
|  |  |  |  | |  |  |
| 4 | Atractylon | C15H20O | 216.319 | |  | ＞10μM |
|  |  |  |  | |  |  |
|  |  |  |  | |  |  |
|  |  |  |  | | 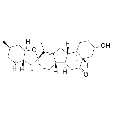 |  |
|  |  |  |  | |  |  |
| 5 | Peimisine | C27H41NO3 | 427.619 | |  | ＞10μM |
|  |  |  |  | |  |  |
|  |  |  |  | | 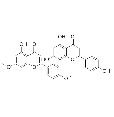 |  |
|  |  |  |  | |  |  |
|  |  |  |  | |  |  |
| 6 | Ginkgetin | C32H22O10 | 566.511 | |  | ＞10μM |
|  |  |  |  | |  |  |
|  |  |  |  | |  |  |
|  |  |  |  | | 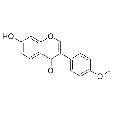 |  |
|  |  |  |  | |  |  |
| 7 | Formononetin | C16H12O4 | 268.264 | |  | ＞10μM |
|  |  |  |  | |  |  |
|  |  |  |  | |  |  |
|  |  |  |  | |  |  |
|  |  |  |  | | 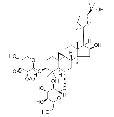 |  |
| 8 | Astragaloside I | C45H72O16 | 869.04 | |  | ＞10μM |
|  |  |  |  | |  |  |
|  |  |  |  | |  |  |
|  |  |  |  | |  |  |
|  |  |  |  | | 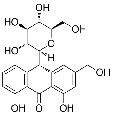 |  |
| 9 | Barbaloin | C21H22O9 | 418.394 | |  | ＞10μM |
|  |  |  |  | |  |  |
|  |  |  |  | |  |  |
|  |  |  |  | | 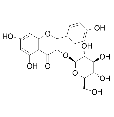 |  |
|  |  |  |  | |  |  |
| 10 | Astragalin | C21H20O11 | 448.377 | |  | ＞10μM |
|  |  |  |  | |  |  |
|  |  |  |  | |  |  |
|  |  |  |  | | 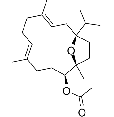 |  |
|  |  |  |  | |  |  |
| 11 | Eugenol acetate | C22H36O3 | 348.519 | |  | ＞10μM |
|  |  |  |  | |  |  |
|  |  |  |  | |  |  |
|  |  |  |  | | 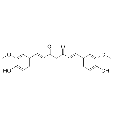 |  |
|  |  |  |  | |  |  |
| 12 | Curcumin | C21H20O6 | 368.38 | |  | ＞10μM |
|  |  |  |  | |  |  |
|  |  |  |  | |  |  |
|  |  |  |  | | 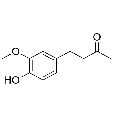 |  |
|  |  |  |  | |  |  |
| 13 | Zingerone | C11H14O3 | 194.227 | |  | ＞10μM |
|  |  |  |  | |  |  |
|  |  |  |  | |  |  |
|  |  |  |  | | 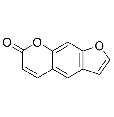 |  |
|  |  |  |  | |  |  |
| 14 | Psoralen | C11H6O3 | 186.163 | |  | ＞10μM |
|  |  |  |  | |  |  |
|  |  |  |  | |  |  |
|  |  |  |  | | 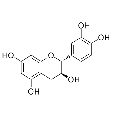 |  |
|  |  |  |  | |  |  |
| 15 | Catechin | C15H14O6 | 290.268 | |  | ＞10μM |
|  |  |  |  | |  |  |
|  |  |  |  | |  |  |
|  |  |  |  | |  |  |
|  |  |  |  | | 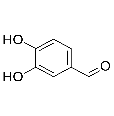 |  |
| 16 | Protocatechuic  aldehyde | C7H6O3 | 138.12 | |  | ＞10μM |
|  |  |  |  | |  |  |
|  |  |  |  | |  |  |
|  |  |  |  | | 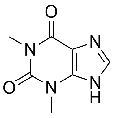 |  |
|  |  |  |  | |  |  |
| 17 | Theophylline | C7H8N4O2 | 180.164 | |  | ＞10μM |
|  |  |  |  | |  |  |
|  |  |  |  | |  |  |
|  |  |  |  | | 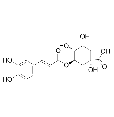 |  |
|  |  |  |  | |  |  |
| 18 | Chlorogenic acid | C16H18O9 | 354.309 | |  | ＞10μM |
|  |  |  |  | |  |  |
|  |  |  |  | |  |  |
|  |  |  |  | | 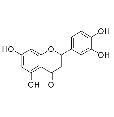 |  |
|  |  |  |  | |  |  |
| 19 | Luteolin | C15H10O6 | 286.236 | |  | ＞10μM |
|  |  |  |  | |  |  |
|  |  |  |  | |  |  |
|  |  |  |  | |  |  |
|  |  |  |  | | 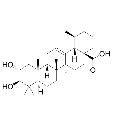 |  |
|  |  |  |  | |  |  |
| 20 | Corosolic acid | C30H48O4 | 472.7 | |  | ＞10μM |
|  |  |  |  | |  |  |
|  |  |  |  | |  |  |
|  |  |  |  | | 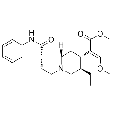 |  |
|  |  |  |  | |  |  |
| 21 | Rhyacophylline | C22H28N2O4 | 384.469 | |  | ＞10μM |
|  |  |  |  | |  |  |
|  |  |  |  | |  |  |
|  |  |  |  | | 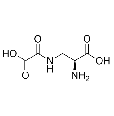 |  |
|  |  |  |  | |  |  |
| 22 | Dencichine | C5H8N2O5 | 176.127 | |  | ＞10μM |
|  |  |  |  | |  |  |
|  |  |  |  | |  |  |
|  |  |  |  | | 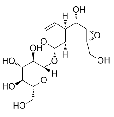 |  |
|  |  |  |  | |  |  |
| 23 | Catalpol | C15H22O10 | 362.329 | |  | ＞10μM |
|  |  |  |  | |  |  |
|  |  |  |  | |  |  |
|  |  |  |  | | 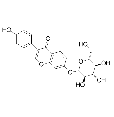 |  |
|  |  |  |  | |  |  |
| 24 | Daidzin | C21H20O9 | 416.378 | |  | ＞10μM |
|  |  |  |  | |  |  |
|  |  |  |  | |  |  |
|  |  |  |  | |  |  |
|  |  |  |  | |  |  |
| 25 | Genistein | C15H10O5 | 270.237 | | 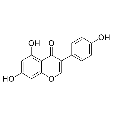 | ＞10μM |
|  |  |  |  | |  |  |
|  |  |  |  | |  |  |
|  |  |  |  | | 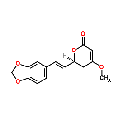 |  |
|  |  |  |  | |  |  |
| 26 | Pellitorine | C15H14O5 | 274.269 | |  | ＞10μM |
|  |  |  |  | |  |  |
|  |  |  |  | |  |  |
|  |  |  |  | | 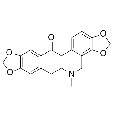 |  |
|  |  |  |  | |  |  |
| 27 | Protopine | C20H19NO5 | 353.369 | |  | ＞10μM |
|  |  |  |  | |  |  |
|  |  |  |  | |  |  |
|  |  |  |  | | 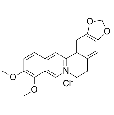 |  |
|  |  |  |  | |  |  |
| 28 | Berberine Hydrochloride | C20H18ClNO4 | 372.822 | |  | ＞10μM |
|  |  |  |  | |  |  |
|  |  |  |  | |  |  |
|  |  |  |  | | 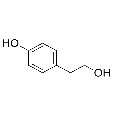 |  |
|  |  |  |  | |  |  |
| 29 | p-Hydroxyphenylethanol | C8H10O2 | 138.16 | |  | ＞10μM |
|  |  |  |  | |  |  |
|  |  |  |  | |  |  |
|  |  |  |  | | 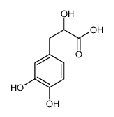 |  |
|  |  |  |  | |  |  |
| 30 | Danshensu lactate | C9H10O5 | 198.173 | |  | ＞10μM |
|  |  |  |  | |  |  |
|  |  |  |  | |  |  |
|  |  |  |  | | 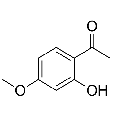 |  |
|  |  |  |  | |  |  |
| 31 | Paeonol | C9H10O3 | 166.174 | |  | ＞10μM |
|  |  |  |  | |  |  |
|  |  |  |  | |  |  |
|  |  |  |  | | 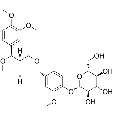 |  |
|  |  |  |  | |  |  |
| 32 | Forsythin | C27H34O11 | 534.552 | |  | ＞10μM |
|  |  |  |  | |  |  |
|  |  |  |  | |  |  |
|  |  |  |  | | 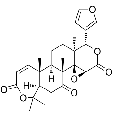 |  |
|  |  |  |  | |  |  |
| 33 | Obacunone | C26H30O7 | 454.512 | |  | ＞10μM |
|  |  |  |  | |  |  |
|  |  |  |  | |  |  |
|  |  |  |  | | 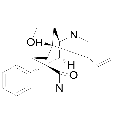 |  |
|  |  |  |  | |  |  |
| 34 | Gelsemine | C20H22N2O2 | 322.401 | |  | ＞10μM |
|  |  |  |  | |  |  |
|  |  |  |  | |  |  |
|  |  |  | 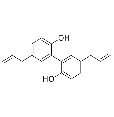 | |  |  |
|  |  |  |  | |  |  |
| 35 | Magnolol | C18H18O2 | 266.334 | |  | ＞10μM |
|  |  |  |  | |  |  |
|  |  |  |  | |  |  |
|  |  |  |  | |  |  |
|  |  |  |  | | 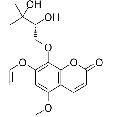 |  |
| 36 | Byakangelicin | C17H18O7 | 334.321 | |  | ＞10μM |
|  |  |  |  | |  |  |
|  |  |  |  | |  |  |
|  |  |  |  | |  |  |
|  |  |  |  | | 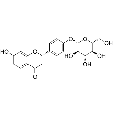 |  |
| 37 | Liquiritin | C21H22O9 | 418.39 | |  | ＞10μM |
|  |  |  |  | |  |  |
|  |  |  |  | |  |  |
|  |  |  |  | | 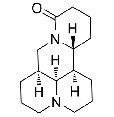 |  |
|  |  |  |  | |  |  |
| 38 | Matrine | C15H24N2O | 248.364 | |  | ＞10μM |
|  |  |  |  | |  |  |
|  |  |  |  | |  |  |
|  |  |  |  | | 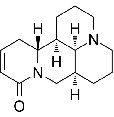 |  |
|  |  |  |  | |  |  |
| 39 | Sophocarpine | C15H22N2O | 246.348 | |  | ＞10μM |
|  |  |  |  | |  |  |
|  |  |  |  | |  |  |
|  |  |  |  | | 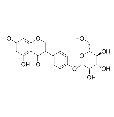 |  |
|  |  |  |  | |  |  |
| 40 | Sophoricoside | C21H20O10 | 432.378 | |  | ＞10μM |
|  |  |  |  | |  |  |
|  |  |  |  | |  |  |
|  |  |  |  | | 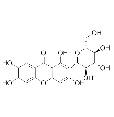 |  |
|  |  |  |  | |  |  |
| 41 | Mangiferin | C19H18O11 | 422.34 | |  | ＞10μM |
|  |  |  |  | |  |  |
|  |  |  |  | |  |  |
|  |  |  |  | |  |  |
|  |  |  |  | |  |  |
| 42 | Polydatin | C20H22O8 | 390.384 | | 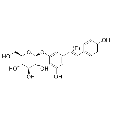 | ＞10μM |
|  |  |  |  | |  |  |
|  |  |  |  | |  |  |
|  |  |  |  | | 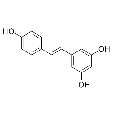 |  |
|  |  |  |  | |  |  |
| 43 | Resveratrol | C14H12O3 | 228.243 | |  | ＞10μM |
|  |  |  |  | |  |  |
|  |  |  |  | |  |  |
|  |  |  |  | | 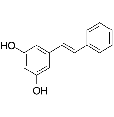 |  |
|  |  |  |  | |  |  |
| 44 | Pinosylvin | C14H12O2 | 212.244 | |  | ＞10μM |
|  |  |  |  | |  |  |
|  |  |  |  | |  |  |
|  |  |  |  | | 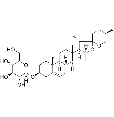 |  |
|  |  |  |  | |  |  |
| 45 | Diosmin | C33H52O8 | 576.761 | |  | ＞10μM |
|  |  |  |  | |  |  |
|  |  |  |  | |  |  |
|  |  |  |  | | 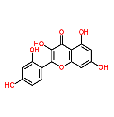 |  |
|  |  |  |  | |  |  |
| 46 | Morin | C15H10O7 | 302.236 | |  | ＞10μM |
|  |  |  |  | |  |  |
|  |  |  |  | |  |  |
|  |  |  |  | | 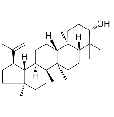 |  |
|  |  |  |  | |  |  |
| 47 | Lupeol | C30H50O | 426.717 | |  | ＞10μM |
|  |  |  |  | |  |  |
|  |  |  |  | |  |  |
|  |  |  |  | |  |  |
|  |  |  |  | | 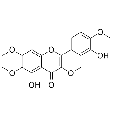 |  |
| 48 | Vitexin | C19H18O8 | 374.341 | |  | ＞10μM |
|  |  |  |  | |  |  |
|  |  |  |  | |  |  |
|  |  |  |  | |  |  |
|  |  |  |  | | 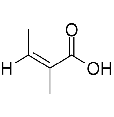 |  |
| 49 | Angelic acid | C5H8O2 | 100.116 | |  | ＞10μM |
|  |  |  |  | |  |  |
|  |  |  |  | |  |  |
|  |  |  |  | |  |  |
|  |  |  |  | | 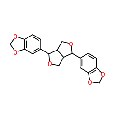 |  |
| 50 | Asarinin | C20H18O6 | 354.353 | |  | ＞10μM |
|  |  |  |  | |  |  |
|  |  |  |  | |  |  |
|  |  |  |  | | 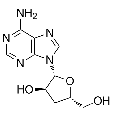 |  |
|  |  |  |  | |  |  |
| 51 | Cordycepin | C10H13N5O3 | 251.242 | |  | ＞10μM |
|  |  |  |  | |  |  |
|  |  |  |  | |  |  |
|  |  |  |  | | 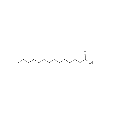 |  |
|  |  |  |  | |  |  |
| 52 | Myristic acid | C14H28O2 | 228.371 | |  | ＞10μM |
|  |  |  |  | |  |  |
|  |  |  |  | |  |  |
|  |  |  | 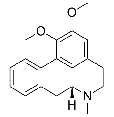 | |  |  |
|  |  |  |  | |  |  |
| 53 | Nuciferine | C19H21NO2 | 295.375 | |  | ＞10μM |
|  |  |  |  | |  |  |
|  |  |  |  | |  |  |
|  |  |  |  | | 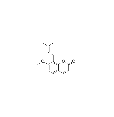 |  |
|  |  |  |  | |  |  |
| 54 | Osthole | C15H16O3 | 244.286 | |  | ＞10μM |
|  |  |  |  | |  |  |
|  |  |  |  | |  |  |
|  |  |  |  | | 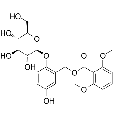 |  |
|  |  |  |  | |  |  |
| 55 | Curculigoside | C22H26O11 | 466.435 | |  | ＞10μM |
|  |  |  |  | |  |  |
|  |  |  |  | |  |  |
|  |  |  |  | | 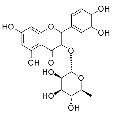 |  |
|  |  |  |  | |  |  |
| 56 | Quercitrin | C21H20O11 | 448.377 | |  | ＞10μM |
|  |  |  |  | |  |  |
|  |  |  |  | |  |  |
|  |  |  |  | | 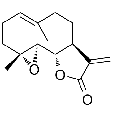 |  |
|  |  |  |  | |  |  |
| 57 | Parthenolide | C15H20O3 | 248.318 | |  | 6μM |
|  |  |  |  | |  |  |
|  |  |  |  | |  |  |
|  |  |  |  | | 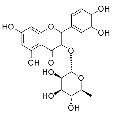 |  |
|  |  |  |  | |  |  |
| 58 | Quercitrin | C21H20O11 | 448.377 | |  | ＞10μM |
|  |  |  |  | |  |  |
|  |  |  |  | |  |  |
|  |  |  |  | |  |  |
|  |  |  |  | | 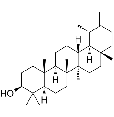 |  |
| 59 | Taraxasterol | C30H50O | 426.717 | |  | ＞10μM |
|  |  |  |  | |  |  |
|  |  |  |  | |  |  |
|  |  |  |  | | 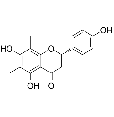 |  |
|  |  |  |  | |  |  |
| 60 | Farrerol | C17H16O5 | 300.306 | |  | ＞10μM |
|  |  |  |  | |  |  |
|  |  |  |  | |  |  |
|  |  |  |  | |  |  |
