# Supplementary material for: Identification of human MLKL Cys184 and HSPBP1 Cys201 as novel cellular targets for necroptosis
Source: Cell Death Dis. 2026 Apr 22;17(1):528. doi: 10.1038/s41419-026-08764-4 (PMC13230738; doi:10.1038/s41419-026-08764-4)
Supplement: Supplementary file 12 — Biological Experiment Replicate Graph [file 41419_2026_8764_MOESM12_ESM.docx]

**Biological replications for review**

**Repeat Figure 1**

**
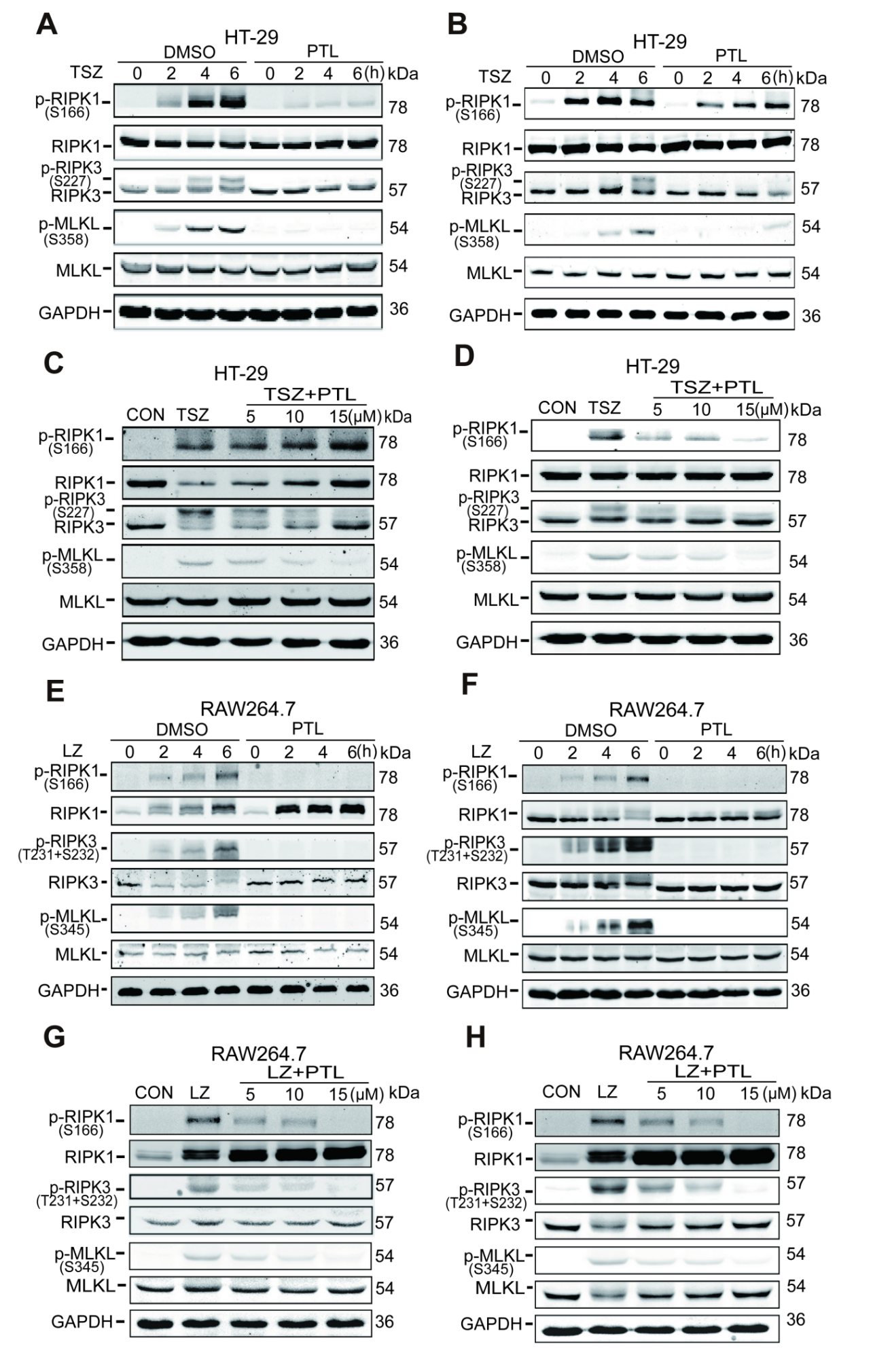
**

**Repeat Figure 1. Immunoblotting of PTL in HT-29 and RAW264.7 cells.**

(A-B) The effect of PTL on necroptosis pathway at different time points in HT-29 cells. TSZ model was induced in HT-29 cells with or without PTL (15 μM) for the indicated time.

(C-D) The effect of different concentrations of PTL on necroptosis pathway in HT-29 cells. TSZ model was induced in HT-29 cells with indicated concentrations of PTL for 6 h.

(E-F) The effect of PTL on necroptosis pathway at different time points in RAW264.7 cells. LZ model was induced in RAW264.7 cells with or without PTL (15 μM) for the indicated time.

(G-H) The effect of different concentrations of PTL on necroptosis pathway in RAW264.7 cells. LZ model was induced in RAW264.7 cells with indicated concentrations of PTL for 6 h.

**Repeat Figure 2**


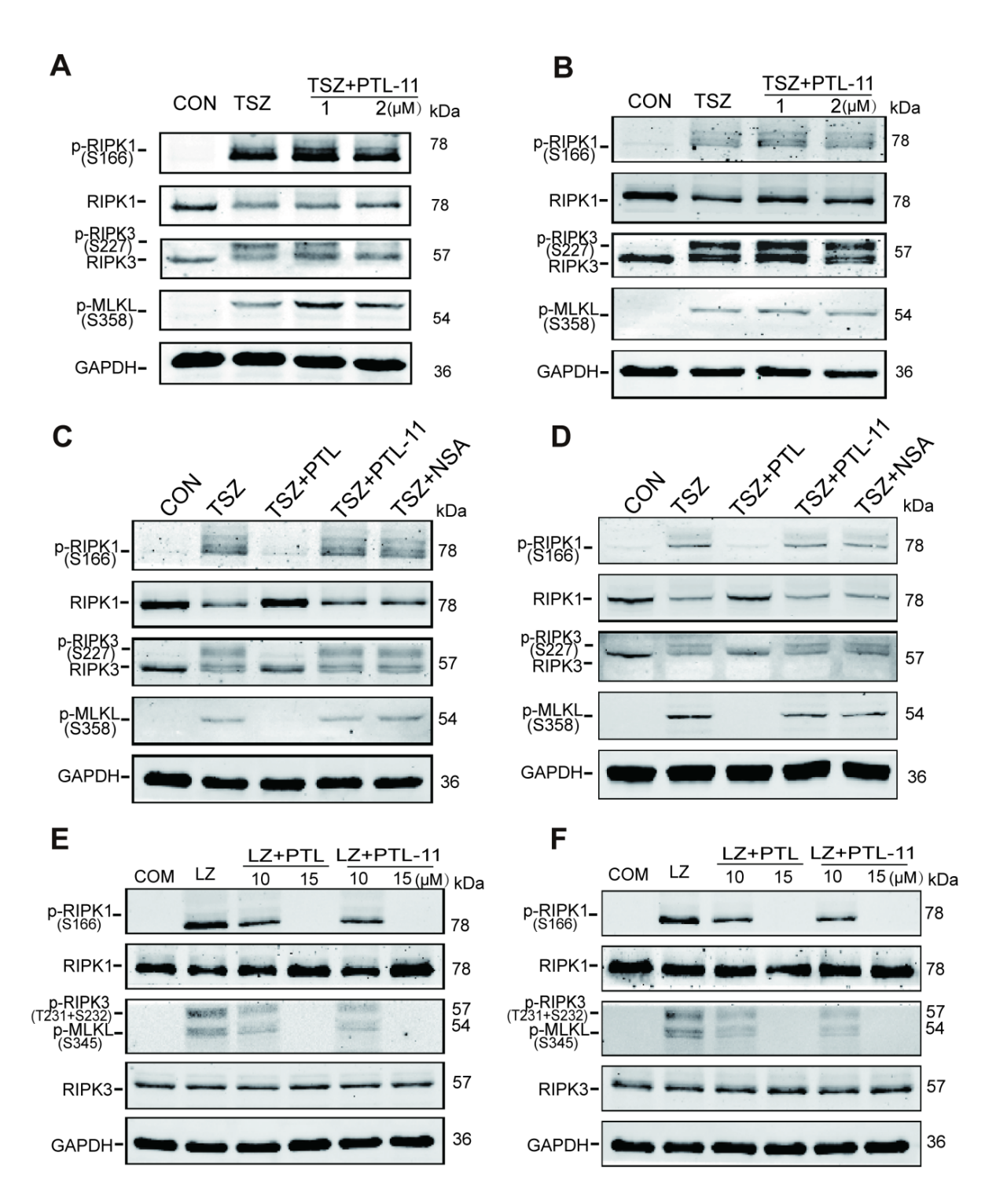


**Repeat Figure 2. Analysis of PTL-11 effects on the necroptotic pathway.**

(**A-B**) The effect of PTL-11 on necroptosis pathway in HT-29 cells at the indicated concentrations.

(**C-D**) The different effects of PTL, PTL-11 and NSA on necroptosis pathway in HT-29 cells.

(**E-F**) The effects of PTL and PTL-11 on necroptosis pathway in RAW264.7 cells at the indicated concentrations.

**Repeat Figure 3**


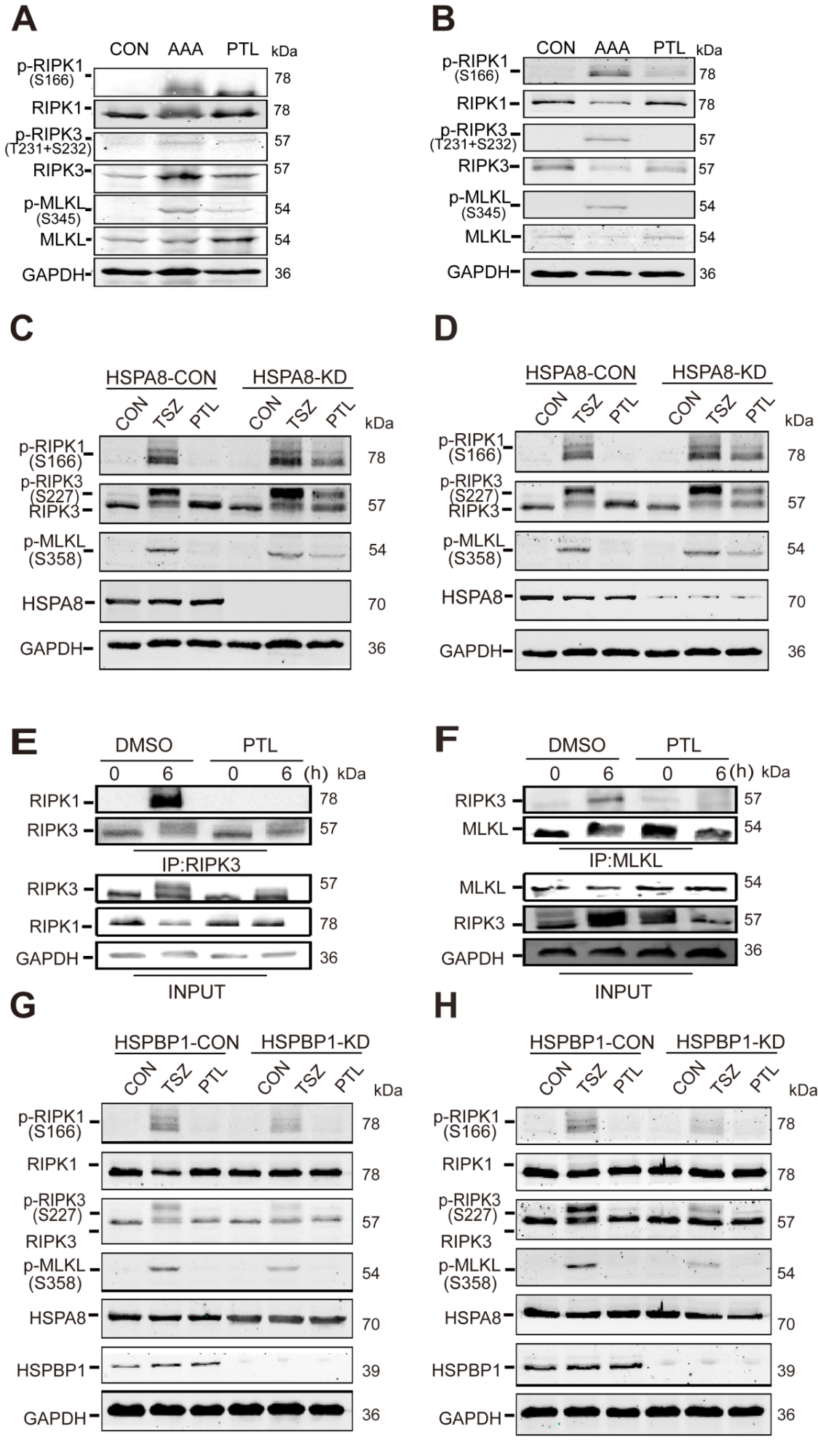


**Repeat Figure 3. Immunoblotting analyses of necroptosis signal pathway in aortic tissue of mice ,immunoblotting analyses of necroptosis signal pathway in HSPBA8-KD and HSPBA8-CON cells and HSPBP1-KD and HSPBP1-CON cells.**

(**A-B**) Immunoblotting analyses of necroptosis signal pathway in aortic tissue of mice.

(**C-D**) Immunoblotting analyses of necroptosis signal pathway in HSPBA8-KD and HSPBA8-CON cells.

(**E**) Interaction between RIPK3 and RIPK1 was detected by co-immunoprecipitation and immunoblotting in cultured HT-29 cells with indicated treatments.

(**F**) Interaction between MLKL and RIPK3 was detected by co-immunoprecipitation and immunoblotting in cultured HT-29 cells with indicated treatments.

(**G-H**)Immunoblotting analyses of necroptosis signal pathway in HSPBP1-KD and HSPBP1-CON cells.

**Repeat Figure 4**


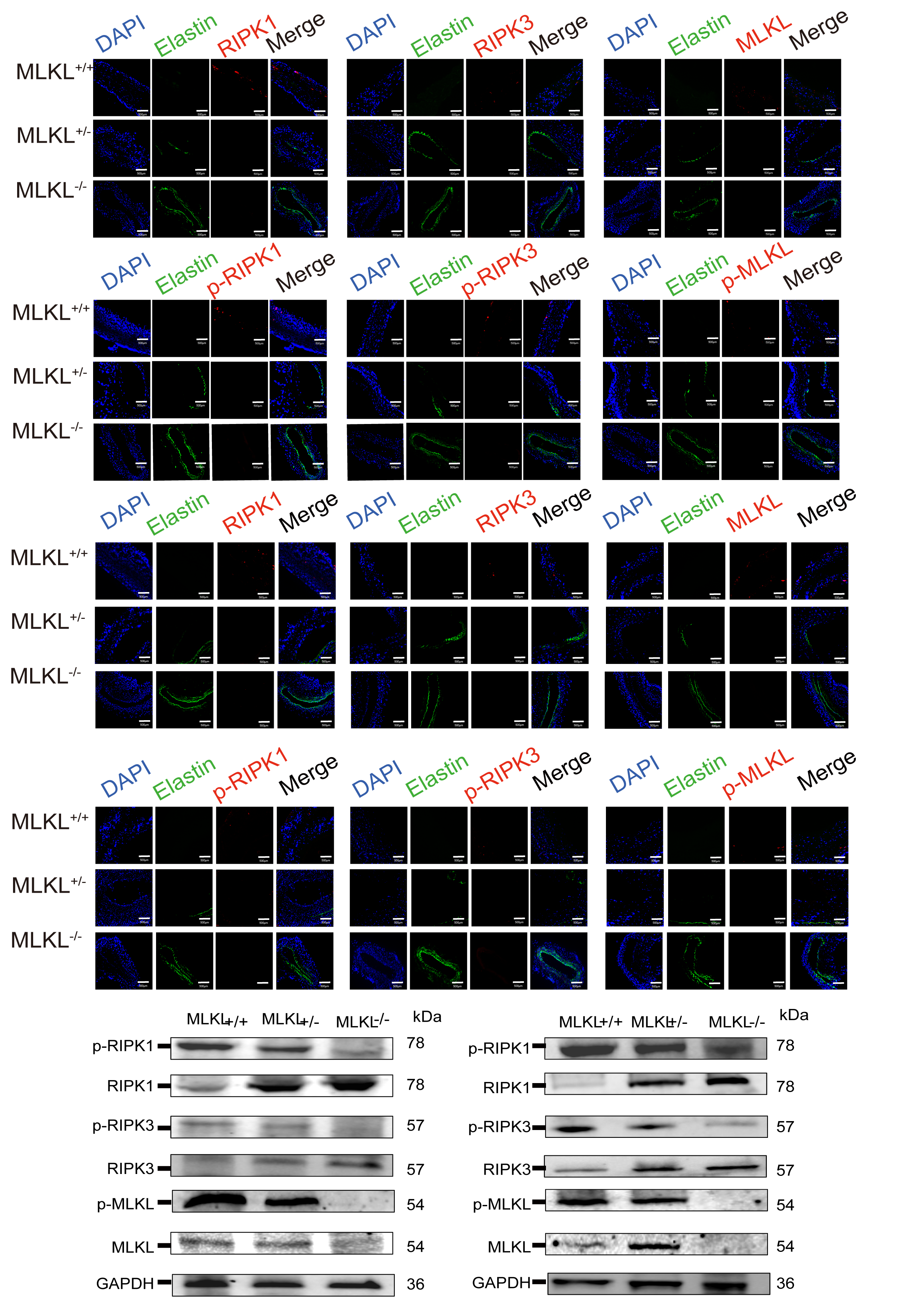


**Repeat Figure 4. Immunoblotting and Immunofluorescence analyses of RIPK1, RIPK3, MLKL p-RIPK1, p-RIPK3 and p-MLKL, in aortic tissue in MLKL deficiency mice.**
