# Supplementary material for: Identification of human MLKL Cys184 and HSPBP1 Cys201 as novel cellular targets for necroptosis
Source: Cell Death Dis. 2026 Apr 22;17(1):528. doi: 10.1038/s41419-026-08764-4 (PMC13230738; doi:10.1038/s41419-026-08764-4)

Fig 2J

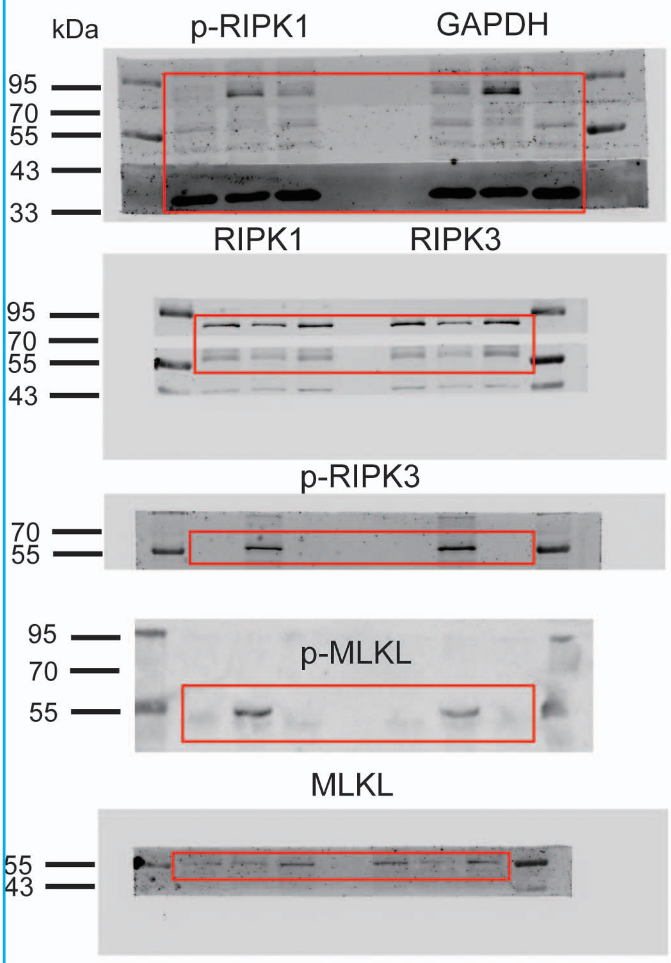

The samples were blotted on the same membrane. GAPDH was run as loading control. Red boxes indicate how the membrane were cropped for the final figure.

Fig 3D

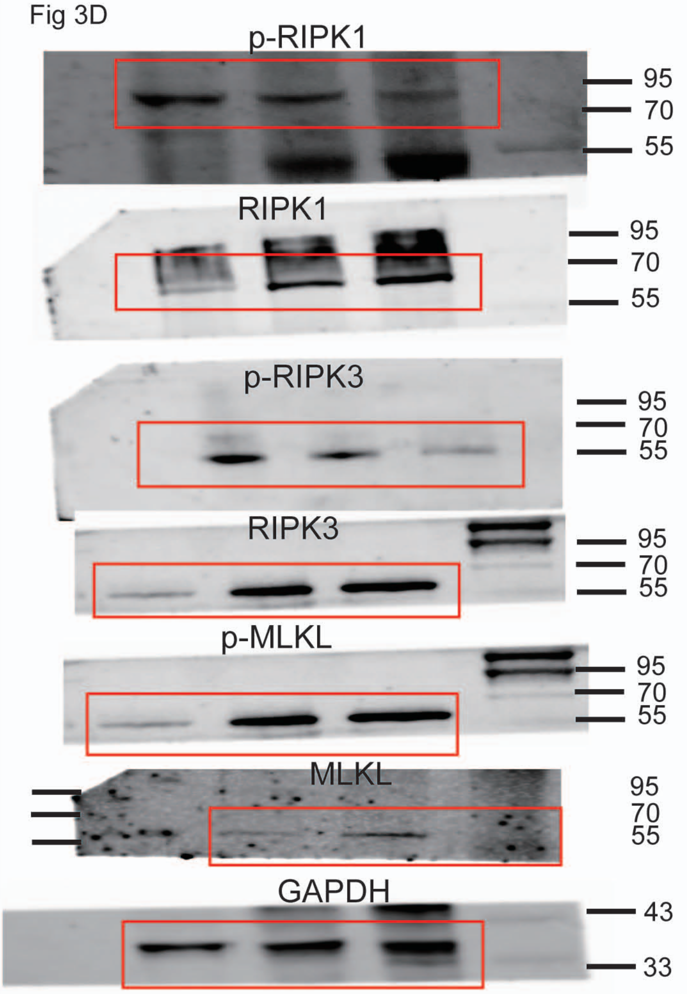

The samples were blotted on the same membrane. GAPDH was run as loading control. Red boxes indicate how the membrane were cropped for the final figure.

Fig 4A

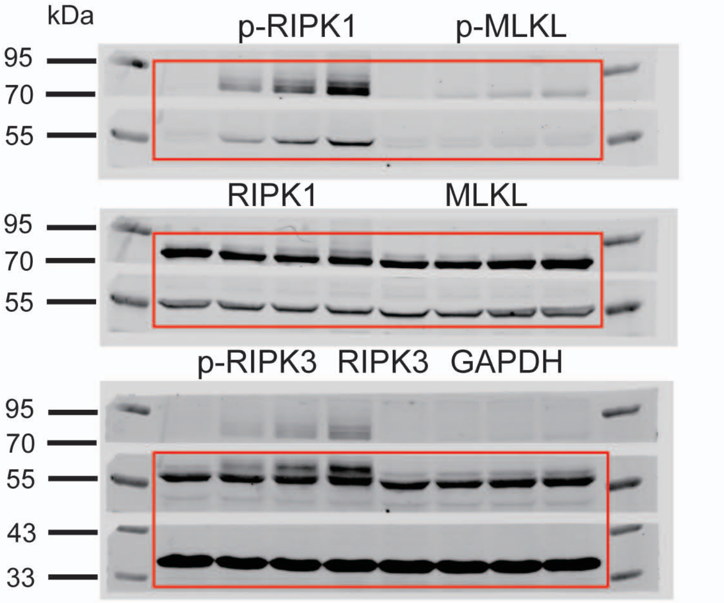

The samples were blotted on the same membrane. GAPDH was run as loading control. Red boxes indicate how the membrane were cropped for the final figure.

Fig 4B

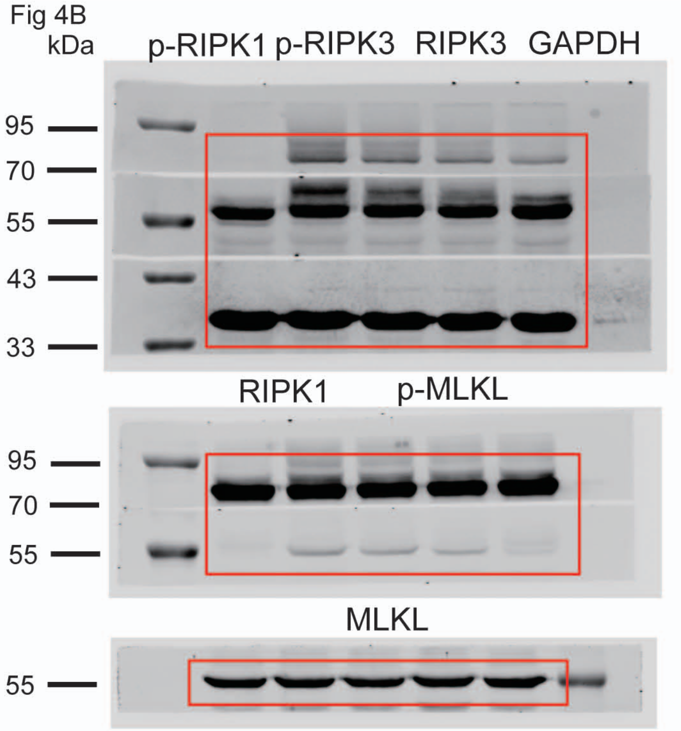

The samples were blotted on the same membrane. GAPDH was run as loading control. Red boxes indicate how the membrane were cropped for the final figure.

Fig 4C

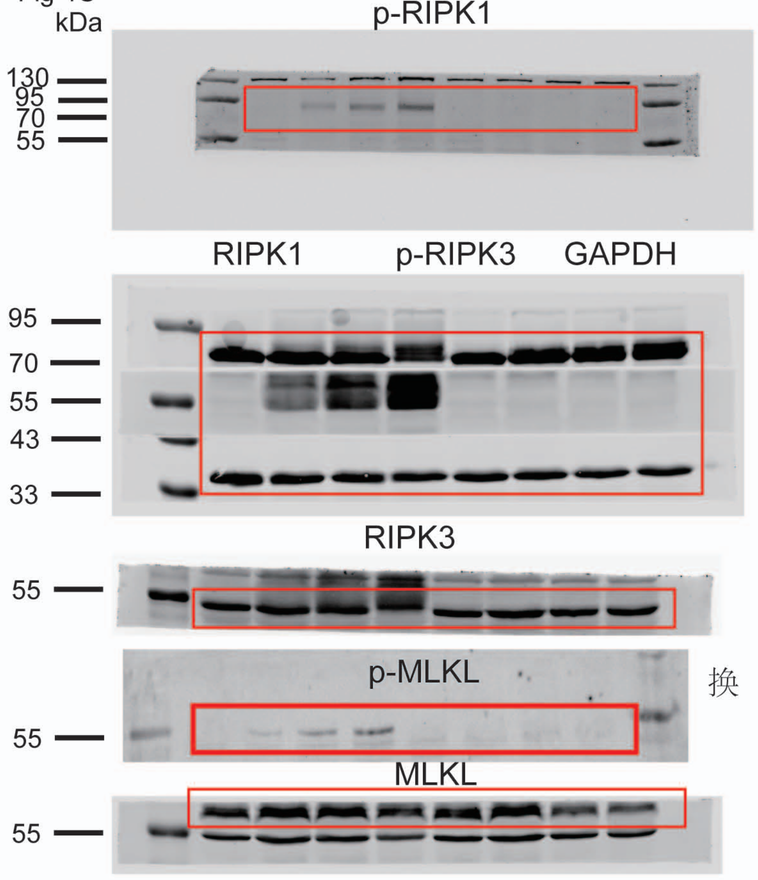

The samples were blotted on the same membrane. GAPDH was run as loading control. Red boxes indicate how the membrane were cropped for the final figure.

Fig 4D

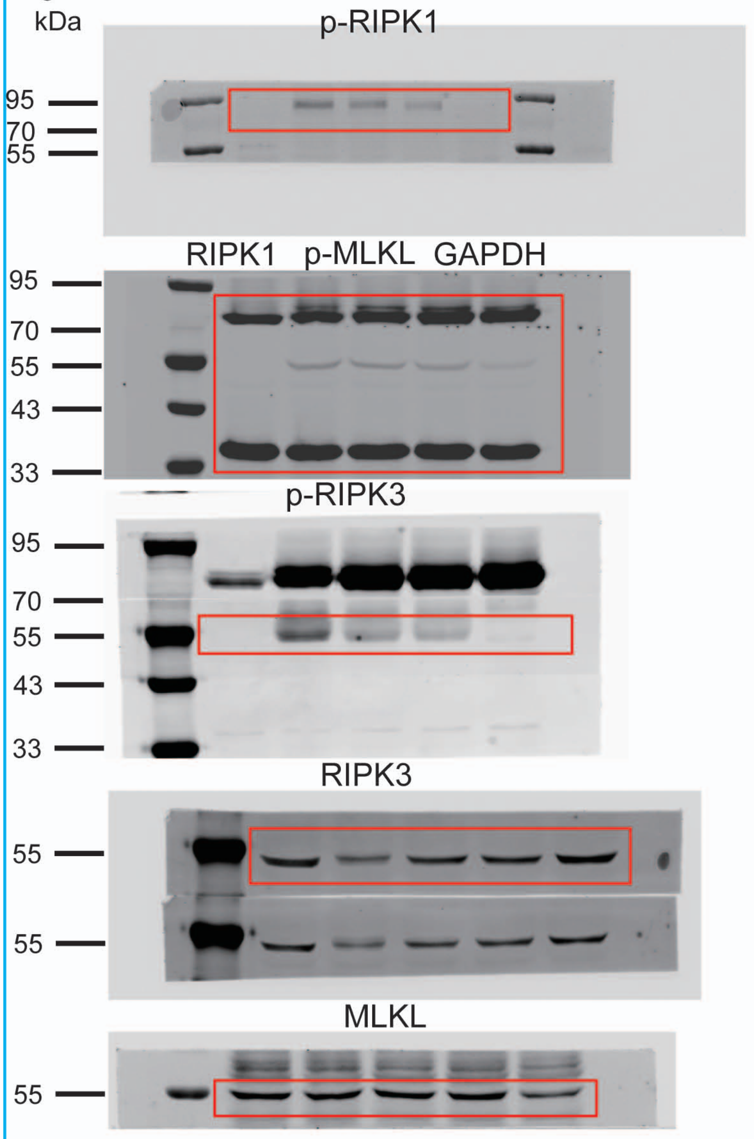

The samples were blotted on the same membrane. GAPDH was run as loading control. Red boxes indicate how the membrane were cropped for the final figure.

Fig 4E

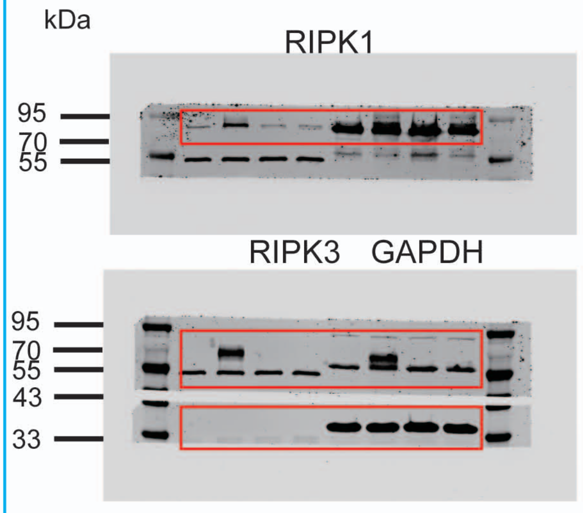

Fig 4F

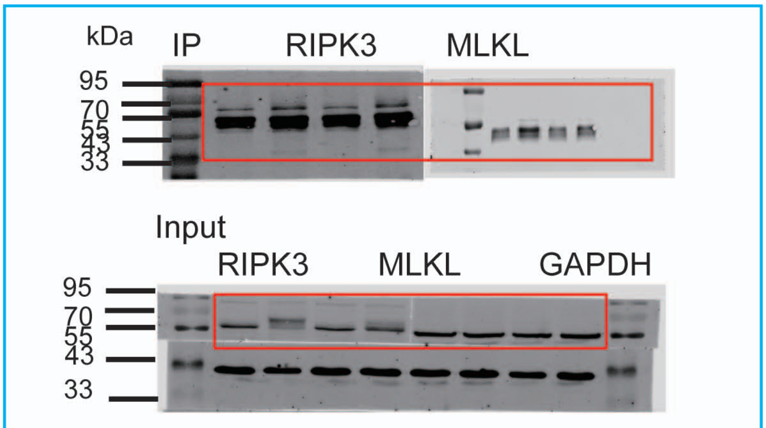

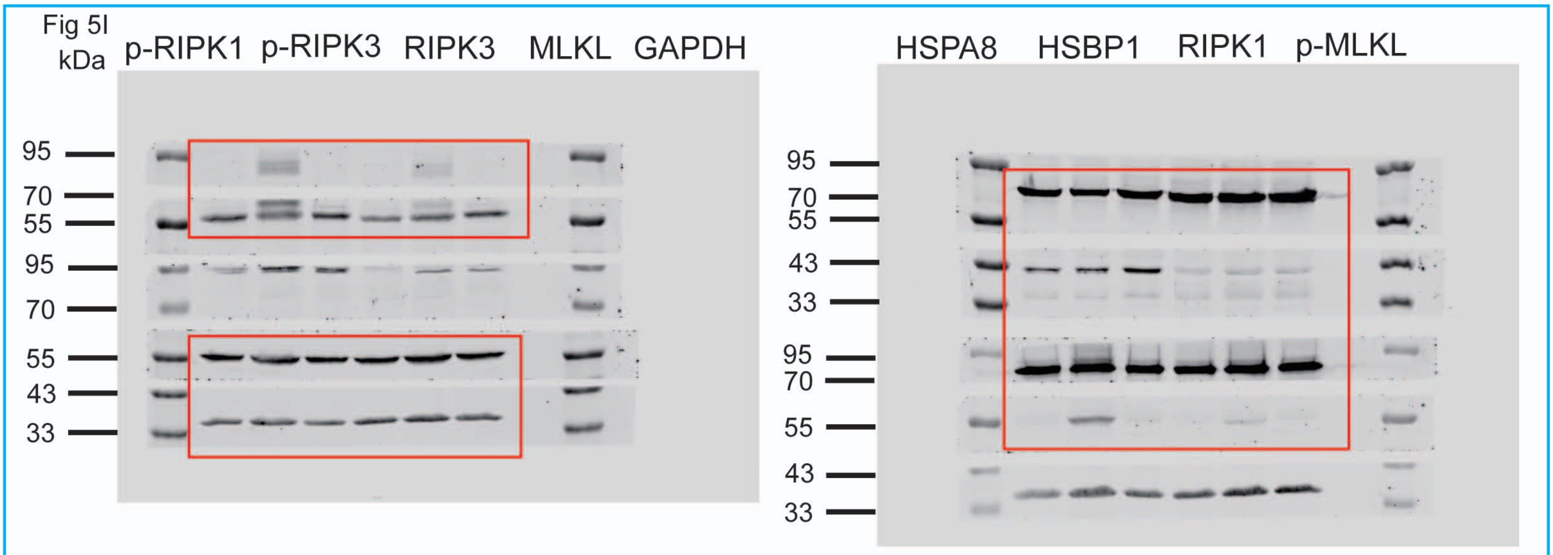

The samples were blotted on the same membrane. GAPDH was run as loading control. Red boxes indicate how the membrane were cropped for the final figure.

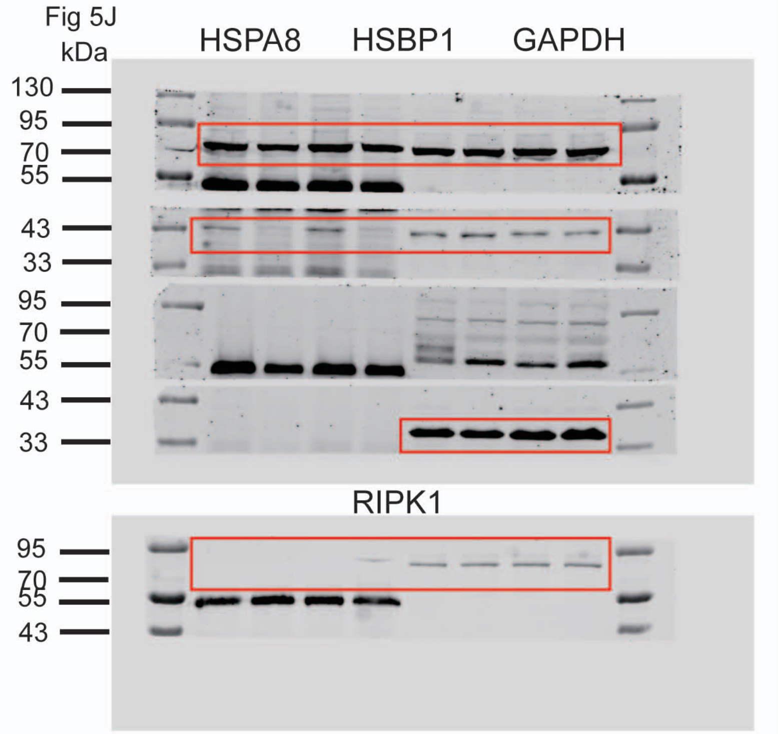

The samples were blotted on the same membrane. GAPDH was run as loading control. Red boxes indicate how the membrane were cropped for the final figure.

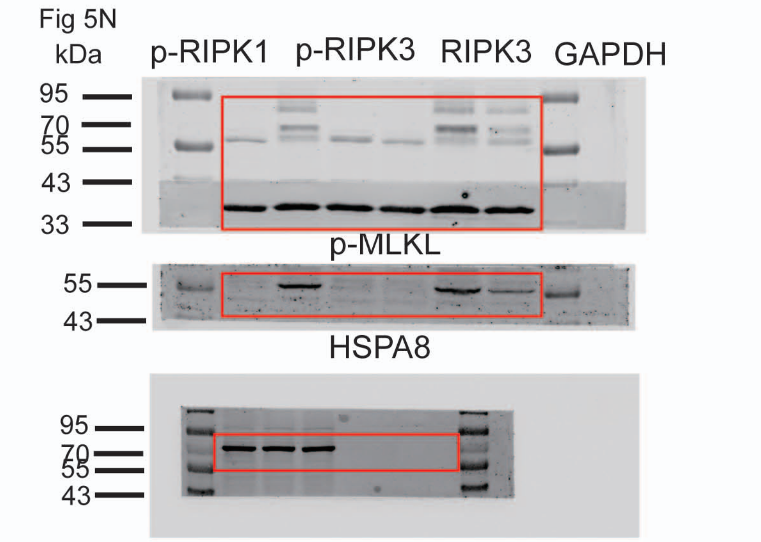

The samples were blotted on the same membrane. GAPDH was run as loading control. Red boxes indicate how the membrane were cropped for the final figure.

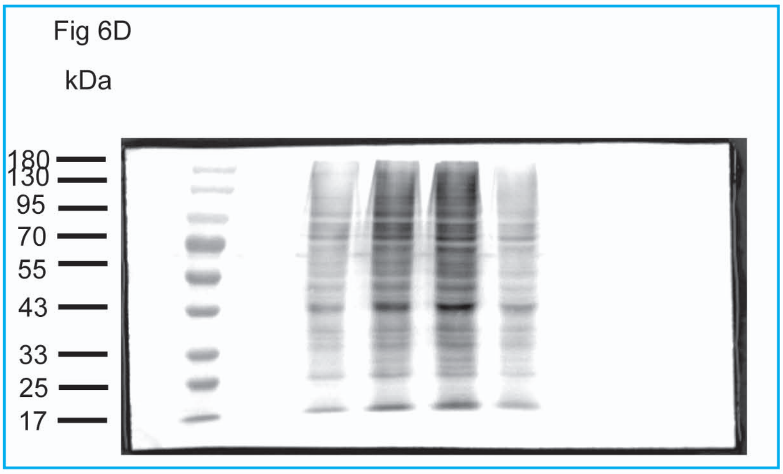

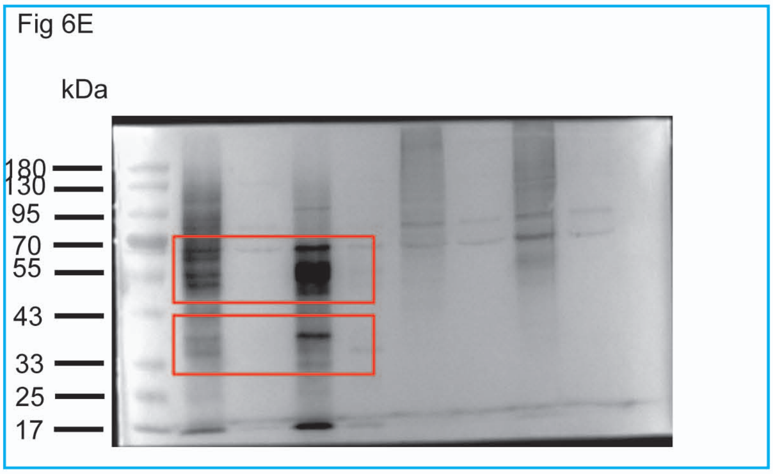

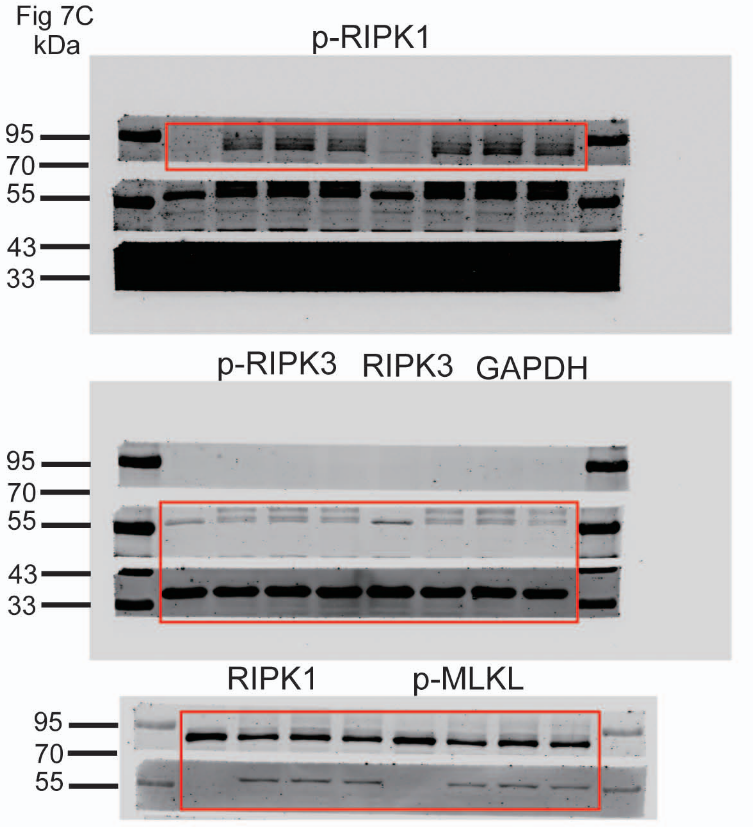

The samples were blotted on the same membrane. GAPDH was run as loading control. Red boxes indicate how the membrane were cropped for the final figure.

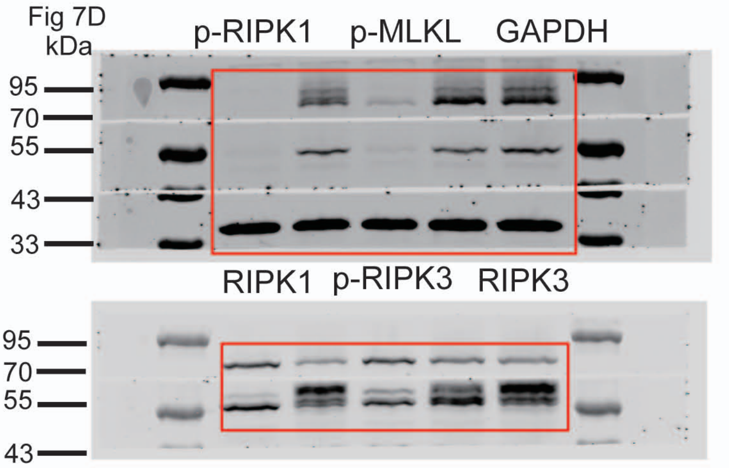

The samples were blotted on the same membrane. GAPDH was run as loading control. Red boxes indicate how the membrane were cropped for the final figure.

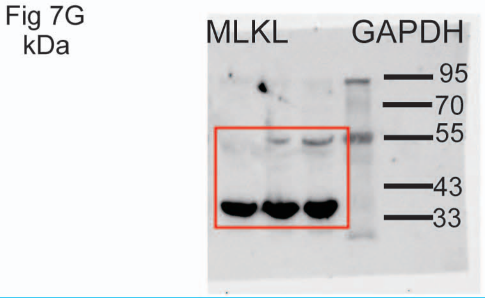

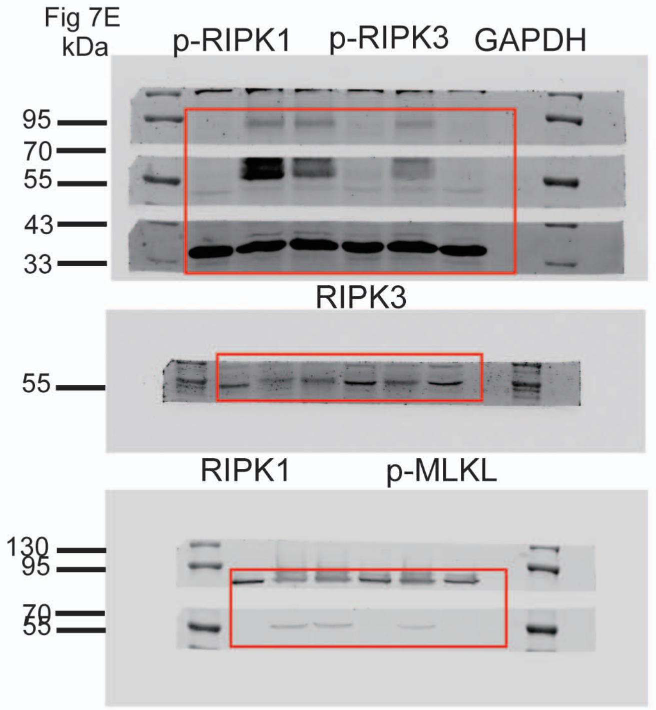

The samples were blotted on the same membrane. GAPDH was run as loading control. Red boxes indicate how the membrane were cropped for the final figure.

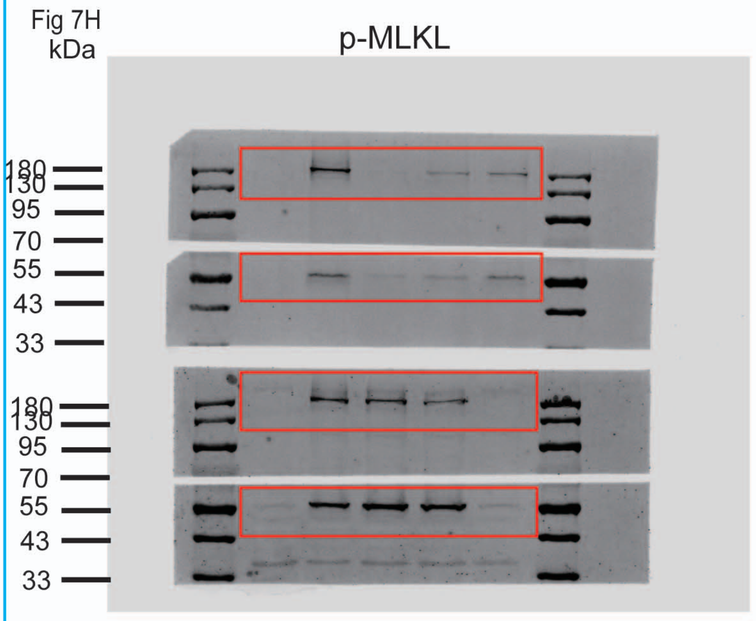

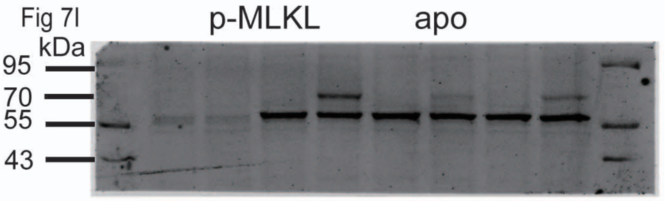

Supplement: Supplementary file 13 — Original Western Blot Images [file 41419_2026_8764_MOESM13_ESM.pdf]
